# Proteogenomic identification of an immunogenic antigen derived from human endogenous retrovirus in renal cell carcinoma

**DOI:** 10.1172/jci.insight.167712

**Published:** 2023-08-22

**Authors:** Shin Kobayashi, Serina Tokita, Keigo Moniwa, Katsuyuki Kitahara, Hiromichi Iuchi, Kazuhiko Matsuo, Hidehiro Kakizaki, Takayuki Kanaseki, Toshihiko Torigoe

**Affiliations:** 1 Department of Pathology, Sapporo Medical University, Sapporo, Japan.; 2 Department of Renal and Urologic Surgery, Asahikawa Medical University, Asahikawa, Japan.; 3 Joint Research Center for Immunoproteogenomics and; 4 Department of Respiratory Medicine and Allergology, Sapporo Medical University, Sapporo, Japan.; 5 JR Sapporo Hospital, Sapporo, Japan.; 6 Hokushinkai Megumino Hospital, Eniwa, Japan.; 7 Sapporo Clinical Laboratory, Sapporo, Japan.

**Keywords:** Immunology, Oncology, Antigen presentation, Cancer immunotherapy, T cells

## Abstract

CD8^+^ T cells can recognize tumor antigens displayed by HLA class I molecules and eliminate tumor cells. Despite their low tumor mutation burden, immune checkpoint blockade (ICB) is often beneficial in patients with renal cell carcinoma (RCC). Here, using a proteogenomic approach, we directly and comprehensively explored the HLA class I–presenting peptidome of RCC tissues and demonstrated that the immunopeptidomes contain a small subset of peptides derived from human endogenous retroviruses (hERV). A comparison between tumor and normal kidney tissues revealed tumor-associated hERV antigens, one of which was immunogenic and recognized by host tumor-infiltrating lymphocytes (TIL). Stimulation with the hERV antigen induced reactive CD8^+^ T cells in healthy donor–derived (HD-derived) peripheral blood mononuclear cells (PBMC). These results highlight the presence of antitumor CD8^+^ T cell surveillance against hERV3895 antigens, suggesting their clinical applications in patients with RCC.

## Introduction

CD8^+^ T cells can discriminate tumor cells from normal cells and eliminate them. CD8^+^ T cells often recognize HLA-presented neoantigens, which arise from somatic gene mutations. Neoantigens are truly specific to tumors, and T cells reactive to neoantigens are not excluded by negative selection in the thymus. As a result, neoantigens can induce host antitumor responses in various tumors, serving as a target of activated T cells after immune checkpoint blockade (ICB) (1–3). Accordingly, tumor mutation burden (TMB) is positively correlated with patient survival after ICB (4). Likewise, histological types of tumors with high TMB, such as melanoma and non–small cell lung cancer, are susceptible to ICB (5). However, Merkel cell carcinoma and renal cell carcinoma (RCC) are exceptions to this trend (6). ICB responses to these tumors were better than those predicted based on TMB. Since the development of Merkel cell carcinoma is associated with a viral infection, ICB responses may be attributed to T cell recognition of Merkel cell polyoma viruses (7). In contrast, the mechanisms by which T cells recognize RCC remain elusive.

In patients with RCC, the immune surveillance against human endogenous retroviruses (hERV) serves as a possible explanation. hERVs are germline-encoded elements of retroviruses that account for approximately 8% of the human genome (8). Although hERVs are inactive or dysfunctional under physiological conditions, some retain protein coding potential (9). Notably, reactivated hERVs likely elicit cytotoxic T cell responses in RCC (10). This notion is supported by the positive correlation between hERV expression and clinical response to ICB in patients with RCC (11). The positive correlation may be attributable to the activation of both innate and adaptive 53 immunity, with evidence of hERV-derived antigens and T cell recognition (12). Such findings strongly suggest the role of hERVs in T cell–mediated antitumor responses in RCC. Although T cell recognition of an hERV-derived antigen was reported in a patient with melanoma in 2002 (13), the technical difficulty associated with defining hERV-derived antigens has prevented a comprehensive analysis of this unique class of peptides. Recent advances in proteogenomics enabled the comprehensive sequencing of peptides bound to HLA molecules in patient materials, beyond the sequences registered in canonical protein databases (14, 15). In this study, using proteogenomic HLA ligandome analysis with mass spectrometry (MS) and next-generation sequencing, we explored the immunopeptidome of RCC tissues to identify the hERV-derived antigens.

## Results

### Tumor microenvironment and immune profiling of RCC.

**Figure 1:**
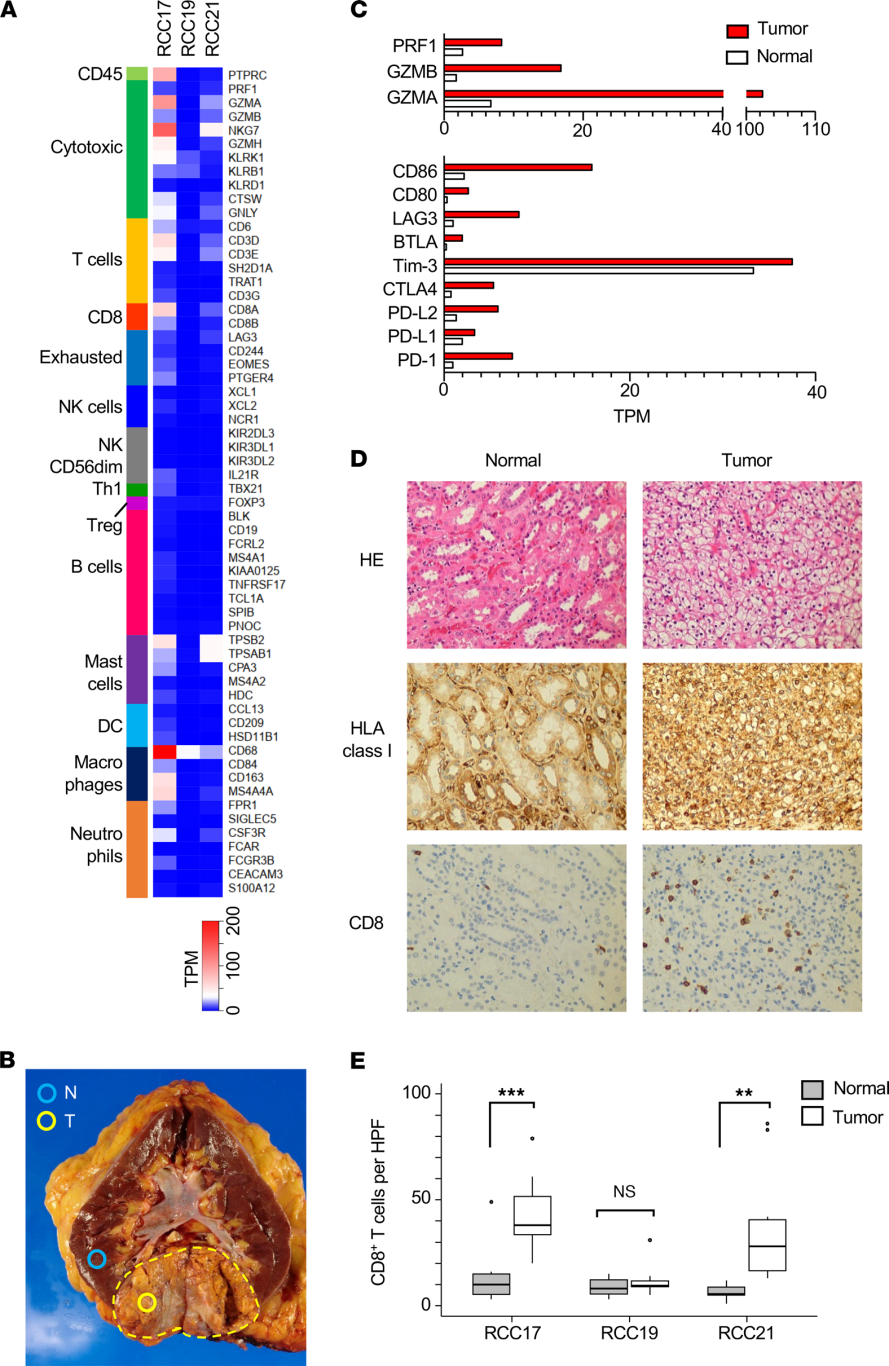
Immune profiling of the RCC tissues. (**A**) Expression of immune-related genes in RCC tissues (RCC17, RCC19, and RCC21). (**B**) Section of the RCC17 kidney showing tumor (T) and normal (N) lesions sampled and used in this study. The dashed line indicates the capsule surrounding the tumor mass. (**C**) Comparison of the expression levels of genes related to cytotoxic T cells (top) and costimulatory or immune checkpoint molecules (bottom) between normal and tumor tissues of RCC17. (**D**) IHC of RCC17 tumor and normal tissues (total original magnification, ×200). (**E**) Numbers of CD8^+^ cells per HPF in RCC17, RCC19, and RCC21 tissues. Box-and-whisker plots represent the median (solid bars), interquartile range (boxes), and 1.5× interquartile range (vertical lines). *P* values were calculated using 2-tailed *t* test. ***P < 0.001, **P < 0.01.

We performed RNA-Seq using the tumor tissues collected from 3 patients with clear cell RCC who underwent surgery for kidney removal. The sets of genes related to immune cells were categorized, and their expression levels were compared across the samples (Figure 1A) (16). This analysis suggested that T cells and macrophages were recruited in the tumor microenvironment (TME) of RCC17. These markers revealed the activation of CD8^+^ cytotoxic T cells. Gene expression was further compared between the tumor and normal tissue lesions of RCC17 (Figure 1B). The expression level of gene sets related to cytotoxic T cell activation (perforin, granzyme A, and granzyme B), costimulation (CD80 and CD86), and immune checkpoints (LAG3, CTLA4, PD-L1, and PD-1) was found to be increased in the tumor tissue (Figure 1C). In addition, IHC revealed that the numbers of CD8^+^ cells observed in tumor lesions were significantly higher than those in normal tissues in RCC17 and RCC21 (Figure 1, D and E). These data indicate the inflamed TME and the induction of spontaneous host T cell responses against tumor cells. The discordance of the inflammatory signature between Figure 1, A and E, in RCC21 may be due to the heterogeneity of the inflammation status within a tumor, since the samples used for RNA-Seq and IHC were obtained from different regions of the same tumor.

### Landscape of peptides presented by HLA class I of RCC tissues.

**Figure 2:**
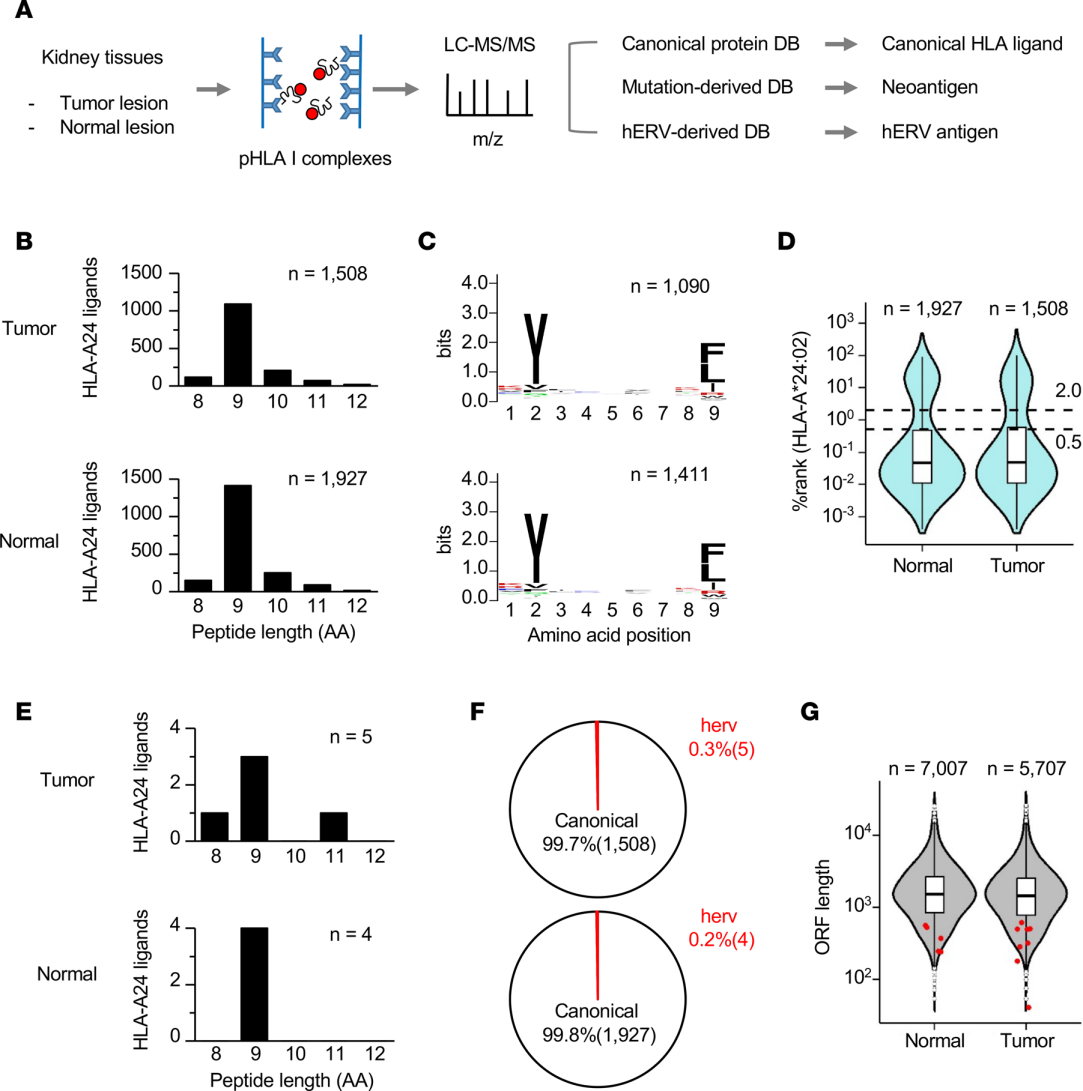
Landscape of the immunopeptidome presented by HLA-A24 of RCC. (**A**) Workflow of the proteogenomic analysis exploring the immunopeptidomes of normal and tumor tissues. pHLA-A24 complexes were captured from RCC17 tumor and normal tissues using a specific antibody, and the eluted peptides were subsequently analyzed by MS. The use of personalized custom databases enabled MS sequencing to detect peptides derived from hERVs. (**B**) Length distribution of canonical peptides identified in normal and tumor tissues. Each bar indicates the number of identified peptides. (**C**) Logo sequence showing the conserved amino acids at each position across all 9 mer peptides. (**D**) Violin plot showing the percentile rank scores (NetMHCpan4.1) of the identified peptides. The dashed lines indicate the thresholds for strong (0.5) and weak (2.0) HLA-A24 binders defined by NetMHCpan4.1. (**E**) Length distribution of hERV-derived peptides identified in normal and tumor tissues. Each bar indicates the number of identified peptides. (**F**) Pie charts showing the frequency of hERV-derived peptides among the identified peptides. (**G**) Violin plot showing the nucleotide lengths of source ORFs encoding the identified peptides. The red dots indicate the distribution of ORFs encoding hERV-derived peptides. Box-and-whisker plots represent the median (solid bars), interquartile range (boxes), and 1.5× interquartile range (vertical lines). The dots denote observations outside the range of adjacent values.

A proteogenomic approach using MS was employed to explore the identity of antigens recognized by CD8^+^ tumor-infiltrating lymphocytes (TILs) (Figure 2A) (14, 15). This approach enables direct and comprehensive analysis of the HLA-presented immunopeptidome, including gene mutation–derived neoantigens, in tissue samples of epithelial solid tumors and nonepithelial tumors or lymph nodes (17, 18). Here, we focused on RCC17 tumor, which was accompanied by the inflamed TME. The peptide–HLA-A24 complexes (pHLA-A24) were immunoprecipitated using a specific antibody, and the eluted peptides were analyzed using MS. After completing the MS database search, only sequences with FDR of 0.01, and the lengths of 8–12 amino acids were selected to ensure rigor. The analysis of RCC17 tumor and normal tissues identified 2,294 nonredundant canonical peptides in total (Supplemental Tables 1 and 2; supplemental material available online with this article; https://doi.org/10.1172/jci.insight.167712DS1). Similar numbers of HLA-A24 ligands were eluted from tumor and normal tissues (Figure 2B). Among the identified peptides, 9 mers were dominant in length, and among the 9 mer peptides, Tyr (Y) was conserved at amino acid position 2, and Phe (F), Leu (L), and Ile (I) were conserved at amino acid position 9 (Figure 2C). These profiles corresponded to those of consensus HLA-A24–bound peptides. Furthermore, the potential for binding to HLA-A24 was estimated using an in silico prediction algorithm. Approximately 73% of the whole identified peptides were predicted as strong HLA-A24 binders (percentile rank scores calculated using NetMHCpan4.1 were below 0.5), ensuring the efficient isolation and purification of naturally presented HLA ligands (Figure 2D) (19).

Here, we leveraged a combination of 3 types of reference databases to conduct the proteogenomic MS analysis to detect neoantigens and a potentially novel class of hERV-derived antigens. First, we searched the immunopeptidome of RCC17 to identify neoantigens. Whole exome sequencing (WES) followed by mutation calling detected 322 and 14 missense and frameshift mutations, respectively, and all the substituted amino acid sequences were integrated into the customized MS reference database for the neoantigen search. However, in contrast to a previous study that used colorectal cancer tissue with mismatch-repair deficiency (18), no neoantigens were detected. This result may be attributed to insufficient sensitivity of the MS analysis or lack of neoantigen presentation in HLA-A24 of RCC17. Next, we proceeded to focus on cryptic peptides that were potentially derived from hERVs. The RNA-Seq data were analyzed using hervQuant to select the hERVs expressed in RCC17 from the reported 3,173 hERV genes (12, 20). A custom reference database containing hypothetical protein sequences originating from hERV-derived potential open-reading frames (ORFs) was prepared. The addition of this database enabled the identification of 8 additional peptides from tumor and normal samples (Table 1). The most dominant length of these peptides was the 9 mer, similar to canonical peptides (Figure 2E). These peptides derived from hERV-ORFs accounted for a minor proportion (0.3%) of the entire peptidome (Figure 2F). These hERV-derived peptides were unique because their peptide sequences were not registered in public protein databases, such as UniProt and RefSeq, and the lengths of their source ORFs were shorter than most ORFs encoding canonical HLA ligands (Figure 2G).

We also explored the immunopeptidome of RCC21, since CD8^+^ T cell infiltration was observed both in RCC17 and RCC21 tumor tissues (Figure 1E). Although patient RCC21 was positive for HLA-A*24:02, we used a pan–HLA class I antibody for immunoprecipitation to expand the analysis. As a result, our proteogenomic pipeline identified 5 hERV peptides in the RCC21 tumor tissue (Table 2). Intriguingly, 2 of the 5 sequences were identical to those detected in RCC17 tumor. This result indicates that some hERV peptides were shared between different individuals with a same HLA type. In contrast, mutation-derived neoantigens were not detected in RCC21 tumor.

### Exploration of hERV peptides associated with tumor.

**Figure 3:**
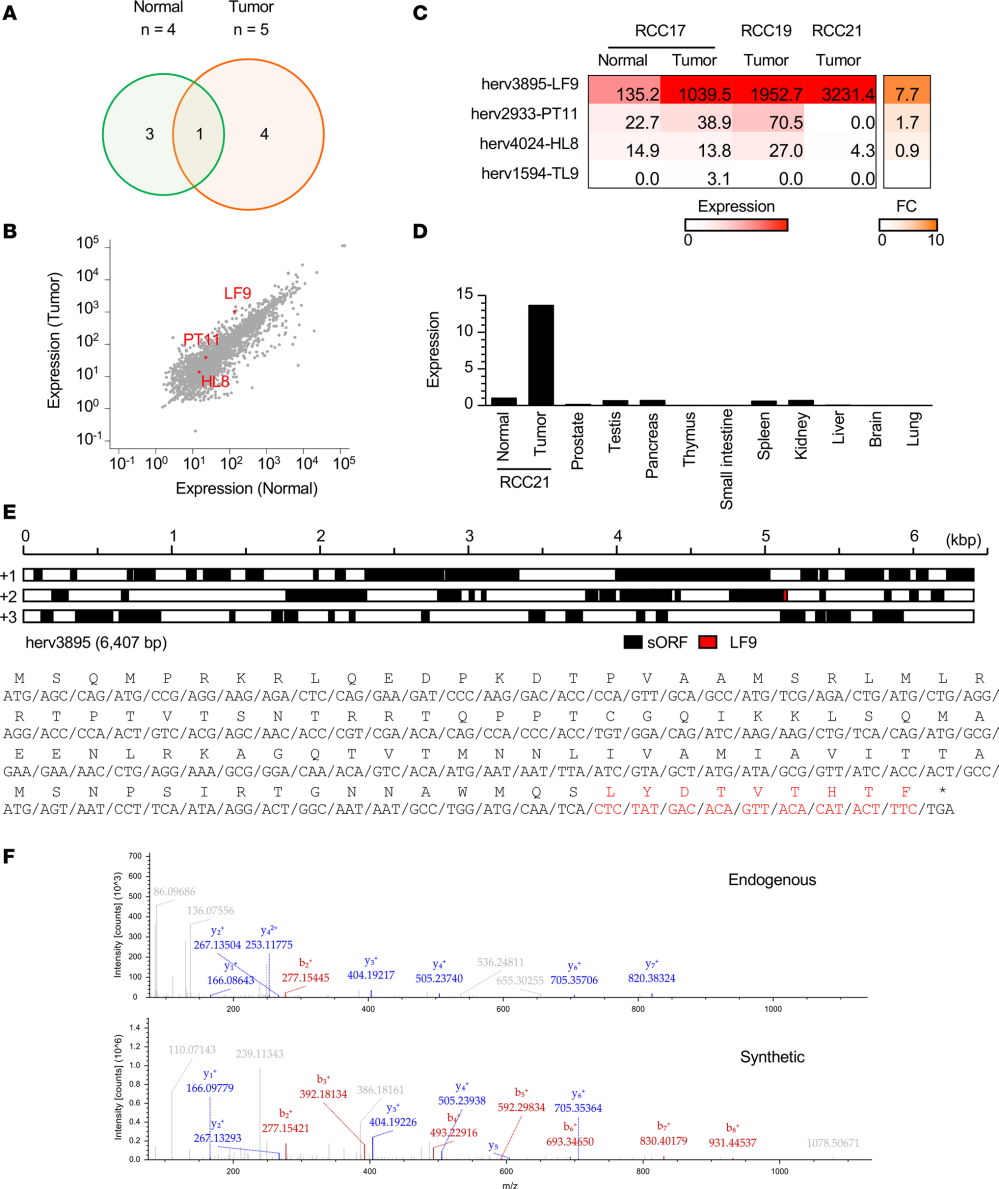
Search of the immunopeptidome for tumor-associated hERV antigens. (**A**) Venn diagram showing the number of hERV-derived peptides exclusive to RCC17 tumor and normal tissues. (**B**) Differential gene expression of hERVs in tumor and normal tissues. hERVs encoding peptides exclusive to the tumor are shown in red. (**C**) Gene expression of the 4 hERV transcripts across 3 RCC tissues. Each fold change (FC) indicates the expression ratio (tumor/normal) in RCC17. (**D**) hERV3895 expression across normal tissues measured by qPCR (*y* axis, FC relative to RCC21 normal tissue). (**E**) Schematic representation of the hERV3895 transcript. Each black box indicates a potential ORF encompassed with ATG and a stop codon. The red box indicates the positions of sequences encoding LF9 (top). Amino acid sequences translated from the ORF encoding LF9 are also shown (bottom). LF9 is shown in red. (**F**) MS/MS spectra and corresponding b- and y-fragment ions of endogenous and synthetic LF9 peptides.

Among the 8 hERV-derived HLA ligands identified in RCC17, 4 and 3 peptides were exclusively detected in tumor and normal tissues, respectively, and 1 peptide was shared by the samples (Figure 3A and Table 1). We focused on the 4 hERV peptides found exclusively in the tumor tissue and assessed their potential as tumor-associated antigens. Differential gene expression analysis between the tumor and normal tissues revealed that hERV3895, 1 of the 4 hERVs encoding the peptides, increased by 7.7-fold in the tumor tissue (Figure 3B). The hERV3895 expression was also high in RCC19 and RCC21 tumor tissues (Figure 3C). Quantitative PCR (qPCR) showed the minimal expression of the transcript across a panel of normal tissues (Figure 3D). The expression levels of the source genes encoding the other 3 hERV peptides were low in the tumor tissue or comparable with those in the normal tissue. In addition, the peptide (LYDTVTHTF [LF9]) encoded by hERV3895 was defined as a strong binder of HLA-A24 based on NetMHCpan4.1, as its percentile rank score was below 0.5 (Table 1). Notably, LF9 presentation was demonstrated in both RCC17 and RCC21 tumor tissues (Tables 1 and 2). Therefore, LF9 was considered as a candidate for tumor-associated hERV antigen, which is shared between patients. LF9 was encoded at the 3′-end of a cryptic ORF, which was not the first ORF or long enough to encode consensus protein sequences (Figure 3E). The MS/MS signal of LF9 detected in RCC17 tumor tissue was validated using a synthetic peptide (Figure 3F).

### Identification of a tumor-associated immunogenic hERV antigen.

**Figure 4:**
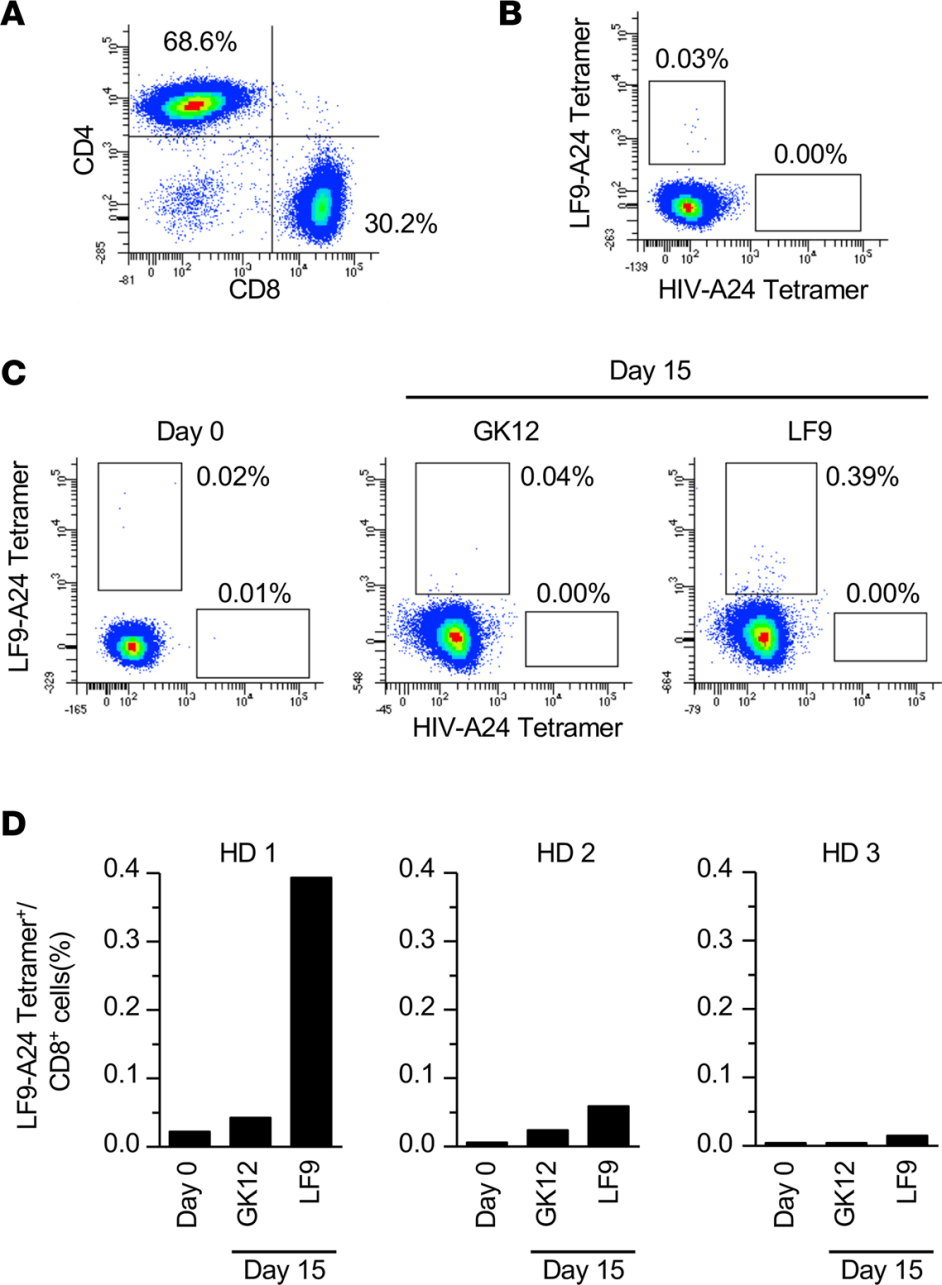
T cell surveillance against the hERV-derived antigen, LF9. (**A**) Flow cytometry of TILs obtained from RCC17 tumor tissue. The TILs were analyzed after in vitro expansion. The data are representative of 3 independent experiments. (**B**) Frequency of CD8^+^ T cells recognizing the LF9–HLA-A24 complex in RCC17 TILs. Staining with the HIV–HLA-A24 tetramer served as a negative control. The data are representative of 3 independent experiments. (**C**) Frequency of CD8^+^ T cells recognizing the LF9–HLA-A24 complex. PBMCs from healthy donors (HD1) were stimulated with the LF9 or an irrelevant peptide (GK12) for 14 days in vitro, and the frequency was measured by flowcytometry. (**D**) Summary of the frequency in different individuals (HD1, HD2, and HD3) after 14-day stimulation with LF9 or GK12.

To assess the immunogenicity of the LF9, RCC17 tumor tissue was minced, and the patient’s TILs were expanded for 4 weeks in vitro. The expanded TILs comprised ~30.2% CD8^+^ and 68.6% CD4^+^ T cells (Figure 4A). Although in vitro expansion often introduces bias in the T cell receptor (TCR) repertoire according to variations in T cell growth (21), the analysis using the LF9–HLA-A24 tetramer revealed a fraction of CD8^+^ T cells specifically recognizing LF9 (Figure 4B). The infiltration of LF9-specific CD8^+^ T cells into the TME strongly suggested the immunogenicity of LF9 eliciting spontaneous host immune responses in vivo, supporting its role as a tumor-associated antigen in the clinical setting. We also evaluated T cell responses to LF9 in healthy donors (HDs). PBMCs from 3 HDs were independently stimulated for 2 weeks with LF9 or an irrelevant peptide (GYISPYFINTSK [GK12]), and the frequency of reactive T cells was compared between day 0 and 15. In contrast to the irrelevant peptide, stimulation with LF9 increased the frequency of CD8^+^ T cells positive for the LF9–HLA-A24 tetramer (Figure 4, C and D). LF9 stimulation resulted in an increase in frequency from 0.02% to 0.39% in 1 of the 3 HDs, whereas GK12 stimulation had no effect on frequency (0.02%–0.04%). These results suggest that LF9 is immunogenic and that there is T cell immune surveillance against the hERV antigen.

## Discussion

In this study, we analyzed the transcriptome of surgically removed RCC tissues and assessed CD8^+^ T cell infiltration into tumors. Furthermore, we explored the immunopeptidomes using a proteogenomic approach with MS in search of responsible tumor antigens. Comprehensive sequencing of the immunopeptidome failed to detect neoantigens arising from somatic gene mutations. However, a new class of antigens derived from hERVs was successfully identified. Notably, a fraction of CD8^+^ TILs recognized one of the hERV antigens, suggesting its immunogenicity in eliciting T cell responses in vivo. Recognition by TILs also implies tumor specificity of the reactive T cells recruited into the TME. These results are in agreement with those of previous studies, demonstrating HLA presentation of hERV-derived unconventional translation products in RCC and supporting T cell–mediated immune surveillance against hERV antigens (10–12, 22, 23). Our proteogenomic pipeline provides an approach for searching HLA peptidomes for hERV antigens.

The immunogenicity of LF9 is likely attributed to its HLA presentation biased toward tumor cells (Table 1). The overexpression of the hERV3895 gene in a tumor is one of the possible explanations of the tumor-specific HLA presentation. However, it remains unclear whether LF9 was an immunodominant antigen responsible for CD8^+^ T cell infiltration in RCC17 tumor. The limited number of LF9-reactive TILs prevented sequencing of their TCRs and estimating their frequency in the TME. In addition, since HLA-A24 immunopeptidomes were focused upon and were explored in RCC17, the possible neoantigen presentation by HLA class I alleles, apart from HLA-A24, cannot be denied.

Meanwhile, the comprehensive analysis of the immunopeptidomes in tumor and normal tissues revealed a caveat. Our result suggests that (a) hERV-derived peptides accounted for only a small proportion (<1%) of the identified HLA class I ligandome in RCC and (b) HLA presentation was not limited to tumor but was equally observed in normal kidney tissue. Therefore, we consider that not all hERV-derived peptides are always immunogenic. Some may be tolerated by host T cells, and adoptive T cell therapy targeting such hERV peptides presented by normal tissues may cause side effects. Thus, careful consideration must be exercised in the search for tumor-specific hERV antigens when leveraging hERV antigens as a target for T cell–based immunotherapy, despite the frequent inactivation of hERVs by epigenetic mechanisms in normal cells (24).

Nevertheless, the presence of antitumor CD8^+^ T cell surveillance against hERV antigens suggest their clinical applications. Unlike most neoantigens, HLA presentation of hERV antigens may not be specific to individuals. Therefore, hERV antigens can serve as tumor antigens shared among HLA-matched patients. Recently, such shared hERV antigens were identified in patients with breast cancer (25). In our study, the expression levels of hERV3895, encoding LF9, were not only high in RCC17 but also in RCC19 and RCC21 tumors (Figure 3C). Moreover, stimulation with LF9 peptide successfully induced reactive CD8^+^ T cells in PBMC from an HLA-A*24:02–matched HD (Figure 4, C and D), indicating that host T cells do not tolerate LF9 in both patients and HDs. Most importantly, LF9 presentation was observed in tumor tissues of different patients (Tables 1 and 2). Hence, LF9 is a tumor antigen shared between patients and potentially serves as an off-the-shelf target for antigen-aware immunotherapy, such as vaccination or gene-engineered T cell therapy.

In summary, our findings highlight the immunogenicity of an hERV antigen, suggesting its role in antitumor T cell surveillance in patients with RCC. Recent studies using proteogenomics revealed the diverse gene source of immunopeptidomes; noncanonical ORFs that do not encode proteins can yield peptides presented by HLA and elicit antitumor T cell responses (26–28). Here, a proteogenomic approach provides direct evidence of HLA class I presentation of hERV-derived peptides, which may be further leveraged as targets of immunotherapy.

## Methods

### Patient material.

Patient material was sampled after surgery at JR Sapporo Hospital (Sapporo, Japan) or Hokushinkai Megumino Hospital (Sapporo, Japan) and immediately frozen at Sapporo Medical University (Sapporo, Japan) until use. All tumors were histologically diagnosed as clear cell RCC. The HLA types were determined using PCR (29) or Polysolver (30). PBMCs were obtained from HLA-A*24:02^+^ HDs.

### IHC.

Formalin-fixed, paraffin-embedded tissues were mounted and stained with H&E, anti–pan HLA class I (EMR8-5, Hokudo), or anti-CD8 (C8/114B, DAKO) on Leica BOND-MAX. Tumor cells were histologically discriminated from normal cells, and the numbers of infiltrating CD8^+^ cells were counted by a pathologist.

### WES.

DNA was extracted from the tumor and normal kidney tissues using the Allprep DNA/RNA/Protein Kit (Qiagen). Exome capture libraries were prepared using SureSelect Human All Exon V6 (Agilent). Sequencing was performed using NovaSeq 6000 (Illumina) with 150 bp paired-end reads with a target depth of 150 coverage per sample. Mutation calling was performed using tumor and normal tissue samples. Library preparation and mutation calling were performed by Macrogen, as previously described (18).

### RNA-Seq.

Total RNA was isolated from the tumor or normal kidney tissues using Allprep DNA/RNA/Protein Kit (Qiagen) or TRIzol Reagent (Invitrogen) with a validated quality of RNA integrity number (RIN) > 7. As previously described, poly A–selected libraries were prepared and sequenced by Macrogen, with 200M of 100 bp paired-end reads per sample (18). The abundance of genes or transcripts was calculated as transcripts per million (TPM). Marker genes for immune cells were selected and grouped as previously described (16). The gene expression of hERVs was calculated using hervQuant (12).

### Isolation of HLA class I ligands and MS analysis.

The samples were prepared, and MS analysis was performed as previously described (18). Frozen tissues were ground under cryogenic conditions and lysed with lysate buffer containing 0.25% sodium deoxycholate (Wako), 0.2 mM iodoacetamide (Wako), 1 mM EDTA (Dojindo), protease inhibitor cocktail (MilliporeSigma), 1 mM PMSF (MilliporeSigma), and 1% octyl-β-D glucopyranoside (Dojindo) in DPBS (Thermo Fisher Scientific). The pHLA-A24 complexes were captured using affinity chromatography of HLA-A24 mAb coupled to CNBr-activated Sepharose 4B (GE Healthcare) overnight. The HLA ligands were eluted with 0.2% TFA and desalted using Sep-Pak tC18 (Waters) with 28% ACN in 0.1% TFA and ZipTip U-C18 (MilliporeSigma) with 50% ACN in 1% FA. The samples were dried by vacuum centrifugation (SPD2010, Thermo Fisher Scientific) at 25°C for 15 minutes and resuspended in 5% ACN in 0.1% TFA for LC-MS/MS analysis. To prepare the HLA-A24 mAb, C7709A2 hybridoma (provided by P.G. Coulie, Institut de Duve, Brussels, Belgium) was cultured in Hybridoma serum-free medium (SFM) (Thermo Fisher Scientific) supplemented with 1% penicillin-streptomycin in CELLine Bioreactor Flasks (CL1000, Corning). Condensed mAbs were collected and purified using a HiTrap Protein G HP (GE Healthcare). For RCC21, W6/32 (ATCC) was used for immunoprecipitation.

Samples containing HLA-A24 ligands isolated from tissues were loaded into a nano-flow LC (Easy-nLC 1000 system, Thermo Fisher Scientific) online coupled to an Orbitrap mass spectrometer equipped with a nanospray ion source (Q Exactive Plus, Thermo Fisher Scientific). Nanoflow LC separation was performed with a linear gradient ranging from 3% to 30% buffer B (100% ACN and 0.1% FA), with a flow rate of 300 nL/min for 80 minutes and a 75 μm × 20 cm capillary column with a particle size of 3 μm (NTCC-360, Nikkyo Technos). For MS, the survey scan spectra were acquired at a resolution of 70,000 at 200 *m*/*z*, with an AGC target value of 3 × 10^6^ ions and a maximum IT of 100 ms, ranging from 350 to 2,000 *m*/*z* with charge states between 1^+^ and 4^+^. A data-dependent top 10 method was employed. The MS/MS resolution was 17,500 at 200 *m*/*z*, with an AGC target value of 1 × 10^5^ ions and a maximum IT of 120 ms.

### Proteogenomic identification of hERV-derived antigens.

A custom database for MS searches was constructed using Python scripts. The database comprised 3 sets of sequences: (a) GENCODE protein-coding transcript translation sequences (release 31), (b) protein sequences altered with the missense or frameshift mutation found in the sample starting from 30 amino acids upstream of a mutated residue and ending with 30 amino acid downstream residues (missense) or stop codons (frameshift), and (c) hERV-derived hypothetical protein sequences, in which potential ORFs that start with ATG and end with stop codons found in hERVs were translated into 3 frames. Only the protein sequences with gene expression (TPM > 0) were included in the database. hERV expression was calculated using hervQuant (12).

MS/MS data were searched against the custom database using Sequest HT and the Percolator algorithm on the Proteome Discoverer 2.3 platform (Thermo Fisher Scientific). The tolerances of the precursor and fragment ions were set at 10 ppm and 0.02 Da, respectively. Methionine oxidation (+15.995 Da) was selected as the dynamic modification. No specific enzymes were selected for the search. Concatenated target-decoy selection was validated based on *q* values, and a FDR of 0.01 was used in the percolator node as a peptide detection threshold. The 8–12 mer peptides were counted as natural HLA-A*24:02 ligands. The hERV-derived peptides were not registered in public protein databases (UniProt and RefSeq). Since MS cannot discriminate L from I, this validation was performed using both the original and alternative hERV-derived peptide sequences, in which Leu and Ile were replaced with Ile and Leu, respectively. In 6 of 8 hERV-derived peptides, their source genes did not overlap. In contrast, the other 2 hERV-derived peptides had multiple source-hERV candidates. Therefore, the most abundant candidates with the highest gene expression levels (hERV4024 for HFNSFHFL and hERV2710 for SQYVFLTLQ) were selected as representatives and shown as their source hERV genes in this study.

### TIL.

Tumor tissues were manually minced and cultured in AIM-V medium (Thermo Fisher Scientific) supplemented with 0.1 mg/mL liberase (Roche) and 1.75 μg/mL DNase (Roche) for 30 minutes. The cells were washed and further cultured in AIM-V with 6,000 U/mL IL-2 (Peprotech) and 2.5 μg/mL amphotericin B (Thermo Fisher Scientific) for 4 weeks. AIM V medium was supplemented with 1% penicillin-streptomycin (Thermo Fisher Scientific), 1% GlutaMAX (Thermo Fisher Scientific), 10 mM HEPES (Thermo Fisher Scientific), 1 mM sodium pyruvate (Thermo Fisher Scientific), 55 μM 2-mercaptoethanol (Thermo Fisher Scientific), and 10% human AB serum (Biowest). The expanded cells were cultured in AIM-V supplemented with 6,000 U/mL IL-2, 30 ng/mL anti-CD3 (OKT3, BioLegend, 317302), and 2.5 μg/mL amphotericin B with irradiated (100 Gy) HD-derived PBMCs for 2 weeks. The expanded cells were cryopreserved as TILs until use.

### Flowcytometry.

PBMCs were isolated from the peripheral blood of 3 HDs using Lymphoprep (Cosmo Bio) according to the manufacturer’s instructions. The cells were stimulated with 1 μM of LF9 peptide or GK12 peptide (negative control) on days 0 and 7, and 50 U/mL rhIL-2 (Peprotech) was added on day 1. TILs or stimulated HD PBMCs were prestained with human FcR blocking reagent (Clear Back, MBL) on day 15 and stained with LF9– or HIV_env584-592_–HLA-A24 tetramer (a tetramer for HIV-1 envelope 584-592 aa and HLA-A*24:02, MBL) conjugated with PE or FITC for 20 minutes at 4°C, followed by anti–CD8-PC5 (SFCI21Thy2D3, Beckman Coulter) for 20 minutes at 4°C. The cells were also stained with anti–CD8-APC (HIT8a, BioLegend), anti–CD4-PE-Cy7 (OKT4, BioLegend), and anti–CD3-PE (UCHT1, BioLegend) for 20 minutes at 4°C. The stained cells were analyzed using FACSCanto II with FACSDiva (BD Biosciences). LF9– and HIV_env584-592_–HLA-A24 tetramers were purchased from MBL. Synthetic peptides (LF9, GK12) with > 80% purity were purchased (MilliporeSigma and Cosmo Bio, respectively).

### qPCR.

RNA extraction was conducted as previously described (28). RNA samples were treated with RNase-Free DNase Set (Qiagen). cDNA was synthesized from 0.25 μg of total RNA isolated from cancer or normal kidney tissues by reverse transcription using SuperScript III (Invitrogen). A panel of cDNAs from human normal tissues was purchased from Clontech. Gene expression was measured using the StepOne Real-Time PCR System (Applied Biosystems) with PowerUp SYBR Green Master Mix (Thermo Fisher Scientific). Primer pairs were as follows: hERV3895, (forward) 5′-CAGGAAGATCCCAAGGACAC-3′ and (reverse) 5′-CATAGAGTGATTGCATCCAGG-3′ (product size 321 bp, designed to encompass the LF9-encoding ORF); G3PDH, (forward) 5′-ACCACAGTCCATGCCATCAC-3′ and (reverse) 5′-TCCACCACCCTGTTGCTGTA-3′ (product size 452 bp). Each sample was analyzed in triplicate, and the threshold cycle values (Ct) of hERV3895 were normalized according to those of G3PDH.

### Statistics.

Statistical analysis was performed using Excel (Microsoft). *P* values were calculated using 2-tailed *t* tests.

### Study approval.

The study was conducted with the approval of the IRB (no. 342-1103) and the Research Ethics Committee of Sapporo Medical University (no. 29-2-69). All the patients and HDs enrolled in this study signed an informed consent form.

### Data availability.

MS raw data and personalized FASTA files generated based on WES and RNA-Seq data were deposited in the ProteomeXchange Consortium via the jPOSTrepo partner repository (https://repository.jpostdb.org) with the data set identifier PXD038140. Supporting Data Values associated with the manuscript are provided in the supplement.

## Author contributions

SK, HI, KK, and HK provided and prepared the patient materials. SK, ST, and K Moniwa performed the experiments. K Matsuo performed IHC. SK, ST, and TK interpreted the data. SK, ST, and TK wrote the manuscript. TK conceived and designed the project. TK and TT jointly supervised the study. All authors reviewed and approved the manuscript content.

## Supplementary Material

Supplemental data

Supplemental table 1

Supplemental table 2

Supporting data values

## Figures and Tables

**Table 2:**
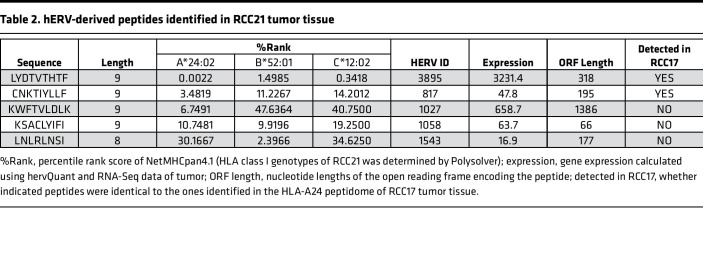
hERV-derived peptides identified in RCC21 tumor tissue

**Table 1:**
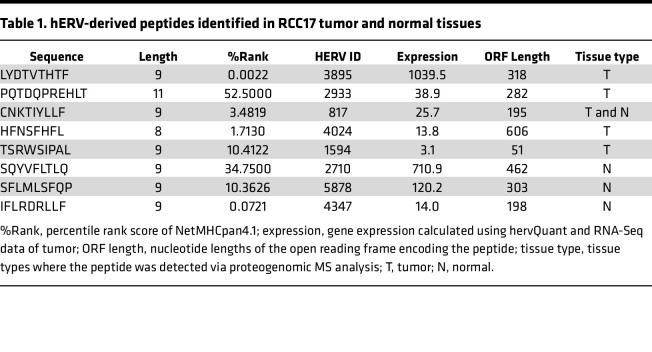
hERV-derived peptides identified in RCC17 tumor and normal tissues

## Version 1 08/22/2023

Electronic publication
