# Supplementary material for: Proteogenomic identification of an immunogenic antigen derived from human endogenous retrovirus in renal cell carcinoma
Source: JCI Insight. 2023 Aug 22;8(16):e167712. doi: 10.1172/jci.insight.167712 (PMC10543709; doi:10.1172/jci.insight.167712)
Supplement: Supplemental data [file jciinsight-8-167712-s127.pdf]

Supplementary table 1: HLA-A24 ligands detected in the RCC17 tumor tissue

Sequence	Length	%rank	Gene	Ensembl gene ID	Ensembl protein ID	Expression	ORF Length
QALLDLHAL	9	8.8193	FTL	ENSG00000087086.15	ENSP00000366525.2	7381.9	525
VMAPRTVLL	9	0.4689	HLA-B	ENSG00000234745.11	ENSP00000399168.2	5712.5	1086
SMRYFYTSV	9	12.8611	HLA-B	ENSG00000234745.11	ENSP00000399168.2	5712.5	1086
YAYDGKDYIAL	11	8.8004	HLA-B	ENSG00000234745.11	ENSP00000399168.2	5712.5	1086
LMISRTPEV	9	5.5142	IGHG1	ENSG00000211896.7	ENSP00000374984.2	5656.8	885
VMAPRTLLL	9	0.3778	HLA-A	ENSG00000206503.13	ENSP00000366005.5	3642.7	1095
HYIRKYNRF	9	0.0164	RPS11	ENSG00000142534.7	ENSP00000270625.1	2985.7	474
KFIDTTSKF	9	0.0212	RPL3	ENSG00000100316.16	ENSP00000346001.3	2655.3	1209
DVIGVTKGK	9	25.0000	RPL3	ENSG00000100316.16	ENSP00000346001.3	2655.3	1209
DVIAQGIGKL	10	28.1250	RPLP2	ENSG00000177600.9	ENSP00000322419.4	2005.9	345
NYIDKVRFL	9	0.0729	VIM	ENSG00000026025.16	ENSP00000224237.5	2172.4	1398
RISLPLPNF	9	0.8058	VIM	ENSG00000026025.16	ENSP00000224237.5	2172.4	1398
LYEEEMREL	9	0.5724	VIM	ENSG00000026025.16	ENSP00000224237.5	2172.4	1398
VYATRSSAVRL	11	0.4953	VIM	ENSG00000026025.16	ENSP00000224237.5	2172.4	1398
SYVTTSTRTYSL	12	1.3997	VIM	ENSG00000026025.16	ENSP00000224237.5	2172.4	1398
LYLKVKGNVF	10	0.1685	RPL19	ENSG00000108298.11	ENSP00000464538.1	2017.5	582
AYLPVNESF	9	0.0029	EEF2	ENSG00000167658.16	ENSP00000307940.5	1542.0	2574
RYFDPANGKF	10	0.0144	EEF2	ENSG00000167658.16	ENSP00000307940.5	1542.0	2574
KYQAVTATL	9	0.0175	RPL13A	ENSG00000142541.17	ENSP00000375730.4	2300.2	609
SQLRVAKV	8	21.5455	RPL35	ENSG00000136942.15	ENSP00000259469.4	1526.2	369
YYTRLGNDF	9	0.0970	RPS17	ENSG00000182774.13	ENSP00000498019.1	1456.7	405
YWSHPRKF	8	0.6397	RPS29	ENSG00000213741.10	ENSP00000245458.6	1143.5	168
LYWSHPRKF	9	0.0225	RPS29	ENSG00000213741.10	ENSP00000245458.6	1143.5	168

GQFKTYAI	8	16.6727	RPS21	ENSG00000171858.18	ENSP00000345957.3	1415.8	249
IYSVFRNAASF	11	0.2225	PLIN2	ENSG00000147872.10	ENSP00000276914.2	1012.4	1311
RLPEEWSQW	9	0.2153	HSPB1	ENSG00000106211.9	ENSP00000248553.6	933.4	615
EVTRVKAVR	9	37.6667	TXNIP	ENSG00000265972.5	ENSP00000462521.1	932.8	1173
HVIGLQMGSNR	11	44.0000	TAGLN	ENSG00000149591.16	ENSP00000376678.4	871.2	603
YVHMVTHF	8	0.8009	TMBIM6	ENSG00000139644.13	ENSP00000447030.1	809.6	600
EHGDQDYIW	9	2.7597	TMBIM6	ENSG00000139644.13	ENSP00000447030.1	809.6	600
IYKPGQTVKF	10	0.0098	A2M	ENSG00000175899.14	ENSP00000323929.7	711.7	4422
EVIPYTPAM	9	5.3868	HMOX1	ENSG00000100292.17	ENSP00000216117.8	668.3	864
EVIPYTPAMQR	11	30.8333	HMOX1	ENSG00000100292.17	ENSP00000216117.8	668.3	864
QQSKILKV	8	18.0526	HSP90AB1	ENSG00000096384.20	ENSP00000360709.5	556.4	2172
RFVNVVPTF	9	0.0122	FAU	ENSG00000149806.11	ENSP00000435370.1	659.0	399
RYESLKGVDPKF	12	0.4209	RPL29	ENSG00000162244.11	ENSP00000294189.4	561.2	477
YFKRYQVKF	9	0.0955	RPL5	ENSG00000122406.14	ENSP00000359345.2	774.6	891
ETIPLTAEK	9	20.4074	CCND1	ENSG00000110092.3	ENSP00000227507.2	489.5	885
RYLTVAAVF	9	0.0167	TUBB	ENSG00000196230.13	ENSP00000339001.7	457.8	1332
EYPDRIMNTF	10	0.0274	TUBB	ENSG00000196230.13	ENSP00000339001.7	457.8	1332
SQVEILQRV	9	4.4332	ID3	ENSG00000117318.9	ENSP00000363689.5	532.8	357
RYSSMAASF	9	0.0077	PDZK1IP1	ENSG00000162366.8	ENSP00000294338.2	478.2	342
DQYKFLAV	8	16.1273	NDUFA4L2	ENSG00000185633.10	ENSP00000450664.1	646.1	261
YWEVQPATF	9	0.1017	BGN	ENSG00000182492.16	ENSP00000327336.4	888.9	1104
ELIQDITQR	9	20.9630	MSN	ENSG00000147065.17	ENSP00000353408.5	379.6	1731
NVIGLQMGTNR	11	48.0000	TAGLN2	ENSG00000158710.14	ENSP00000357077.4	383.6	597
SYMEVPTYL	9	0.0073	LAPTM5	ENSG00000162511.8	ENSP00000294507.3	373.6	786
SYIELPAYL	9	0.0221	LAPTM5	ENSG00000162511.8	ENSP00000294507.3	373.6	786
QQLTAMKV	8	32.6000	MYH9	ENSG00000100345.21	ENSP00000216181.5	378.3	5880
KYLYVDKNF	9	0.0370	MYH9	ENSG00000100345.21	ENSP00000216181.5	378.3	5880

TYISSVYHV	9	0.0593	SLC17A3	ENSG00000124564.17	ENSP00000355307.6	809.5	1260
DYVEGLRVF	9	0.1353	MYL6	ENSG00000092841.18	ENSP00000446955.1	991.5	453
AYVLNIVRF	9	0.0134	ATP1B1	ENSG00000143153.12	ENSP00000356789.3	439.0	909
KYMTAVVKLF	10	0.0213	CYBA	ENSG00000051523.11	ENSP00000261623.3	380.3	585
SYTSHTNEI	9	0.1620	C7	ENSG00000112936.18	ENSP00000322061.9	307.9	2529
IYRDLISHDEMF	12	0.3038	TPT1	ENSG00000133112.16	ENSP00000431872.1	1437.9	516
KYTPPPHHI	9	0.0036	NDUFS5	ENSG00000168653.11	ENSP00000362058.3	430.2	318
AYVVKVFSL	9	0.0716	C3	ENSG00000125730.16	ENSP00000245907.4	309.6	4989
AYKYIQELW	9	0.0402	RPL15	ENSG00000174748.21	ENSP00000483260.1	568.4	612
HSYSPRAI	8	23.0000	PSMB4	ENSG00000159377.11	ENSP00000290541.6	260.7	792
IYSQTYFRF	9	0.0018	TNFSF10	ENSG00000121858.11	ENSP00000241261.2	281.8	843
TYFRFQEEI	9	0.0914	TNFSF10	ENSG00000121858.11	ENSP00000241261.2	281.8	843
TYVYFTNEL	9	0.1507	TNFSF10	ENSG00000121858.11	ENSP00000241261.2	281.8	843
RFKTQPVTF	9	0.0563	C11orf96	ENSG00000187479.7	ENSP00000479976.1	240.8	366
IWDTAGQERF	10	0.3388	RAB1B	ENSG00000174903.16	ENSP00000310226.6	247.0	603
KYPDAVATW	9	0.0027	SPP1	ENSG00000118785.14	ENSP00000237623.7	725.9	900
VYGPMKNYL	9	0.0244	ANPEP	ENSG00000166825.14	ENSP00000300060.6	316.1	2901
AQTTLRNV	8	40.0000	STOM	ENSG00000148175.12	ENSP00000286713.2	262.6	864
RYLQTLTTI	9	0.0160	STOM	ENSG00000148175.12	ENSP00000286713.2	262.6	864
VFIKAVHV	8	3.8628	JCHAIN	ENSG00000132465.11	ENSP00000440066.2	241.5	477
VYKVPPFTF	9	0.0019	CAVIN1	ENSG00000177469.13	ENSP00000349541.4	234.4	1170
EVNEAELLRR	10	45.5000	CAVIN1	ENSG00000177469.13	ENSP00000349541.4	234.4	1170
SGPPVSELITK	11	16.1818	HIST1H1C	ENSG00000187837.3	ENSP00000339566.2	225.8	639
ASGPPVSELITK	12	38.3333	HIST1H1C	ENSG00000187837.3	ENSP00000339566.2	225.8	639
ETAEAYLGK	9	28.0000	HSPA5	ENSG00000044574.8	ENSP00000324173.6	224.0	1962
TVFDAKRLIGR	11	27.3000	HSPA5	ENSG00000044574.8	ENSP00000324173.6	224.0	1962
NYARGHYTI	9	0.0422	TUBA1A	ENSG00000167552.14	ENSP00000446637.1	269.4	1248

AYGFVARI	8	1.8018	VWF	ENSG00000110799.13	ENSP00000261405.5	228.0	8439
EVIASYAHL	9	5.6274	VWF	ENSG00000110799.13	ENSP00000261405.5	228.0	8439
RYLSDHSFL	9	0.0771	VWF	ENSG00000110799.13	ENSP00000261405.5	228.0	8439
EVIEKLFAM	9	4.8930	EPAS1	ENSG00000116016.14	ENSP00000263734.3	249.6	2610
LYLKALEGF	9	0.0273	EPAS1	ENSG00000116016.14	ENSP00000263734.3	249.6	2610
AQLLQLKV	8	25.6667	FKBP8	ENSG00000105701.16	ENSP00000476767.1	289.5	1239
NYKKFYEQF	9	0.0105	HSP90AA1	ENSG00000080824.18	ENSP00000216281.7	254.3	2196
TIGEILKK	8	55.0000	HNRNPK	ENSG00000165119.21	ENSP00000317788.4	383.6	1389
ETIGEILKK	9	20.2222	HNRNPK	ENSG00000165119.21	ENSP00000317788.4	383.6	1389
AQYLLQNSV	9	8.6807	HNRNPK	ENSG00000165119.21	ENSP00000317788.4	383.6	1389
EIAYSDVAKR	10	49.0000	CLIC4	ENSG00000169504.15	ENSP00000363500.4	215.4	759
YIEHIFEI	8	1.1212	PEA15	ENSG00000162734.12	ENSP00000353660.4	250.7	390
SYIEHIFEI	9	0.0059	PEA15	ENSG00000162734.12	ENSP00000353660.4	250.7	390
RYGDLVDYL	9	0.1140	PDGFRB	ENSG00000113721.14	ENSP00000261799.4	261.1	3318
VLPKLYVKL	9	0.3164	RPS26	ENSG00000197728.11	ENSP00000496643.1	379.2	345
YLEKYYKF	8	0.2200	NNMT	ENSG00000166741.7	ENSP00000299964.3	259.0	792
DYLEKYYKF	9	0.0085	NNMT	ENSG00000166741.7	ENSP00000299964.3	259.0	792
YYMIGEQKF	9	0.0041	NNMT	ENSG00000166741.7	ENSP00000299964.3	259.0	792
RYQDAIRVF	9	0.0072	EIF3L	ENSG00000100129.18	ENSP00000384634.1	258.9	1398
EIINEDIAKR	10	49.0000	PDIA6	ENSG00000143870.12	ENSP00000272227.3	230.6	1320
TYLDHNNFW	9	0.0261	MAOB	ENSG00000069535.14	ENSP00000367309.4	186.4	1560
HQFDSVKRI	9	4.1305	ENPEP	ENSG00000138792.10	ENSP00000265162.5	195.9	2871
SYMGHFDLL	9	0.0245	SF3B5	ENSG00000169976.6	ENSP00000356541.2	181.5	258
ESITDVLVR	9	38.0000	MCL1	ENSG00000143384.13	ENSP00000358022.2	196.6	1050
EVIEIMTDR	9	31.5000	HNRNPA1	ENSG00000135486.17	ENSP00000341826.6	444.3	1116
SSSYGSGRRF	10	7.5071	HNRNPA1	ENSG00000135486.17	ENSP00000341826.6	444.3	1116
SGPYGGGGQYF	11	0.1730	HNRNPA1	ENSG00000135486.17	ENSP00000341826.6	444.3	1116

SSSSYGSGRRF	11	8.2038	HNRNPA1	ENSG00000135486.17	ENSP00000341826.6	444.3	1116
SSSSSYGSGRRF	12	12.6042	HNRNPA1	ENSG00000135486.17	ENSP00000341826.6	444.3	1116
HQAAIVSKI	9	3.7087	CALD1	ENSG00000122786.20	ENSP00000354513.2	230.8	1614
ETAGLKVGVSSR	12	53.7500	CALD1	ENSG00000122786.20	ENSP00000354513.2	230.8	1614
YLDKTEQW	8	0.7174	C4A	ENSG00000244731.8	ENSP00000396688.2	747.9	5232
RYLDKTEQW	9	0.0047	C4A	ENSG00000244731.8	ENSP00000396688.2	747.9	5232
YYPRVEYGF	9	0.0091	C4A	ENSG00000244731.8	ENSP00000396688.2	747.9	5232
AFRGILQR	8	21.5000	HLA-DQB1	ENSG00000179344.16	ENSP00000364080.4	294.9	807
EVAFRGILQR	10	37.6667	HLA-DQB1	ENSG00000179344.16	ENSP00000364080.4	294.9	807
SLFAGGMLR	9	12.8264	TMED9	ENSG00000184840.11	ENSP00000330945.6	183.8	705
IGAEVYHNL	9	1.4658	ENO1	ENSG00000074800.16	ENSP00000495530.1	1690.0	1302
VYHNLKNVI	9	0.1946	ENO1	ENSG00000074800.16	ENSP00000495530.1	1690.0	1302
RYISPDQLADL	11	0.2320	ENO1	ENSG00000074800.16	ENSP00000495530.1	1690.0	1302
HQLSLRTV	8	19.1290	NPM1	ENSG00000181163.13	ENSP00000377408.2	449.4	777
TYMGHTGAVW	10	0.1733	EIF3I	ENSG00000084623.11	ENSP00000362688.1	178.8	975
KYDEIFYNL	9	0.0149	EHD2	ENSG00000024422.12	ENSP00000263277.2	164.7	1629
IYNGDMEKI	9	0.1535	BNIP3	ENSG00000176171.11	ENSP00000357625.5	204.2	777
LYSSENISNF	10	0.0756	PDLIM1	ENSG00000107438.9	ENSP00000360305.3	171.2	987
YIIGLLQQR	9	13.5225	FBXL5	ENSG00000118564.14	ENSP00000425472.1	181.7	1836
RYLDGWNAI	9	0.0772	CYP27A1	ENSG00000135929.9	ENSP00000258415.4	184.5	1593
EVIDDFMTR	9	10.9466	CYP27A1	ENSG00000135929.9	ENSP00000258415.4	184.5	1593
YYLNDLERI	9	0.0630	GNAI2	ENSG00000114353.17	ENSP00000312999.6	420.9	1065
KYPDRVPVI	9	0.0180	GABARAPL2	ENSG00000034713.8	ENSP00000037243.2	164.1	351
KYGPIRQI	8	0.5562	SF3B6	ENSG00000115128.7	ENSP00000233468.4	157.0	375
VFIVSTGRGNF	11	1.5794	VWA1	ENSG00000179403.12	ENSP00000417185.1	202.9	1335
RYIPTAAAF	9	0.0046	SEC61A1	ENSG00000058262.10	ENSP00000243253.3	156.2	1428
VYLGHVIYL	9	0.0162	GJA4	ENSG00000187513.9	ENSP00000343676.4	176.1	999

RMNPNSPSI	9	1.1774	ERH	ENSG00000100632.11	ENSP00000451080.1	154.5	312
LYYVDEHGTRL	11	0.7654	PSMB8	ENSG00000204264.11	ENSP00000364016.3	245.9	828
ETIKGIQKR	9	21.4545	ITM2B	ENSG00000136156.15	ENSP00000497221.1	262.3	798
ELIAKIPNF	9	0.9133	SET	ENSG00000119335.16	ENSP00000318012.8	174.5	831
RYIANTVEL	9	0.0295	RPN2	ENSG00000118705.17	ENSP00000237530.6	252.3	1893
PYISNIYLI	9	0.0371	PLOD1	ENSG00000083444.17	ENSP00000196061.4	144.4	2181
DYLKRFYLY	9	0.3325	MMP7	ENSG00000137673.9	ENSP00000260227.4	134.4	801
PYSQVWIHF	9	0.0108	CUBN	ENSG00000107611.15	ENSP00000367064.4	141.8	10869
HVVHGRILEM	10	24.2857	CUBN	ENSG00000107611.15	ENSP00000367064.4	141.8	10869
KYLATLETL	9	0.0213	JAK1	ENSG00000162434.12	ENSP00000343204.4	135.9	3462
HYPLNTVTF	9	0.0074	TNS1	ENSG00000079308.19	ENSP00000498399.1	161.9	5481
VYTSGIYNI	9	0.0158	TNS1	ENSG00000079308.19	ENSP00000498399.1	161.9	5481
FQYFVKVV	8	27.9000	ERGIC3	ENSG00000125991.19	ENSP00000399124.1	327.1	1191
KMPKVKMPKF	10	0.3886	AHNAK	ENSG00000124942.14	ENSP00000367263.4	142.4	17670
KMPKMKMPTF	10	0.3399	AHNAK	ENSG00000124942.14	ENSP00000367263.4	142.4	17670
KYAPSGFYI	9	0.0119	WDR1	ENSG00000071127.17	ENSP00000371890.2	183.0	1818
KYLDIPKML	9	0.0220	ACTN4	ENSG00000130402.12	ENSP00000252699.2	490.3	2733
KYLSDNVHL	9	0.0732	CDC37	ENSG00000105401.9	ENSP00000222005.1	204.7	1134
VYVQHPITF	9	0.0016	LITAF	ENSG00000189067.12	ENSP00000340118.5	131.5	483
KYLSVQGQLF	10	0.0077	MTCH1	ENSG00000137409.19	ENSP00000362730.5	204.4	1167
MQIRIHQI	8	6.1989	DNAJA1	ENSG00000086061.16	ENSP00000369127.3	112.5	1191
AQYLINVRL	9	3.6929	PCBP2	ENSG00000197111.15	ENSP00000448762.1	501.6	1086
TYISKTIAL	9	0.0533	EXOC3L2	ENSG00000283632.2	ENSP00000400713.2	108.7	2406
EVMRLVKGM	9	17.5778	ASNA1	ENSG00000198356.11	ENSP00000481254.1	119.5	993
VYSRTFTW	8	0.1570	MYO1C	ENSG00000197879.17	ENSP00000354283.2	137.3	3084
VYSRTFTWL	9	0.0981	MYO1C	ENSG00000197879.17	ENSP00000354283.2	137.3	3084
NYPQSVPRL	9	0.0404	MYO1C	ENSG00000197879.17	ENSP00000354283.2	137.3	3084

FYPYGLQTF	9	0.0102	SUN2	ENSG00000100242.15	ENSP00000385616.1	158.7	2214
VYGPLPQSF	9	0.0008	COX4I1	ENSG00000131143.8	ENSP00000455301.1	323.9	324
HQQVVSRI	8	15.9855	SLC9A3R1	ENSG00000109062.12	ENSP00000262613.5	112.3	1074
YSVYVYKVLK	10	43.0000	HIST1H2BD	ENSG00000158373.8	ENSP00000289316.2	135.1	378
VYPRPIITW	9	0.0036	HHLA2	ENSG00000114455.13	ENSP00000482187.1	120.4	1242
RQLVFRYHV	9	4.2040	STAB1	ENSG00000010327.10	ENSP00000312946.6	172.7	7710
KYKDQPQQTF	10	0.0156	STAB1	ENSG00000010327.10	ENSP00000312946.6	172.7	7710
EITESGRIGF	10	14.0333	ITGB2	ENSG00000160255.18	ENSP00000303242.6	185.7	2379
SYTNVYRMF	9	0.0100	GLYATL1	ENSG00000166840.13	ENSP00000479741.1	194.6	906
PEPAKSAPAPK	11	48.0000	HIST1H2BK	ENSG00000197903.7	ENSP00000349430.1	101.2	378
ETPSWTGSGF	10	4.5089	LAMB2	ENSG00000172037.14	ENSP00000307156.4	283.2	5394
VYGFVREAL	9	0.2693	UBXN6	ENSG00000167671.12	ENSP00000301281.5	124.0	1323
DVYENLYAGR	10	30.0000	MAPKAPK2	ENSG00000162889.10	ENSP00000356070.3	111.4	1200
GYIHVTQTF	9	0.0035	NKG7	ENSG00000105374.10	ENSP00000221978.4	138.6	495
VYLAAVNRL	9	0.0222	PLXND1	ENSG00000004399.12	ENSP00000317128.4	211.5	5775
GLQFPVGR	8	45.5000	H2AFJ	ENSG00000246705.4	ENSP00000438553.1	119.8	387
AGLQFPVGR	9	23.7500	H2AFJ	ENSG00000246705.4	ENSP00000438553.1	119.8	387
GLQFPVGRVHR	11	25.3333	H2AFJ	ENSG00000246705.4	ENSP00000438553.1	119.8	387
KFTPVASKF	9	0.0207	ZYX	ENSG00000159840.16	ENSP00000376642.2	118.7	1245
VYGQESGGF	9	0.2200	EWSR1	ENSG00000182944.17	ENSP00000331699.6	167.7	1800
KYWDVPPPGF	10	0.0536	U2AF2	ENSG00000063244.12	ENSP00000388475.1	133.4	1413
YISEHEHF	8	0.4765	CLPTM1	ENSG00000104853.16	ENSP00000443192.1	114.6	1701
VYISEHEHF	9	0.0020	CLPTM1	ENSG00000104853.16	ENSP00000443192.1	114.6	1701
EVIKQLKEKM	10	22.2632	SBDS	ENSG00000126524.10	ENSP00000246868.2	97.1	750
YYEEQHPEL	9	0.1204	IL32	ENSG00000008517.16	ENSP00000433866.3	460.4	663
EVIQHRFESL	10	14.7111	SPTBN1	ENSG00000115306.16	ENSP00000334156.5	262.0	6465
KWNPTAGVAF	10	0.1926	POLR2C	ENSG00000102978.13	ENSP00000219252.4	100.6	825

YFKDTHPKF	9	0.0239	CYB5R3	ENSG00000100243.21	ENSP00000338461.6	176.1	903
EIISHDTRR	9	39.0000	CYB5R3	ENSG00000100243.21	ENSP00000338461.6	176.1	903
LYADVGGKQF	10	0.1143	SSR4	ENSG00000180879.13	ENSP00000359103.3	246.5	519
IQYALRTL	8	7.6586	SARS	ENSG00000031698.13	ENSP00000234677.2	139.0	1542
EVMQEVAQL	9	6.5793	SARS	ENSG00000031698.13	ENSP00000234677.2	139.0	1542
KYGSNVHRI	9	0.0247	PPP1R9B	ENSG00000108819.11	ENSP00000478767.1	102.3	2451
AYLEAIHNF	9	0.0009	AP2S1	ENSG00000042753.11	ENSP00000263270.5	135.9	426
VYTVVDEMF	9	0.0213	AP2S1	ENSG00000042753.11	ENSP00000263270.5	135.9	426
SYGGPASQQL	10	0.2050	HNRNPH2	ENSG00000126945.9	ENSP00000361927.2	85.7	1347
KYGNLSNYL	9	0.1072	FLT1	ENSG00000102755.12	ENSP00000282397.4	129.2	4014
RYVRKFVLM	9	0.0737	CHMP2A	ENSG00000130724.8	ENSP00000469240.1	160.5	666
EYVNDFDLMKF	11	0.2465	MAF	ENSG00000178573.7	ENSP00000377019.1	103.5	1119
SMMGSDMRTERF	12	3.3376	SFPQ	ENSG00000116560.11	ENSP00000349748.5	206.9	2121
QYLSRFESW	9	0.0096	BHLHE41	ENSG00000123095.6	ENSP00000242728.4	89.5	1446
RYRGDYDRF	9	0.1135	HNRNPUL2	ENSG00000214753.3	ENSP00000301785.5	86.5	2241
NYIEKVRFL	9	0.0724	DES	ENSG00000175084.11	ENSP00000363071.3	84.4	1410
LYIVHPTMF	9	0.0088	ARHGAP1	ENSG00000175220.12	ENSP00000310491.4	86.7	1317
NYTPYKYNL	9	0.0744	HGD	ENSG00000113924.12	ENSP00000283871.5	143.1	1335
SYKNGFLNL	9	0.0659	UBA1	ENSG00000130985.17	ENSP00000338413.6	224.2	3174
EIVSRVSKR	9	28.1250	UBA1	ENSG00000130985.17	ENSP00000338413.6	224.2	3174
EVVDFIQSKI	10	21.8182	PPM1G	ENSG00000115241.11	ENSP00000342778.4	82.2	1638
LQLQIPRI	8	12.0903	PSME1	ENSG00000092010.15	ENSP00000206451.6	279.0	747
LYDIILKNF	9	0.0433	PSME1	ENSG00000092010.15	ENSP00000206451.6	279.0	747
VFMKSVKLEW	10	0.3481	PRPF6	ENSG00000101161.8	ENSP00000266079.4	79.8	2823
KYPENFFLL	9	0.0032	PPP1CA	ENSG00000172531.15	ENSP00000350974.4	151.6	858
EVVAKFLHK	9	21.3636	PPP1CA	ENSG00000172531.15	ENSP00000350974.4	151.6	858
YFAERVTSL	9	0.3110	CRIP1	ENSG00000213145.9	ENSP00000332449.7	228.2	231

VYFAERVTSL	10	0.2413	CRIP1	ENSG00000213145.9	ENSP00000332449.7	228.2	231
KYLDNPNAL	9	0.0499	KANK2	ENSG00000197256.10	ENSP00000465650.1	97.8	2553
EVADPTAHR	9	11.2283	KANK2	ENSG00000197256.10	ENSP00000465650.1	97.8	2553
AYTTVLQEW	9	0.0210	KANK2	ENSG00000197256.10	ENSP00000465650.1	97.8	2553
EVADPTAHRR	10	20.5926	KANK2	ENSG00000197256.10	ENSP00000465650.1	97.8	2553
QYESKVFYL	9	0.1158	YWHAG	ENSG00000170027.7	ENSP00000306330.3	78.0	741
RYDEHAYKW	9	0.0111	BBOX1	ENSG00000129151.9	ENSP00000433772.1	80.3	1161
QYSRFSLENNF	11	0.2339	STAT1	ENSG00000115415.18	ENSP00000438703.1	107.1	579
KYVKILYDF	9	0.0095	EPS8L2	ENSG00000177106.16	ENSP00000436035.1	265.7	2145
RYVAGIEGF	9	0.0228	SEL1L3	ENSG00000091490.11	ENSP00000425438.1	135.2	2937
YYITGNLETF	10	0.0090	SEL1L3	ENSG00000091490.11	ENSP00000425438.1	135.2	2937
TYQEVAQKF	9	0.0014	ADA2	ENSG00000093072.18	ENSP00000496894.1	143.9	207
KFVETHPEF	9	0.0294	ADA2	ENSG00000093072.18	ENSP00000496894.1	143.9	207
YYHARVYEF	9	0.0023	PSMD3	ENSG00000108344.15	ENSP00000264639.4	89.9	1602
RYIKKLAKW	9	0.0163	CNDP2	ENSG00000133313.15	ENSP00000325548.4	206.8	1425
KYQEEFEHF	9	0.0016	LMAN1	ENSG00000074695.6	ENSP00000251047.4	77.5	1530
GQRYFLNHI	9	9.7647	WWTR1	ENSG00000018408.14	ENSP00000419465.1	88.5	1200
IFSNVRINL	9	0.4230	CAV1	ENSG00000105974.12	ENSP00000377110.1	92.6	441
SQIPLSKI	8	10.3550	XRCC5	ENSG00000079246.16	ENSP00000375977.2	106.7	2196
VYEGPELNHAF	11	0.0416	LRP1	ENSG00000123384.13	ENSP00000243077.3	104.7	13632
QQIRINEV	8	20.7407	TWF2	ENSG00000247596.9	ENSP00000303908.4	72.6	1047
EVAPPEYHRK	10	20.9259	MORF4L1	ENSG00000185787.14	ENSP00000408880.2	174.3	969
KYLAKNSATLF	11	0.0584	MORF4L1	ENSG00000185787.14	ENSP00000408880.2	174.3	969
KYQEVTNNL	9	0.0116	CAPRIN1	ENSG00000135387.21	ENSP00000434150.1	106.8	2127
KYQEVTNNLEF	11	0.0154	CAPRIN1	ENSG00000135387.21	ENSP00000434150.1	106.8	2127
TVVEDVTVGR	10	28.0000	ZFYVE21	ENSG00000100711.13	ENSP00000310543.2	83.2	702
SYKYEHLRW	9	0.1621	FLT4	ENSG00000037280.16	ENSP00000261937.6	92.8	4089

KYGNLSNFL	9	0.1237	FLT4	ENSG00000037280.16	ENSP00000261937.6	92.8	4089
KFYTDPSYF	9	0.0750	WASF2	ENSG00000158195.11	ENSP00000483313.1	69.3	1494
YYFEGIKQTF	10	0.0027	AHSA1	ENSG00000100591.8	ENSP00000216479.3	93.8	1014
IYISTLKTEF	10	0.0238	AHSA1	ENSG00000100591.8	ENSP00000216479.3	93.8	1014
EYSKQMQRF	9	0.0416	HDAC1	ENSG00000116478.12	ENSP00000362649.3	107.2	1446
VQLRLNSI	8	12.9167	PPP2R1A	ENSG00000105568.18	ENSP00000324804.6	139.0	1767
KYVPAIAHL	9	0.0073	CPVL	ENSG00000106066.15	ENSP00000265394.5	75.9	1428
KYVPAIAHLIHS	12	6.9851	CPVL	ENSG00000106066.15	ENSP00000265394.5	75.9	1428
HQNVFPNHI	9	2.8119	ANKRD10	ENSG00000088448.14	ENSP00000267339.2	162.9	1260
QYLPHVARL	9	0.0178	SURF4	ENSG00000148248.14	ENSP00000361057.3	72.6	807
KYLTAEAFGF	10	0.1492	FSCN1	ENSG00000075618.18	ENSP00000371798.3	132.9	1479
HFIAEHYMF	9	0.0437	DEGS1	ENSG00000143753.13	ENSP00000316476.4	76.2	969
DTISPYSRM	9	6.3865	DEGS1	ENSG00000143753.13	ENSP00000316476.4	76.2	969
KYPEIKSLM	9	0.0567	DEGS1	ENSG00000143753.13	ENSP00000316476.4	76.2	969
AYDTVRWEF	9	0.0303	DPP7	ENSG00000176978.14	ENSP00000360635.2	266.4	1476
EYALLYHTL	9	0.0333	AOC1	ENSG00000002726.20	ENSP00000411613.2	149.2	2310
FFKPHWDEKF	10	0.2620	SERPINH1	ENSG00000149257.14	ENSP00000434412.1	82.4	1254
YLEKPMEI	8	1.7088	STAT3	ENSG00000168610.14	ENSP00000264657.4	111.6	2310
RYLEKPMEI	9	0.0074	STAT3	ENSG00000168610.14	ENSP00000264657.4	111.6	2310
RYLEQLHQL	9	0.0039	STAT3	ENSG00000168610.14	ENSP00000264657.4	111.6	2310
IYQRDPLKL	9	0.0232	STAT6	ENSG00000166888.12	ENSP00000451546.2	228.6	2595
AYNEGIINLL	10	0.2395	PICALM	ENSG00000073921.18	ENSP00000433846.1	156.3	1935
RYLQSTQAI	9	0.0388	SETD3	ENSG00000183576.13	ENSP00000327436.5	67.7	1782
PYKVTQDEL	9	0.5289	NCL	ENSG00000115053.16	ENSP00000318195.4	161.2	2130
AVVDVRIGMTR	11	31.3333	NCL	ENSG00000115053.16	ENSP00000318195.4	161.2	2130
VVTNRFDQL	9	3.6831	SERBP1	ENSG00000142864.14	ENSP00000360034.2	104.1	1224
GQWKKGFVL	9	2.7258	SERBP1	ENSG00000142864.14	ENSP00000360034.2	104.1	1224

VVTNRFDQLF	10	3.1855	SERBP1	ENSG00000142864.14	ENSP00000360034.2	104.1	1224
EYTDVYPEI	9	0.0982	MAGED2	ENSG00000102316.17	ENSP00000364198.1	172.8	1818
AEPAVQRTLLEK	12	45.0000	CD99	ENSG00000002586.20	ENSP00000485427.1	115.0	552
RWFDKSFTF	9	0.0040	CPT1A	ENSG00000110090.13	ENSP00000265641.4	66.6	2319
RYPDRITLI	9	0.0115	PPP4C	ENSG00000149923.14	ENSP00000455995.1	96.7	921
RYASINTHL	9	0.0285	CSNK1E	ENSG00000213923.12	ENSP00000380044.1	279.7	1248
RYFIAPAKF	9	0.0053	LAMC1	ENSG00000135862.6	ENSP00000258341.3	66.1	4827
TYTDVTPRQF	10	0.0234	STARD7	ENSG00000084090.13	ENSP00000338030.5	78.2	1110
TYTDVTPRQFF	11	0.0277	STARD7	ENSG00000084090.13	ENSP00000338030.5	78.2	1110
VYGTVFHI	8	0.4262	GLYAT	ENSG00000149124.11	ENSP00000278400.3	89.0	489
HVIVDEIHER	10	27.7000	DHX9	ENSG00000135829.17	ENSP00000356520.3	76.8	3810
YIDRVRSL	8	2.5583	KRT18	ENSG00000111057.11	ENSP00000373487.3	125.9	1290
RYALQMEQL	9	0.0971	KRT18	ENSG00000111057.11	ENSP00000373487.3	125.9	1290
TYLVDMRRF	9	0.0387	FZD1	ENSG00000157240.3	ENSP00000287934.2	59.1	1941
KFIWTNHKF	9	0.0245	NDUFA12	ENSG00000184752.13	ENSP00000330737.2	68.3	435
SYGKVTAEF	9	0.0034	ALPK2	ENSG00000198796.7	ENSP00000354991.3	83.9	6510
ETVGFGMLK	9	33.5000	LAMTOR2	ENSG00000116586.11	ENSP00000357288.4	122.1	375
EVGVGFATR	9	36.0000	FABP4	ENSG00000170323.8	ENSP00000256104.4	63.0	396
IYIAGHPAF	9	0.0099	HNRNPL	ENSG00000104824.17	ENSP00000221419.4	167.1	1767
SQFSYQHAI	9	2.5023	EIF3H	ENSG00000147677.11	ENSP00000428195.1	142.6	732
VYHHLVETL	9	0.0106	GGT5	ENSG00000099998.17	ENSP00000381340.3	78.5	1761
YIKHPVSL	8	1.2395	PSMD8	ENSG00000099341.11	ENSP00000215071.4	103.0	1050
VYIKHPVSL	9	0.0059	PSMD8	ENSG00000099341.11	ENSP00000215071.4	103.0	1050
TYTLRVFEL	9	0.0961	PTPRC	ENSG00000081237.20	ENSP00000306782.7	86.9	3435
ESIGRVLAQR	10	39.5000	MRPL18	ENSG00000112110.10	ENSP00000356001.4	62.9	540
IYNHITTRV	9	0.1704	ADD1	ENSG00000087274.17	ENSP00000348100.3	145.3	1986
EYATKISRF	9	0.0315	PITHD1	ENSG00000057757.10	ENSP00000246151.4	62.3	633

QYAPSTAQF	9	0.0024	ZMIZ2	ENSG00000122515.15	ENSP00000265346.7	116.9	2682
MQVLVSRI	8	20.1852	BCL2L1	ENSG00000171552.13	ENSP00000365230.2	100.1	699
TYGGSWKF	8	0.1706	AIG1	ENSG00000146416.18	ENSP00000350509.4	88.3	714
AYVPGFAHI	9	0.0192	CUEDC2	ENSG00000107874.11	ENSP00000358953.4	81.2	861
HQITVLHV	8	19.0968	ELOVL5	ENSG00000012660.14	ENSP00000306640.6	71.9	897
KYITQGQLL	9	0.0246	ELOVL5	ENSG00000012660.14	ENSP00000306640.6	71.9	897
KYITQGQLLQF	11	0.0263	ELOVL5	ENSG00000012660.14	ENSP00000306640.6	71.9	897
KYSKRFPEL	9	0.0329	PRPF31	ENSG00000105618.14	ENSP00000375635.1	77.5	1479
FVVEKVLDR	9	27.9000	CBX3	ENSG00000122565.19	ENSP00000387348.1	104.4	303
SVFPQNGAAR	10	17.0667	KAT5	ENSG00000172977.13	ENSP00000434765.1	94.0	1482
KQMRILMV	8	14.7778	ARF5	ENSG00000004059.11	ENSP00000000233.5	123.1	540
IYPELQIERF	10	0.0069	APBB1IP	ENSG00000077420.16	ENSP00000365411.4	56.6	1998
QYVTQINRL	9	0.0954	PREX1	ENSG00000124126.14	ENSP00000361009.3	84.4	4977
VFHPSQDLVF	10	0.1043	PRPF19	ENSG00000110107.9	ENSP00000227524.4	81.6	1512
VYSPHVLNL	9	0.0042	DNM2	ENSG00000079805.16	ENSP00000352721.6	157.6	2598
VYSPHVLNLTL	11	0.0719	DNM2	ENSG00000079805.16	ENSP00000352721.6	157.6	2598
EVIPPYYSY	9	2.6495	TDP2	ENSG00000111802.14	ENSP00000367440.4	72.5	1086
IYLEKLKTI	9	0.0161	PLEC	ENSG00000178209.15	ENSP00000434583.1	86.0	13710
YVIDPIKGL	9	3.7410	PLEC	ENSG00000178209.15	ENSP00000434583.1	86.0	13710
RYLSPKYIKMF	11	0.0459	EIF4A1	ENSG00000161960.15	ENSP00000293831.8	133.8	1218
KQMRILHV	8	10.6412	GNAS	ENSG00000087460.25	ENSP00000360136.3	672.6	1140
KYQKGFSLW	9	0.0098	TRAM1	ENSG00000067167.8	ENSP00000262213.2	70.6	1122
AYLLNLNHL	9	0.0529	TRAM1	ENSG00000067167.8	ENSP00000262213.2	70.6	1122
AYPHNLMTF	9	0.0041	TRAM1	ENSG00000067167.8	ENSP00000262213.2	70.6	1122
VYSEVHFTL	9	0.0025	SYNPO	ENSG00000171992.13	ENSP00000302139.4	62.7	2709
SYLERYQRF	9	0.0021	TNK2	ENSG00000061938.18	ENSP00000329425.6	58.9	3114
YLENALKL	8	2.0426	GBP4	ENSG00000162654.9	ENSP00000359490.5	60.9	1920

EYLENALKL	9	0.1132	GBP4	ENSG00000162654.9	ENSP00000359490.5	60.9	1920
YYIDKLEYL	9	0.0077	NET1	ENSG00000173848.19	ENSP00000369717.3	62.0	1626
KYMAEALLL	9	0.0244	TOMM70	ENSG00000154174.7	ENSP00000284320.5	53.0	1824
FQIQISRI	8	12.4722	OSMR	ENSG00000145623.13	ENSP00000274276.3	57.2	2937
RYHSDFNRQDRF	12	0.3049	SAFB	ENSG00000160633.13	ENSP00000415895.1	102.0	2544
IIGLQMGTNKF	11	4.6650	CNN1	ENSG00000130176.8	ENSP00000252456.1	92.5	891
RYMSINTHL	9	0.0176	CSNK1G2	ENSG00000133275.16	ENSP00000255641.7	63.6	1245
RYVRRLDFF	9	0.0371	CSNK1G2	ENSG00000133275.16	ENSP00000255641.7	63.6	1245
RYMPQNPHI	9	0.0093	RBBP7	ENSG00000102054.17	ENSP00000386068.1	76.8	1248
RYMPQNPHII	10	0.0583	RBBP7	ENSG00000102054.17	ENSP00000386068.1	76.8	1248
VMSQALKATF	10	1.4321	ETS2	ENSG00000157557.13	ENSP00000499315.1	102.8	1407
LYRLKFNEF	9	0.1612	ARL4C	ENSG00000188042.8	ENSP00000375057.2	49.9	576
NYPETLGRLLI	11	0.5073	SEC14L1	ENSG00000129657.16	ENSP00000389838.1	125.4	2043
TQFKWIHI	8	5.9304	KHK	ENSG00000138030.13	ENSP00000260598.5	93.9	894
TQRIFQEAV	9	18.5789	NRARP	ENSG00000198435.4	ENSP00000349041.2	49.1	342
YYLTDVDRI	9	0.1693	GNA11	ENSG00000088256.9	ENSP00000078429.3	55.2	1077
YVVEKVLDR	9	21.2727	CBX1	ENSG00000108468.15	ENSP00000225603.4	50.4	555
IYTSSVNRL	9	0.0348	COPB2	ENSG00000184432.10	ENSP00000422295.1	83.6	2631
TYMDAQLFKKV	11	0.8788	RGL1	ENSG00000143344.15	ENSP00000354097.3	49.0	2304
TYQDIQNTI	9	0.0114	POLR2A	ENSG00000181222.16	ENSP00000499521.1	62.9	5910
AYPYNFSNL	9	0.1366	ZBTB4	ENSG00000174282.12	ENSP00000369973.4	49.1	3039
KYSLVKNKI	9	0.0825	S100A16	ENSG00000188643.11	ENSP00000357694.1	113.0	309
NYLVLPNRI	9	0.0900	ESYT2	ENSG00000117868.16	ENSP00000499020.1	54.4	2679
IYLLIHNNF	9	0.0094	CD53	ENSG00000143119.14	ENSP00000271324.5	108.1	657
IFKPPDPDNTF	11	0.1027	MOAP1	ENSG00000165943.5	ENSP00000298894.4	49.6	1053
YYDKHFTEF	9	0.0045	ATP6V1A	ENSG00000114573.10	ENSP00000273398.3	48.2	1851
EYYDKHFTEF	10	0.0239	ATP6V1A	ENSG00000114573.10	ENSP00000273398.3	48.2	1851

SMFQVRTL	8	6.5233	CD36	ENSG00000135218.19	ENSP00000308165.7	102.3	1416
EYMYQVMKF	9	0.0145	MIOX	ENSG00000100253.13	ENSP00000216075.6	81.8	855
VYNANINLF	9	0.0025	PKD2	ENSG00000118762.8	ENSP00000237596.2	51.6	2904
YYTKGFALL	9	0.0233	PLOD2	ENSG00000152952.12	ENSP00000353170.3	52.5	2211
PYMANVYLI	9	0.0795	PLOD2	ENSG00000152952.12	ENSP00000353170.3	52.5	2211
VYVYGVDRF	9	0.0215	SLC6A19	ENSG00000174358.16	ENSP00000305302.10	47.9	1902
SVIGFRATQR	10	25.6667	SLC6A19	ENSG00000174358.16	ENSP00000305302.10	47.9	1902
FVIDSDHLGHR	11	30.6667	COASY	ENSG00000068120.15	ENSP00000393564.2	60.6	1692
TQYRAMFV	8	27.0000	EAPP	ENSG00000129518.9	ENSP00000250454.3	49.8	855
EAAQKFLER	9	18.6579	KPNB1	ENSG00000108424.10	ENSP00000290158.3	74.6	2628
TYQRWQFTL	9	0.0189	PRPF8	ENSG00000174231.17	ENSP00000304350.6	123.1	7005
EIRDIILGM	9	17.3556	PRPF8	ENSG00000174231.17	ENSP00000304350.6	123.1	7005
RYIDRIHIF	9	0.0010	PRPF8	ENSG00000174231.17	ENSP00000304350.6	123.1	7005
VYTTTVHWL	9	0.0444	PRPF8	ENSG00000174231.17	ENSP00000304350.6	123.1	7005
RYIDRIHIFF	10	0.0048	PRPF8	ENSG00000174231.17	ENSP00000304350.6	123.1	7005
RYPTSIASL	9	0.0318	BUB3	ENSG00000154473.18	ENSP00000357851.5	68.7	978
RYPTSIASLAF	11	0.0548	BUB3	ENSG00000154473.18	ENSP00000357851.5	68.7	978
SYTHIQYLF	9	0.0022	SH2D3C	ENSG00000095370.20	ENSP00000485866.1	59.5	2100
YYVTELTHRI	10	0.2118	GBP1	ENSG00000117228.10	ENSP00000359504.4	62.9	1776
VYGFQWRHF	9	0.0350	TYMS	ENSG00000176890.16	ENSP00000315644.10	67.7	939
EVINYEPIGY	10	37.6667	MYLK	ENSG00000065534.18	ENSP00000418335.1	104.4	5742
IYGLLKNPF	9	0.1190	YME1L1	ENSG00000136758.18	ENSP00000318480.3	54.2	2319
KYINTDAKF	9	0.0121	TRPC4AP	ENSG00000100991.12	ENSP00000252015.2	64.1	2391
TYGRSFTL	8	0.6139	CHIT1	ENSG00000133063.16	ENSP00000423778.1	71.6	1161
YAHIYHQHF	9	0.7868	MOB1A	ENSG00000114978.18	ENSP00000379364.3	47.2	648
VYAHIYHQHF	10	0.0089	MOB1A	ENSG00000114978.18	ENSP00000379364.3	47.2	648
RYMEVSGNL	9	0.1194	SF3A3	ENSG00000183431.12	ENSP00000362110.4	62.7	1503

YYQHIVTTL	9	0.0054	PCYOX1	ENSG00000116005.12	ENSP00000387654.2	44.5	1515
TYIESASEL	9	0.0940	GALNT14	ENSG00000158089.15	ENSP00000385435.1	152.7	1596
SYILRPVAF	9	0.0127	SLC28A1	ENSG00000156222.12	ENSP00000286749.3	77.2	1947
KYPLLLDNI	9	0.1412	ARHGEF12	ENSG00000196914.9	ENSP00000432984.1	52.8	4323
SYFDEPVEL	9	0.0327	ARFGAP3	ENSG00000242247.11	ENSP00000263245.5	46.5	1548
RYPNRFKLW	9	0.0463	CYB5R1	ENSG00000159348.13	ENSP00000356218.4	62.5	915
SYFPTPAAL	9	0.0414	FOLR2	ENSG00000165457.14	ENSP00000298223.6	58.8	765
SYIAHLRQI	9	0.0574	MSC	ENSG00000178860.8	ENSP00000321445.4	46.4	618
KYPNVDARL	9	0.1058	UGCG	ENSG00000148154.10	ENSP00000363397.3	45.5	1182
EAILGTIGAR	10	48.0000	DOCK6	ENSG00000130158.14	ENSP00000294618.6	104.3	6141
VQRLEPVI	8	18.5526	PFKFB3	ENSG00000170525.21	ENSP00000492001.1	72.5	1704
VYLIEHPSL	9	0.0372	ASPA	ENSG00000108381.11	ENSP00000263080.2	43.6	939
VYLQNWSHVL	10	0.1293	GPS1	ENSG00000169727.12	ENSP00000485035.1	83.5	1578
VYLPTHTSL	9	0.0083	MYLIP	ENSG00000007944.15	ENSP00000349298.3	50.5	1335
YMKHTRLF	8	0.5788	FLII	ENSG00000177731.16	ENSP00000324573.4	128.0	3807
EYMKHTRLF	9	0.0160	FLII	ENSG00000177731.16	ENSP00000324573.4	128.0	3807
SYQRAFNEF	9	0.0054	TNFAIP2	ENSG00000185215.9	ENSP00000452634.1	112.3	1962
EVITAVRKM	9	8.2245	COL6A3	ENSG00000163359.15	ENSP00000418285.1	49.2	7710
QYVSAFSKL	9	0.0686	STOML2	ENSG00000165283.16	ENSP00000348886.5	113.4	1068
EVVQKMTGL	9	14.5889	CTSK	ENSG00000143387.13	ENSP00000271651.3	43.1	987
RYLPKLKAF	9	0.0169	CRY2	ENSG00000121671.11	ENSP00000478187.1	50.8	1842
MYANHFDLL	9	0.0800	FMO1	ENSG00000010932.17	ENSP00000481732.1	84.4	1596
VYFSAIQKI	9	0.0061	BAIAP2L2	ENSG00000128298.17	ENSP00000371085.3	44.1	1587
IYKSIMEQF	9	0.0032	BAIAP2L2	ENSG00000128298.17	ENSP00000371085.3	44.1	1587
HYAYSFKYL	9	0.1651	SUPT6H	ENSG00000109111.15	ENSP00000319104.6	51.0	5178
SFLKAKVI	8	4.5604	RBM42	ENSG00000126254.12	ENSP00000467278.1	64.3	1374
EVAQLIQGGR	10	60.0000	MPC1	ENSG00000060762.19	ENSP00000354223.6	80.4	327

RYQERFKHI	9	0.0756	SPRYD3	ENSG00000167778.9	ENSP00000301463.4	44.7	1326
SQANIAQVL	9	3.4422	AP1B1	ENSG00000100280.16	ENSP00000319361.7	90.8	2757
VYDVVELKF	9	0.0183	CPSF7	ENSG00000149532.15	ENSP00000397203.3	65.0	1386
EVFNKFFKER	10	25.0833	SUPT16H	ENSG00000092201.10	ENSP00000216297.2	51.2	3141
TYLPAPEGLKF	11	0.0056	TNC	ENSG00000041982.16	ENSP00000443478.1	51.3	4692
FYLEGGFSKF	10	0.0183	DUSP6	ENSG00000139318.8	ENSP00000279488.6	48.0	1143
RYLNEFEEL	9	0.0243	EIF2AK1	ENSG00000086232.13	ENSP00000199389.6	43.6	1890
RYAQNVAFF	9	0.0039	ZNF32	ENSG00000169740.14	ENSP00000363556.2	52.3	819
KYKGIFNGF	9	0.0226	SLC25A3	ENSG00000075415.12	ENSP00000383898.3	107.3	1083
KYIHSANVL	9	0.0925	MAPK1	ENSG00000100030.14	ENSP00000215832.6	42.8	1080
NQYRLIVNV	9	8.1306	MCM3	ENSG00000112118.19	ENSP00000480987.1	45.9	2559
RYLEVMRKL	9	0.0208	PTPA	ENSG00000119383.19	ENSP00000351885.4	118.4	969
NYADQISRL	9	0.1401	MAPK8IP3	ENSG00000138834.12	ENSP00000348290.5	99.9	3990
EQLSILKV	8	22.4211	MYH11	ENSG00000133392.18	ENSP00000300036.5	135.9	5916
TQRQVNITV	9	9.7676	HACD3	ENSG00000074696.13	ENSP00000261875.5	40.4	1086
LYANMFERL	9	0.0553	FKBP4	ENSG00000004478.8	ENSP00000001008.4	48.5	1377
DVISSIRNF	9	1.7773	TOMM5	ENSG00000175768.13	ENSP00000313584.6	40.9	153
KYRRYHPLF	9	0.1047	ARAP1	ENSG00000186635.14	ENSP00000335506.8	116.3	3615
EMISDEIHER	10	24.2857	SH3BP5	ENSG00000131370.16	ENSP00000373301.3	82.2	1365
KYTKNIVSW	9	0.0096	ARHGAP29	ENSG00000137962.13	ENSP00000260526.6	47.5	3783
EVATFFAKM	9	8.7311	TOP1	ENSG00000198900.6	ENSP00000354522.2	38.5	2295
TYTDRVFFL	9	0.0326	PLXNB2	ENSG00000196576.15	ENSP00000409171.1	401.3	5514
YYTDIMHTL	9	0.0046	PLXNB2	ENSG00000196576.15	ENSP00000409171.1	401.3	5514
VYVVGTAHF	9	0.0062	TRABD	ENSG00000170638.9	ENSP00000379171.1	52.1	1128
YFHPPAHL	8	0.8542	MBNL1	ENSG00000152601.17	ENSP00000418427.1	69.1	906
KYFHPPAHL	9	0.0180	MBNL1	ENSG00000152601.17	ENSP00000418427.1	69.1	906
QQHVIETLI	9	7.1052	CCT5	ENSG00000150753.12	ENSP00000280326.4	120.4	1623

ETAGRYITY	9	8.2978	POLR2E	ENSG00000099817.12	ENSP00000478303.1	72.6	630
IYSIDFTRF	9	0.0066	UNC119B	ENSG00000175970.11	ENSP00000344942.4	41.2	753
EVVERLLSV	9	12.5972	INF2	ENSG00000203485.13	ENSP00000376406.3	95.6	3720
IQHLIPKI	8	9.3447	PSME2	ENSG00000100911.16	ENSP00000216802.5	113.3	717
GPREAGLR	8	80.0000	CCDC115	ENSG00000136710.10	ENSP00000387301.1	55.4	525
EVGPREAGLR	10	52.5000	CCDC115	ENSG00000136710.10	ENSP00000387301.1	55.4	525
DVINPMALR	9	27.8000	SART1	ENSG00000175467.15	ENSP00000310448.5	41.7	2400
RYTVGGLETF	10	0.0291	PTPN6	ENSG00000111679.17	ENSP00000326010.9	119.5	1785
KYLEMIYSM	9	0.0251	IPO7	ENSG00000205339.10	ENSP00000369042.3	42.5	3114
EYAVLTSTI	9	0.1604	IRF2	ENSG00000168310.11	ENSP00000377218.3	44.0	1047
ETAPRTIFQR	10	23.9375	ATXN10	ENSG00000130638.17	ENSP00000252934.4	41.2	1425
KYLAHLEQL	9	0.0292	GBP2	ENSG00000162645.13	ENSP00000359497.3	89.1	1773
RYLTGAWRL	9	0.0240	ALG5	ENSG00000120697.9	ENSP00000239891.3	46.2	972
YYEKQFPEI	9	0.0863	NCOR2	ENSG00000196498.13	ENSP00000400281.2	82.0	7512
VYPQPGMQRF	10	0.0053	BAG3	ENSG00000151929.9	ENSP00000358081.3	39.7	1725
WYMDNPQNL	9	0.0562	PLEKHA2	ENSG00000169499.15	ENSP00000482228.1	49.6	1275
TQFLPRTI	8	6.9179	PDZD11	ENSG00000120509.10	ENSP00000239666.4	61.5	420
IYIKHPHLF	9	0.0006	DNTTIP1	ENSG00000101457.13	ENSP00000361705.3	40.7	987
EYLTKVDKL	9	0.1835	CLTC	ENSG00000141367.11	ENSP00000376763.1	95.7	4917
AYVEMLQHL	9	0.0160	FGD5	ENSG00000154783.11	ENSP00000285046.5	40.9	4386
KYADAVKNF	9	0.0051	RASA3	ENSG00000185989.11	ENSP00000335029.7	36.7	2502
VYAAGSQFHSF	11	0.0123	GPT	ENSG00000167701.14	ENSP00000378408.2	66.4	1488
RWFDKTLQF	9	0.0068	CRAT	ENSG00000095321.17	ENSP00000315013.2	55.6	1878
PADLSRPK	8	90.0000	MAP4	ENSG00000047849.21	ENSP00000379083.3	57.5	3405
VYIDRVRSL	9	0.0290	LMNA	ENSG00000160789.20	ENSP00000357283.4	119.4	1992
RFIRPMGLRF	10	0.1038	NSA2	ENSG00000164346.10	ENSP00000483484.1	36.5	780
KYMPSVKVSVF	11	0.0222	DDX39A	ENSG00000123136.14	ENSP00000242776.3	72.0	1281

MYIARQLSF	9	0.0080	SBNO2	ENSG00000064932.16	ENSP00000400762.1	59.0	3927
AVIHLALKER	10	46.0000	SF3A1	ENSG00000099995.19	ENSP00000215793.7	76.4	2379
VYIHHFDRI	9	0.0327	MOB3A	ENSG00000172081.14	ENSP00000349575.2	61.8	651
EVIPPMKEF	9	1.2290	NDUFB6	ENSG00000165264.11	ENSP00000369176.3	40.6	384
VYSSAEFHSL	10	0.1182	LTBP3	ENSG00000168056.16	ENSP00000435276.1	208.9	3408
QYPVIIHLI	9	0.0054	ELOVL1	ENSG00000066322.15	ENSP00000361536.3	74.1	837
DIYSGGGGGGSR	12	65.0000	HNRNPA0	ENSG00000177733.6	ENSP00000316042.4	34.9	915
RYNPENNTW	9	0.0220	IVNS1ABP	ENSG00000116679.16	ENSP00000356468.3	535.7	1926
VPYPLPKI	8	9.7088	NPEPPS	ENSG00000141279.16	ENSP00000320324.4	60.5	2757
ESIHQYLLQR	10	41.5000	NPEPPS	ENSG00000141279.16	ENSP00000320324.4	60.5	2757
AIVDVVANR	9	17.4222	ANXA7	ENSG00000138279.16	ENSP00000362012.4	73.9	1398
IYAGGIKSI	9	0.0969	SLC12A4	ENSG00000124067.17	ENSP00000318557.3	80.8	3255
HVITGLLEHY	10	18.7632	SCRN2	ENSG00000141295.14	ENSP00000383935.3	83.3	1134
RYVQQLQRL	9	0.0226	MRPL43	ENSG00000055950.16	ENSP00000299179.5	99.1	606
EVNPNTRVM	9	7.9958	ORMDL1	ENSG00000128699.14	ENSP00000326869.3	52.7	459
RYLPQTYVV	9	0.0334	ITM2A	ENSG00000078596.11	ENSP00000362395.2	77.3	789
AFKHKHHLI	9	0.6469	ZEB2	ENSG00000169554.21	ENSP00000302501.4	72.4	3639
HYLDTTTLI	9	0.0353	CRKL	ENSG00000099942.13	ENSP00000346300.3	34.4	909
RYFYKVHQL	9	0.0092	BSDC1	ENSG00000160058.18	ENSP00000397759.2	71.8	1341
KYVESFRRF	9	0.0054	HNMT	ENSG00000150540.14	ENSP00000280097.3	59.8	876
EVMKAVKVGM	10	29.5714	PEPD	ENSG00000124299.15	ENSP00000391890.2	89.2	1287
QYQKILERL	9	0.0217	TAB1	ENSG00000100324.14	ENSP00000216160.6	36.6	1512
RYISQTQGL	9	0.0723	HIPK2	ENSG00000064393.16	ENSP00000385571.3	35.0	3594
KYQEIPFYHI	10	0.0533	UNC5B	ENSG00000107731.12	ENSP00000334329.6	34.8	2835
NYQKRFQNL	9	0.0611	OGFR	ENSG00000060491.16	ENSP00000359491.1	62.4	1875
VYNENLVHMI	10	0.2172	BCAS2	ENSG00000116752.6	ENSP00000358554.3	35.2	675
KQALKYFNL	9	1.2698	SEL1L	ENSG00000071537.14	ENSP00000337053.4	40.0	2382

DVVSKFLNR	9	19.4839	PPP1CB	ENSG00000213639.10	ENSP00000351298.2	68.6	981
NYGRVFSEW	9	0.0341	SNX4	ENSG00000114520.11	ENSP00000251775.4	35.5	1350
SYNDYVREF	9	0.0084	YTHDC1	ENSG00000083896.12	ENSP00000463982.1	78.7	2205
VWDTAGQEKF	10	0.2912	RAN	ENSG00000132341.12	ENSP00000446215.1	47.3	648
KYMDINFDF	9	0.0063	MYO1D	ENSG00000176658.17	ENSP00000324527.5	47.8	3018
YGEIFEKF	8	0.2399	NDUFC2	ENSG00000151366.13	ENSP00000281031.4	45.6	357
VQPGQNFHMF	10	0.3125	PSMA5	ENSG00000143106.13	ENSP00000440618.1	50.0	549
TVQPGQNFHMF	11	0.9652	PSMA5	ENSG00000143106.13	ENSP00000440618.1	50.0	549
AYFQKPLLL	9	0.0125	AGXT2	ENSG00000113492.14	ENSP00000231420.6	63.0	1542
AYARIGNSYF	10	0.0904	STIP1	ENSG00000168439.16	ENSP00000445957.1	62.3	1557
AYLEALSHL	9	0.0271	PIK3CD	ENSG00000171608.15	ENSP00000366563.4	38.1	3132
RYLDELMKL	9	0.0132	IQGAP1	ENSG00000140575.13	ENSP00000488618.1	132.1	3573
AYQDRLAYL	9	0.1020	IQGAP1	ENSG00000140575.13	ENSP00000488618.1	132.1	3573
KYGIQMPAF	9	0.0474	IQGAP1	ENSG00000140575.13	ENSP00000488618.1	132.1	3573
RYPPDIRATF	10	0.0042	SBF1	ENSG00000100241.21	ENSP00000370196.2	56.3	5679
KYLSGIAHF	9	0.0016	PMPCA	ENSG00000165688.12	ENSP00000360782.3	51.8	1575
VYGSFASKL	9	0.0827	AZIN1	ENSG00000155096.14	ENSP00000337180.5	38.8	1344
TYLMLANHL	9	0.0856	GBE1	ENSG00000114480.13	ENSP00000410833.2	32.5	2106
EQPETYYKW	9	0.2333	INTS1	ENSG00000164880.16	ENSP00000385722.3	50.3	6570
KYEELFPAF	9	0.0153	NAPA	ENSG00000105402.8	ENSP00000263354.2	73.5	885
IYARAANMF	9	0.0084	NAPA	ENSG00000105402.8	ENSP00000263354.2	73.5	885
IYTDMGRFTI	10	0.2109	NAPA	ENSG00000105402.8	ENSP00000263354.2	73.5	885
AYLHTTTTF	9	0.0029	ABCC3	ENSG00000108846.16	ENSP00000285238.8	154.6	4581
HQALLHNKI	9	3.7262	ABCC3	ENSG00000108846.16	ENSP00000285238.8	154.6	4581
VYLHLRQTW	9	0.0164	SLC44A2	ENSG00000129353.15	ENSP00000385135.3	146.6	2112
EIKERIAKR	9	22.6842	BIN2	ENSG00000110934.11	ENSP00000445874.1	43.9	1617
KYGDPGSLF	9	0.0063	ITGA6	ENSG00000091409.15	ENSP00000406694.1	49.9	3390

STIGETSNR	9	18.2368	RBM23	ENSG00000100461.18	ENSP00000339220.5	49.5	1215
KYTKIFNDF	9	0.0077	ZMYND11	ENSG00000015171.19	ENSP00000487386.1	51.1	1755
VYVKDLSSF	9	0.0059	KIF3B	ENSG00000101350.8	ENSP00000364864.3	30.3	2241
EVFDERAANF	10	2.5226	VCL	ENSG00000035403.17	ENSP00000211998.4	34.7	3402
SVIDYQTHFR	10	13.6036	TMED7	ENSG00000134970.14	ENSP00000405926.3	30.7	672
SYLEKVVTL	9	0.0043	ABI3	ENSG00000108798.9	ENSP00000225941.1	41.4	1098
LLPGKTYSF	9	0.1344	FNDC3A	ENSG00000102531.16	ENSP00000381362.3	32.8	3426
EYLKLLHSF	9	0.0058	SREBF2	ENSG00000198911.12	ENSP00000354476.4	41.2	3423
YYQNYFEKL	9	0.0119	PATL1	ENSG00000166889.14	ENSP00000300146.9	31.9	2310
IYLSKPTHW	9	0.0061	POLR2B	ENSG00000047315.16	ENSP00000491706.1	55.0	3297
RYLLKFEQI	9	0.0315	POLR2B	ENSG00000047315.16	ENSP00000491706.1	55.0	3297
DVRDRMIHR	9	26.4000	ABLIM1	ENSG00000099204.20	ENSP00000376679.3	38.7	1365
KYMDIEFDF	9	0.0087	MYO1B	ENSG00000128641.19	ENSP00000376132.3	50.8	3408
KYLEDRGEVTF	11	0.0267	GRK2	ENSG00000173020.11	ENSP00000312262.5	100.0	2067
RYVVTSVSW	9	0.0274	EIF3B	ENSG00000106263.18	ENSP00000354125.4	128.9	2442
KYIQRQETI	9	0.0202	MX1	ENSG00000157601.14	ENSP00000381599.3	39.8	1986
AYAPPPHVI	9	0.0079	APOL2	ENSG00000128335.14	ENSP00000249066.6	38.2	1011
NYIKRNWRI	9	0.0872	CSE1L	ENSG00000124207.17	ENSP00000262982.2	32.1	2913
SYSGTFHSL	9	0.0117	NBPF1	ENSG00000219481.10	ENSP00000474456.1	50.3	3417
VWNPRSHEKL	10	0.5127	WDR6	ENSG00000178252.18	ENSP00000477389.1	60.2	3363
TYNRIINQI	9	0.0240	ANGPTL2	ENSG00000136859.10	ENSP00000362524.3	31.1	1479
KYFAKHPRL	9	0.0541	UTRN	ENSG00000152818.18	ENSP00000356515.3	43.0	10299
EIFDGNVAHI	10	7.8626	UTRN	ENSG00000152818.18	ENSP00000356515.3	43.0	10299
RYLGKVLEL	9	0.0161	CLUH	ENSG00000132361.17	ENSP00000464732.2	85.4	3753
KFHSEVAKF	9	0.0743	GGA2	ENSG00000103365.15	ENSP00000311962.4	47.0	1839
RYIHPQQEAF	10	0.0198	CPSF1	ENSG00000071894.17	ENSP00000484669.1	78.3	4329
EIMELDTSGF	10	9.7265	CPSF1	ENSG00000071894.17	ENSP00000484669.1	78.3	4329

IYTDQAGQWRI	11	0.4904	C12orf10	ENSG00000139637.14	ENSP00000267103.5	65.3	1128
QYLRNPPKL	9	0.0404	SHC1	ENSG00000160691.18	ENSP00000357438.4	81.5	1422
VYPDGIRHI	9	0.0220	SF3B3	ENSG00000189091.13	ENSP00000305790.5	46.1	3651
YYFPVKNVI	9	0.0190	SF3B3	ENSG00000189091.13	ENSP00000305790.5	46.1	3651
NYISGIQTI	9	0.0088	SF3B3	ENSG00000189091.13	ENSP00000305790.5	46.1	3651
EYIPDLYNHF	10	0.0109	RABGAP1	ENSG00000011454.17	ENSP00000362751.4	34.6	3207
RYQQWMERF	9	0.0050	ELAC2	ENSG00000006744.19	ENSP00000463740.2	50.2	1878
KFMPVSSLI	9	0.0906	FTSJ3	ENSG00000108592.17	ENSP00000396673.2	40.9	2541
HYAHLIKTF	9	0.0078	DOCK1	ENSG00000150760.12	ENSP00000280333.6	31.3	5595
MYIRYLYKL	9	0.0257	DOCK1	ENSG00000150760.12	ENSP00000280333.6	31.3	5595
KYDPNVYSI	9	0.0092	ITGAV	ENSG00000138448.12	ENSP00000261023.3	28.9	3144
AYLDALQTL	9	0.0105	KANSL3	ENSG00000114982.19	ENSP00000499674.1	35.4	2712
TYMKDLYQL	9	0.0083	ARHGAP18	ENSG00000146376.11	ENSP00000357131.2	27.5	1989
EQFKFKNMV	9	11.4239	FAM192A	ENSG00000172775.17	ENSP00000335808.6	51.2	762
RQALMPTL	8	7.1087	EP300	ENSG00000100393.13	ENSP00000263253.7	32.9	7242
EYVVRTFTL	9	0.0977	PTPRK	ENSG00000152894.14	ENSP00000357209.4	35.9	4320
QYNMNFEKL	9	0.0913	CASP7	ENSG00000165806.19	ENSP00000358324.3	36.0	909
TYDNVHQQF	9	0.0055	ARHGAP24	ENSG00000138639.18	ENSP00000378610.2	38.6	1959
VYSTPTPFF	9	0.0031	WAS	ENSG00000015285.10	ENSP00000365891.4	32.3	1506
KYQELQVLF	9	0.0015	VPS37B	ENSG00000139722.7	ENSP00000267202.2	27.9	855
MQQILTRV	8	18.4737	GNL2	ENSG00000134697.13	ENSP00000362153.3	33.4	2193
EYISKTYKI	9	0.0142	GNL2	ENSG00000134697.13	ENSP00000362153.3	33.4	2193
SEFRAFSSFKNR	12	46.0000	CD58	ENSG00000116815.16	ENSP00000358501.5	47.5	750
QYEEQFVTL	9	0.0796	XPO1	ENSG00000082898.16	ENSP00000385942.2	65.9	3213
VYIGKLNMI	9	0.0190	XPO1	ENSG00000082898.16	ENSP00000385942.2	65.9	3213
KYVHLFPKL	9	0.0203	SNRNP200	ENSG00000144028.15	ENSP00000498933.1	196.3	4125
ETQLPVSFR	9	24.0000	SNRNP200	ENSG00000144028.15	ENSP00000498933.1	196.3	4125

FYLPKDAVKHL	11	0.3200	RASSF1	ENSG00000068028.17	ENSP00000333327.3	47.2	810
YYITTRVQF	9	0.0072	FGR	ENSG00000000938.13	ENSP00000363115.3	46.1	1587
NYAWVYYHL	9	0.0406	IFIT3	ENSG00000119917.14	ENSP00000360876.4	43.0	1470
IYKDYQYYF	9	0.0028	MRC1	ENSG00000260314.3	ENSP00000455897.1	26.1	4368
RYPDSHQLF	9	0.0020	G3BP2	ENSG00000138757.14	ENSP00000379069.3	36.3	1446
VYPEKLATKF	10	0.0074	ECPAS	ENSG00000136813.14	ENSP00000339889.5	39.6	5517
RWMSQHNRF	9	0.0504	PAFAH1B2	ENSG00000168092.14	ENSP00000435289.1	27.8	687
AQLAVTKI	8	17.6000	UBR7	ENSG00000012963.15	ENSP00000013070.6	27.2	1275
VYANGIRNI	9	0.0653	MARCH6	ENSG00000145495.16	ENSP00000274140.4	47.1	2730
TYKQVVDLI	9	0.0532	CYTIP	ENSG00000115165.10	ENSP00000264192.3	26.4	1077
KYHGYPYSF	9	0.0019	MAGT1	ENSG00000102158.19	ENSP00000478379.1	25.7	1101
RYSSAFTNRIFF	12	0.1923	MAGT1	ENSG00000102158.19	ENSP00000478379.1	25.7	1101
KYGFGYQL	8	0.6496	PLK2	ENSG00000145632.15	ENSP00000274289.3	101.4	2055
QYSNAFDVF	9	0.0399	TOB1	ENSG00000141232.5	ENSP00000268957.3	37.5	1035
RYLPDTLLL	9	0.0071	COMT	ENSG00000093010.13	ENSP00000354511.6	62.9	813
EQYYVRKV	8	22.1053	BICD2	ENSG00000185963.14	ENSP00000364662.3	31.9	2472
HYMSINDSF	9	0.0118	BICD2	ENSG00000185963.14	ENSP00000364662.3	31.9	2472
RYFPDRNVALF	11	0.0098	INPP5E	ENSG00000148384.13	ENSP00000360777.3	26.5	1932
IYSTPLPEKF	10	0.0051	KLF3	ENSG00000109787.13	ENSP00000261438.5	27.8	1035
QQWPVRSI	8	8.4347	XPO6	ENSG00000169180.11	ENSP00000302790.4	54.1	3375
YYMKDLPTSF	10	0.0052	PI4KA	ENSG00000241973.10	ENSP00000255882.6	60.5	6306
SYMPTVSHL	9	0.0043	BSCL2	ENSG00000168000.14	ENSP00000413209.1	61.8	1194
AYLRIHAHF	9	0.0068	BSCL2	ENSG00000168000.14	ENSP00000413209.1	61.8	1194
WQAKVPQI	8	6.7285	DYNC1H1	ENSG00000197102.12	ENSP00000348965.4	69.9	13938
RYKLYQEMF	9	0.0326	DYNC1H1	ENSG00000197102.12	ENSP00000348965.4	69.9	13938
ETFDFQAMGR	10	23.7500	DYNC1H1	ENSG00000197102.12	ENSP00000348965.4	69.9	13938
RYYGNISRF	9	0.0041	EHMT2	ENSG00000204371.11	ENSP00000364687.4	49.1	3630

KYAHMINGF	9	0.0156	ADSS	ENSG00000035687.10	ENSP00000355493.3	27.0	1368
EVINRSMDTY	10	26.1000	CAMLG	ENSG00000164615.5	ENSP00000297156.2	32.3	888
EVISLINTR	9	18.1579	FAF2	ENSG00000113194.13	ENSP00000261942.6	26.1	1335
KYSLIKGNF	9	0.0796	S100A8	ENSG00000143546.10	ENSP00000357722.3	26.9	279
LYQHEINLF	9	0.0061	CEBPZ	ENSG00000115816.15	ENSP00000234170.5	26.5	3162
VYADQPHIF	9	0.0016	ABHD4	ENSG00000100439.10	ENSP00000414558.2	26.6	1026
EQIDKKLER	9	15.9275	PTPN12	ENSG00000127947.16	ENSP00000248594.6	65.8	2340
YSAPATLSSR	10	24.7143	PDLIM2	ENSG00000120913.23	ENSP00000477546.1	78.8	438
RYGDGGSTF	9	0.0128	HNRNPH1	ENSG00000169045.17	ENSP00000426275.1	311.2	1287
EVIVTEVGQR	10	60.0000	AKAP12	ENSG00000131016.17	ENSP00000253332.1	43.4	5346
SVVEIASLR	9	17.9333	MCRS1	ENSG00000187778.14	ENSP00000349640.4	62.6	1425
LYDPVISKL	9	0.0417	MCRS1	ENSG00000187778.14	ENSP00000349640.4	62.6	1425
ETILRTNKR	9	28.3750	SH3KBP1	ENSG00000147010.18	ENSP00000369020.4	36.4	1884
ELIQEITQR	9	20.1111	RDX	ENSG00000137710.16	ENSP00000496503.1	41.5	1749
KYNLVTHIL	9	0.0219	ZNF710	ENSG00000140548.10	ENSP00000268154.3	28.9	1992
LYPDHFHLL	9	0.0048	PPP5C	ENSG00000011485.14	ENSP00000012443.4	52.4	1497
KYLTSLQLVQF	11	0.0452	ELOVL7	ENSG00000164181.14	ENSP00000424123.1	31.2	843
LYTEKFEEF	9	0.0048	TXLNA	ENSG00000084652.16	ENSP00000362712.3	35.0	1638
YYLTHGLYL	9	0.0587	PIGU	ENSG00000101464.10	ENSP00000217446.3	25.1	1305
AYNAVVRYF	9	0.0167	FMNL3	ENSG00000161791.14	ENSP00000335655.5	44.6	3081
MDSSLTRR	8	58.3333	FDPS	ENSG00000160752.14	ENSP00000391755.1	128.1	1059
KQPEDYFYL	9	0.3808	MYO9B	ENSG00000099331.13	ENSP00000471367.1	82.2	6471
KYMPNVKVAVF	11	0.0325	DDX39B	ENSG00000198563.14	ENSP00000399371.1	316.8	867
HVPGSPFTAR	10	16.0364	FLNA	ENSG00000196924.17	ENSP00000358866.3	509.3	7941
RFIPRENGVYL	11	0.6566	FLNA	ENSG00000196924.17	ENSP00000358866.3	509.3	7941
VFIDKQTNL	9	0.2593	CELF1	ENSG00000149187.18	ENSP00000378705.2	38.3	1455
RYMILGQNGF	10	0.0911	PGM5	ENSG00000154330.13	ENSP00000379678.1	24.0	1701

KYGVHEAIF	9	0.0971	PPARD	ENSG00000112033.14	ENSP00000310928.4	35.9	1323
KYFLWEEKF	9	0.0073	GRAP	ENSG00000154016.13	ENSP00000284154.5	25.3	651
NYGDQVQHF	9	0.0077	GRAP	ENSG00000154016.13	ENSP00000284154.5	25.3	651
RYSDSTFTF	9	0.0014	PARP8	ENSG00000151883.18	ENSP00000422217.2	32.1	2562
NYTKAMRLF	9	0.0481	ADI1	ENSG00000182551.14	ENSP00000333666.3	39.0	537
TWTEVSYTF	9	0.0087	FBXO6	ENSG00000116663.11	ENSP00000365944.4	23.4	879
RYLEAGAAGLRW	12	0.2881	HSPBP1	ENSG00000133265.11	ENSP00000467574.1	35.5	1077
SYNEHWNYL	9	0.0269	AC005840.1	ENSG00000111321.11	ENSP00000228918.4	113.3	1305
SVISGISSR	9	11.0000	STAG2	ENSG00000101972.18	ENSP00000360186.3	41.4	3693
IYLDTHFRL	9	0.0074	ZNFX1	ENSG00000124201.15	ENSP00000379412.1	28.4	5754
LAHQSWALLH	10	27.3000	FUT11	ENSG00000196968.11	ENSP00000361932.3	83.4	1476
EYGVIRDVL	9	0.8919	H6PD	ENSG00000049239.12	ENSP00000366620.1	24.1	2373
STAPFGLKPR	10	27.9000	LRCH1	ENSG00000136141.15	ENSP00000308493.5	24.2	2088
EYGIFNQKI	9	0.2321	PYGL	ENSG00000100504.17	ENSP00000443787.1	58.6	2439
SYAPPTDSF	9	0.0041	SGK1	ENSG00000118515.11	ENSP00000396242.3	71.9	1335
TYDKGYQF	8	0.1860	PEX5	ENSG00000139197.10	ENSP00000266564.3	45.8	1893
EVIDLMIKEY	10	25.6667	PHF10	ENSG00000130024.15	ENSP00000355743.4	31.1	1488
VYTVHHVVW	9	0.0522	MAST3	ENSG00000099308.10	ENSP00000262811.4	23.8	3927
EVVEDSLRQR	10	41.5000	TMX4	ENSG00000125827.9	ENSP00000246024.2	22.9	1047
VYTTNIQEL	9	0.0248	LPXN	ENSG00000110031.12	ENSP00000378512.2	31.2	1158
RYVPRASYF	9	0.0061	CCND3	ENSG00000112576.12	ENSP00000397545.2	41.1	660
TYLPTSPLL	9	0.0084	NRDC	ENSG00000078618.21	ENSP00000262679.8	82.1	3453
RWFTHASPTL	10	0.5035	RRM1	ENSG00000167325.15	ENSP00000300738.5	28.4	2376
QYTHKIYHL	9	0.0221	PITPNA	ENSG00000174238.14	ENSP00000316809.7	45.1	810
RYVMTTTTL	9	0.0529	EIF2S1	ENSG00000134001.13	ENSP00000256383.4	36.5	945
RYKAPFHQL	9	0.0113	SMARCA5	ENSG00000153147.6	ENSP00000283131.3	22.8	3156
KYGMVTYLL	9	0.0229	GPAT4	ENSG00000158669.11	ENSP00000380184.3	54.1	1368

HYTIVFNTF	9	0.0053	ATP2B4	ENSG00000058668.14	ENSP00000340930.2	27.7	3510
IYQYIQSRF	9	0.0057	DYRK1A	ENSG00000157540.21	ENSP00000495571.1	34.4	2175
ETVNLRSLGF	10	10.4237	CRYBG1	ENSG00000112297.15	ENSP00000358062.3	22.8	5169
RMPNSPAPHF	10	0.2059	CRYBG1	ENSG00000112297.15	ENSP00000358062.3	22.8	5169
VYLPNINKI	9	0.0031	RHOBTB1	ENSG00000072422.17	ENSP00000350595.4	31.4	2088
RYTIVQQQI	9	0.0257	TUBGCP2	ENSG00000130640.13	ENSP00000252936.3	64.8	2706
SQRPIQMV	8	18.9211	EHD3	ENSG00000013016.16	ENSP00000327116.5	21.8	1605
YLNHWNHF	8	0.5572	FN3KRP	ENSG00000141560.15	ENSP00000269373.6	36.7	927
HYLNHWNHF	9	0.0057	FN3KRP	ENSG00000141560.15	ENSP00000269373.6	36.7	927
LYIDEAHSI	9	0.0215	SPTLC2	ENSG00000100596.6	ENSP00000216484.2	22.7	1686
LYMRIRDNW	9	0.0883	SPTLC2	ENSG00000100596.6	ENSP00000216484.2	22.7	1686
SYVQVTSNF	9	0.0048	AFF4	ENSG00000072364.13	ENSP00000265343.5	31.9	3489
SYSSTFHSL	9	0.0086	NBPF15	ENSG00000266338.6	ENSP00000463178.1	36.8	2010
VYTTMAEHF	9	0.0049	TSPAN1	ENSG00000117472.10	ENSP00000361072.1	46.3	723
RYPPKSGNYF	10	0.0139	MGRN1	ENSG00000102858.13	ENSP00000262370.6	51.0	1728
HQLPLPHNV	9	1.7648	HIF1A	ENSG00000100644.17	ENSP00000323326.6	57.0	2205
VYTVVSSHEQF	11	0.0653	ITGAX	ENSG00000140678.16	ENSP00000268296.4	62.4	3489
SYLSHSEQLVF	11	0.0506	TMEM106A	ENSG00000184988.8	ENSP00000483246.1	25.8	786
PYHPHPHVF	9	0.0090	FOXN3	ENSG00000053254.15	ENSP00000343288.4	28.3	1470
YYIRGATTTF	10	0.0094	DPT	ENSG00000143196.5	ENSP00000356791.3	21.3	603
RYLPTGSFPF	10	0.0172	TUSC3	ENSG00000104723.20	ENSP00000424544.1	26.5	1044
VYKENLVDGF	10	0.1036	NELFE	ENSG00000204356.14	ENSP00000364574.5	87.1	1161
EYNTVASKF	9	0.0450	PARP14	ENSG00000173193.15	ENSP00000420649.1	46.2	4563
VYVGAVNRI	9	0.0502	PLXNA1	ENSG00000114554.11	ENSP00000377061.2	23.3	5688
RYLPKGFLNQF	11	0.0095	PTK2	ENSG00000169398.19	ENSP00000341189.3	92.3	3195
HGMIMLRI	8	23.6875	ARCN1	ENSG00000095139.14	ENSP00000264028.4	27.8	1533
EYMEHTYLI	9	0.0138	OSBPL1A	ENSG00000141447.18	ENSP00000382372.3	28.2	1311

RYSVFFQSL	9	0.0221	PSMD6	ENSG00000163636.10	ENSP00000295901.4	33.3	1167
VGARIYHTI	9	1.7211	MGST1	ENSG00000008394.13	ENSP00000379513.3	98.3	465
AYIERMNYI	9	0.0535	FYN	ENSG00000010810.17	ENSP00000357667.4	55.2	1602
YYITTRAQF	9	0.0132	FYN	ENSG00000010810.17	ENSP00000357667.4	55.2	1602
DIPIHNALR	9	31.8333	GUCY1A1	ENSG00000164116.16	ENSP00000377418.3	35.3	1296
KFSNVTMLF	9	0.0142	GUCY1A1	ENSG00000164116.16	ENSP00000377418.3	35.3	1296
LYSSKLYRF	9	0.0036	KCTD20	ENSG00000112078.14	ENSP00000412205.2	34.5	759
SYMLVNENRF	10	0.0500	LDOC1	ENSG00000182195.8	ENSP00000359557.2	24.8	438
LYGKIAEAF	9	0.0168	GIPC1	ENSG00000123159.16	ENSP00000376753.3	77.8	999
VYAAQPHVI	9	0.0201	FAM168A	ENSG00000054965.10	ENSP00000064778.4	23.4	732
KYAMMFAEL	9	0.1095	HUWE1	ENSG00000086758.16	ENSP00000479451.1	60.9	13095
GYASRFIVI	9	0.2298	RPIA	ENSG00000153574.9	ENSP00000283646.3	20.5	933
IQRRIMESV	9	9.6147	SKIV2L	ENSG00000204351.12	ENSP00000364543.2	65.6	3738
SFSPKTYSF	9	0.0057	PIK3C2A	ENSG00000011405.13	ENSP00000265970.6	22.9	5058
RYMQWRETM	9	0.0438	ITPKC	ENSG00000086544.3	ENSP00000263370.1	20.9	2049
VYITRAQLM	9	0.0807	NACC1	ENSG00000160877.6	ENSP00000292431.3	25.0	1581
GYIERPQLI	9	0.0382	TENT2	ENSG00000164329.13	ENSP00000421966.1	39.4	1323
KYPVLVERI	9	0.0235	ARHGEF28	ENSG00000214944.9	ENSP00000296799.4	35.3	4176
FTMGQVVSR	9	13.4955	TNFSF13	ENSG00000161955.16	ENSP00000379794.4	38.2	741
RYPLLLQKI	9	0.0121	ARHGEF37	ENSG00000183111.12	ENSP00000328083.6	20.9	2025
RYKEKVAEL	9	0.0759	KAT7	ENSG00000136504.12	ENSP00000409477.2	28.6	1275
TYLDKVENI	9	0.0127	IFIT1	ENSG00000185745.10	ENSP00000360869.3	20.3	1434
GQFKIGLI	8	21.0000	VPS29	ENSG00000111237.18	ENSP00000447058.1	66.4	546
AYSHLRYVF	9	0.0231	FAT1	ENSG00000083857.14	ENSP00000406229.2	68.0	13764
VYVQNVVKL	9	0.0130	AP3D1	ENSG00000065000.18	ENSP00000495274.1	58.2	3645
NYLNHWNHF	9	0.0085	FN3K	ENSG00000167363.14	ENSP00000300784.7	31.5	927
RYLVEVEEL	9	0.0556	PDRG1	ENSG00000088356.6	ENSP00000202017.4	20.0	399

YLGLTNELF	9	0.9426	PNPLA6	ENSG00000032444.16	ENSP00000473211.1	68.3	4095
SQFELLKV	8	14.7222	RPS6KA2	ENSG00000071242.12	ENSP00000422435.1	22.6	2274
QYFPKAPEF	9	0.0029	USP48	ENSG00000090686.15	ENSP00000363864.3	49.3	1563
DYKTVNNLL	9	0.3204	USP48	ENSG00000090686.15	ENSP00000363864.3	49.3	1563
YYTPITPHL	9	0.0047	PTBP3	ENSG00000119314.15	ENSP00000363375.1	22.8	1572
KYTTLIAKL	9	0.0439	TES	ENSG00000135269.18	ENSP00000350937.4	27.4	1263
SYLKQLPHF	9	0.0023	SNRNP200	ENSG00000144028.15	ENSP00000317123.5	196.3	6408
VYMDWYEKF	9	0.0007	SNRNP200	ENSG00000144028.15	ENSP00000317123.5	196.3	6408
TYKVISNEF	9	0.0172	UBE3A	ENSG00000114062.20	ENSP00000486349.1	28.6	2556
EVIKNFIQY	9	5.4624	EIF3L	ENSG00000100129.18	ENSP00000499067.1	258.9	1692
RYQDIIHSI	9	0.0020	BAZ1B	ENSG00000009954.11	ENSP00000342434.4	31.8	4449
LYMVNGPPHF	10	0.0245	EIF2A	ENSG00000144895.12	ENSP00000417229.1	45.6	1755
YYSPHGHILVL	11	0.1875	EIF2A	ENSG00000144895.12	ENSP00000417229.1	45.6	1755
YLKTVDKF	8	0.5929	TRAPPC2B	ENSG00000256060.2	ENSP00000442778.1	20.9	420
MYLKTVDKF	9	0.0039	TRAPPC2B	ENSG00000256060.2	ENSP00000442778.1	20.9	420
TYQVLAVTF	9	0.0055	SNRNP40	ENSG00000060688.13	ENSP00000263694.4	26.1	1071
VYIEKNDKL	9	0.0512	ERBB3	ENSG00000065361.15	ENSP00000448483.1	96.3	2763
HYGKVYLL	8	0.3747	VPS41	ENSG00000006715.16	ENSP00000309457.4	25.8	2562
RYVEIVSQF	9	0.0015	VPS41	ENSG00000006715.16	ENSP00000309457.4	25.8	2562
PYPPPPPEF	9	0.0069	WBP2	ENSG00000132471.12	ENSP00000466999.1	89.8	693
EYQKVGQSF	9	0.0225	SNX18	ENSG00000178996.14	ENSP00000370817.4	19.6	1872
VYQEMPAQL	9	0.0119	BCKDHA	ENSG00000248098.12	ENSP00000416000.2	66.7	1344
NYIKSLSSF	9	0.0075	SMG1	ENSG00000157106.16	ENSP00000402515.2	27.1	10983
RYDNVTILF	9	0.0074	GUCY1B1	ENSG00000061918.13	ENSP00000425065.1	40.2	1653
EVVTGVIGQR	10	45.5000	GUCY1B1	ENSG00000061918.13	ENSP00000425065.1	40.2	1653
VYDITRRETF	10	0.1019	RAB2B	ENSG00000129472.15	ENSP00000380869.1	28.2	648
DPASLPRVLRV	11	28.1250	THEMIS2	ENSG00000130775.16	ENSP00000363031.3	49.3	1929

EYLIKVNEI	9	0.1539	ASAP2	ENSG00000151693.11	ENSP00000281419.3	27.8	3018
QYTQALEKF	9	0.0092	ASAP2	ENSG00000151693.11	ENSP00000281419.3	27.8	3018
EIISEVQRM	9	8.8803	ASAP2	ENSG00000151693.11	ENSP00000281419.3	27.8	3018
PYNPLWAQL	9	0.0583	FNIP2	ENSG00000052795.13	ENSP00000264433.6	28.8	3342
AQRAFILTV	9	6.4542	CABIN1	ENSG00000099991.18	ENSP00000263119.5	52.5	6660
VYAQVARLF	9	0.0034	SIN3A	ENSG00000169375.15	ENSP00000378403.4	28.1	3819
EVIHRKALQR	10	46.0000	SIN3A	ENSG00000169375.15	ENSP00000378403.4	28.1	3819
AQHKFLVAV	9	8.6933	TCF25	ENSG00000141002.20	ENSP00000457329.1	192.2	795
TYMPHVHIL	9	0.0026	FBXO38	ENSG00000145868.16	ENSP00000342023.5	24.8	3564
NYLSHHLTI	9	0.0216	PAIP1	ENSG00000172239.14	ENSP00000425675.1	49.6	600
KYVDKLEKI	9	0.0196	USP5	ENSG00000111667.13	ENSP00000373883.5	43.7	2505
FVIDAVRTK	9	21.2273	EIF3M	ENSG00000149100.13	ENSP00000436049.1	37.9	1122
VWNPRTHQF	9	0.0075	ATP1B2	ENSG00000129244.9	ENSP00000250111.4	18.8	870
KYLGQLHYL	9	0.0108	PTPN23	ENSG00000076201.15	ENSP00000265562.4	20.6	4908
YQIAVTKV	8	8.7479	SMC1A	ENSG00000072501.17	ENSP00000323421.3	42.7	3699
QQSVLQRI	8	15.8406	SMC1A	ENSG00000072501.17	ENSP00000323421.3	42.7	3699
KYPNVFKKI	9	0.0209	AHCYL1	ENSG00000168710.18	ENSP00000377238.4	45.2	1449
RYLLFARQF	9	0.0237	MCM6	ENSG00000076003.5	ENSP00000264156.2	18.6	2463
KYIQKPGLL	9	0.0906	PAQR7	ENSG00000182749.5	ENSP00000363414.3	18.4	1038
KYFSDGSSNTF	11	0.0197	BTBD3	ENSG00000132640.15	ENSP00000483520.1	22.7	1383
SYLNTVERW	9	0.0099	KLHL5	ENSG00000109790.16	ENSP00000425512.1	39.8	846
LYPPRQPTF	9	0.0027	SIGLEC1	ENSG00000088827.12	ENSP00000341141.4	25.1	5127
SYAPRHVTL	9	0.0112	SIGLEC1	ENSG00000088827.12	ENSP00000341141.4	25.1	5127
NQTPLPLI	8	9.3325	TAF9	ENSG00000273841.5	ENSP00000370193.1	33.1	792
IYGPIESTI	9	0.0192	CPA3	ENSG00000163751.4	ENSP00000296046.3	18.2	1251
RQPSLFYHL	9	0.2162	MAP3K5	ENSG00000197442.10	ENSP00000351908.4	18.4	4122
VYIPMSPGAHHF	12	0.0076	GAB2	ENSG00000033327.13	ENSP00000354952.4	18.7	2028

EYVKQTWNL	9	0.0598	SYK	ENSG00000165025.15	ENSP00000364898.1	25.8	1905
KYMKLRDFF	9	0.0160	GLB1L2	ENSG00000149328.14	ENSP00000344659.6	24.8	1908
RYPNSHTHYF	10	0.0083	CNOT1	ENSG00000125107.18	ENSP00000320949.5	58.7	7128
RYFDHALTL	9	0.0072	FAM91A1	ENSG00000176853.16	ENSP00000429491.1	30.9	2367
TYLKAVKLF	9	0.0042	IREB2	ENSG00000136381.13	ENSP00000258886.8	20.8	2889
EVAQFLTGR	9	20.5556	DESI1	ENSG00000100418.8	ENSP00000263256.6	21.2	504
RYLNSQQQYF	10	0.0203	NFRKB	ENSG00000170322.14	ENSP00000400476.2	21.9	3897
KYITKSFNF	9	0.0024	PARN	ENSG00000140694.17	ENSP00000498650.1	28.2	1743
KYITKSFNFY	10	0.5365	PARN	ENSG00000140694.17	ENSP00000498650.1	28.2	1743
FTAPSTVGKR	10	27.5000	PARN	ENSG00000140694.17	ENSP00000498650.1	28.2	1743
VFTPVVQRI	9	0.0740	CMIP	ENSG00000153815.16	ENSP00000478272.1	37.3	1758
KYVENFGLI	9	0.1207	SPCS2	ENSG00000118363.12	ENSP00000263672.6	38.6	678
VYVAALKTL	9	0.0519	MAP3K1	ENSG00000095015.6	ENSP00000382423.3	20.0	4536
ESYQMGHMRR	10	16.4727	ADAM10	ENSG00000137845.15	ENSP00000260408.3	23.1	2244
SVIDGRFEGF	10	2.2240	ADAM10	ENSG00000137845.15	ENSP00000260408.3	23.1	2244
AYLITLGKF	9	0.0754	CYFIP1	ENSG00000273749.5	ENSP00000481038.1	76.5	3759
RYVFQSENTF	10	0.0357	SUFU	ENSG00000107882.11	ENSP00000358918.3	19.1	1452
IYIINVHSM	9	0.0272	DENND3	ENSG00000105339.10	ENSP00000410594.1	80.5	3438
SQALILKI	8	9.8147	NUP188	ENSG00000095319.14	ENSP00000361658.2	26.2	5247
YYISPRLTF	9	0.0026	SLA	ENSG00000155926.14	ENSP00000378759.3	41.1	879
LYLLNTTKL	9	0.0714	MLH1	ENSG00000076242.14	ENSP00000398272.2	24.5	1545
AYRRIFQKF	9	0.0296	MYO1E	ENSG00000157483.8	ENSP00000288235.4	23.4	3324
RYAILTKATW	10	0.1595	MYO1E	ENSG00000157483.8	ENSP00000288235.4	23.4	3324
RYITKGNLF	9	0.0081	PPP4R3B	ENSG00000275052.5	ENSP00000484116.1	33.7	2451
DVAQWNIGSLR	11	44.0000	KDM4B	ENSG00000127663.15	ENSP00000440495.1	30.2	3390
IYIDRFEDL	9	0.0541	ARL14EP	ENSG00000152219.5	ENSP00000282032.3	20.5	780
TYLIQHQKI	9	0.0253	ZNF189	ENSG00000136870.10	ENSP00000363995.3	26.5	1836

IYQKAFDLI	9	0.0330	KPNA6	ENSG00000025800.14	ENSP00000362728.3	18.4	1608
VFIGTGHLL	9	0.1314	LRRK2	ENSG00000188906.16	ENSP00000341930.2	56.5	3813
SYLSRLQYF	9	0.0028	IQGAP2	ENSG00000145703.16	ENSP00000274364.6	19.3	4725
KYQDILNEI	9	0.0182	IQGAP2	ENSG00000145703.16	ENSP00000274364.6	19.3	4725
TYPEVKNKL	9	0.0417	IQGAP2	ENSG00000145703.16	ENSP00000274364.6	19.3	4725
EVFPEHLARF	10	0.4834	LATS2	ENSG00000150457.9	ENSP00000372035.4	17.4	3264
QQFKILNV	8	15.6812	UPF2	ENSG00000151461.20	ENSP00000380244.2	18.5	3816
RYPSLWRRL	9	0.0443	SMARCB1	ENSG00000099956.19	ENSP00000383984.3	48.1	1128
TYIPVPAKI	9	0.0036	ST3GAL1	ENSG00000008513.16	ENSP00000430515.1	26.3	1020
VYPESFREL	9	0.0197	ST3GAL1	ENSG00000008513.16	ENSP00000430515.1	26.3	1020
EVVGIIESR	9	24.0000	NUP160	ENSG00000030066.13	ENSP00000367721.2	28.4	4308
VYAILTHGI	9	0.0592	PRPS1	ENSG00000147224.11	ENSP00000496286.1	32.1	963
VYVKHSISF	9	0.0038	AP3M1	ENSG00000185009.12	ENSP00000347408.4	20.5	1254
EYIRSLNQF	9	0.0080	RMC1	ENSG00000141452.9	ENSP00000467007.1	31.4	1827
RYQRLYVKF	9	0.0045	PRR12	ENSG00000126464.14	ENSP00000394510.1	19.7	6108
TAEQIRLAQM	10	27.1000	UBAP2L	ENSG00000143569.19	ENSP00000271877.8	88.7	3237
IITPIHEQW	9	0.5891	PSMF1	ENSG00000125818.18	ENSP00000246015.4	37.6	789
KYVERIHYI	9	0.0052	HMOX2	ENSG00000103415.12	ENSP00000394103.3	46.0	948
WVIGSVVAR	9	32.6000	GART	ENSG00000159131.17	ENSP00000371236.4	31.2	3030
VYIHPSSALF	10	0.0059	DHX8	ENSG00000067596.11	ENSP00000262415.2	26.5	3660
LYDIVFKHF	9	0.0202	SP100	ENSG00000067066.17	ENSP00000386427.1	95.2	2064
RYLSKATTL	9	0.0202	CDC16	ENSG00000130177.16	ENSP00000252458.6	103.0	1425
YSHVIQKL	8	1.0537	SDS	ENSG00000135094.11	ENSP00000257549.4	21.2	984
VYSHVIQKL	9	0.0033	SDS	ENSG00000135094.11	ENSP00000257549.4	21.2	984
VYSHVIQKLQL	11	0.2220	SDS	ENSG00000135094.11	ENSP00000257549.4	21.2	984
RQHEIVLKV	9	2.7243	NUP133	ENSG00000069248.12	ENSP00000261396.3	18.0	3468
IYQDSFEQRF	10	0.0057	CUL4B	ENSG00000158290.16	ENSP00000360373.5	32.4	2685

VGYLVRLV	8	26.9000	FZD8	ENSG00000177283.7	ENSP00000363826.1	16.1	2082
RYVEQSNLM	9	0.1373	FKBP15	ENSG00000119321.9	ENSP00000238256.3	23.3	3657
YYAEVETRI	9	0.0558	EXOC2	ENSG00000112685.14	ENSP00000230449.4	17.0	2772
PYNPILGETF	10	0.0268	OSBPL8	ENSG00000091039.17	ENSP00000261183.3	22.5	2667
ESVGGRVAER	10	53.7500	LIMA1	ENSG00000050405.13	ENSP00000448779.1	44.4	1800
KQQKLWHLF	9	0.1720	C16orf72	ENSG00000182831.12	ENSP00000331720.7	24.7	825
DVITDSPTHKR	11	28.7500	C16orf72	ENSG00000182831.12	ENSP00000331720.7	24.7	825
DLIGQDLNSR	10	42.5000	FBRS	ENSG00000156860.15	ENSP00000348489.5	34.9	2940
VYVKHNISF	9	0.0059	AP3M2	ENSG00000070718.12	ENSP00000428787.1	20.4	1254
QQRLVSELI	9	5.8896	ARHGEF40	ENSG00000165801.10	ENSP00000298694.4	52.3	4557
DYGEINQLL	9	0.2159	SESN1	ENSG00000080546.13	ENSP00000349061.7	33.2	1476
KYSEVFEAI	9	0.0179	CSNK2A1	ENSG00000101266.19	ENSP00000493931.1	39.6	1062
RYFKGPELL	9	0.0100	CSNK2A1	ENSG00000101266.19	ENSP00000493931.1	39.6	1062
EVFDHMMKR	9	13.0631	USP9X	ENSG00000124486.13	ENSP00000367558.2	26.1	7662
EVFDHMMKRM	10	14.7556	USP9X	ENSG00000124486.13	ENSP00000367558.2	26.1	7662
AYSAKIALF	9	0.0062	DPYD	ENSG00000188641.13	ENSP00000359211.3	16.9	3075
IYAPPLPSL	9	0.0054	AKAP1	ENSG00000121057.13	ENSP00000337736.3	24.6	2709
EVIEQIENL	9	5.2494	NKTR	ENSG00000114857.18	ENSP00000232978.8	43.2	4386
TYQNDIALI	9	0.1291	CFI	ENSG00000205403.13	ENSP00000378131.3	116.8	1773
GQWEVKKI	8	18.6053	SEC13	ENSG00000157020.18	ENSP00000312122.4	55.0	966
RYFKTPRKF	9	0.0066	CIZ1	ENSG00000148337.21	ENSP00000398011.1	93.0	2460
EVIDFSHGL	9	5.1162	ABHD17A	ENSG00000129968.15	ENSP00000292577.6	56.8	930
PYTKVEESF	9	0.0412	ALG12	ENSG00000182858.14	ENSP00000333813.5	18.5	1464
IYNKVTPTF	9	0.0011	SPRED1	ENSG00000166068.13	ENSP00000299084.4	17.5	1332
IWISKLPHF	9	0.0086	TDRD7	ENSG00000196116.8	ENSP00000347444.4	16.9	3294
YYQSSVQYL	9	0.0083	DNAAF5	ENSG00000164818.16	ENSP00000384884.3	28.0	840
EQYRQVISV	9	8.4538	VPS11	ENSG00000160695.14	ENSP00000481126.1	58.0	2823

SVIEAVAHF	9	0.4722	UBR2	ENSG00000024048.10	ENSP00000361992.1	18.4	5265
RYTALQAFKF	10	0.0827	UBR2	ENSG00000024048.10	ENSP00000361992.1	18.4	5265
EVYEGVYTNH	10	21.7273	PTK2B	ENSG00000120899.18	ENSP00000391995.2	32.3	2901
TYIKSPPFF	9	0.0020	ACO1	ENSG00000122729.19	ENSP00000309477.5	18.4	2667
SYSMIVNNL	9	0.0581	CACYBP	ENSG00000116161.17	ENSP00000356652.2	21.3	684
SQLQALHI	8	12.2778	ROCK2	ENSG00000134318.14	ENSP00000317985.6	16.3	4164
SFKQRSDLF	9	0.2681	ZNF394	ENSG00000160908.15	ENSP00000337363.6	21.6	1683
RYSDGLLRF	9	0.0053	LZTS1	ENSG00000061337.15	ENSP00000265801.6	26.8	1788
ETVEKFRQR	9	16.7636	COMMD10	ENSG00000145781.9	ENSP00000488332.1	21.9	564
RFLNDPGHLLW	11	0.1454	RPP21	ENSG00000241370.5	ENSP00000397778.2	35.1	432
RYVIIPTTF	9	0.0029	CAPN5	ENSG00000149260.18	ENSP00000498132.1	19.1	1920
QYQQIISRL	9	0.0279	PPP1R13L	ENSG00000104881.16	ENSP00000354218.4	61.2	2484
RYNPITKRI	9	0.0169	AOX1	ENSG00000138356.14	ENSP00000363832.2	16.2	4014
VYPPIRHHL	9	0.0075	VPS37A	ENSG00000155975.10	ENSP00000318629.4	18.9	1191
QYSDVNNRW	9	0.0627	SPTBN1	ENSG00000115306.16	ENSP00000349259.4	262.0	7092
SYTSVKENF	9	0.0066	FAM120B	ENSG00000112584.13	ENSP00000417970.1	18.1	2730
AYFKDFHLL	9	0.0062	SLC13A1	ENSG00000081800.9	ENSP00000194130.2	15.9	1785
SVVGFLSQR	9	13.2613	DCAF5	ENSG00000139990.17	ENSP00000451845.1	41.8	2823
VYQHLFTRI	9	0.0176	AP5Z1	ENSG00000242802.9	ENSP00000497395.1	34.0	1464
KYVYVVTEL	9	0.0604	RPS6KA3	ENSG00000177189.14	ENSP00000368884.3	16.4	2220
AYTPFHAVL	9	0.0646	INTS14	ENSG00000138614.15	ENSP00000326379.2	22.8	1554
SYLENVDHF	9	0.0037	CAMTA2	ENSG00000108509.21	ENSP00000354828.5	45.3	3603
EAIGIISKM	9	7.1005	NCAPD2	ENSG00000010292.13	ENSP00000325017.5	25.6	4203
DLGGLEALRQR	11	45.0000	FLYWCH1	ENSG00000059122.16	ENSP00000344122.5	59.4	1482
ALPSKLPTF	9	0.0710	TRIM25	ENSG00000121060.18	ENSP00000323889.4	29.4	1890
KYHTHLLQF	9	0.0065	ABCD1	ENSG00000101986.12	ENSP00000218104.3	15.3	2235
TYDRVESLF	9	0.0432	EXT2	ENSG00000151348.14	ENSP00000342656.3	22.8	2154

FYHKYFNYL	9	0.0550	EXT2	ENSG00000151348.14	ENSP00000342656.3	22.8	2154
RYGLVTNEI	9	0.1078	IFIH1	ENSG00000115267.8	ENSP00000497271.1	22.1	3075
ILGPPPPSF	9	0.1364	MATR3	ENSG00000015479.18	ENSP00000426030.1	68.8	1527
VYLPLTSHI	9	0.0047	SNX8	ENSG00000106266.11	ENSP00000222990.3	16.8	1395
AVIGYLSTR	9	9.8029	NOP9	ENSG00000196943.14	ENSP00000267425.3	15.3	1908
KYLVLANML	9	0.1245	COPS2	ENSG00000166200.15	ENSP00000373553.5	20.9	1329
TYVRWYTQL	9	0.0521	CLCC1	ENSG00000121940.15	ENSP00000358987.3	21.2	1503
KYHIVKQIF	9	0.0238	AC098582.1	ENSG00000287542.1	ENSP00000421021.2	14.3	3126
NYITAALKL	9	0.1203	AC098582.1	ENSG00000287542.1	ENSP00000421021.2	14.3	3126
AYVEKVEKL	9	0.0288	AMOTL1	ENSG00000166025.18	ENSP00000387739.2	22.4	2868
KYLEESTIRHF	11	0.0224	AMOTL1	ENSG00000166025.18	ENSP00000387739.2	22.4	2868
RYTPVGRSFF	10	0.0225	AGO4	ENSG00000134698.11	ENSP00000362306.3	17.2	2583
SYIDLLQRF	9	0.0013	WDR24	ENSG00000127580.17	ENSP00000293883.4	15.6	2370
HYVEKPSTF	9	0.0078	GMPPA	ENSG00000144591.18	ENSP00000363027.3	52.0	1419
EVMQAVARL	9	7.4740	TCAF1	ENSG00000198420.10	ENSP00000419235.1	17.2	2763
EIYTGTSVAR	10	14.3556	LMTK2	ENSG00000164715.6	ENSP00000297293.5	14.2	4509
QYSPLLAAF	9	0.0084	BZW1	ENSG00000082153.18	ENSP00000394316.2	36.6	1353
YYEYNHDLF	9	0.0995	SAV1	ENSG00000151748.14	ENSP00000324729.4	17.7	1149
VYSDYLQTI	9	0.0057	PCED1B	ENSG00000179715.13	ENSP00000446688.1	16.3	1296
AYAENIALL	9	0.0387	PIK3R4	ENSG00000196455.8	ENSP00000349205.3	15.4	4074
MLLVGKDGNV	10	56.6667	CCDC80	ENSG00000091986.15	ENSP00000206423.3	18.5	2850
YWMHVQNTF	9	0.0130	KLHL24	ENSG00000114796.16	ENSP00000395012.1	19.3	1800
KYLTEGLLQF	10	0.0110	NFE2L1	ENSG00000082641.16	ENSP00000355190.3	108.9	2283
AVIDQGLITR	10	21.2273	C11orf98	ENSG00000278615.4	ENSP00000432523.1	17.3	369
NYSNIRFQF	9	0.0096	MTMR6	ENSG00000139505.11	ENSP00000371221.5	14.1	1863
RYSEYAEEF	9	0.0054	MTMR6	ENSG00000139505.11	ENSP00000371221.5	14.1	1863
PYQSQIAVF	9	0.0263	RAD17	ENSG00000152942.19	ENSP00000350725.2	20.0	1515

EYMEHVYLI	9	0.0119	OSBPL2	ENSG00000130703.16	ENSP00000494549.1	39.2	1440
MVVGRGLLGR	10	41.5000	HAGH	ENSG00000063854.13	ENSP00000380514.3	62.6	924
RYTDRDFYF	9	0.0094	AL022312.1	ENSG00000285025.1	ENSP00000490747.1	13.9	210
AYTLLLHTW	9	0.0151	DOCK2	ENSG00000134516.17	ENSP00000429283.2	44.2	5490
RYQTQPVTL	9	0.0166	SVIL	ENSG00000197321.14	ENSP00000348128.4	49.6	6642
QYNPKFQTL	9	0.0043	YY1AP1	ENSG00000163374.19	ENSP00000357314.2	53.7	2250
FYIRRVHNL	9	0.0262	NUP205	ENSG00000155561.15	ENSP00000285968.6	17.6	6036
SYLIIHQRI	9	0.0210	ZNF397	ENSG00000186812.13	ENSP00000331577.6	19.1	1602
SYLTSASSL	9	0.1760	UBR5	ENSG00000104517.13	ENSP00000429084.1	38.4	8397
VYSQIPAAVKL	11	0.1193	UBR5	ENSG00000104517.13	ENSP00000429084.1	38.4	8397
AYGGAFYAF	9	0.0102	L3HYPDH	ENSG00000126790.12	ENSP00000247194.4	46.1	1062
TYQSITERI	9	0.0213	EXOC4	ENSG00000131558.15	ENSP00000253861.4	22.8	2922
KYVSGEFRF	9	0.0074	RIOK1	ENSG00000124784.9	ENSP00000369162.2	15.6	1704
AYLSKAMEI	9	0.0860	RIOK1	ENSG00000124784.9	ENSP00000369162.2	15.6	1704
ELKQLAAAR	9	38.3333	SPTAN1	ENSG00000197694.15	ENSP00000487444.1	112.3	7494
EVIEKFDYV	9	9.2330	PMM1	ENSG00000100417.12	ENSP00000216259.7	91.1	786
ETVGTGIMGR	10	47.0000	INPP5B	ENSG00000204084.13	ENSP00000362118.1	19.4	2247
VYYPVRHHL	9	0.0050	TRRAP	ENSG00000196367.13	ENSP00000403708.3	19.9	11544
RYFENPQVI	9	0.0138	TRRAP	ENSG00000196367.13	ENSP00000403708.3	19.9	11544
RYLVISEKL	9	0.0254	LRRC42	ENSG00000116212.15	ENSP00000360421.3	21.6	1284
KYFEKQFEL	9	0.0092	TATDN1	ENSG00000147687.19	ENSP00000430274.1	29.2	966
SYQKLLFKF	9	0.0027	RB1CC1	ENSG00000023287.13	ENSP00000396067.2	14.8	4773
EQYYAMKI	8	19.0968	PRKACB	ENSG00000142875.19	ENSP00000479339.1	25.8	1014
AYINRASLL	9	0.1419	COPS4	ENSG00000138663.9	ENSP00000424655.1	28.2	1260
NYQNVVHKL	9	0.0081	ZMYM4	ENSG00000146463.11	ENSP00000322915.6	16.8	4644
FYQPISHQW	9	0.0018	ADAMTSL3	ENSG00000156218.13	ENSP00000286744.5	15.9	5073
KYFEVPSVL	9	0.0163	ACTR10	ENSG00000131966.14	ENSP00000254286.4	37.4	1251

ETAFGYKGL	9	15.4783	HAT1	ENSG00000128708.13	ENSP00000264108.4	31.0	1257
EYLADLYHF	9	0.0065	SH3BP1	ENSG00000100092.23	ENSP00000497104.1	24.1	2103
IFTNTVARF	9	0.0604	AKAP11	ENSG00000023516.9	ENSP00000025301.2	13.0	5703
DYAELLQHF	9	0.0174	VAC14	ENSG00000103043.15	ENSP00000439284.2	34.6	642
RYIRDAHTF	9	0.0026	ARHGAP35	ENSG00000160007.18	ENSP00000385720.2	16.7	4497
IYHPNVDKL	9	0.0167	UBE2N	ENSG00000177889.10	ENSP00000316176.2	17.6	456
RYLDEINLL	9	0.0090	GNA15	ENSG00000060558.3	ENSP00000262958.2	12.9	1122
RYNPRTNQW	9	0.0172	KLHL20	ENSG00000076321.11	ENSP00000209884.4	15.8	1827
PYVNNVPHL	9	0.0854	MARS	ENSG00000166986.15	ENSP00000446168.2	71.0	1638
DYAYLREHF	9	0.1404	ZNF598	ENSG00000167962.14	ENSP00000455308.2	22.3	2712
IYQFIMDRF	9	0.0122	FOXC2	ENSG00000176692.8	ENSP00000497759.1	12.7	1503
KYISKPENL	9	0.0178	CAB39	ENSG00000135932.11	ENSP00000258418.5	15.7	1023
KYAQWEESL	9	0.1067	CRNKL1	ENSG00000101343.14	ENSP00000440733.1	15.6	2061
RFEEKHAYF	9	0.1403	CRNKL1	ENSG00000101343.14	ENSP00000440733.1	15.6	2061
LYLGRAEEF	9	0.0144	CHPF2	ENSG00000033100.16	ENSP00000035307.2	17.4	2316
TYLNYVVHL	9	0.0154	ZNF292	ENSG00000188994.13	ENSP00000342847.4	15.7	8154
VYSFVTPTF	9	0.0016	PLXNA3	ENSG00000130827.6	ENSP00000358696.3	23.5	5613
VYVDLGGSHVF	11	0.0196	BMS1	ENSG00000165733.8	ENSP00000363642.4	12.5	3846
YATHPFKF	8	0.3811	NCOA7	ENSG00000111912.20	ENSP00000357341.3	33.6	2826
AYATHPFKF	9	0.0033	NCOA7	ENSG00000111912.20	ENSP00000357341.3	33.6	2826
YYNINQVTL	9	0.0300	NFIX	ENSG00000008441.16	ENSP00000353219.4	25.3	1209
VYPLMKEYF	9	0.0120	TFIP11	ENSG00000100109.17	ENSP00000384421.1	25.7	2511
KYGIVQEF	8	0.0790	SOGA1	ENSG00000149639.15	ENSP00000237536.4	13.1	4983
KYGIVQEFF	9	0.0093	SOGA1	ENSG00000149639.15	ENSP00000237536.4	13.1	4983
RYKQDVERF	9	0.0141	SMC5	ENSG00000198887.9	ENSP00000354957.5	16.7	3303
PYSEKMTVLF	10	0.0749	SMC5	ENSG00000198887.9	ENSP00000354957.5	16.7	3303
IYEETRGVL	9	1.1586	HIST2H4B	ENSG00000270276.2	ENSP00000482412.1	12.5	309

IYEETRGVLKVF	12	0.8699	HIST2H4B	ENSG00000270276.2	ENSP00000482412.1	12.5	309
YEETRGVLKVFL	12	30.8333	HIST2H4B	ENSG00000270276.2	ENSP00000482412.1	12.5	309
DNIQGITKPAIR	12	70.0000	HIST2H4B	ENSG00000270276.2	ENSP00000482412.1	12.5	309
KYVKIFDNF	9	0.0045	ZNF195	ENSG00000005801.18	ENSP00000435828.1	19.3	1830
VYPSSLSKI	9	0.0257	CAND1	ENSG00000111530.13	ENSP00000442318.1	20.1	3690
HYIERIQKL	9	0.0099	KANK1	ENSG00000107104.18	ENSP00000371730.3	42.9	3582
VYNTATNQWF	10	0.0599	HCFC1	ENSG00000172534.14	ENSP00000359001.4	20.5	6240
KYGQGFYLI	9	0.0105	LLGL2	ENSG00000073350.13	ENSP00000464397.1	64.2	3057
KYLINLETL	9	0.0319	JAK2	ENSG00000096968.13	ENSP00000371067.3	13.7	3396
YQRAFQHL	8	1.3761	PRKDC	ENSG00000253729.7	ENSP00000345182.4	20.5	12291
QSTRLPLI	8	18.3947	PRKDC	ENSG00000253729.7	ENSP00000345182.4	20.5	12291
LYQRAFQHL	9	0.0160	PRKDC	ENSG00000253729.7	ENSP00000345182.4	20.5	12291
VYTPVLEHL	9	0.0038	PRKDC	ENSG00000253729.7	ENSP00000345182.4	20.5	12291
PYRLIFEKF	9	0.1154	PRKDC	ENSG00000253729.7	ENSP00000345182.4	20.5	12291
ESILLPRI	8	22.3684	SPG11	ENSG00000104133.15	ENSP00000445278.2	38.4	6990
SYASLQQNKW	10	0.1314	SPG11	ENSG00000104133.15	ENSP00000445278.2	38.4	6990
NYNEKIYEL	9	0.0292	UFD1	ENSG00000070010.19	ENSP00000263202.9	34.1	921
DYSSILQKF	9	0.0077	FNBP1	ENSG00000187239.17	ENSP00000407548.2	40.9	1836
AYHNSPAYL	9	0.1615	SMARCE1	ENSG00000073584.20	ENSP00000493649.1	59.7	345
YYLNRTHML	9	0.0132	FARS2	ENSG00000145982.12	ENSP00000274680.3	17.0	1353
FYMDTSHLF	9	0.0010	PRRC2C	ENSG00000117523.16	ENSP00000495867.1	53.2	3270
GYIESVQHI	9	0.0253	MGME1	ENSG00000125871.14	ENSP00000366939.5	28.6	1032
EVVDLLLAR	9	25.8333	ANKHD1-EIF4EBP3	ENSG00000254996.5	ENSP00000432016.1	44.5	7851
EVVSLLLDR	9	43.0000	ANKHD1-EIF4EBP3	ENSG00000254996.5	ENSP00000432016.1	44.5	7851
EVFANRFTQM	10	7.5680	DENND5A	ENSG00000184014.8	ENSP00000328524.3	98.5	3861
TQPGTGWVQF	10	0.6774	NECAP2	ENSG00000157191.20	ENSP00000427620.1	57.0	789
HVFDRPYPLA	10	15.5362	CUEDC1	ENSG00000180891.13	ENSP00000462717.1	31.8	1158

RYGPIVDVY	9	0.1877	SRSF10	ENSG00000188529.14	ENSP00000344149.4	50.0	783
YYLTDIDRI	9	0.1415	GNA14	ENSG00000156049.7	ENSP00000365807.4	12.6	1065
HYSTLVHMF	9	0.0039	DNAH11	ENSG00000105877.18	ENSP00000475939.1	23.2	13548
KYIGNLDLL	9	0.0547	DNAH11	ENSG00000105877.18	ENSP00000475939.1	23.2	13548
AYGDIKERL	9	0.1340	TBKBP1	ENSG00000198933.9	ENSP00000354777.3	15.1	1845
AYLEAHETF	9	0.0026	NUP107	ENSG00000111581.10	ENSP00000441448.1	19.3	2688
EVFDFRGMRL	10	8.8067	NCKAP1	ENSG00000061676.15	ENSP00000355348.3	18.7	3384
IYGYVAEQF	9	0.0035	SPCS1	ENSG00000114902.14	ENSP00000478310.2	42.9	306
RYPAIIYNI	9	0.0087	SLC35B1	ENSG00000121073.14	ENSP00000423323.1	45.9	681
TYLTSIPSI	9	0.0302	MCF2L	ENSG00000126217.21	ENSP00000397285.1	91.3	3201
VYAKLLHRY	9	0.1186	FBXO31	ENSG00000103264.18	ENSP00000310841.4	15.9	1617
FEYQVRSI	8	16.0545	DDX42	ENSG00000198231.13	ENSP00000352308.5	67.7	2457
SYVHYVFRL	9	0.0545	DOCK8	ENSG00000107099.15	ENSP00000371766.1	41.4	4698
NYIGLINRI	9	0.0555	EIF2AK2	ENSG00000055332.18	ENSP00000233057.4	13.3	1653
SYLEKQVVTF	10	0.0074	TTC27	ENSG00000018699.13	ENSP00000313953.4	12.0	2529
NYKDLNGNVF	10	0.2590	SSR1	ENSG00000124783.14	ENSP00000244763.4	15.1	858
RYLRVEHHF	9	0.0028	ACACB	ENSG00000076555.15	ENSP00000367079.3	23.5	7374
AYLEAYKEF	9	0.0072	ETNK1	ENSG00000139163.15	ENSP00000266517.3	15.6	1356
RYLEKSGVL	9	0.2609	MYCBP	ENSG00000214114.9	ENSP00000380702.2	13.2	309
EVWPHSTER	9	13.3694	LYSMD1	ENSG00000163155.12	ENSP00000357904.5	11.8	681
KYPDIISRI	9	0.0067	LIG1	ENSG00000105486.14	ENSP00000471836.1	27.7	2403
RYSPVLSRF	9	0.0012	COG1	ENSG00000166685.12	ENSP00000400111.3	33.8	2886
QYNPHVHQL	9	0.0061	DLG5	ENSG00000151208.17	ENSP00000394797.1	18.6	2640
SQRFTVQI	8	14.4444	TAF9B	ENSG00000187325.5	ENSP00000339917.5	11.6	753
VYMNRVKEI	9	0.0801	C1D	ENSG00000197223.11	ENSP00000386468.3	22.3	423
KYAELRDTI	9	0.0930	MYO6	ENSG00000196586.15	ENSP00000358992.1	18.4	3759
PLPKETWKF	9	0.2212	AOAH	ENSG00000136250.11	ENSP00000479664.1	47.4	2064

VYMNVMTRL	9	0.0185	PPP2R3A	ENSG00000073711.11	ENSP00000264977.3	12.6	3450
LFIATSQKF	9	0.0541	NCAPH2	ENSG00000025770.19	ENSP00000410088.2	46.8	1815
SYTRLFSNF	9	0.0087	UBP1	ENSG00000153560.12	ENSP00000283629.3	27.6	1620
EVISKTPTL	9	2.3082	CREBRF	ENSG00000164463.12	ENSP00000296953.2	12.9	1917
QYVVDLTSF	9	0.0385	NTPCR	ENSG00000135778.12	ENSP00000355587.4	26.8	570
VMYRVIQV	8	13.3514	USF1	ENSG00000158773.14	ENSP00000435005.1	123.5	651
EVLQGGSQR	9	32.2000	USF1	ENSG00000158773.14	ENSP00000435005.1	123.5	651
RYLELISSI	9	0.0205	IFT122	ENSG00000163913.11	ENSP00000425536.1	36.0	3702
SYNPITHQL	9	0.0025	IFT122	ENSG00000163913.11	ENSP00000425536.1	36.0	3702
TMLGKFYHF	9	0.0873	IFT122	ENSG00000163913.11	ENSP00000425536.1	36.0	3702
PWPKVLKI	8	1.6966	EPB41	ENSG00000159023.21	ENSP00000497875.1	27.4	1626
KYQPRIAVF	9	0.0027	TDG	ENSG00000139372.15	ENSP00000376611.3	17.9	1230
YYAKEIHKF	9	0.0007	TGFB3	ENSG00000119699.7	ENSP00000238682.3	22.8	1236
IYVPSSYHL	9	0.0039	SLF2	ENSG00000119906.13	ENSP00000238961.3	19.2	3519
KYQIINEEF	9	0.0066	HPS3	ENSG00000163755.8	ENSP00000296051.2	42.1	3012
QYLPSPPLL	9	0.0066	KCNJ5	ENSG00000120457.12	ENSP00000433295.1	11.5	1257
NYIMKIHNF	9	0.0043	ADNP	ENSG00000101126.17	ENSP00000495540.1	15.0	2622
VYPEEHSRW	9	0.0249	RSRP1	ENSG00000117616.18	ENSP00000391510.3	71.3	726
SYGDILHVI	9	0.0092	DLG3	ENSG00000082458.12	ENSP00000441393.1	13.0	1098
IYNKIKQII	9	0.0481	DLG3	ENSG00000082458.12	ENSP00000441393.1	13.0	1098
KYIDQKFVL	9	0.0265	PPP2R5D	ENSG00000112640.15	ENSP00000417963.1	17.0	1806
NYAQVLDKF	9	0.0094	ASAP1	ENSG00000153317.15	ENSP00000350297.2	30.0	3366
TYDSVTDKF	9	0.0408	USP34	ENSG00000115464.14	ENSP00000381577.2	21.7	10638
KYIKDDFRF	9	0.0068	RIOK3	ENSG00000101782.15	ENSP00000462548.1	34.9	1548
LYQDQILEKF	10	0.0214	TUBE1	ENSG00000074935.14	ENSP00000474458.1	21.8	660
YYLNDLDRI	9	0.1787	GNAI1	ENSG00000127955.17	ENSP00000343027.3	18.2	1062
RYDSVINRL	9	0.0451	PCF11	ENSG00000165494.11	ENSP00000298281.4	54.1	4665

DVIERVIQY	9	5.7231	NIPBL	ENSG00000164190.19	ENSP00000282516.8	17.2	8412
EVPSFTMGR	9	13.1892	PRKRIP1	ENSG00000128563.13	ENSP00000419270.1	51.5	552
RFKNKAYLL	9	0.2653	DICER1	ENSG00000100697.14	ENSP00000376783.1	14.3	5766
GVISMPVAAR	10	41.5000	FP565260.6	ENSG00000280433.1	ENSP00000485615.1	11.1	609
EVAEKIWSNR	10	27.1000	ARAP3	ENSG00000120318.16	ENSP00000421468.1	48.9	3579
RYASINAHL	9	0.0369	CSNK1A1	ENSG00000113712.18	ENSP00000499757.1	33.5	975
HYMPPPYASL	10	0.0343	RNF111	ENSG00000157450.15	ENSP00000453015.1	15.3	3009
IYTNLNQKI	9	0.0304	EXOC6B	ENSG00000144036.15	ENSP00000272427.6	12.2	2433
SVYDGEEHGR	10	12.8750	EXOC6B	ENSG00000144036.15	ENSP00000272427.6	12.2	2433
TYLPQVSHYRF	11	0.0111	EXOC6B	ENSG00000144036.15	ENSP00000272427.6	12.2	2433
VYPVITARL	9	0.0206	ZNF106	ENSG00000103994.17	ENSP00000263805.4	14.4	5649
HYNSHYEKF	9	0.0073	ASTN2	ENSG00000148219.16	ENSP00000363098.3	12.2	3186
VYVERAEVL	9	0.0898	BRAP	ENSG00000089234.16	ENSP00000403524.3	10.9	1776
VYLHDFQRF	9	0.0021	PLCG2	ENSG00000197943.10	ENSP00000482457.1	19.3	3795
DLISEDVQRR	10	45.0000	ARHGEF1	ENSG00000076928.17	ENSP00000367394.3	201.0	2844
TYAQRTQLF	9	0.0047	MKLN1	ENSG00000128585.18	ENSP00000398094.2	22.7	1929
KYITDWQNVF	10	0.0204	PDCD6	ENSG00000249915.8	ENSP00000423815.1	112.2	567
AYVEKVERL	9	0.0429	AMOTL2	ENSG00000114019.14	ENSP00000424765.1	38.8	2511
KYLEERAMRQF	11	0.0864	AMOTL2	ENSG00000114019.14	ENSP00000424765.1	38.8	2511
IYTKIMDLI	9	0.0965	DPY19L1	ENSG00000173852.14	ENSP00000490722.1	12.0	2244
SYSQSSNLF	9	0.0098	ZNF24	ENSG00000172466.16	ENSP00000261332.5	23.0	1104
ETAEQGLLNR	10	40.0000	ZFX	ENSG00000005889.15	ENSP00000368475.1	16.6	2415
AYNPMARDLF	10	0.0647	MTOR	ENSG00000198793.12	ENSP00000354558.4	27.1	7647
IYADNQVMHF	10	0.0050	POLE	ENSG00000177084.16	ENSP00000322570.5	18.5	6858
VYTYIQSRF	9	0.0084	DYRK2	ENSG00000127334.10	ENSP00000342105.3	13.7	1803
SYPDNFLHI	9	0.0090	ATG5	ENSG00000057663.16	ENSP00000358072.3	17.9	825
KYMSVIAEL	9	0.0139	ATPAF1	ENSG00000123472.12	ENSP00000460964.1	17.7	1053

VYAVIPAEKF	10	0.0179	ATPAF1	ENSG00000123472.12	ENSP00000460964.1	17.7	1053
QYEKLFHKW	9	0.0203	ORC2	ENSG00000115942.9	ENSP00000234296.2	18.9	1731
MYINEVERL	9	0.0286	PTPN14	ENSG00000152104.12	ENSP00000355923.4	11.4	3561
RYLHSHHFL	9	0.0123	VIRMA	ENSG00000164944.12	ENSP00000297591.5	20.6	5436
EVVELPVTER	10	37.0000	TBC1D10A	ENSG00000099992.15	ENSP00000215790.7	23.1	1524
LYSEVDVHF	9	0.0052	POGZ	ENSG00000143442.22	ENSP00000431259.1	36.9	4044
IYQRHVYNL	9	0.0095	TMEM209	ENSG00000146842.17	ENSP00000380747.2	10.7	1683
PYNHQHEYF	9	0.0658	FADS2	ENSG00000134824.14	ENSP00000431091.1	25.0	1158
EYRKVFENF	9	0.0584	IGF1R	ENSG00000140443.15	ENSP00000496919.1	30.8	4098
VYNSEYYHF	9	0.0011	PTK7	ENSG00000112655.16	ENSP00000418754.1	14.7	3234
TYLEKAVEV	9	0.2187	ATM	ENSG00000149311.18	ENSP00000278616.4	39.2	9168
KYSSGFRNI	9	0.0808	ATM	ENSG00000149311.18	ENSP00000278616.4	39.2	9168
VYTSVVEEL	9	0.0209	MAN2B2	ENSG00000013288.8	ENSP00000285599.3	20.7	3027
YIKQIKTF	8	0.4863	RBL2	ENSG00000103479.16	ENSP00000262133.6	111.5	3417
IYIKQIKTF	9	0.0027	RBL2	ENSG00000103479.16	ENSP00000262133.6	111.5	3417
EVADRLFSSF	10	4.4254	TAPT1	ENSG00000169762.17	ENSP00000385347.2	18.1	1701
VQPSLFYHL	9	0.2026	MAP3K6	ENSG00000142733.17	ENSP00000363152.2	54.3	3840
EVIDVVRTM	9	3.9045	TAF1	ENSG00000147133.15	ENSP00000276072.3	19.8	5679
DYMKTTSNF	9	0.0203	NEMF	ENSG00000165525.18	ENSP00000298310.5	18.3	3228
TYDYNFHSF	9	0.0054	ASH1L	ENSG00000116539.13	ENSP00000376204.3	12.9	8892
FYIHEVQEL	9	0.0347	HERC4	ENSG00000148634.15	ENSP00000362804.4	23.0	3147
RYPQSIFSTF	10	0.0036	SBF2	ENSG00000133812.15	ENSP00000256190.8	26.7	5547
RYALIMHKL	9	0.0091	ZBTB11	ENSG00000066422.4	ENSP00000326200.4	11.2	3159
TYLTNHLRL	9	0.0482	ZNF697	ENSG00000143067.5	ENSP00000396857.2	9.5	1635
VYTTTRSHL	9	0.2157	CLPTM1L	ENSG00000049656.14	ENSP00000313854.5	143.5	1614
PYMAKFVVF	9	0.0179	ANK3	ENSG00000151150.22	ENSP00000362933.2	21.7	5583
TVISRVIGR	9	15.8406	ANKRD17	ENSG00000132466.18	ENSP00000427151.2	26.8	7470

SYLTIHHRI	9	0.0074	ZNF12	ENSG00000164631.19	ENSP00000385939.1	15.2	2091
RYMDLAENARF	11	0.0206	SPECC1L	ENSG00000100014.20	ENSP00000499052.1	12.9	3408
KYSNMEQSLF	10	0.0586	AQR	ENSG00000021776.11	ENSP00000156471.5	12.7	4455
IYPQVLHSL	9	0.0045	AC124319.1	ENSG00000173821.19	ENSP00000464087.1	156.1	15621
ETASKTLSR	9	15.2754	AC124319.1	ENSG00000173821.19	ENSP00000464087.1	156.1	15621
DLAPFSLRKR	10	29.2857	AC124319.1	ENSG00000173821.19	ENSP00000464087.1	156.1	15621
KYSVINEINKI	11	0.3989	AC124319.1	ENSG00000173821.19	ENSP00000464087.1	156.1	15621
KYMEDVTQI	9	0.0173	PPM1D	ENSG00000170836.11	ENSP00000306682.2	11.1	1815
HYHPIVQRF	9	0.0015	NOC3L	ENSG00000173145.11	ENSP00000360412.3	10.9	2400
TYTSNLVRL	9	0.0535	DNAJC16	ENSG00000116138.13	ENSP00000480224.1	12.1	1410
AQRLMQLI	8	19.2581	MED12	ENSG00000184634.16	ENSP00000333125.7	27.7	6081
EYMIQFNRL	9	0.0961	HELZ2	ENSG00000130589.16	ENSP00000393257.2	14.7	6240
VYTSGVYHI	9	0.0076	TNS2	ENSG00000111077.17	ENSP00000449361.1	204.6	4032
PYFNAPVYL	9	0.0836	GAR1	ENSG00000109534.17	ENSP00000378127.3	26.6	651
GQFLVRII	8	22.9474	GUCY1B1	ENSG00000061918.13	ENSP00000420842.1	40.2	1758
MYGFVNHAL	9	0.1536	GUCY1B1	ENSG00000061918.13	ENSP00000420842.1	40.2	1758
VYPNSFFRY	9	0.0928	MORN1	ENSG00000116151.14	ENSP00000367792.3	16.5	1491
TYLEKFQNL	9	0.0057	CLUAP1	ENSG00000103351.13	ENSP00000460850.1	18.7	1239
EYNSALPLL	9	0.1042	GTF3C3	ENSG00000119041.11	ENSP00000263956.3	22.4	2658
VYTDFAFRI	9	0.0366	NOL9	ENSG00000162408.11	ENSP00000366934.5	11.6	2106
RYLEKNVKL	9	0.0248	TGS1	ENSG00000137574.10	ENSP00000260129.5	9.9	2559
YYSAVTPHL	9	0.0111	PTBP2	ENSG00000117569.18	ENSP00000359216.1	16.6	1611
TYISAIRERF	10	0.0194	NXF1	ENSG00000162231.14	ENSP00000294172.2	101.7	1857
RYLEAALRL	9	0.0225	P3H4	ENSG00000141696.13	ENSP00000347649.2	23.2	1311
SQYLTRITI	9	2.3817	ANAPC1	ENSG00000153107.13	ENSP00000339109.3	13.7	5832
VYSEAARVLQF	11	0.0210	GALK2	ENSG00000156958.15	ENSP00000453133.1	18.9	1302
RYKFHNSRW	9	0.2913	TBX3	ENSG00000135111.16	ENSP00000257567.2	11.9	2169

KYLHPPTHL	9	0.0187	MBNL2	ENSG00000139793.18	ENSP00000432422.1	24.8	765
NYTDIKDYL	9	0.2450	NUDT12	ENSG00000112874.10	ENSP00000230792.2	11.2	1386
SFYKIRTI	8	1.6850	NEK9	ENSG00000119638.13	ENSP00000238616.5	47.4	2937
RYQEVIQEL	9	0.0036	ZFYVE26	ENSG00000072121.16	ENSP00000251119.5	14.2	7617
RYHLVQQLF	9	0.0035	FANCF	ENSG00000183161.5	ENSP00000330875.3	8.9	1122
AMVRVISV	8	30.1667	EDC4	ENSG00000038358.15	ENSP00000351811.5	43.0	4203
KYPSDLPYM	9	0.0753	ERG	ENSG00000157554.19	ENSP00000288319.7	14.1	1437
RFEEAHFTF	9	0.0219	PIAS3	ENSG00000131788.16	ENSP00000376765.2	39.5	1884
FYTVIPHNF	9	0.0028	PARP3	ENSG00000041880.14	ENSP00000395951.2	27.5	1599
NYSPYVNYF	9	0.0028	DPY19L4	ENSG00000156162.16	ENSP00000389630.2	12.1	2169
VYPGDPLRF	9	0.0068	TSEN34	ENSG00000170892.12	ENSP00000379667.1	25.4	930
TYVSGTLRF	9	0.0075	CEPT1	ENSG00000134255.14	ENSP00000477632.1	20.4	840
RYAVIGADL	9	0.4147	LCMT1	ENSG00000205629.12	ENSP00000382021.3	31.7	1002
RQYLAINQI	9	2.0411	ITPR2	ENSG00000123104.12	ENSP00000370744.3	9.2	8103
TYPQLEGFKF	10	0.0214	WDFY4	ENSG00000128815.19	ENSP00000320563.5	12.4	9552
KYVKGLISI	9	0.0479	WDR35	ENSG00000118965.14	ENSP00000281405.4	9.5	3510
KYKEAAHLL	9	0.0110	KLC2	ENSG00000174996.11	ENSP00000377631.2	22.5	1866
VYENVSHFL	9	0.0503	SLC25A32	ENSG00000164933.12	ENSP00000297578.4	10.0	945
SMPDVLKI	8	3.5362	TASOR	ENSG00000163946.13	ENSP00000399410.2	19.2	3699
RYIFAKNLF	9	0.0116	DGUOK	ENSG00000114956.20	ENSP00000264093.4	42.0	831
EVQDLHLAQR	10	36.0000	MBOAT7	ENSG00000125505.17	ENSP00000375634.1	33.3	1032
KYRQKFNTL	9	0.0859	ITSN2	ENSG00000198399.14	ENSP00000479408.1	31.4	5040
NYLTKIYGL	9	0.1445	PODN	ENSG00000174348.13	ENSP00000308315.5	15.8	1983
KYFSQASSL	9	0.0975	ZNF226	ENSG00000167380.16	ENSP00000465121.1	21.3	2409
EVMLRVKEM	9	11.2446	CDYL2	ENSG00000166446.15	ENSP00000476295.1	8.4	1518
EVIRTLPSL	9	4.4804	INPP5D	ENSG00000168918.14	ENSP00000405338.2	60.0	3567
RYQFIEEAF	9	0.0253	TRIM33	ENSG00000197323.12	ENSP00000351250.2	14.2	3381

RYPVGRFPSL	10	0.1852	FEM1A	ENSG00000141965.4	ENSP00000269856.3	8.3	2007
FWRGGQYENF	10	0.5601	ZC3H18	ENSG00000158545.15	ENSP00000416951.2	29.8	2931
IYSTKLYRF	9	0.0022	BTBD10	ENSG00000148925.10	ENSP00000435257.1	20.9	1281
KYSDVKNLI	9	0.0247	ZFAT	ENSG00000066827.16	ENSP00000428483.1	12.8	3693
SYQKVMALW	9	0.0167	MACF1	ENSG00000127603.26	ENSP00000362016.2	70.9	13302
KYPLLISRI	9	0.0231	ARHGEF2	ENSG00000116584.18	ENSP00000354837.4	92.7	2958
IYPSDSFRQSL	11	0.2160	ARHGEF2	ENSG00000116584.18	ENSP00000354837.4	92.7	2958
YYEKLHTYF	9	0.0121	ITGB4	ENSG00000132470.14	ENSP00000400217.2	14.3	5415
EYLERAPEL	9	0.1951	XRN1	ENSG00000114127.10	ENSP00000264951.4	12.7	5118
IMPSSSHLF	9	0.0176	XRN1	ENSG00000114127.10	ENSP00000264951.4	12.7	5118
TYVTILPEL	9	0.0485	XRN1	ENSG00000114127.10	ENSP00000264951.4	12.7	5118
TYPAELNNI	9	0.0939	AHR	ENSG00000106546.14	ENSP00000242057.4	8.8	2544
RYGIHPAKF	9	0.0094	VWA8	ENSG00000102763.18	ENSP00000368612.3	8.8	5715
PYADQVFRI	9	0.0718	HELZ	ENSG00000198265.12	ENSP00000351524.5	16.3	5826
YYPNPPLVL	9	0.0318	NT5DC1	ENSG00000178425.14	ENSP00000326858.3	10.6	1365
IYNFPIHAF	9	0.0042	MAK16	ENSG00000198042.11	ENSP00000353246.5	8.6	900
HYKPTPLYF	9	0.0037	SMC4	ENSG00000113810.16	ENSP00000349961.3	19.9	3864
VYLDKFIRL	9	0.0061	GNL3L	ENSG00000130119.16	ENSP00000354091.2	8.4	1746
KYLESVKPF	9	0.0194	CROT	ENSG00000005469.11	ENSP00000331981.3	10.6	1836
LYSDIGHLL	9	0.0129	SESN3	ENSG00000149212.11	ENSP00000441927.1	8.4	1476
ETIGRLLQL	9	3.7222	DEPDC7	ENSG00000121690.11	ENSP00000308971.3	15.3	1506
SYIHYVFRL	9	0.0262	DOCK7	ENSG00000116641.17	ENSP00000340742.5	16.3	6327
DVIGNEILRR	10	48.0000	CLIP3	ENSG00000105270.15	ENSP00000353732.3	8.5	1641
SYVWRTYHL	9	0.0320	SNRNP25	ENSG00000161981.10	ENSP00000372482.3	23.4	396
KYFLKPEVL	9	0.0347	NCEH1	ENSG00000144959.10	ENSP00000442464.1	23.3	1344
VYVDKVEKM	9	0.0346	AMOT	ENSG00000126016.15	ENSP00000361027.3	13.6	3252
KYLEENVMRHF	11	0.0369	AMOT	ENSG00000126016.15	ENSP00000361027.3	13.6	3252

TQYKFRLTA	9	14.3000	FNDC3B	ENSG00000075420.13	ENSP00000338523.4	23.0	3612
QYMERLQLL	9	0.0112	MTX1	ENSG00000173171.14	ENSP00000357360.3	22.8	1398
NYVEVTRKW	9	0.0602	MTX1	ENSG00000173171.14	ENSP00000357360.3	22.8	1398
EYAKIEETM	9	0.2458	CADPS2	ENSG00000081803.16	ENSP00000400401.2	11.3	3765
YYSHLEGARF	10	0.0970	URB1	ENSG00000142207.7	ENSP00000372199.3	8.1	6813
TYITSVSRL	9	0.0426	OSGIN2	ENSG00000164823.11	ENSP00000297438.2	9.9	1515
KYNIFRSTF	9	0.0240	ZNF181	ENSG00000197841.15	ENSP00000419435.1	12.2	1710
KYKDAANLL	9	0.0484	KLC1	ENSG00000126214.21	ENSP00000412693.4	100.0	1647
RYTLQASTF	9	0.0214	CUL1	ENSG00000055130.17	ENSP00000499276.1	37.6	2277
EVAEHVQYM	9	5.5985	GMNN	ENSG00000112312.10	ENSP00000230056.3	11.5	627
HGMTLKSV	8	38.6667	CDC73	ENSG00000134371.13	ENSP00000356405.3	8.2	1593
DIISQQLVER	10	49.0000	MPV17	ENSG00000115204.15	ENSP00000385175.1	97.9	339
DVHNFPGTSR	10	24.8571	KPTN	ENSG00000118162.14	ENSP00000337850.2	11.1	1308
KYGALVNNF	9	0.0052	NSUN4	ENSG00000117481.10	ENSP00000471937.1	12.2	453
SYGSVFKAI	9	0.1405	STK3	ENSG00000104375.17	ENSP00000482260.1	14.1	1140
VYGTTYQF	8	0.0623	TXNDC16	ENSG00000087301.9	ENSP00000281741.4	8.0	2475
SYIDVAVKL	9	0.0170	TXNDC16	ENSG00000087301.9	ENSP00000281741.4	8.0	2475
RYLIVHQQI	9	0.0117	ZNF432	ENSG00000256087.7	ENSP00000221315.4	9.7	1956
YYDPHQATF	9	0.0021	PCNX1	ENSG00000100731.15	ENSP00000304192.2	15.7	7023
NYSRNFSSF	9	0.0183	PDZD2	ENSG00000133401.16	ENSP00000402033.1	26.1	8517
KYPVLFQRI	9	0.0107	AKAP13	ENSG00000170776.21	ENSP00000378018.3	38.4	4302
IYQREWQRF	9	0.0043	ACTR8	ENSG00000113812.14	ENSP00000336842.3	11.3	1872
HQFRMLVV	8	22.4211	UVSSA	ENSG00000163945.16	ENSP00000421741.1	39.8	2127
NYATRIVTL	9	0.0542	BIRC6	ENSG00000115760.14	ENSP00000393596.2	35.9	14571
VFKPISSTW	9	0.0396	BIRC6	ENSG00000115760.14	ENSP00000393596.2	35.9	14571
DVKDYLRNM	9	8.3678	CCPG1	ENSG00000260916.7	ENSP00000311656.6	14.7	2271
TYGPSFPAF	9	0.0025	RALGAPB	ENSG00000170471.15	ENSP00000262879.6	12.8	4482

RYPDNLKHL	9	0.0512	CCDC59	ENSG00000133773.12	ENSP00000256151.7	30.1	723
RYPDNLKHLYL	11	0.1569	CCDC59	ENSG00000133773.12	ENSP00000256151.7	30.1	723
KYMLKANLI	9	0.0535	NAA15	ENSG00000164134.13	ENSP00000381920.1	16.9	2595
QYIDVSHVF	9	0.0019	RFX2	ENSG00000087903.13	ENSP00000306335.4	12.4	2169
AYALLLQHL	9	0.0246	SLC26A6	ENSG00000225697.13	ENSP00000401066.2	19.9	2169
EVIAYILER	9	22.2632	MED23	ENSG00000112282.18	ENSP00000346588.4	21.5	4095
YDTVFKHF	8	0.8126	SP140L	ENSG00000185404.16	ENSP00000395195.1	26.1	1314
VYDTVFKHF	9	0.0053	SP140L	ENSG00000185404.16	ENSP00000395195.1	26.1	1314
AYVGDLQTL	9	0.0679	ASB1	ENSG00000065802.12	ENSP00000264607.4	7.9	1005
KFVNKSYLL	9	0.1769	SHQ1	ENSG00000144736.14	ENSP00000315182.8	8.4	1731
RYIHKLYDL	9	0.0583	DOCK4	ENSG00000128512.22	ENSP00000499282.1	36.8	2694
MYIRYIHKL	9	0.0063	DOCK4	ENSG00000128512.22	ENSP00000499282.1	36.8	2694
KYGDMRVTM	9	0.1194	DOCK4	ENSG00000128512.22	ENSP00000499282.1	36.8	2694
RFIGATANF	9	0.0477	RNASEH2C	ENSG00000172922.9	ENSP00000308193.4	22.4	492
HYILHNSFF	9	0.0389	TIPARP	ENSG00000163659.13	ENSP00000420612.1	11.4	1971
VTSEDTRIK	9	45.5000	DHX33	ENSG00000005100.13	ENSP00000225296.3	8.4	2121
YYLNEIQSF	9	0.0017	SPATC1L	ENSG00000160284.15	ENSP00000333869.6	10.5	558
SYDPDFNQL	9	0.0814	FTSJ1	ENSG00000068438.15	ENSP00000326948.2	23.0	987
EVFMHVSTAY	10	9.4976	FAR1	ENSG00000197601.13	ENSP00000437111.1	24.2	1005
AYSRVQYQF	9	0.0024	BCAR3	ENSG00000137936.18	ENSP00000359264.1	23.1	2475
VYAKVTHLF	9	0.0004	ANO4	ENSG00000151572.18	ENSP00000494481.1	7.5	3363
VYPERTPLL	9	0.0092	HLA-DOB	ENSG00000241106.8	ENSP00000496848.1	9.3	819
IYDTSTDRLLW	11	0.2313	ISG20	ENSG00000172183.15	ENSP00000306565.5	22.3	543
VYKPAQNSW	9	0.0224	KBTBD7	ENSG00000120696.8	ENSP00000368797.3	6.5	2052
EYIKFLRSI	9	0.1105	HDAC2	ENSG00000196591.12	ENSP00000357621.2	22.3	1374
IFTDVALKF	9	0.0213	GTF3C4	ENSG00000125484.12	ENSP00000361219.4	7.1	2466
KYADLSHNRL	10	0.1365	RARS	ENSG00000113643.9	ENSP00000231572.3	35.5	1980

KYPVEWAKF	9	0.0055	ZC2HC1A	ENSG00000104427.12	ENSP00000263849.3	7.5	975
VYNNNSSRF	9	0.0213	MYO10	ENSG00000145555.15	ENSP00000274203.10	10.7	6207
NYSLIDKHL	9	0.2876	ARHGAP10	ENSG00000071205.11	ENSP00000336923.3	18.7	2358
RYQALFHDF	9	0.0059	VCPIP1	ENSG00000175073.8	ENSP00000309031.4	6.4	3666
RYLNKAFHI	9	0.0183	NCAPD3	ENSG00000151503.12	ENSP00000433681.2	15.6	4494
EVTGFGVTR	9	23.1250	KLC4	ENSG00000137171.15	ENSP00000418546.1	78.0	135
EAIMKVLQR	9	21.9091	SYTL2	ENSG00000137501.17	ENSP00000435238.1	35.1	2730
VYADKLLNI	9	0.0118	CTDSPL2	ENSG00000137770.14	ENSP00000260327.4	15.5	1398
NYIKDLNIL	9	0.1257	CTDSPL2	ENSG00000137770.14	ENSP00000260327.4	15.5	1398
DTITETDLR	9	43.5000	RBM22	ENSG00000086589.12	ENSP00000199814.4	50.5	1260
YQFGEIRTI	9	1.3180	RBM22	ENSG00000086589.12	ENSP00000199814.4	50.5	1260
LYGRHFNYL	9	0.1117	TENT4B	ENSG00000121274.12	ENSP00000455837.1	7.2	1764
SYARHFLDF	9	0.0206	ZADH2	ENSG00000180011.7	ENSP00000323678.3	22.7	1131
YYLLKNEQF	9	0.0044	EPG5	ENSG00000152223.15	ENSP00000282041.4	12.5	7737
SYTSVLSRL	9	0.0565	EPG5	ENSG00000152223.15	ENSP00000282041.4	12.5	7737
KYPHYFPLL	9	0.0063	AC006538.1	ENSG00000172009.15	ENSP00000304467.5	22.3	2067
TYVIYAHLL	9	0.0434	HECTD2	ENSG00000165338.16	ENSP00000298068.5	7.8	2328
GYSEHFVEF	9	0.0065	GMPR	ENSG00000137198.9	ENSP00000259727.4	6.2	1035
AWAPKPYHKF	10	0.0280	TNIK	ENSG00000154310.17	ENSP00000399511.2	11.7	4080
NYSNVSIHL	9	0.0769	CCDC82	ENSG00000149231.14	ENSP00000498266.1	17.0	1563
IYHKLKHFKF	10	0.0942	CCDC82	ENSG00000149231.14	ENSP00000498266.1	17.0	1563
RYDPHLNRW	9	0.0184	KLHL26	ENSG00000167487.12	ENSP00000300976.3	6.4	1845
NYIPYLTKL	9	0.0120	SAMD9	ENSG00000205413.8	ENSP00000369292.2	6.3	4767
KETFYKHSPEAF	12	1.2259	GALNT15	ENSG00000131386.19	ENSP00000344260.5	6.8	1917
SNQMLVPVI	9	10.3015	WDR3	ENSG00000065183.16	ENSP00000308179.4	6.8	2829
AYLNITKHL	9	0.0573	POLK	ENSG00000122008.15	ENSP00000423526.1	12.9	1281
SYPSAFSKL	9	0.0211	MARF1	ENSG00000166783.22	ENSP00000450309.1	45.9	5226

NYKSHHLQL	9	0.1715	CWF19L1	ENSG00000095485.18	ENSP00000326411.6	17.1	1614
RYLPHEELF	9	0.0027	ZNF45	ENSG00000124459.12	ENSP00000481895.1	16.2	2046
SYLTQHQRI	9	0.0377	ZNF264	ENSG00000083844.10	ENSP00000263095.5	8.2	1881
KYQHLKAEF	9	0.0187	EEA1	ENSG00000102189.16	ENSP00000317955.8	5.9	4233
VYVQELQKL	9	0.0133	EEA1	ENSG00000102189.16	ENSP00000317955.8	5.9	4233
QYGEVANLL	9	0.0729	VPS53	ENSG00000141252.20	ENSP00000401435.2	10.4	2496
EAFPSQGTKR	10	18.0000	VPS53	ENSG00000141252.20	ENSP00000401435.2	10.4	2496
RYNHITATY	9	0.1472	SNRK	ENSG00000163788.14	ENSP00000296088.7	21.1	2295
RYNHITATYF	10	0.0305	SNRK	ENSG00000163788.14	ENSP00000296088.7	21.1	2295
VYATMPINSF	10	0.0113	ZNF148	ENSG00000163848.19	ENSP00000353863.4	10.8	2382
RYKGPGFEYF	10	0.0083	RPUSD2	ENSG00000166133.18	ENSP00000323288.7	10.8	1635
KYITDVVKL	9	0.0227	UTP20	ENSG00000120800.5	ENSP00000261637.4	6.1	8355
SYLPISPTF	9	0.0007	SLC23A2	ENSG00000089057.15	ENSP00000344322.1	6.8	1950
QYMELFNKI	9	0.0087	MED20	ENSG00000124641.16	ENSP00000265350.4	7.9	636
VYNPVRAEW	9	0.0093	KBTBD6	ENSG00000165572.7	ENSP00000368799.1	5.6	2022
KYELDFSHF	9	0.0484	TLR2	ENSG00000137462.8	ENSP00000494425.1	9.9	2352
VYSIVPQSW	9	0.0078	LARP4	ENSG00000161813.22	ENSP00000293618.8	11.2	1959
KYLEETNSL	9	0.0234	L3MBTL3	ENSG00000198945.7	ENSP00000437185.1	6.9	2265
EAIGTLTAR	9	26.1000	APBB3	ENSG00000113108.19	ENSP00000349177.2	43.0	1473
RYSVASFKF	9	0.0048	MYORG	ENSG00000164976.9	ENSP00000297625.8	5.5	2142
EYFRVPDSATF	11	0.0650	VPS13B	ENSG00000132549.18	ENSP00000349685.2	18.5	11991
EYTLMRATF	9	0.1153	IRS2	ENSG00000185950.9	ENSP00000365016.3	5.5	4014
KYIEGVSDF	9	0.0087	IPP	ENSG00000197429.10	ENSP00000379739.3	8.0	1752
RYGRFRKF	8	0.5504	DCAF11	ENSG00000100897.17	ENSP00000452898.1	31.6	1638
KYYDKKYQVF	10	0.0196	FGGY	ENSG00000172456.17	ENSP00000360262.4	21.4	1725
VYTGIDHHW	9	0.0124	DCAF13	ENSG00000164934.14	ENSP00000297579.5	13.3	1791
RYSGNQVLF	9	0.0057	RAPGEF6	ENSG00000158987.20	ENSP00000421684.1	9.8	4803

NYENKQYLF	9	0.0346	WDR66	ENSG00000158023.10	ENSP00000380595.2	15.9	2823
IYPMKNHMF	9	0.0089	RALGAPA1	ENSG00000174373.16	ENSP00000450574.1	16.2	1068
RQIKINEV	8	13.0541	TWF1	ENSG00000151239.13	ENSP00000378886.2	24.3	1050
SYANIKEKL	9	0.0550	MTERF1	ENSG00000127989.13	ENSP00000384986.2	9.0	1137
LYQDGVFKF	9	0.0022	AKTIP	ENSG00000166971.17	ENSP00000300245.4	23.6	879
LYEKDIQLF	9	0.0241	AKTIP	ENSG00000166971.17	ENSP00000300245.4	23.6	879
AYLVHIEHL	9	0.0239	MCC	ENSG00000171444.18	ENSP00000305617.4	8.7	2487
AYGKDFHLI	9	0.0208	MIER1	ENSG00000198160.14	ENSP00000347514.3	9.8	1536
IYQKAFEHL	9	0.0057	BBS4	ENSG00000140463.14	ENSP00000268057.4	22.6	1557
QYASAFHFL	9	0.0114	BBS4	ENSG00000140463.14	ENSP00000268057.4	22.6	1557
LYAHTIAGF	9	0.0549	CTDP1	ENSG00000060069.16	ENSP00000075430.7	19.4	2601
KYADKIYSI	9	0.0033	FOLH1	ENSG00000086205.18	ENSP00000256999.2	11.3	2250
SYLKDTKLW	9	0.0551	STK24	ENSG00000102572.14	ENSP00000442539.2	27.9	1293
IYLPEVRKI	9	0.0098	TULP4	ENSG00000130338.13	ENSP00000356064.3	7.6	4629
EYVKFLHKF	9	0.0036	ZNF506	ENSG00000081665.14	ENSP00000440625.1	15.9	1332
TYSPALNKMF	10	0.0206	TP53	ENSG00000141510.17	ENSP00000478219.1	17.2	1062
RYQEVDRRF	9	0.0112	YTHDC1	ENSG00000083896.12	ENSP00000339245.4	78.7	2181
KYVKVFHKF	9	0.0007	ZNF680	ENSG00000173041.12	ENSP00000309330.6	6.7	1590
RYINNPLLI	9	0.0196	TTLL5	ENSG00000119685.20	ENSP00000298832.9	12.9	3843
TYIQKIFRM	9	0.0217	ACOX3	ENSG00000087008.16	ENSP00000348775.4	13.5	2100
IYFEYSHAF	9	0.0022	MSH3	ENSG00000113318.11	ENSP00000499502.1	11.0	3216
VYVQPPLEL	9	0.0205	BBS9	ENSG00000122507.20	ENSP00000388114.1	12.7	1362
EYLGKLVRF	9	0.0124	RSAD2	ENSG00000134321.12	ENSP00000371471.3	6.8	1083
VYSFGFERL	9	0.0620	SLC30A6	ENSG00000152683.14	ENSP00000349563.4	7.2	1296
SYYSPSIGF	9	0.0205	YTHDF2	ENSG00000198492.16	ENSP00000362918.3	9.8	1737
YLEHPLLL	8	0.8666	TTL	ENSG00000114999.8	ENSP00000233336.5	6.6	1131
KYLEHPLLL	9	0.0043	TTL	ENSG00000114999.8	ENSP00000233336.5	6.6	1131

VYAEVSRLLL	10	0.1287	TTL	ENSG00000114999.8	ENSP00000233336.5	6.6	1131
HSTIMPRL	8	16.4909	MCM4	ENSG00000104738.18	ENSP00000497093.1	13.7	2247
VYAVVRGLF	9	0.0388	MFSD13A	ENSG00000138111.14	ENSP00000238936.3	8.5	1551
LYRLQFNEF	9	0.1323	ARL4A	ENSG00000122644.13	ENSP00000379899.2	12.2	600
TQVTVQKI	8	14.9111	UVRAG	ENSG00000198382.9	ENSP00000348455.3	26.0	2097
EVPSFLVERM	10	12.1736	MKS1	ENSG00000011143.16	ENSP00000376827.2	36.8	1677
KYLDEKLAL	9	0.1352	BAIAP3	ENSG00000007516.13	ENSP00000380625.2	12.5	3507
IYPEVVHMF	9	0.0005	PPP2R5C	ENSG00000078304.19	ENSP00000408389.3	44.8	1347
SYVNHLYVI	9	0.0396	RAPGEF5	ENSG00000136237.19	ENSP00000479340.1	23.9	1332
SYLIQHQRF	9	0.0091	ZNF71	ENSG00000197951.9	ENSP00000328245.5	5.8	1467
AYLESFYKF	9	0.0009	ATP6V0D1	ENSG00000159720.12	ENSP00000290949.3	155.4	1053
LYPEGLAQL	9	0.0651	ATP6V0D1	ENSG00000159720.12	ENSP00000290949.3	155.4	1053
KYVKVIHKF	9	0.0009	AC008554.1	ENSG00000237440.9	ENSP00000395733.3	7.4	1608
IYRAQLELF	9	0.0197	FAM126B	ENSG00000155744.9	ENSP00000393667.2	6.3	1590
EYPKFLKRF	9	0.0262	COL4A3BP	ENSG00000113163.16	ENSP00000369862.5	16.3	1572
SQRLARLV	8	31.1667	IDO1	ENSG00000131203.13	ENSP00000430950.1	7.3	1209
RYMPPAHRNF	10	0.0021	IDO1	ENSG00000131203.13	ENSP00000430950.1	7.3	1209
RYAEAVQLL	9	0.0058	TTC30A	ENSG00000197557.7	ENSP00000347915.4	4.6	1995
VYVAGAPRF	9	0.0070	ITGA11	ENSG00000137809.17	ENSP00000403392.2	6.6	3567
KMPKFKMPSF	10	0.4228	AHNAK2	ENSG00000185567.7	ENSP00000353114.4	7.1	17385
VYTLLTTHL	9	0.0290	C11orf80	ENSG00000173715.16	ENSP00000494645.1	16.2	1731
KYVKVFHQF	9	0.0007	ZNF486	ENSG00000256229.8	ENSP00000335042.7	4.8	1389
IYTFPHASF	9	0.0101	BTN3A3	ENSG00000111801.16	ENSP00000355238.3	59.7	1605
VWSDVTPLTF	10	0.0205	MMP11	ENSG00000099953.10	ENSP00000408070.1	21.8	1062
VYKTIMEQF	9	0.0045	BAIAP2	ENSG00000175866.15	ENSP00000315685.7	26.0	1563
KYGVFEESL	9	0.2812	TRIM34	ENSG00000258659.6	ENSP00000402595.2	11.2	1464
EYNTIKDKF	9	0.0842	PARP15	ENSG00000173200.13	ENSP00000417214.2	5.9	2034

EYLRQIFRL	9	0.0536	SMPD4	ENSG00000136699.19	ENSP00000401648.1	54.3	2223
SYIQRLVQI	9	0.0142	SAMHD1	ENSG00000101347.9	ENSP00000493536.1	29.9	1878
NYIEKVVAI	9	0.0630	ABI1	ENSG00000136754.17	ENSP00000365308.3	26.5	1356
KYFDNSQSL	9	0.0152	TTC17	ENSG00000052841.15	ENSP00000299240.5	70.0	2868
QYAVLLNRF	9	0.0115	NSUN3	ENSG00000178694.10	ENSP00000318986.4	7.0	1020
VYLDGIVRI	9	0.0109	KIAA0895L	ENSG00000196123.13	ENSP00000290881.7	57.0	1413
HYAALAHYF	9	0.0053	RHPN2	ENSG00000131941.8	ENSP00000254260.2	24.4	2058
RYFDRAALF	9	0.0038	WDR11	ENSG00000120008.16	ENSP00000263461.5	49.4	3672
QALEVLKI	8	18.3684	MOCS3	ENSG00000124217.4	ENSP00000244051.1	4.2	1380
PFAKPLPTF	9	0.0240	EP400	ENSG00000183495.13	ENSP00000374212.2	36.5	9369
KYAALFSQM	9	0.0872	SAMD4B	ENSG00000179134.15	ENSP00000470237.1	72.5	2076
KYFDKVVTL	9	0.0039	KIF7	ENSG00000166813.15	ENSP00000377934.3	4.7	4029
RYLDASNEL	9	0.0559	NEK4	ENSG00000114904.13	ENSP00000233027.5	9.9	2523
RYQTKFRHL	9	0.0887	CEP89	ENSG00000121289.18	ENSP00000465141.1	22.9	1284
RYTEITREKF	10	0.0135	CEP89	ENSG00000121289.18	ENSP00000465141.1	22.9	1284
VYSEKVMHMF	10	0.0071	MYO18A	ENSG00000196535.16	ENSP00000437073.1	41.3	6162
HVVGQGVLHR	10	35.0000	OGFOD2	ENSG00000111325.16	ENSP00000442817.1	20.1	558
EYIPKWEQF	9	0.0029	C3orf14	ENSG00000114405.10	ENSP00000232519.5	8.7	384
IYTKLNQKI	9	0.0210	EXOC6	ENSG00000138190.16	ENSP00000260762.6	6.0	2412
RLFQSVKLH	9	19.8387	POT1	ENSG00000128513.16	ENSP00000377002.1	11.6	1509
KYPHYFPVM	9	0.0445	NLN	ENSG00000123213.23	ENSP00000423214.1	5.4	1800
VYQNHVQHL	9	0.0069	ARHGAP5	ENSG00000100852.13	ENSP00000371897.1	22.6	4506
RYLADLPTL	9	0.0275	CEP85	ENSG00000130695.15	ENSP00000391790.1	9.1	1305
KYTAVVKMI	9	0.1167	KIN	ENSG00000151657.12	ENSP00000368881.3	5.3	1179
EVIGRLDTM	9	6.7761	ADH1B	ENSG00000196616.14	ENSP00000306606.8	21.6	1125
IYKIDFVRF	9	0.0145	UNC119	ENSG00000109103.11	ENSP00000337040.3	15.8	720
TYARNLPAF	9	0.0080	TTI2	ENSG00000129696.12	ENSP00000411169.2	9.4	1524

EIMEVAKER	9	26.0000	ESS2	ENSG00000100056.12	ENSP00000252137.6	16.2	1428
RYTERVDYL	9	0.0795	USP13	ENSG00000058056.9	ENSP00000263966.3	4.1	2589
KYLKVFYKF	9	0.0014	ZNF254	ENSG00000213096.10	ENSP00000349494.3	4.8	1977
SFTQKSHLF	9	0.0377	ZNF175	ENSG00000105497.8	ENSP00000262259.2	5.0	2133
SYVHKVPSF	9	0.0040	MED13L	ENSG00000123066.8	ENSP00000281928.3	30.3	6630
KYNANFVTF	9	0.0016	KDM2A	ENSG00000173120.15	ENSP00000432786.1	67.4	3486
VYEYVVERF	9	0.0088	TUT4	ENSG00000134744.14	ENSP00000257177.4	32.7	4935
SIVDSILMER	10	27.5000	RBM25	ENSG00000119707.14	ENSP00000261973.7	44.7	2529
RYFDVGLHNF	10	0.0031	HHAT	ENSG00000054392.13	ENSP00000261458.3	6.3	1479
EYVEKFYRI	9	0.0259	RAD50	ENSG00000113522.14	ENSP00000368100.4	11.1	3936
KYLTQHERI	9	0.0738	ZNF473	ENSG00000142528.16	ENSP00000375697.1	4.6	2613
SYNPAENAVLL	11	0.2088	COPA	ENSG00000122218.16	ENSP00000497433.1	91.4	1365
KYLADLPTL	9	0.0317	CEP85L	ENSG00000111860.14	ENSP00000357477.3	4.3	2415
VYFPDQHINF	10	0.0037	PARD3B	ENSG00000116117.18	ENSP00000484434.1	5.6	3207
RYKEENNDHL	10	0.4608	USP8	ENSG00000138592.14	ENSP00000412682.3	26.6	3036
SYIEVSHGL	9	0.0316	SYNPO2	ENSG00000172403.11	ENSP00000425496.1	11.5	3465
VMPKKVFKI	9	0.1848	XYLT1	ENSG00000103489.11	ENSP00000261381.6	3.7	2877
SQLRTTVV	8	23.1875	MTSS1	ENSG00000170873.18	ENSP00000367256.3	74.4	2190
RYAEQISVL	9	0.0454	CCDC121	ENSG00000176714.9	ENSP00000339087.2	5.0	834
KYLTVKDYL	9	0.0991	ATAD2	ENSG00000156802.13	ENSP00000287394.5	6.5	4170
AYAIIKEEL	9	0.1534	ATAD2	ENSG00000156802.13	ENSP00000287394.5	6.5	4170
VYSPLGSAYKL	11	0.0942	SLC38A9	ENSG00000177058.12	ENSP00000380074.2	7.9	1683
IFHEKTSEF	9	0.0529	SLC7A2	ENSG00000003989.17	ENSP00000419140.1	4.2	1974
RYPDTIALTF	10	0.0026	MAPKBP1	ENSG00000137802.14	ENSP00000397570.2	12.9	4524
VYNDHSIYVW	10	0.0502	MAPKBP1	ENSG00000137802.14	ENSP00000397570.2	12.9	4524
KYVSSVLSF	9	0.0043	LTN1	ENSG00000198862.14	ENSP00000478783.1	11.8	5436
KYLDVKFEYL	10	0.0921	PICK1	ENSG00000100151.16	ENSP00000385205.3	42.7	1245

SYISRTNQL	9	0.0234	AHCTF1	ENSG00000153207.15	ENSP00000355464.1	13.3	6903
EYDLIVHQL	9	0.1080	VPS26A	ENSG00000122958.15	ENSP00000263559.6	28.8	981
EVSWKVLER	9	21.6364	PHKB	ENSG00000102893.16	ENSP00000313504.5	41.4	3279
QYVDFHNQL	9	0.0552	ERCC5	ENSG00000134899.21	ENSP00000498881.1	57.0	3558
IFIQNSKLYF	10	0.1828	SLC35A5	ENSG00000138459.9	ENSP00000417654.1	6.9	1272
KYIDFDHVF	9	0.0027	CRAMP1	ENSG00000007545.15	ENSP00000293925.5	10.1	3807
TFTFSHATF	9	0.0994	TRIM27	ENSG00000204713.11	ENSP00000366404.3	25.2	1539
DTASIFSLK	9	24.9286	CDK18	ENSG00000117266.15	ENSP00000353176.2	255.2	1422
KYQEYTNEL	9	0.0277	TBK1	ENSG00000183735.10	ENSP00000498341.1	19.7	2193
IYKDSSTFL	9	0.0775	METTL14	ENSG00000145388.15	ENSP00000373474.3	7.4	1368
AQRSFRVSI	9	5.8930	VPS13C	ENSG00000129003.18	ENSP00000249837.3	32.5	11130
TYISWKEEL	9	0.0517	VPS13C	ENSG00000129003.18	ENSP00000249837.3	32.5	11130
IYHPDIQLL	9	0.0039	VPS13C	ENSG00000129003.18	ENSP00000249837.3	32.5	11130
SYVAGLPRF	9	0.0069	KLHL36	ENSG00000135686.13	ENSP00000456743.1	38.0	1848
RYPGKFYRV	9	0.0511	MAP3K20	ENSG00000091436.17	ENSP00000364361.3	15.7	2400
AWPKVLKI	8	1.0777	EPB41L3	ENSG00000082397.17	ENSP00000441174.1	31.1	2649
LYIANVAHF	9	0.0047	SLC37A2	ENSG00000134955.11	ENSP00000384407.2	26.6	1503
LYHNNNQFF	9	0.0149	NFATC1	ENSG00000131196.17	ENSP00000316553.5	9.5	2436
KYIENLDNITF	11	0.0112	ZFYVE16	ENSG00000039319.17	ENSP00000423663.1	13.5	4617
VYERELQTF	9	0.0106	EEF1AKMT2	ENSG00000203791.15	ENSP00000357829.2	7.3	873
YYPAKIEAI	9	0.0564	PHF20L1	ENSG00000129292.20	ENSP00000378788.2	19.5	2976
RYKPGEPITF	10	0.0074	DENND1B	ENSG00000213047.13	ENSP00000479816.1	4.3	2325
YLEKAIKI	8	2.3986	USP7	ENSG00000187555.15	ENSP00000343535.4	53.6	3306
TYLEKAIKI	9	0.0206	USP7	ENSG00000187555.15	ENSP00000343535.4	53.6	3306
AYLDKSPQF	9	0.0061	DHX29	ENSG00000067248.10	ENSP00000481966.1	16.5	4110
VYMELSQKL	9	0.0043	FRMD6	ENSG00000139926.15	ENSP00000379068.2	7.5	1842
KYIKEAVRL	9	0.0236	FRMD6	ENSG00000139926.15	ENSP00000379068.2	7.5	1842

LYQKDFLRI	9	0.0559	FRMD6	ENSG00000139926.15	ENSP00000379068.2	7.5	1842
IYPTAPPRF	9	0.0031	LYPLAL1	ENSG00000143353.12	ENSP00000355894.3	15.5	663
RYAAQLPAL	9	0.1300	PELP1	ENSG00000141456.15	ENSP00000460300.2	20.0	3390
IYEQVFAKL	9	0.0513	NRDE2	ENSG00000119720.18	ENSP00000346335.3	7.1	3492
KYMDVVKERI	10	0.1639	DDX24	ENSG00000089737.17	ENSP00000481495.1	34.6	2577
QYLTSVQQL	9	0.0226	CEP97	ENSG00000182504.11	ENSP00000342510.3	3.1	2595
IYIRHSNLM	9	0.0891	EPB41	ENSG00000159023.21	ENSP00000348397.3	27.4	1866
SYLNSVQRL	9	0.0162	NUPL2	ENSG00000136243.17	ENSP00000415511.1	20.8	672
TYLDKIKKF	9	0.0058	SYNE2	ENSG00000054654.16	ENSP00000341781.4	100.6	20655
TYVENLRLL	9	0.0305	SYNE2	ENSG00000054654.16	ENSP00000341781.4	100.6	20655
VYINTAQEF	9	0.0023	DHPS	ENSG00000095059.16	ENSP00000482709.1	68.5	1110
RYLSKVLEL	9	0.0106	C15orf40	ENSG00000169609.14	ENSP00000307071.6	14.4	459
AVIEQEVRER	10	35.7500	CC2D2A	ENSG00000048342.16	ENSP00000421809.1	21.9	4860
RYLTVATVF	9	0.0105	TUBB6	ENSG00000176014.12	ENSP00000318697.4	33.3	1338
IYKAPSENW	9	0.0213	DNMT1	ENSG00000130816.16	ENSP00000345739.3	54.9	4848
VFKEKHHSW	9	0.1530	RNASEH2B	ENSG00000136104.20	ENSP00000495755.1	14.1	867
IFLNKTFQW	9	0.0262	ALG10B	ENSG00000175548.9	ENSP00000310120.4	2.9	1419
RYLETILEI	9	0.0123	NAIP	ENSG00000249437.7	ENSP00000429545.1	15.1	4041
EVVNYVQKR	9	28.0000	RMI1	ENSG00000178966.16	ENSP00000317039.3	7.5	1875
EYSKYLQQAF	10	0.1837	ZNF281	ENSG00000162702.8	ENSP00000356322.1	3.3	2685
RMYEKALQI	9	0.7523	TTC19	ENSG00000011295.16	ENSP00000261647.5	14.1	1140
KYLTQAQLVQF	11	0.0526	ELOVL2	ENSG00000197977.4	ENSP00000346693.3	2.5	888
RYMEHLEKI	9	0.0075	KIAA0895	ENSG00000164542.12	ENSP00000319251.6	4.1	1407
TYHPGVPVF	9	0.0025	CHORDC1	ENSG00000110172.12	ENSP00000319255.6	11.3	996
GYPEVALHF	9	0.0050	COPA	ENSG00000122218.16	ENSP00000357048.3	91.4	3699
IYLRDPVQV	9	0.1470	TPP2	ENSG00000134900.12	ENSP00000498955.1	24.3	3519
QVYGLPDL	8	30.6667	KLHL42	ENSG00000087448.11	ENSP00000370671.2	5.6	1515

SYRNSLDSF	9	0.0814	ZNF326	ENSG00000162664.17	ENSP00000340796.4	7.4	1746
KYALLLQEL	9	0.0286	PLEKHG4	ENSG00000196155.13	ENSP00000398030.1	13.8	3330
IQMSVQRI	8	6.7323	POLR2B	ENSG00000047315.16	ENSP00000312735.5	55.0	3522
EVIEMVRKR	9	23.6875	ROR1	ENSG00000185483.12	ENSP00000441637.2	3.0	2646
RYLNKSFVL	9	0.0373	ANKEF1	ENSG00000132623.16	ENSP00000367631.3	2.4	2328
SFSTVHEKF	9	0.0219	WDR36	ENSG00000134987.11	ENSP00000479950.1	5.3	2853
AYLTVHQRI	9	0.0237	ZNF570	ENSG00000171827.10	ENSP00000331540.1	3.0	1608
RYMNHMQSL	9	0.0187	ARIH1	ENSG00000166233.15	ENSP00000369217.4	16.2	1671
RYFDGNLEKL	10	0.0944	ARIH1	ENSG00000166233.15	ENSP00000369217.4	16.2	1671
RYMNHMQSLRF	11	0.0475	ARIH1	ENSG00000166233.15	ENSP00000369217.4	16.2	1671
KYLSGLVQW	9	0.0113	EMC3	ENSG00000125037.12	ENSP00000408803.1	28.2	609
LYKDVRDLL	9	0.1675	ANKZF1	ENSG00000163516.13	ENSP00000386337.3	66.4	2178
VYSLIKNKI	9	0.0528	PRKAG2	ENSG00000106617.14	ENSP00000287878.3	20.9	1707
VYILQPEVI	9	0.0364	CPLANE1	ENSG00000197603.14	ENSP00000498265.1	9.9	6852
SQYPQPKL	8	5.3959	GBGT1	ENSG00000148288.13	ENSP00000493832.1	10.9	480
KYMEDYHQF	9	0.0005	PPL	ENSG00000118898.16	ENSP00000340510.2	3.6	5268
VLPQRFPQF	9	0.0393	CNOT4	ENSG00000080802.18	ENSP00000399108.2	11.9	1716
EVIGLLGGRY	10	39.0000	MYSM1	ENSG00000162601.11	ENSP00000478391.1	13.4	702
KYQRILERL	9	0.0301	OPA1	ENSG00000198836.10	ENSP00000354681.3	14.7	2991
VFVDSVPEF	9	0.0235	INTS9	ENSG00000104299.15	ENSP00000429065.1	14.5	1974
RYTTEFHEL	9	0.0150	WEE1	ENSG00000166483.11	ENSP00000402084.2	5.3	1938
EFVAKGHLF	9	0.1996	MED18	ENSG00000130772.14	ENSP00000362948.4	4.0	624
SYQKVIELF	9	0.0009	PBK	ENSG00000168078.10	ENSP00000301905.4	2.3	966
TYAEKLHRL	9	0.0099	DST	ENSG00000151914.20	ENSP00000354508.3	33.1	22383
IYTRLLPAL	9	0.1740	ACY1	ENSG00000243989.9	ENSP00000419262.1	25.5	1029
RYQEITKLQLF	11	0.0053	CCNDBP1	ENSG00000166946.14	ENSP00000456041.1	61.1	426
QQRLVVVSV	9	9.1359	CIAO3	ENSG00000103245.14	ENSP00000457058.1	55.9	1122

KYVTVYNLI	9	0.0238	USP8	ENSG00000138592.14	ENSP00000302239.4	26.6	3354
KYLLTPVNF	9	0.0028	DCLRE1B	ENSG00000118655.7	ENSP00000498042.1	3.7	1596
EYLKHHNRI	9	0.1628	GZF1	ENSG00000125812.16	ENSP00000366250.2	4.4	2133
RYNPNLNTW	9	0.0060	KLHL11	ENSG00000178502.6	ENSP00000314608.3	1.4	2124
KYTEITATYF	10	0.0092	AGL	ENSG00000162688.17	ENSP00000355106.3	5.2	4596
SYLAIHQRI	9	0.0129	ZNF665	ENSG00000197497.11	ENSP00000379702.2	5.9	2034
KWFDNSNMTF	10	0.0348	CD302	ENSG00000241399.7	ENSP00000451446.2	12.8	585
IYQDVTLKW	9	0.0040	BBS9	ENSG00000122507.20	ENSP00000242067.6	12.7	2661
RYQEMIQKL	9	0.0039	SCLT1	ENSG00000151466.12	ENSP00000281142.5	7.0	2064
SFITHSLKF	9	0.0430	CEP192	ENSG00000101639.18	ENSP00000427550.1	11.3	7611
HYINMPVQF	9	0.0032	CEP192	ENSG00000101639.18	ENSP00000427550.1	11.3	7611
IYVTGGHLF	9	0.0043	KLHDC7B	ENSG00000130487.8	ENSP00000379034.2	1.6	1782
KYGNFIDKL	9	0.0902	GTPBP10	ENSG00000105793.15	ENSP00000222511.6	3.0	1161
RVQDKYFHI	9	0.8341	ABLIM2	ENSG00000163995.20	ENSP00000421283.1	5.0	1677
VYIDKVRSL	9	0.0199	LMNB1	ENSG00000113368.12	ENSP00000378761.1	20.1	1161
SYANVQDGF	9	0.0646	ANKRD22	ENSG00000152766.6	ENSP00000360998.4	1.3	573
GYKAIHEYL	9	0.2256	SYNE1	ENSG00000131018.23	ENSP00000356224.5	43.4	26391
RYVDRVTEF	9	0.0042	CDK5RAP3	ENSG00000108465.15	ENSP00000464286.1	43.6	816
KYMETIEKL	9	0.0049	PPP1R21	ENSG00000162869.16	ENSP00000415696.2	26.9	2214
KYGVVIASF	9	0.0050	DOCK4	ENSG00000128512.22	ENSP00000404179.1	36.8	5898
LYTVNLHRI	9	0.1289	SGSM1	ENSG00000167037.18	ENSP00000383211.4	3.4	3279
LYEEINQVF	9	0.0325	CYP20A1	ENSG00000119004.15	ENSP00000348380.4	8.2	1386
IYPPKLHQF	9	0.0012	CCNE1	ENSG00000105173.14	ENSP00000262643.3	3.0	1230
NQIILNKV	8	21.5000	LIN54	ENSG00000189308.11	ENSP00000341947.3	7.0	2247
YYIFIPSKF	9	0.0026	DDX47	ENSG00000213782.7	ENSP00000319578.6	39.5	1218
KYVKDFHKF	9	0.0014	ZNF724	ENSG00000196081.9	ENSP00000413411.1	1.1	1857
VYVSRPSHF	9	0.0058	STAT2	ENSG00000170581.14	ENSP00000450751.1	78.3	2541

PYTTSTPNI	9	0.2942	DOCK9	ENSG00000088387.19	ENSP00000401958.4	49.5	6243
KYTHFIQSF	9	0.0021	GBGT1	ENSG00000148288.13	ENSP00000361110.3	10.9	1041
ETISQHIAKR	10	41.5000	GBGT1	ENSG00000148288.13	ENSP00000361110.3	10.9	1041
DVAQEHLKER	10	53.7500	GLI2	ENSG00000074047.21	ENSP00000354586.4	1.8	4758
FYSRLLQKF	9	0.0046	ZWILCH	ENSG00000174442.12	ENSP00000311429.5	3.3	1773
DIIEKVMAR	9	19.8065	TSC2	ENSG00000103197.18	ENSP00000496611.1	87.8	5307
NYTLVSHLI	9	0.0455	MARS	ENSG00000166986.15	ENSP00000262027.5	71.0	2700
KYLQSTISF	9	0.0031	TOP3B	ENSG00000100038.19	ENSP00000381773.2	22.2	2586
DLFKAVDAALKK	12	43.5000	KNG1	ENSG00000113889.14	ENSP00000493985.1	1.5	1932
AYANIISQF	9	0.0035	SLC47A2	ENSG00000180638.17	ENSP00000338084.6	1.2	1740
KYVKVFDKF	9	0.0034	ZNF107	ENSG00000196247.11	ENSP00000378789.1	1.4	2349
LYLRQTETL	9	0.0286	ZBTB20	ENSG00000181722.16	ENSP00000349803.3	2.9	2004
KYITEVSRW	9	0.0214	CEP135	ENSG00000174799.10	ENSP00000257287.3	8.3	3420
EVFTGGIGSY	10	15.8116	TENT4B	ENSG00000121274.12	ENSP00000350054.4	7.2	909
TPLTSMVVTKPD	12	100.0000	ITIH4	ENSG00000055955.16	ENSP00000266041.4	8.1	2790
KYGDILHVI	9	0.0077	DLG2	ENSG00000150672.17	ENSP00000381355.2	1.4	2610
DIIAKVTRR	9	26.2000	ARHGAP27	ENSG00000159314.11	ENSP00000437100.1	63.4	642
TYLRKIPRF	9	0.0044	CCDC14	ENSG00000175455.15	ENSP00000420180.3	22.1	2736
KYVKVMHKF	9	0.0016	ZNF138	ENSG00000197008.9	ENSP00000399528.1	6.3	861
KFFRVLNSYKI	11	2.2546	CHD5	ENSG00000116254.18	ENSP00000262450.3	0.2	5862
KYWTEPEKF	9	0.0062	CYP3A7	ENSG00000160870.14	ENSP00000479282.1	1.6	1509
FLAEGGGVR	9	36.3333	FGA	ENSG00000171560.16	ENSP00000385981.3	0.1	1932
FTSSTSYNR	9	17.6667	FGA	ENSG00000171560.16	ENSP00000385981.3	0.1	1932
KYGKVWGF	8	0.2160	CYP3A4	ENSG00000160868.15	ENSP00000498939.1	0.1	1509
EYQKVWNLF	9	0.0077	PARP12	ENSG00000059378.12	ENSP00000263549.3	57.7	2103
KHNYELDFKAF	11	1.3328	PARP12	ENSG00000059378.12	ENSP00000263549.3	57.7	2103
YYEHVKARF	9	0.0315	MLPH	ENSG00000115648.14	ENSP00000264605.3	3.9	1800

%rank, %rank score of NetMHCpan4.1; Expression, gene expression calculated using RNA-seq data; ORF length, nucleotide lengths of the open reading frame encoding the peptide

Supplementary table 2: HLA-A24 ligands detected in the RCC17 normal tissue

Sequence	Length	%rank	Gene	Ensembl gene ID	Ensembl protein ID	Expression	ORF Length
EYFESPFTI	9	0.0215	MT-CO3	ENSG00000198938.2	ENSP00000354982.2	41345.3	783
YHLPPSPKPMK	11	7.9334	MT-ATP8	ENSG00000228253.1	ENSP00000355265.1	16160.4	204
ESVGTMGNR	9	52.5000	ALDOB	ENSG00000136872.20	ENSP00000497990.1	5531.6	1092
TIQGLDGLSER	11	42.5000	ALDOB	ENSG00000136872.20	ENSP00000497990.1	5531.6	1092
RMPEFYNRF	9	0.0212	ASS1	ENSG00000130707.17	ENSP00000253004.6	2606.0	1236
QYAGKYVLF	9	0.0031	GPX3	ENSG00000211445.12	ENSP00000484258.1	4704.2	675
AYLPVNESF	9	0.0029	EEF2	ENSG00000167658.16	ENSP00000307940.5	1569.4	2574
RYFDPANGKF	10	0.0144	EEF2	ENSG00000167658.16	ENSP00000307940.5	1569.4	2574
LYLKVKGNVF	10	0.1685	RPL19	ENSG00000108298.11	ENSP00000464538.1	1658.7	582
AYVLNIVRF	9	0.0134	ATP1B1	ENSG00000143153.12	ENSP00000356789.3	1688.5	909
KFIDTTSKF	9	0.0212	RPL3	ENSG00000100316.16	ENSP00000346001.3	1705.2	1209
KYCQVIRVI	9	0.1831	RPL3	ENSG00000100316.16	ENSP00000346001.3	1705.2	1209
DVIGVTKGK	9	25.0000	RPL3	ENSG00000100316.16	ENSP00000346001.3	1705.2	1209
RYSSMAASF	9	0.0077	PDZK1IP1	ENSG00000162366.8	ENSP00000294338.2	1488.6	342
YENVPEEEGK	10	60.0000	PDZK1IP1	ENSG00000162366.8	ENSP00000294338.2	1488.6	342
YENVPEEEGKVR	12	70.0000	PDZK1IP1	ENSG00000162366.8	ENSP00000294338.2	1488.6	342
YVHMVTHF	8	0.8009	TMBIM6	ENSG00000139644.13	ENSP00000447030.1	1600.8	600
EHGDQDYIW	9	2.7597	TMBIM6	ENSG00000139644.13	ENSP00000447030.1	1600.8	600
HYIRKYNRF	9	0.0164	RPS11	ENSG00000142534.7	ENSP00000270625.1	1686.6	474
VQNGKHFKF	9	0.2918	FABP1	ENSG00000163586.9	ENSP00000295834.3	1063.0	381
KWFFQKLRF	9	0.0894	RPL27	ENSG00000131469.14	ENSP00000253788.4	1066.5	408
KYQAVTATL	9	0.0175	RPL13A	ENSG00000142541.17	ENSP00000375730.4	1635.9	609
RLPEEWSQW	9	0.2153	HSPB1	ENSG00000106211.9	ENSP00000248553.6	1075.6	615

DVIAQGIGKL	10	28.1250	RPLP2	ENSG00000177600.9	ENSP00000322419.4	952.9	345
IYNEALKG	8	19.8387	S100A6	ENSG00000197956.10	ENSP00000357708.3	1100.2	270
NRPIPQWIR	9	11.2826	RPL39	ENSG00000198918.8	ENSP00000355315.3	892.7	153
SQLRVAKV	8	21.5455	RPL35	ENSG00000136942.15	ENSP00000259469.4	884.6	369
IIEVDPDTKE	10	80.0000	RPS17	ENSG00000182774.13	ENSP00000498019.1	849.6	405
VYGPMKNYL	9	0.0244	ANPEP	ENSG00000166825.14	ENSP00000300060.6	894.0	2901
QQSKILKV	8	18.0526	HSP90AB1	ENSG00000096384.20	ENSP00000360709.5	719.0	2172
EIAEAYLGY	9	16.4000	HSPA1B	ENSG00000204388.7	ENSP00000364801.3	675.4	1923
TVFDAKRLIGR	11	27.3000	HSPA1B	ENSG00000204388.7	ENSP00000364801.3	675.4	1923
DVIPTDIHQR	10	26.7000	FBP1	ENSG00000165140.10	ENSP00000364475.4	657.8	1014
EVTRVKAVR	9	37.6667	TXNIP	ENSG00000265972.5	ENSP00000462521.1	654.2	1173
SMRYFYTSV	9	12.8611	HLA-B	ENSG00000234745.11	ENSP00000399168.2	906.6	1086
VMAPRTVLL	9	0.4689	HLA-B	ENSG00000234745.11	ENSP00000399168.2	906.6	1086
GQFKTYAI	8	16.6727	RPS21	ENSG00000171858.18	ENSP00000345957.3	837.7	249
VMAPRTLLL	9	0.3778	HLA-A	ENSG00000206503.13	ENSP00000366005.5	698.8	1095
RFKEANNFL	9	0.3820	RPL7	ENSG00000147604.14	ENSP00000339795.2	603.2	744
DFFRDEAERIMR	12	36.3333	DPEP1	ENSG00000015413.9	ENSP00000261615.4	1230.0	1233
LYWSHPRKF	9	0.0225	RPS29	ENSG00000213741.10	ENSP00000245458.6	590.7	168
EYMYQVMKF	9	0.0145	MIOX	ENSG00000100253.13	ENSP00000216075.6	1212.8	855
MYQVMKFNKF	10	0.1675	MIOX	ENSG00000100253.13	ENSP00000216075.6	1212.8	855
DIIQRMHLR	9	17.0667	GNAS	ENSG00000087460.25	ENSP00000499392.1	720.5	960
SYTSHTNEI	9	0.1620	C7	ENSG00000112936.18	ENSP00000322061.9	506.5	2529
DVKFGADARA	10	80.0000	HSPD1	ENSG00000144381.17	ENSP00000340019.2	459.7	1719
AKDVKFGADARA	12	90.0000	HSPD1	ENSG00000144381.17	ENSP00000340019.2	459.7	1719
DINSRVEMKPK	11	80.0000	AQP1	ENSG00000240583.12	ENSP00000498806.1	639.9	597
KYNDTFWKEF	10	0.0157	HSP90B1	ENSG00000166598.15	ENSP00000299767.4	433.1	2409
VQVTVPKI	8	11.5435	A2M	ENSG00000175899.14	ENSP00000323929.7	384.5	4422

IYKPGQTVKF	10	0.0098	A2M	ENSG00000175899.14	ENSP00000323929.7	384.5	4422
ELCDERVSSR	10	30.8333	UQCRH	ENSG00000173660.12	ENSP00000309565.5	489.0	273
VYVYGVDRF	9	0.0215	SLC6A19	ENSG00000174358.16	ENSP00000305302.10	359.8	1902
SVIGFRATQR	10	25.6667	SLC6A19	ENSG00000174358.16	ENSP00000305302.10	359.8	1902
KYFSLPSVVF	10	0.0231	UGT1A9	ENSG00000241119.2	ENSP00000346768.4	353.0	1590
RYDEHAYKW	9	0.0111	BBOX1	ENSG00000129151.9	ENSP00000433772.1	359.9	1161
SYTNVYRMF	9	0.0100	GLYATL1	ENSG00000166840.13	ENSP00000479741.1	550.9	906
RWPKKSAEF	9	0.0535	RPL17	ENSG00000265681.7	ENSP00000462385.1	533.9	552
GYNVKFDMF	9	0.1275	GPX4	ENSG00000167468.16	ENSP00000483655.1	527.7	510
VVRHQLLKT	9	55.0000	COX7C	ENSG00000127184.13	ENSP00000247655.3	388.0	189
LVVRHQLLKT	10	80.0000	COX7C	ENSG00000127184.13	ENSP00000247655.3	388.0	189
NYKKFYEQF	9	0.0105	HSP90AA1	ENSG00000080824.18	ENSP00000335153.7	651.8	2562
YIAHPKLGKR	10	36.6667	RPL29	ENSG00000162244.11	ENSP00000294189.4	340.2	477
KYPDAVATW	9	0.0027	SPP1	ENSG00000118785.14	ENSP00000378517.3	2172.3	942
HVIGLQMGSNR	11	44.0000	TAGLN	ENSG00000149591.16	ENSP00000376678.4	341.5	603
TGYGRPRQIIS	11	27.4000	TAGLN	ENSG00000149591.16	ENSP00000376678.4	341.5	603
AYKYIQELW	9	0.0402	RPL15	ENSG00000174748.21	ENSP00000483260.1	521.3	612
HSYSPRAI	8	23.0000	PSMB4	ENSG00000159377.11	ENSP00000290541.6	257.0	792
ELYDGPRQMGR	11	17.2222	CUBN	ENSG00000107611.15	ENSP00000367064.4	264.6	10869
KYTPPPHHI	9	0.0036	NDUFS5	ENSG00000168653.11	ENSP00000362058.3	423.4	318
QQLTAMKV	8	32.6000	MYH9	ENSG00000100345.21	ENSP00000216181.5	239.8	5880
KYLYVDKNF	9	0.0370	MYH9	ENSG00000100345.21	ENSP00000216181.5	239.8	5880
HGLTVPLI	8	19.3871	ATP6V0E1	ENSG00000113732.9	ENSP00000429690.1	238.2	243
VYPVALVSF	9	0.0052	CLCNKB	ENSG00000184908.17	ENSP00000364831.4	284.2	2061
QQYRALTV	8	18.3421	TUBB4B	ENSG00000188229.6	ENSP00000341289.4	240.2	1335
RYLTVAAVF	9	0.0167	TUBB4B	ENSG00000188229.6	ENSP00000341289.4	240.2	1335
EYPDRIMNTF	10	0.0274	TUBB4B	ENSG00000188229.6	ENSP00000341289.4	240.2	1335

TQFKWIHI	8	5.9304	KHK	ENSG00000138030.13	ENSP00000260598.5	308.6	894
HYEMLFNSF	9	0.0781	PTH1R	ENSG00000160801.14	ENSP00000402723.1	387.0	1779
DYVEGLRVF	9	0.1353	MYL6	ENSG00000092841.18	ENSP00000446955.1	721.7	453
ETIKGIQKR	9	21.4545	ITM2B	ENSG00000136156.15	ENSP00000497221.1	384.3	798
TYLPQSYLI	9	0.0033	ITM2B	ENSG00000136156.15	ENSP00000497221.1	384.3	798
EVMKAVKVGM	10	29.5714	PEPD	ENSG00000124299.15	ENSP00000498922.1	724.7	1566
RYCTDTGVLF	10	0.0351	PEPD	ENSG00000124299.15	ENSP00000498922.1	724.7	1566
VFMPHGLGHFL	11	0.4524	PEPD	ENSG00000124299.15	ENSP00000498922.1	724.7	1566
TYLPAGQSVLL	11	0.0752	PHB	ENSG00000167085.11	ENSP00000300408.3	228.5	816
RYDPYTQRI	9	0.0229	PAH	ENSG00000171759.10	ENSP00000448059.1	449.3	1356
EYNHIFPLL	9	0.0466	PAH	ENSG00000171759.10	ENSP00000448059.1	449.3	1356
KYRVDFHNF	9	0.0277	KCNJ1	ENSG00000151704.15	ENSP00000376432.2	323.3	1173
SYMGHFDLL	9	0.0245	SF3B5	ENSG00000169976.6	ENSP00000356541.2	212.0	258
QYAIISEAW	9	0.0589	CRYL1	ENSG00000165475.15	ENSP00000493784.1	433.2	795
VMSILPKI	8	9.9324	SERINC1	ENSG00000111897.7	ENSP00000342962.3	208.6	1359
NYIDKVRFL	9	0.0729	VIM	ENSG00000026025.16	ENSP00000224237.5	389.1	1398
RISLPLPNF	9	0.8058	VIM	ENSG00000026025.16	ENSP00000224237.5	389.1	1398
KYMTAVVKLF	10	0.0213	CYBA	ENSG00000051523.11	ENSP00000261623.3	224.8	585
ELIQDITQR	9	20.9630	MSN	ENSG00000147065.17	ENSP00000353408.5	204.7	1731
KYGDFNKEV	9	0.7009	MSN	ENSG00000147065.17	ENSP00000353408.5	204.7	1731
EYALLYHTL	9	0.0333	AOC1	ENSG00000002726.20	ENSP00000411613.2	421.4	2310
EIINEDIAKR	10	49.0000	PDIA6	ENSG00000143870.12	ENSP00000272227.3	202.1	1320
SYANVKQWL	9	0.1450	RAB1B	ENSG00000174903.16	ENSP00000310226.6	208.0	603
ETIGEILKK	9	20.2222	HNRNPK	ENSG00000165119.21	ENSP00000317788.4	327.4	1389
AQYLLQNSV	9	8.6807	HNRNPK	ENSG00000165119.21	ENSP00000317788.4	327.4	1389
RYFVSEGFEF	10	0.0306	GOT1	ENSG00000120053.12	ENSP00000359539.5	194.5	1239
LYLHRQWLL	9	0.0501	CYP4A11	ENSG00000187048.13	ENSP00000311095.4	266.1	1557

EIAYSDVAKR	10	49.0000	CLIC4	ENSG00000169504.15	ENSP00000363500.4	203.6	759
IAYSDVAKRLTK	12	28.8750	CLIC4	ENSG00000169504.15	ENSP00000363500.4	203.6	759
TYSAGHNLL	9	0.1310	KL	ENSG00000133116.8	ENSP00000369442.3	192.4	3036
AYLLTLGKF	9	0.0760	CYFIP2	ENSG00000055163.20	ENSP00000479968.1	250.0	3759
HQQVVSRI	8	15.9855	SLC9A3R1	ENSG00000109062.12	ENSP00000262613.5	189.4	1074
EVIEIMTDR	9	31.5000	HNRNPA1	ENSG00000135486.17	ENSP00000341826.6	367.5	1116
AYFQKPLLL	9	0.0125	AGXT2	ENSG00000113492.14	ENSP00000231420.6	230.9	1542
IYYFKANVF	9	0.0169	ARPC3	ENSG00000111229.16	ENSP00000228825.7	198.4	534
VYTTMAEHF	9	0.0049	TSPAN1	ENSG00000117472.10	ENSP00000361072.1	383.1	723
TYPENWRAF	9	0.0134	EEF1G	ENSG00000254772.10	ENSP00000331901.4	279.4	1311
IYSQTYFRF	9	0.0018	TNFSF10	ENSG00000121858.11	ENSP00000241261.2	187.2	843
TYFRFQEEI	9	0.0914	TNFSF10	ENSG00000121858.11	ENSP00000241261.2	187.2	843
TYVYFTNEL	9	0.1507	TNFSF10	ENSG00000121858.11	ENSP00000241261.2	187.2	843
SQVEILQRV	9	4.4332	ID3	ENSG00000117318.9	ENSP00000363689.5	197.9	357
AQLLQLKV	8	25.6667	FKBP8	ENSG00000105701.16	ENSP00000476767.1	260.3	1239
KYPDRVPVI	9	0.0180	GABARAPL2	ENSG00000034713.8	ENSP00000037243.2	176.9	351
RYQDAIRVF	9	0.0072	EIF3L	ENSG00000100129.18	ENSP00000384634.1	246.8	1398
TYMGHTGAVW	10	0.1733	EIF3I	ENSG00000084623.11	ENSP00000362688.1	177.6	975
EYILVPSTF	9	0.0065	CAPN2	ENSG00000162909.18	ENSP00000295006.5	202.9	2100
SYKNGFLNL	9	0.0659	UBA1	ENSG00000130985.17	ENSP00000338413.6	274.7	3174
EIVSRVSKR	9	28.1250	UBA1	ENSG00000130985.17	ENSP00000338413.6	274.7	3174
YIIGLLQQR	9	13.5225	FBXL5	ENSG00000118564.14	ENSP00000425472.1	184.3	1836
NYTPYKYNL	9	0.0744	HGD	ENSG00000113924.12	ENSP00000283871.5	239.2	1335
VYTTQPGVQF	10	0.0260	GALM	ENSG00000143891.17	ENSP00000272252.5	165.2	1026
EVAPPEYHR	9	10.8168	MORF4L1	ENSG00000185787.14	ENSP00000452717.1	253.4	705
EVAPPEYHRK	10	20.9259	MORF4L1	ENSG00000185787.14	ENSP00000452717.1	253.4	705
VYGPLPQSF	9	0.0008	COX4I1	ENSG00000131143.8	ENSP00000455301.1	481.3	324

EQRAIAKI	8	25.2500	EIF4G2	ENSG00000110321.17	ENSP00000379778.2	218.4	2607
HQLSLRTV	8	19.1290	NPM1	ENSG00000181163.13	ENSP00000377408.2	340.4	777
ECIPKTHER	9	20.4074	PPP1R1A	ENSG00000135447.17	ENSP00000257905.8	160.1	513
RYLQTLTTI	9	0.0160	STOM	ENSG00000148175.12	ENSP00000286713.2	167.0	864
ELLQGGTSR	9	26.0000	BHLHE40	ENSG00000134107.4	ENSP00000256495.3	161.1	1236
LDEEEDTHL	9	20.7407	PODXL	ENSG00000128567.17	ENSP00000367817.3	197.9	1674
YISEHEHF	8	0.4765	CLPTM1	ENSG00000104853.16	ENSP00000443192.1	198.7	1701
VYISEHEHF	9	0.0020	CLPTM1	ENSG00000104853.16	ENSP00000443192.1	198.7	1701
EVMRLVKGM	9	17.5778	ASNA1	ENSG00000198356.11	ENSP00000481254.1	156.9	993
RYLHTLQSL	9	0.0149	NPHS2	ENSG00000116218.12	ENSP00000356587.4	148.3	1149
DYLKNTYRL	9	0.0631	DPP4	ENSG00000197635.10	ENSP00000353731.3	144.2	2298
SSARVGLARART	12	80.0000	NAPSA	ENSG00000131400.8	ENSP00000253719.1	155.1	1260
MQIRIHQI	8	6.1989	DNAJA1	ENSG00000086061.16	ENSP00000369127.3	142.0	1191
VQPRNWLLF	9	0.1451	MPC1	ENSG00000060762.19	ENSP00000354223.6	220.7	327
EVAQLIQGGR	10	60.0000	MPC1	ENSG00000060762.19	ENSP00000354223.6	220.7	327
RFKTQPVTF	9	0.0563	C11orf96	ENSG00000187479.7	ENSP00000479976.1	135.2	366
DVIRPGMRR	9	27.1000	DNAJB1	ENSG00000132002.8	ENSP00000254322.1	142.6	1020
KYGPIRQI	8	0.5562	SF3B6	ENSG00000115128.7	ENSP00000233468.4	139.2	375
VYGFVREAL	9	0.2693	UBXN6	ENSG00000167671.12	ENSP00000301281.5	143.5	1323
IFRAPDMDQ	9	30.1667	MARC2	ENSG00000117791.16	ENSP00000355880.3	149.4	1005
LIFRAPDMDQ	10	80.0000	MARC2	ENSG00000117791.16	ENSP00000355880.3	149.4	1005
DLIGCIIGR	9	34.0000	PCBP2	ENSG00000197111.15	ENSP00000456842.1	531.0	552
TYQLGFHSI	9	0.0211	PSMB6	ENSG00000142507.10	ENSP00000270586.3	147.7	717
ETIHADSVAKR	11	28.8750	MSRB1	ENSG00000198736.11	ENSP00000480943.1	125.1	345
EVIQHRFESL	10	14.7111	SPTBN1	ENSG00000115306.16	ENSP00000334156.5	224.4	6465
TYISSVYHV	9	0.0593	SLC17A3	ENSG00000124564.17	ENSP00000355307.6	178.6	1260
DQNPDAALR	9	25.5833	COPE	ENSG00000105669.14	ENSP00000262812.3	152.4	924

FQYFVKVV	8	27.9000	ERGIC3	ENSG00000125991.19	ENSP00000399124.1	312.8	1191
ELIAKIPNF	9	0.9133	SET	ENSG00000119335.16	ENSP00000318012.8	131.4	831
YYFEGIKQTF	10	0.0027	AHSA1	ENSG00000100591.8	ENSP00000216479.3	144.7	1014
IYISTLKTEF	10	0.0238	AHSA1	ENSG00000100591.8	ENSP00000216479.3	144.7	1014
RYQKSTELL	9	0.0247	H3F3A	ENSG00000163041.11	ENSP00000355781.1	240.4	408
RYIANTVEL	9	0.0295	RPN2	ENSG00000118705.17	ENSP00000237530.6	234.8	1893
RYIPTAAAF	9	0.0046	SEC61A1	ENSG00000058262.10	ENSP00000243253.3	112.9	1428
QYNTYPIKL	9	0.0493	SEC61A1	ENSG00000058262.10	ENSP00000243253.3	112.9	1428
KYAPSGFYI	9	0.0119	WDR1	ENSG00000071127.17	ENSP00000371890.2	188.5	1818
RYNPITKRI	9	0.0169	AOX1	ENSG00000138356.14	ENSP00000363832.2	112.6	4014
YYDKHFTEF	9	0.0045	ATP6V1A	ENSG00000114573.10	ENSP00000273398.3	110.3	1851
EYYDKHFTEF	10	0.0239	ATP6V1A	ENSG00000114573.10	ENSP00000273398.3	110.3	1851
YYQHIVTTL	9	0.0054	PCYOX1	ENSG00000116005.12	ENSP00000387654.2	119.5	1515
SLFAGGMLR	9	12.8264	TMED9	ENSG00000184840.11	ENSP00000330945.6	107.7	705
KYLSVQGQLF	10	0.0077	MTCH1	ENSG00000137409.19	ENSP00000362730.5	177.3	1167
EVIKQLKEK	9	28.8750	SBDS	ENSG00000126524.10	ENSP00000246868.2	112.5	750
SQIPLSKI	8	10.3550	XRCC5	ENSG00000079246.16	ENSP00000375977.2	109.6	2196
RYLDGWNAI	9	0.0772	CYP27A1	ENSG00000135929.9	ENSP00000258415.4	110.9	1593
EVIDDFMTR	9	10.9466	CYP27A1	ENSG00000135929.9	ENSP00000258415.4	110.9	1593
TYGGSWKF	8	0.1706	AIG1	ENSG00000146416.18	ENSP00000350509.4	126.5	714
NYLNHWNHF	9	0.0085	FN3K	ENSG00000167363.14	ENSP00000300784.7	126.6	927
YIKHPVSL	8	1.2395	PSMD8	ENSG00000099341.11	ENSP00000481136.1	119.8	1050
VYIKHPVSL	9	0.0059	PSMD8	ENSG00000099341.11	ENSP00000481136.1	119.8	1050
EVAGIVAAR	9	22.3158	ACOT7	ENSG00000097021.19	ENSP00000367086.2	201.1	1140
KYNPTWHCI	9	0.0245	DYNLL1	ENSG00000088986.11	ENSP00000242577.6	312.3	267
KFNPVSFTW	9	0.0110	KLHDC3	ENSG00000124702.18	ENSP00000313995.4	101.1	1146
VYQEMPAQL	9	0.0119	BCKDHA	ENSG00000248098.12	ENSP00000269980.2	104.5	1335

ECIGNVKVM	9	24.8571	ADH5	ENSG00000197894.11	ENSP00000296412.8	212.5	1122
PYISNIYLI	9	0.0371	PLOD1	ENSG00000083444.17	ENSP00000196061.4	101.4	2181
RYLDKTEQW	9	0.0047	C4A	ENSG00000244731.8	ENSP00000396688.2	253.2	5232
YYPRVEYGF	9	0.0091	C4A	ENSG00000244731.8	ENSP00000396688.2	253.2	5232
HYPLNTVTF	9	0.0074	TNS1	ENSG00000079308.19	ENSP00000498399.1	129.3	5481
VYTSGIYNI	9	0.0158	TNS1	ENSG00000079308.19	ENSP00000498399.1	129.3	5481
RWFDKSFTF	9	0.0040	CPT1A	ENSG00000110090.13	ENSP00000265641.4	101.9	2319
DVYENLYAGR	10	30.0000	MAPKAPK2	ENSG00000162889.10	ENSP00000356070.3	101.0	1200
LQLQLPRL	8	10.2137	PSME1	ENSG00000092010.15	ENSP00000206451.6	188.9	747
LYDIILKNF	9	0.0433	PSME1	ENSG00000092010.15	ENSP00000206451.6	188.9	747
NYTKAMRLF	9	0.0481	ADI1	ENSG00000182551.14	ENSP00000333666.3	137.5	537
RYVRKFVLM	9	0.0737	CHMP2A	ENSG00000130724.8	ENSP00000469240.1	152.0	666
NFTNVAATF	9	0.1463	ACLY	ENSG00000131473.17	ENSP00000345398.1	105.6	3273
SYLEAFYKF	9	0.0009	ATP6V0D2	ENSG00000147614.4	ENSP00000285393.3	92.3	1050
EVIPYTPAM	9	5.3868	HMOX1	ENSG00000100292.17	ENSP00000216117.8	92.4	864
HQAAIVSKI	9	3.7087	CALD1	ENSG00000122786.20	ENSP00000354513.2	118.5	1614
IQYALRTL	8	7.6586	SARS	ENSG00000031698.13	ENSP00000234677.2	128.9	1542
EVMQEVAQL	9	6.5793	SARS	ENSG00000031698.13	ENSP00000234677.2	128.9	1542
KYLDIPKML	9	0.0220	ACTN4	ENSG00000130402.12	ENSP00000252699.2	583.1	2733
VYVQHPITF	9	0.0016	LITAF	ENSG00000189067.12	ENSP00000340118.5	108.6	483
EVIEKLFAM	9	4.8930	EPAS1	ENSG00000116016.14	ENSP00000263734.3	98.8	2610
LYLKALEGF	9	0.0273	EPAS1	ENSG00000116016.14	ENSP00000263734.3	98.8	2610
QWPPDPPLHF	10	0.0248	EPAS1	ENSG00000116016.14	ENSP00000263734.3	98.8	2610
TFLKARLV	8	7.3156	SEMA3B	ENSG00000012171.19	ENSP00000480680.1	146.1	2262
MWSPTHPALF	10	0.0565	DYNC1I2	ENSG00000077380.15	ENSP00000386591.1	104.3	1896
ETPSWTGSGF	10	4.5089	LAMB2	ENSG00000172037.14	ENSP00000307156.4	183.4	5394
KFIWTNHKF	9	0.0245	NDUFA12	ENSG00000184752.13	ENSP00000330737.2	97.6	435

ELVSGVVAR	9	24.5000	VAT1	ENSG00000108828.16	ENSP00000347872.2	105.8	1179
EVVASIKAR	9	33.0000	SLC9A3R2	ENSG00000065054.14	ENSP00000456895.1	158.8	678
APQMDWNRKRE	11	65.0000	SLC9A3R2	ENSG00000065054.14	ENSP00000456895.1	158.8	678
KEKEPEKLDKL	11	7.7535	SF3B2	ENSG00000087365.15	ENSP00000318861.6	133.9	2685
VYCNLLHSNSF	11	0.1475	PLPP1	ENSG00000067113.17	ENSP00000302229.8	103.4	852
TYTDVTPRQF	10	0.0234	STARD7	ENSG00000084090.13	ENSP00000338030.5	98.6	1110
TYTDVTPRQFF	11	0.0277	STARD7	ENSG00000084090.13	ENSP00000338030.5	98.6	1110
MQVLVSRI	8	20.1852	BCL2L1	ENSG00000171552.13	ENSP00000365230.2	130.2	699
KYKGIFNGF	9	0.0226	SLC25A3	ENSG00000075415.12	ENSP00000383898.3	178.2	1083
VYQPEHMSF	9	0.0012	MSRA	ENSG00000175806.15	ENSP00000429461.1	207.0	639
ESITDVLVR	9	38.0000	MCL1	ENSG00000143384.13	ENSP00000358022.2	91.3	1050
ETAFQGMLR	9	29.8571	MCL1	ENSG00000143384.13	ENSP00000358022.2	91.3	1050
AYFKDFHLL	9	0.0062	SLC13A1	ENSG00000081800.9	ENSP00000194130.2	91.5	1785
EVNEAELLRR	10	45.5000	CAVIN1	ENSG00000177469.13	ENSP00000349541.4	83.4	1170
EVIEESRAER	10	45.0000	CAVIN1	ENSG00000177469.13	ENSP00000349541.4	83.4	1170
NYVKDLSRL	9	0.1965	CTDSP1	ENSG00000144579.7	ENSP00000273062.2	145.1	783
SYIEHIFEI	9	0.0059	PEA15	ENSG00000162734.12	ENSP00000353660.4	87.2	390
KYVPAIAHL	9	0.0073	CPVL	ENSG00000106066.15	ENSP00000265394.5	103.0	1428
KYVPAIAHLIHS	12	6.9851	CPVL	ENSG00000106066.15	ENSP00000265394.5	103.0	1428
RYMDAWNTV	9	0.0783	TIMM13	ENSG00000099800.8	ENSP00000215570.2	82.4	285
RFIPRENGVYL	11	0.6566	FLNA	ENSG00000196924.17	ENSP00000358872.4	171.2	7860
TYTEVVTEF	9	0.0027	AEBP1	ENSG00000106624.11	ENSP00000223357.3	102.7	3474
LYANMFERL	9	0.0553	FKBP4	ENSG00000004478.8	ENSP00000001008.4	87.7	1377
EVLSKALSQR	10	35.5000	GADD45A	ENSG00000116717.13	ENSP00000360025.4	94.1	495
EVVDFIQSKI	10	21.8182	PPM1G	ENSG00000115241.11	ENSP00000342778.4	80.1	1638
PYKVTQDEL	9	0.5289	NCL	ENSG00000115053.16	ENSP00000318195.4	157.9	2130
AVVDVRIGM	9	14.1222	NCL	ENSG00000115053.16	ENSP00000318195.4	157.9	2130

AVVDVRIGMTR	11	31.3333	NCL	ENSG00000115053.16	ENSP00000318195.4	157.9	2130
IYNHITTRV	9	0.1704	ADD1	ENSG00000087274.17	ENSP00000421907.2	201.5	1896
KQMRILHV	8	10.6412	GNAS	ENSG00000087460.25	ENSP00000360136.3	720.5	1140
TYQNDIALI	9	0.1291	CFI	ENSG00000205403.13	ENSP00000378131.3	196.5	1773
VYDDLKYVW	9	0.0175	RENBP	ENSG00000102032.13	ENSP00000377303.3	129.1	1281
VYGNERFIQYL	11	0.4116	PICALM	ENSG00000073921.18	ENSP00000433846.1	128.0	1935
VFMKSVKLEW	10	0.3481	PRPF6	ENSG00000101161.8	ENSP00000266079.4	75.4	2823
KYLELCVDL	9	0.3992	EIF3A	ENSG00000107581.13	ENSP00000358140.3	75.6	4146
DVIAALRSR	9	26.1000	KIFC3	ENSG00000140859.16	ENSP00000438805.2	185.8	2172
KWNPTAGVAF	10	0.1926	POLR2C	ENSG00000102978.13	ENSP00000219252.4	81.3	825
KYWDVPPPGF	10	0.0536	U2AF2	ENSG00000063244.12	ENSP00000388475.1	96.6	1413
KYIPICPVF	9	0.0044	SLC23A1	ENSG00000170482.17	ENSP00000302701.4	90.6	1794
EYTDVYPEI	9	0.0982	MAGED2	ENSG00000102316.17	ENSP00000364198.1	179.1	1818
RFPRTIHMTF	10	0.0242	ACY1	ENSG00000243989.9	ENSP00000419830.1	398.1	684
RSFQTSAI	8	22.3158	ATP5MC2	ENSG00000135390.19	ENSP00000377878.4	155.5	423
KMPKMKMPTF	10	0.3399	AHNAK	ENSG00000124942.14	ENSP00000367263.4	75.7	17670
KMPKVKMPKF	10	0.3886	AHNAK	ENSG00000124942.14	ENSP00000367263.4	75.7	17670
RYRGDYDRF	9	0.1135	HNRNPUL2	ENSG00000214753.3	ENSP00000301785.5	71.1	2241
VYLQNWSHVL	10	0.1293	GPS1	ENSG00000169727.12	ENSP00000485035.1	125.8	1578
KYQEVTNNL	9	0.0116	CAPRIN1	ENSG00000135387.21	ENSP00000434150.1	99.2	2127
KYQEVTNNLEF	11	0.0154	CAPRIN1	ENSG00000135387.21	ENSP00000434150.1	99.2	2127
VQLRLNSI	8	12.9167	PPP2R1A	ENSG00000105568.18	ENSP00000324804.6	113.2	1767
EVCGQDITTK	10	49.0000	PPP2R1A	ENSG00000105568.18	ENSP00000324804.6	113.2	1767
EVISPDTTER	10	30.0000	COL6A2	ENSG00000142173.15	ENSP00000300527.4	106.4	3057
SYNRTFMKL	9	0.0236	MAN1A1	ENSG00000111885.7	ENSP00000357453.3	68.2	1959
KYIDKTIRV	9	0.0893	LSM7	ENSG00000130332.15	ENSP00000252622.8	97.8	309
KYPSPFFVF	9	0.0015	DHX9	ENSG00000135829.17	ENSP00000356520.3	74.0	3810

NYLVRINEI	9	0.1370	DHX9	ENSG00000135829.17	ENSP00000356520.3	74.0	3810
HVIVDEIHER	10	27.7000	DHX9	ENSG00000135829.17	ENSP00000356520.3	74.0	3810
DTISPTLGF	9	2.2759	ARL2	ENSG00000213465.8	ENSP00000246747.4	106.5	552
NYLDRFLSL	9	0.0387	CCND1	ENSG00000110092.3	ENSP00000227507.2	68.7	885
ETIPLTAEK	9	20.4074	CCND1	ENSG00000110092.3	ENSP00000227507.2	68.7	885
RYTQEIFSF	9	0.0039	SLC4A1	ENSG00000004939.15	ENSP00000262418.6	127.7	2733
EVPREKLASR	10	31.8333	LARP1	ENSG00000155506.17	ENSP00000336721.4	80.4	3057
KYLSDNVHL	9	0.0732	CDC37	ENSG00000105401.9	ENSP00000222005.1	106.8	1134
EVIPPYYSY	9	2.6495	TDP2	ENSG00000111802.14	ENSP00000367440.4	86.1	1086
KYLATLETL	9	0.0213	JAK1	ENSG00000162434.12	ENSP00000343204.4	66.3	3462
SYMPTVSHL	9	0.0043	BSCL2	ENSG00000168000.14	ENSP00000413209.1	86.2	1194
AYLRIHAHF	9	0.0068	BSCL2	ENSG00000168000.14	ENSP00000413209.1	86.2	1194
RYPNRFKLW	9	0.0463	CYB5R1	ENSG00000159348.13	ENSP00000356218.4	76.2	915
EYFSYPVSL	9	0.0790	SCNN1A	ENSG00000111319.13	ENSP00000353292.3	183.2	2184
RLPETLPSL	9	0.4957	SCNN1A	ENSG00000111319.13	ENSP00000353292.3	183.2	2184
KYPLLLDNI	9	0.1412	ARHGEF12	ENSG00000196914.9	ENSP00000432984.1	70.9	4323
KWGPASQQI	9	0.1498	HNF1B	ENSG00000275410.5	ENSP00000480291.1	106.2	1671
YYLNDLERI	9	0.0630	GNAI2	ENSG00000114353.17	ENSP00000312999.6	154.7	1065
KYQEEFEHF	9	0.0016	LMAN1	ENSG00000074695.6	ENSP00000251047.4	63.2	1530
TVVGHWAGSR	10	44.0000	FAM120A	ENSG00000048828.17	ENSP00000277165.5	76.5	3354
VQPSKYHFL	9	0.2639	MAGEF1	ENSG00000177383.4	ENSP00000315064.3	61.9	921
FVIDSDHLGHR	11	30.6667	COASY	ENSG00000068120.15	ENSP00000393564.2	75.1	1692
KYNMVSYLL	9	0.0247	GPAT3	ENSG00000138678.11	ENSP00000378651.2	132.0	1302
KYNPQFGDAFW	11	0.2131	GPAT3	ENSG00000138678.11	ENSP00000378651.2	132.0	1302
KYGIVLKEF	9	0.0168	GTF2F1	ENSG00000125651.14	ENSP00000377969.3	84.7	1551
VYSRTFTW	8	0.1570	MYO1C	ENSG00000197879.17	ENSP00000354283.2	77.8	3084
KYMDVQFDF	9	0.0045	MYO1C	ENSG00000197879.17	ENSP00000354283.2	77.8	3084

VYSRTFTWL	9	0.0981	MYO1C	ENSG00000197879.17	ENSP00000354283.2	77.8	3084
NYPQSVPRL	9	0.0404	MYO1C	ENSG00000197879.17	ENSP00000354283.2	77.8	3084
AIVDVVANR	9	17.4222	ANXA7	ENSG00000138279.16	ENSP00000362012.4	116.4	1398
ESIGRVLAQR	10	39.5000	MRPL18	ENSG00000112110.10	ENSP00000356001.4	67.1	540
YFKDTHPKF	9	0.0239	CYB5R3	ENSG00000100243.21	ENSP00000338461.6	85.0	903
EIISHDTRR	9	39.0000	CYB5R3	ENSG00000100243.21	ENSP00000338461.6	85.0	903
IYTSSVNRL	9	0.0348	COPB2	ENSG00000184432.10	ENSP00000422295.1	83.5	2631
TYPQGFKVW	9	0.0537	CYP4F2	ENSG00000186115.13	ENSP00000221700.3	75.1	1560
YYLTPDGQRF	10	0.0090	CYP4F2	ENSG00000186115.13	ENSP00000221700.3	75.1	1560
VYIIGSSGF	9	0.0393	SCCPDH	ENSG00000143653.10	ENSP00000355467.3	59.4	1287
KYTGVLTEL	9	0.0483	GLDC	ENSG00000178445.9	ENSP00000370737.3	104.4	3060
TYMKEILRF	9	0.0014	SLC17A5	ENSG00000119899.13	ENSP00000348019.5	58.4	1485
KYIQKPGLL	9	0.0906	PAQR7	ENSG00000182749.5	ENSP00000363414.3	57.7	1038
IYEPLHTHW	9	0.0109	PAQR7	ENSG00000182749.5	ENSP00000363414.3	57.7	1038
YIQKPGLLGR	10	23.4375	PAQR7	ENSG00000182749.5	ENSP00000363414.3	57.7	1038
GQWKKGFVL	9	2.7258	SERBP1	ENSG00000142864.14	ENSP00000360034.2	104.3	1224
YKYKLLPRS	9	56.6667	MAOA	ENSG00000189221.9	ENSP00000340684.3	69.4	1581
IYIAGHPAF	9	0.0099	HNRNPL	ENSG00000104824.17	ENSP00000221419.4	153.0	1767
AVASFPKKQE	10	80.0000	COX7A2	ENSG00000112695.11	ENSP00000359098.2	152.5	345
IYNGDMEKI	9	0.1535	BNIP3	ENSG00000176171.11	ENSP00000357625.5	70.0	777
DVISSIRNF	9	1.7773	TOMM5	ENSG00000175768.13	ENSP00000313584.6	57.9	153
NYARGHYTI	9	0.0422	TUBA1A	ENSG00000167552.14	ENSP00000446637.1	65.6	1248
YIRGSKIRF	9	2.8192	SNRPD3	ENSG00000100028.12	ENSP00000215829.3	59.7	378
VYIRGSKIRF	10	0.0309	SNRPD3	ENSG00000100028.12	ENSP00000215829.3	59.7	378
KYSDITIMF	9	0.0019	GAMT	ENSG00000130005.12	ENSP00000492031.1	109.1	639
ETAPAVLER	9	16.2545	RGN	ENSG00000130988.13	ENSP00000253303.4	71.4	897
LYSHLVDYF	9	0.0212	GNA11	ENSG00000088256.9	ENSP00000078429.3	62.1	1077

VYQNIFTAM	9	0.1127	GNA11	ENSG00000088256.9	ENSP00000078429.3	62.1	1077
YYIDKLEYL	9	0.0077	NET1	ENSG00000173848.19	ENSP00000369717.3	59.9	1626
KYPLLLKEI	9	0.0815	NET1	ENSG00000173848.19	ENSP00000369717.3	59.9	1626
DVRMGGSFR	9	48.0000	NET1	ENSG00000173848.19	ENSP00000369717.3	59.9	1626
NYVDLVSSL	9	0.1586	ECI2	ENSG00000198721.12	ENSP00000417170.2	212.3	420
GYLADPAKF	9	0.0404	MRPL15	ENSG00000137547.8	ENSP00000260102.4	57.7	888
RYPTTFVMV	9	0.1323	ARL6IP5	ENSG00000144746.7	ENSP00000273258.3	54.9	564
RYPDSHQLF	9	0.0020	G3BP2	ENSG00000138757.14	ENSP00000350518.3	77.4	1347
AYVPGFAHI	9	0.0192	CUEDC2	ENSG00000107874.11	ENSP00000358953.4	67.9	861
KYLRRLFRF	9	0.0142	UNC50	ENSG00000115446.11	ENSP00000350409.2	57.6	777
RYAEWEERI	9	0.0993	GBA2	ENSG00000070610.14	ENSP00000367343.3	120.7	2781
TYDNVHQQF	9	0.0055	ARHGAP24	ENSG00000138639.18	ENSP00000378610.2	128.5	1959
MYPDVFAHF	9	0.0008	PC	ENSG00000173599.14	ENSP00000377527.2	70.0	3534
EVIPPMKEF	9	1.2290	NDUFB6	ENSG00000165264.11	ENSP00000369176.3	60.8	384
RWSPVRPLVF	10	0.0409	WDR34	ENSG00000119333.11	ENSP00000361800.2	101.5	1608
AYLEAIHNF	9	0.0009	AP2S1	ENSG00000042753.11	ENSP00000263270.5	84.8	426
VYTVVDEMF	9	0.0213	AP2S1	ENSG00000042753.11	ENSP00000263270.5	84.8	426
NYGPMKGGSF	10	0.3896	HNRNPA3	ENSG00000170144.20	ENSP00000408487.1	71.9	1068
KYQKGFSLW	9	0.0098	TRAM1	ENSG00000067167.8	ENSP00000262213.2	67.6	1122
AYLLNLNHL	9	0.0529	TRAM1	ENSG00000067167.8	ENSP00000262213.2	67.6	1122
AYPHNLMTF	9	0.0041	TRAM1	ENSG00000067167.8	ENSP00000262213.2	67.6	1122
NYISDPTIL	9	0.1974	TRAM1	ENSG00000067167.8	ENSP00000262213.2	67.6	1122
RWFDKTLQF	9	0.0068	CRAT	ENSG00000095321.17	ENSP00000315013.2	122.3	1878
EYSKQMQRF	9	0.0416	HDAC1	ENSG00000116478.12	ENSP00000362649.3	64.3	1446
RMLPHAPGV	9	3.9079	HDAC1	ENSG00000116478.12	ENSP00000362649.3	64.3	1446
PYNDYFEYF	9	0.0117	HDAC1	ENSG00000116478.12	ENSP00000362649.3	64.3	1446
VYHHLVETL	9	0.0106	GGT5	ENSG00000099998.17	ENSP00000381340.3	70.1	1761

HVVAPVLSR	9	18.0263	GGT5	ENSG00000099998.17	ENSP00000381340.3	70.1	1761
VYHHLVETLKF	11	0.0541	GGT5	ENSG00000099998.17	ENSP00000381340.3	70.1	1761
VYAAGSQFHSF	11	0.0123	GPT	ENSG00000167701.14	ENSP00000378408.2	78.2	1488
RYGDLVDYL	9	0.1140	PDGFRB	ENSG00000113721.14	ENSP00000261799.4	68.7	3318
TYGEIFEKF	9	0.0009	NDUFC2	ENSG00000151366.13	ENSP00000281031.4	72.2	357
LYADVGGKQF	10	0.1143	SSR4	ENSG00000180879.13	ENSP00000359103.3	131.4	519
TYIKSPPFF	9	0.0020	ACO1	ENSG00000122729.19	ENSP00000309477.5	60.0	2667
HQQEISKI	8	14.2889	CDC42BPB	ENSG00000198752.11	ENSP00000355237.2	60.3	5133
KYVAEFLEW	9	0.0170	CDC42BPB	ENSG00000198752.11	ENSP00000355237.2	60.3	5133
VYAIKNHYW	9	0.0144	TM9SF3	ENSG00000077147.16	ENSP00000360184.4	54.8	1767
EVINYEPIGY	10	37.6667	MYLK	ENSG00000065534.18	ENSP00000418335.1	130.1	5742
QYLEEAPKF	9	0.0061	TRAPPC6A	ENSG00000007255.10	ENSP00000468612.1	71.2	477
KYLLADCNEAF	11	0.3640	RAD21	ENSG00000164754.14	ENSP00000297338.2	62.5	1893
GYSPYQLTW	9	0.0218	NFKBIA	ENSG00000100906.10	ENSP00000450514.1	76.4	681
KYEELFPAF	9	0.0153	NAPA	ENSG00000105402.8	ENSP00000263354.2	80.5	885
IYARAANMF	9	0.0084	NAPA	ENSG00000105402.8	ENSP00000263354.2	80.5	885
IYTDMGRFTI	10	0.2109	NAPA	ENSG00000105402.8	ENSP00000263354.2	80.5	885
RYSVFFQSL	9	0.0221	PSMD6	ENSG00000163636.10	ENSP00000295901.4	61.1	1167
SYVVAMETF	9	0.0110	DDB1	ENSG00000167986.13	ENSP00000301764.7	164.1	3420
LQFPVGRV	8	25.8333	HIST2H2AA4	ENSG00000272196.2	ENSP00000475814.1	49.3	390
QYLDRAEKL	9	0.0838	VPS4A	ENSG00000132612.16	ENSP00000254950.11	55.2	1311
RYLGKVLEL	9	0.0161	CLUH	ENSG00000132361.17	ENSP00000464732.2	122.3	3753
YYALEVSYF	9	0.0078	COPS5	ENSG00000121022.14	ENSP00000350512.4	65.4	1002
VFHPSQDLVF	10	0.1043	PRPF19	ENSG00000110107.9	ENSP00000227524.4	56.6	1512
IFKPPDPDNTF	11	0.1027	MOAP1	ENSG00000165943.5	ENSP00000298894.4	48.8	1053
TYLKKIPGF	9	0.0076	SERINC3	ENSG00000132824.14	ENSP00000340243.4	49.8	1419
AQHKFLVAV	9	8.6933	TCF25	ENSG00000141002.20	ENSP00000457329.1	153.3	795

HYQDVSCLQF	10	0.0270	WDR18	ENSG00000065268.10	ENSP00000466551.1	57.8	804
GYPLWKVTF	9	0.0240	PNP	ENSG00000198805.11	ENSP00000452421.1	278.6	663
LQFPVGRI	8	16.9273	H2AFZ	ENSG00000164032.12	ENSP00000296417.5	76.1	384
RCAHIEAKF	9	3.0442	NAP1L4	ENSG00000205531.13	ENSP00000436397.1	97.7	1158
AYDTVRWEF	9	0.0303	DPP7	ENSG00000176978.14	ENSP00000360635.2	117.5	1476
IYTRLLPAL	9	0.1740	ACY1	ENSG00000243989.9	ENSP00000490149.1	398.1	1224
RYLEQLHQL	9	0.0039	STAT3	ENSG00000168610.14	ENSP00000264657.4	78.6	2310
RYLEKPMEI	9	0.0074	STAT3	ENSG00000168610.14	ENSP00000264657.4	78.6	2310
RYLNEFEEL	9	0.0243	EIF2AK1	ENSG00000086232.13	ENSP00000199389.6	48.5	1890
SYLKDTKLW	9	0.0551	STK24	ENSG00000102572.14	ENSP00000365730.3	68.2	1329
VWNPRSHEKL	10	0.5127	WDR6	ENSG00000178252.18	ENSP00000413432.1	70.0	3210
LYMKNGQGF	9	0.0203	RAP1B	ENSG00000127314.18	ENSP00000445090.1	67.5	354
TYQDIQNTI	9	0.0114	POLR2A	ENSG00000181222.16	ENSP00000499521.1	57.3	5910
VYASDRATF	9	0.0093	ECI2	ENSG00000198721.12	ENSP00000369461.3	212.3	1182
ELAYADVAKR	10	44.0000	CLIC5	ENSG00000112782.17	ENSP00000344165.6	49.4	753
RYLEVMRKL	9	0.0208	PTPA	ENSG00000119383.19	ENSP00000351885.4	102.2	969
KYSKRFPEL	9	0.0329	PRPF31	ENSG00000105618.14	ENSP00000375635.1	60.4	1479
VYFSAIQKI	9	0.0061	BAIAP2L2	ENSG00000128298.17	ENSP00000371085.3	46.2	1587
IYKSIMEQF	9	0.0032	BAIAP2L2	ENSG00000128298.17	ENSP00000371085.3	46.2	1587
EVVGEQVTSY	10	30.1667	CNDP2	ENSG00000133313.15	ENSP00000462311.1	167.7	1425
YYFDSYAHF	9	0.0014	PRMT1	ENSG00000126457.21	ENSP00000406162.2	147.9	1113
EYMLQNHVF	9	0.0320	IPO7	ENSG00000205339.10	ENSP00000369042.3	55.4	3114
KYLEMIYSM	9	0.0251	IPO7	ENSG00000205339.10	ENSP00000369042.3	55.4	3114
NYGRVFSEW	9	0.0341	SNX4	ENSG00000114520.11	ENSP00000251775.4	47.3	1350
RYLPTGSFPF	10	0.0172	TUSC3	ENSG00000104723.20	ENSP00000424544.1	52.2	1044
RYLPTGSFPFL	11	0.1518	TUSC3	ENSG00000104723.20	ENSP00000424544.1	52.2	1044
VFAGVFNTF	9	0.0083	F13A1	ENSG00000124491.15	ENSP00000264870.3	44.8	2196

EYATKISRF	9	0.0315	PITHD1	ENSG00000057757.10	ENSP00000246151.4	45.7	633
EVVAKFLHK	9	21.3636	PPP1CA	ENSG00000172531.15	ENSP00000365936.4	110.7	990
KYPENFFLL	9	0.0032	PPP1CA	ENSG00000172531.15	ENSP00000365936.4	110.7	990
EYMKHTRLF	9	0.0160	FLII	ENSG00000177731.16	ENSP00000438536.2	103.2	3642
ETVDAVQER	9	25.5833	SUN1	ENSG00000164828.18	ENSP00000409909.1	74.3	2130
VYSPHVLNL	9	0.0042	DNM2	ENSG00000079805.16	ENSP00000352721.6	119.9	2598
KYLSGIAHF	9	0.0016	PMPCA	ENSG00000165688.12	ENSP00000360782.3	60.2	1575
RFLDDLGLKF	10	0.1283	DSG2	ENSG00000046604.13	ENSP00000261590.8	43.4	3354
EYLKLLHSF	9	0.0058	SREBF2	ENSG00000198911.12	ENSP00000354476.4	50.9	3423
TYTDRVFFL	9	0.0326	PLXNB2	ENSG00000196576.15	ENSP00000409171.1	297.5	5514
YYTDIMHTL	9	0.0046	PLXNB2	ENSG00000196576.15	ENSP00000409171.1	297.5	5514
ETFDYIGSSR	10	21.7273	NCSTN	ENSG00000162736.17	ENSP00000294785.5	112.1	2127
KYGSVTVW	8	0.3841	PPP4C	ENSG00000149923.14	ENSP00000455995.1	74.8	921
RYPDRITLI	9	0.0115	PPP4C	ENSG00000149923.14	ENSP00000455995.1	74.8	921
IYSIDFTRF	9	0.0066	UNC119B	ENSG00000175970.11	ENSP00000344942.4	44.8	753
RYQFTPAFL	9	0.0734	UNC119B	ENSG00000175970.11	ENSP00000344942.4	44.8	753
SQFSYQHAI	9	2.5023	EIF3H	ENSG00000147677.11	ENSP00000428669.1	121.7	705
RYQERFKHI	9	0.0756	SPRYD3	ENSG00000167778.9	ENSP00000301463.4	45.0	1326
TQYRAMFV	8	27.0000	EAPP	ENSG00000129518.9	ENSP00000250454.3	45.8	855
AYLESFYKF	9	0.0009	ATP6V0D1	ENSG00000159720.12	ENSP00000441282.1	214.3	1176
LYPEGLAQL	9	0.0651	ATP6V0D1	ENSG00000159720.12	ENSP00000441282.1	214.3	1176
VGARIYHTI	9	1.7211	MGST1	ENSG00000008394.13	ENSP00000379513.3	166.0	465
RYVLYPNNF	9	0.0080	HEXA	ENSG00000213614.9	ENSP00000268097.5	87.9	1587
SYLKQLPHF	9	0.0023	SNRNP200	ENSG00000144028.15	ENSP00000317123.5	112.4	6408
KYVHLFPKL	9	0.0203	SNRNP200	ENSG00000144028.15	ENSP00000317123.5	112.4	6408
ETQLPVSFR	9	24.0000	SNRNP200	ENSG00000144028.15	ENSP00000317123.5	112.4	6408
VYMDWYEKF	9	0.0007	SNRNP200	ENSG00000144028.15	ENSP00000317123.5	112.4	6408

IYTDQAGQWRI	11	0.4904	C12orf10	ENSG00000139637.14	ENSP00000267103.5	65.0	1128
YYTSVTPVL	9	0.0425	PTBP1	ENSG00000011304.20	ENSP00000342332.5	115.6	1581
EVFDFSQRR	9	14.6556	NFE2L2	ENSG00000116044.16	ENSP00000411575.2	72.7	1746
RYPTSIASL	9	0.0318	BUB3	ENSG00000154473.18	ENSP00000357851.5	54.3	978
SFHNIHNTF	9	0.0220	BUB3	ENSG00000154473.18	ENSP00000357851.5	54.3	978
RYPTSIASLAF	11	0.0548	BUB3	ENSG00000154473.18	ENSP00000357851.5	54.3	978
RYISQTQGL	9	0.0723	HIPK2	ENSG00000064393.16	ENSP00000385571.3	42.7	3594
YQRDPLKL	8	2.9613	STAT6	ENSG00000166888.12	ENSP00000451546.2	115.7	2595
IYQRDPLKL	9	0.0232	STAT6	ENSG00000166888.12	ENSP00000451546.2	115.7	2595
QYMKIISSF	9	0.0035	HSPA4	ENSG00000170606.15	ENSP00000302961.2	53.7	2520
YVVEKVLDR	9	21.2727	CBX1	ENSG00000108468.15	ENSP00000225603.4	44.2	555
TYMDAQLFKKV	11	0.8788	RGL1	ENSG00000143344.15	ENSP00000354097.3	40.7	2304
SYIELPAYL	9	0.0221	LAPTM5	ENSG00000162511.8	ENSP00000294507.3	40.9	786
SYMEVPTYL	9	0.0073	LAPTM5	ENSG00000162511.8	ENSP00000294507.3	40.9	786
VYSEVAAYEF	10	0.0130	DRG1	ENSG00000185721.12	ENSP00000329715.4	41.3	1101
FVVEKVLDR	9	27.9000	CBX3	ENSG00000122565.19	ENSP00000387348.1	69.8	303
YAHIYHQHF	9	0.7868	MOB1A	ENSG00000114978.18	ENSP00000379364.3	43.2	648
VYAHIYHQHF	10	0.0089	MOB1A	ENSG00000114978.18	ENSP00000379364.3	43.2	648
QYTHKIYHL	9	0.0221	PITPNA	ENSG00000174238.14	ENSP00000316809.7	53.5	810
TYLMLANHL	9	0.0856	GBE1	ENSG00000114480.13	ENSP00000410833.2	41.5	2106
VYADTIKDLL	10	0.1813	ACOT11	ENSG00000162390.17	ENSP00000340260.2	47.9	1782
EVFDKTYQFL	10	3.6101	RRP36	ENSG00000124541.7	ENSP00000244496.5	46.1	777
NYMDCISSL	9	0.1751	BICC1	ENSG00000122870.11	ENSP00000362993.3	40.1	2922
FYPYGLQTF	9	0.0102	SUN2	ENSG00000100242.15	ENSP00000385616.1	102.5	2214
KYKEAAHLL	9	0.0110	KLC4	ENSG00000137171.15	ENSP00000395806.2	79.1	1626
RYNPENNTW	9	0.0220	IVNS1ABP	ENSG00000116679.16	ENSP00000356468.3	181.6	1926
DVTSCISYR	9	38.0000	IVNS1ABP	ENSG00000116679.16	ENSP00000356468.3	181.6	1926

EWSPYTKIFQF	11	0.3630	IVNS1ABP	ENSG00000116679.16	ENSP00000356468.3	181.6	1926
EVATFFAKM	9	8.7311	TOP1	ENSG00000198900.6	ENSP00000354522.2	38.9	2295
VYNENLVHMI	10	0.2172	BCAS2	ENSG00000116752.6	ENSP00000358554.3	40.6	675
KYMAEALLL	9	0.0244	TOMM70	ENSG00000154174.7	ENSP00000284320.5	40.6	1824
HYAYSFKYL	9	0.1651	SUPT6H	ENSG00000109111.15	ENSP00000319104.6	42.8	5178
HQIVVDRV	8	26.1000	SEMA3G	ENSG00000010319.6	ENSP00000231721.2	42.3	2346
TYVTEVREL	9	0.1220	KTN1	ENSG00000126777.18	ENSP00000391964.2	126.3	3900
VYNANINLF	9	0.0025	PKD2	ENSG00000118762.8	ENSP00000237596.2	41.9	2904
LYIVHPTMF	9	0.0088	ARHGAP1	ENSG00000175220.12	ENSP00000310491.4	40.0	1317
KYVADFSQF	9	0.0069	KCNJ15	ENSG00000157551.19	ENSP00000381905.1	164.3	1125
MWKEGNRIHF	10	0.2915	HSD17B4	ENSG00000133835.16	ENSP00000425993.1	168.3	1797
TYDKGYQF	8	0.1860	PEX5	ENSG00000139197.10	ENSP00000266564.3	51.0	1893
EVFNKFFKER	10	25.0833	SUPT16H	ENSG00000092201.10	ENSP00000216297.2	39.9	3141
RYFYKVHQL	9	0.0092	BSDC1	ENSG00000160058.18	ENSP00000397759.2	63.3	1341
TYNYPVHYF	9	0.0034	TMEM30A	ENSG00000112697.16	ENSP00000230461.6	51.3	1083
TYIPVPAKI	9	0.0036	ST3GAL1	ENSG00000008513.16	ENSP00000428540.1	52.5	1020
KYFQGDLQLF	10	0.0077	PDK2	ENSG00000005882.11	ENSP00000007708.3	131.1	1029
TYQRWQFTL	9	0.0189	PRPF8	ENSG00000174231.17	ENSP00000304350.6	84.6	7005
EIRDIILGM	9	17.3556	PRPF8	ENSG00000174231.17	ENSP00000304350.6	84.6	7005
RYIDRIHIF	9	0.0010	PRPF8	ENSG00000174231.17	ENSP00000304350.6	84.6	7005
VYTTTVHWL	9	0.0444	PRPF8	ENSG00000174231.17	ENSP00000304350.6	84.6	7005
ETIHPRKSY	9	12.8958	PRPF8	ENSG00000174231.17	ENSP00000304350.6	84.6	7005
NYLHLDYNF	9	0.0210	PRPF8	ENSG00000174231.17	ENSP00000304350.6	84.6	7005
RYIDRIHIFF	10	0.0048	PRPF8	ENSG00000174231.17	ENSP00000304350.6	84.6	7005
VFQHYHAKF	9	0.0658	SCAP	ENSG00000114650.20	ENSP00000497087.1	73.9	3837
MQFRSILI	8	9.9824	UPF1	ENSG00000005007.12	ENSP00000262803.4	68.5	3354
RWSEVTRKLEF	11	0.1884	KIF13B	ENSG00000197892.13	ENSP00000427900.1	38.7	5478

RYTIVQQQI	9	0.0257	TUBGCP2	ENSG00000130640.13	ENSP00000252936.3	61.3	2706
EYGQVNHAL	9	0.2286	TUBGCP2	ENSG00000130640.13	ENSP00000252936.3	61.3	2706
KFYTDPSYF	9	0.0750	WASF2	ENSG00000158195.11	ENSP00000483313.1	36.0	1494
RYQPQSGYW	9	0.0459	TRIM6	ENSG00000121236.21	ENSP00000421079.1	124.8	939
VFLDYEAGTVSF	12	0.3169	TRIM6	ENSG00000121236.21	ENSP00000421079.1	124.8	939
LYLTNCEKL	9	0.2723	ZER1	ENSG00000160445.11	ENSP00000291900.2	39.6	2298
KFMPVSSLI	9	0.0906	FTSJ3	ENSG00000108592.17	ENSP00000396673.2	60.1	2541
RFIHNSHDLTY	11	1.5461	TBC1D4	ENSG00000136111.13	ENSP00000366852.2	35.7	3705
VYSETVDLIKF	11	0.0233	AFF1	ENSG00000172493.20	ENSP00000378578.4	47.0	3654
RYMEVSGNL	9	0.1194	SF3A3	ENSG00000183431.12	ENSP00000362110.4	44.2	1503
IYNPTTGEF	9	0.0082	ATP1B3	ENSG00000069849.11	ENSP00000286371.3	40.4	837
DVINPMALR	9	27.8000	SART1	ENSG00000175467.15	ENSP00000310448.5	37.8	2400
KYINTDAKF	9	0.0121	TRPC4AP	ENSG00000100991.12	ENSP00000252015.2	46.1	2391
SYADQMFLL	9	0.0304	TRPC4AP	ENSG00000100991.12	ENSP00000252015.2	46.1	2391
QYLRNPPKL	9	0.0404	SHC1	ENSG00000160691.18	ENSP00000398864.1	67.9	387
ETAPRTIFQR	10	23.9375	ATXN10	ENSG00000130638.17	ENSP00000252934.4	48.6	1425
VQRLEPVI	8	18.5526	PFKFB3	ENSG00000170525.21	ENSP00000492001.1	60.4	1704
HQNVFPNHI	9	2.8119	ANKRD10	ENSG00000088448.14	ENSP00000267339.2	75.7	1260
KQIEINTI	8	6.3780	GSS	ENSG00000100983.12	ENSP00000494819.1	74.7	1080
KYDPNVYSI	9	0.0092	ITGAV	ENSG00000138448.12	ENSP00000261023.3	34.8	3144
IYGAPGVEF	9	0.0076	LLGL2	ENSG00000073350.13	ENSP00000464397.1	134.2	3057
SVAHFGSRR	9	20.3704	LLGL2	ENSG00000073350.13	ENSP00000464397.1	134.2	3057
KFTPVASKF	9	0.0207	ZYX	ENSG00000159840.16	ENSP00000376642.2	44.4	1245
YIDRVRSL	8	2.5583	KRT18	ENSG00000111057.11	ENSP00000373487.3	85.6	1290
RYALQMEQL	9	0.0971	KRT18	ENSG00000111057.11	ENSP00000373487.3	85.6	1290
EYRPEFSNF	9	0.0662	PARP4	ENSG00000102699.6	ENSP00000371419.3	34.5	5172
QYPSICPRL	9	0.0573	PARP4	ENSG00000102699.6	ENSP00000371419.3	34.5	5172

AYGTVHYF	8	0.0970	LMF2	ENSG00000100258.18	ENSP00000216080.5	52.7	2046
TYAEISQAF	9	0.0040	SLC2A9	ENSG00000109667.12	ENSP00000264784.3	64.1	1620
RYVQQLQRL	9	0.0226	MRPL43	ENSG00000055950.16	ENSP00000299179.5	100.7	606
GPREAGLR	8	80.0000	CCDC115	ENSG00000136710.10	ENSP00000387301.1	45.1	525
EVGPREAGLR	10	52.5000	CCDC115	ENSG00000136710.10	ENSP00000387301.1	45.1	525
RLPSSWALF	9	0.1195	ITFG1	ENSG00000129636.12	ENSP00000319918.6	48.7	1836
RYLTGAWRL	9	0.0240	ALG5	ENSG00000120697.9	ENSP00000239891.3	38.8	972
SYFDEPVEL	9	0.0327	ARFGAP3	ENSG00000242247.11	ENSP00000263245.5	35.9	1548
KFHSEVAKF	9	0.0743	GGA2	ENSG00000103365.15	ENSP00000311962.4	42.6	1839
HMYIFKTL	8	8.0939	UGT8	ENSG00000174607.11	ENSP00000311648.6	37.6	1623
DVVSKFLNR	9	19.4839	PPP1CB	ENSG00000213639.10	ENSP00000351298.2	42.8	981
EVYDIAFSR	9	12.7500	DCAF7	ENSG00000136485.15	ENSP00000483236.1	35.1	1026
RYLPKLKAF	9	0.0169	CRY2	ENSG00000121671.11	ENSP00000478187.1	37.3	1842
GYSEHFVEF	9	0.0065	GMPR	ENSG00000137198.9	ENSP00000259727.4	33.5	1035
AVIHLALKER	10	46.0000	SF3A1	ENSG00000099995.19	ENSP00000215793.7	66.1	2379
EVVEDSLRQR	10	41.5000	TMX4	ENSG00000125827.9	ENSP00000246024.2	33.4	1047
KYPEIKSLM	9	0.0567	DEGS1	ENSG00000143753.13	ENSP00000316476.4	38.5	969
DTISPYSRM	9	6.3865	DEGS1	ENSG00000143753.13	ENSP00000316476.4	38.5	969
HFIAEHYMF	9	0.0437	DEGS1	ENSG00000143753.13	ENSP00000316476.4	38.5	969
KYIPKQSFL	9	0.0167	EMC3	ENSG00000125037.12	ENSP00000245046.2	40.5	783
AYANIISQF	9	0.0035	SLC47A2	ENSG00000180638.17	ENSP00000326671.5	56.3	1806
NYPETLGRLLI	11	0.5073	SEC14L1	ENSG00000129657.16	ENSP00000389838.1	62.0	2043
EVIKNRFHR	9	19.6452	USP19	ENSG00000172046.19	ENSP00000400090.1	49.8	4116
TYARLAQLL	9	0.0278	USP19	ENSG00000172046.19	ENSP00000400090.1	49.8	4116
SYILRPVAF	9	0.0127	SLC28A1	ENSG00000156222.12	ENSP00000286749.3	59.7	1947
QYSDINNRW	9	0.0494	SPTBN2	ENSG00000173898.13	ENSP00000433593.1	48.6	7095
STIGETSNR	9	18.2368	RBM23	ENSG00000100461.18	ENSP00000339220.5	45.9	1215

EQPETYYKW	9	0.2333	INTS1	ENSG00000164880.16	ENSP00000385722.3	41.0	6570
SVIDYQTHFR	10	13.6036	TMED7	ENSG00000134970.14	ENSP00000405926.3	32.2	672
KYIIELNHM	9	0.0526	YPEL3	ENSG00000090238.11	ENSP00000381818.4	49.8	357
KYLDNPNAL	9	0.0499	KANK2	ENSG00000197256.10	ENSP00000465650.1	38.7	2553
EVADPTAHR	9	11.2283	KANK2	ENSG00000197256.10	ENSP00000465650.1	38.7	2553
AYTTVLQEW	9	0.0210	KANK2	ENSG00000197256.10	ENSP00000465650.1	38.7	2553
EVADPTAHRR	10	20.5926	KANK2	ENSG00000197256.10	ENSP00000465650.1	38.7	2553
VYRNKDALSHF	11	0.1064	TIMMDC1	ENSG00000113845.9	ENSP00000418803.1	42.3	855
TQFLPRTI	8	6.9179	PDZD11	ENSG00000120509.10	ENSP00000239666.4	48.0	420
VMLEVLRV	8	15.3913	ERMP1	ENSG00000099219.14	ENSP00000340427.5	38.3	2712
EVFHSGHSR	9	17.8000	FAM102A	ENSG00000167106.12	ENSP00000362187.1	34.3	1152
SQRITNQV	8	27.2000	PSMD7	ENSG00000103035.11	ENSP00000219313.4	74.6	972
KYPASSSVF	9	0.0233	HOXB7	ENSG00000260027.4	ENSP00000239165.7	34.6	651
VYSEVHFTL	9	0.0025	SYNPO	ENSG00000171992.13	ENSP00000302139.4	38.6	2709
EVSGCGSLR	9	37.6667	LRRC47	ENSG00000130764.10	ENSP00000367498.1	33.0	1749
MVVGRGLLGR	10	41.5000	HAGH	ENSG00000063854.13	ENSP00000380514.3	169.5	924
EVIGRLDTM	9	6.7761	ADH1B	ENSG00000196616.14	ENSP00000486614.1	39.1	1005
KYSKIKHLL	9	0.0091	SCNN1B	ENSG00000168447.11	ENSP00000345751.2	32.0	1920
TQYKMTISM	9	3.7106	SCNN1B	ENSG00000168447.11	ENSP00000345751.2	32.0	1920
SYLSHSEQLVF	11	0.0506	TMEM106A	ENSG00000184988.8	ENSP00000483246.1	34.0	786
VYWKIYNSI	9	0.0655	SF3B1	ENSG00000115524.16	ENSP00000335321.5	98.2	3912
YYTREVMLI	9	0.0383	SF3B1	ENSG00000115524.16	ENSP00000335321.5	98.2	3912
GQRYFLNHI	9	9.7647	WWTR1	ENSG00000018408.14	ENSP00000419465.1	46.6	1200
TYLPAPEGLKF	11	0.0056	TNC	ENSG00000041982.16	ENSP00000442242.1	66.6	5514
SYLDNFIKI	9	0.0093	PAK4	ENSG00000130669.17	ENSP00000469413.1	45.3	1773
EVTDYTTGR	9	21.5909	ACSL4	ENSG00000068366.19	ENSP00000262835.7	34.4	2010
VPYPLPKI	8	9.7088	NPEPPS	ENSG00000141279.16	ENSP00000320324.4	52.0	2757

NYADQISRL	9	0.1401	SPAG9	ENSG00000008294.21	ENSP00000349636.4	55.7	3921
VYSEAARVLQF	11	0.0210	GALK2	ENSG00000156958.15	ENSP00000453133.1	37.9	1302
RYLPDTLLL	9	0.0071	COMT	ENSG00000093010.13	ENSP00000354511.6	59.6	813
EIISEVQRM	9	8.8803	ASAP2	ENSG00000151693.11	ENSP00000281419.3	31.9	3018
QYTQALEKF	9	0.0092	ASAP2	ENSG00000151693.11	ENSP00000281419.3	31.9	3018
EYLLKVNEI	9	0.1535	ASAP2	ENSG00000151693.11	ENSP00000281419.3	31.9	3018
VYIDRVRSL	9	0.0290	LMNA	ENSG00000160789.20	ENSP00000357282.3	81.9	1842
AYIADHLGF	9	0.0522	ANK2	ENSG00000145362.18	ENSP00000264366.6	106.3	11772
PYMAKFVVF	9	0.0179	ANK2	ENSG00000145362.18	ENSP00000264366.6	106.3	11772
SYAEIEQTI	9	0.0119	ANK2	ENSG00000145362.18	ENSP00000264366.6	106.3	11772
SYADESFHF	9	0.0034	ANK2	ENSG00000145362.18	ENSP00000264366.6	106.3	11772
QLPDKWQHDL	10	2.4613	ALYREF	ENSG00000183684.7	ENSP00000421592.2	32.5	792
RYQQWMERF	9	0.0050	ELAC2	ENSG00000006744.19	ENSP00000463740.2	47.5	1878
IYIKHPHLF	9	0.0006	DNTTIP1	ENSG00000101457.13	ENSP00000361705.3	34.5	987
ETAGRYITY	9	8.2978	POLR2E	ENSG00000099817.12	ENSP00000478303.1	77.7	630
QGYTVARI	8	30.5000	SEL1L	ENSG00000071537.14	ENSP00000337053.4	36.1	2382
KQALKYFNL	9	1.2698	SEL1L	ENSG00000071537.14	ENSP00000337053.4	36.1	2382
QYAPSTAQF	9	0.0024	ZMIZ2	ENSG00000122515.15	ENSP00000265346.7	54.0	2682
AYHNSPAYL	9	0.1615	SMARCE1	ENSG00000073584.20	ENSP00000464511.1	52.6	1023
NYLVLPNRI	9	0.0900	ESYT2	ENSG00000117868.16	ENSP00000499020.1	36.0	2679
EVIGKLKGL	9	9.4126	SYNE2	ENSG00000054654.16	ENSP00000450831.1	137.7	10623
EVISGYEEL	9	5.1184	CYFIP1	ENSG00000273749.5	ENSP00000478779.1	50.4	3759
LYGKIAEAF	9	0.0168	GIPC1	ENSG00000123159.16	ENSP00000376753.3	96.0	999
VYLGHVIYL	9	0.0162	GJA4	ENSG00000187513.9	ENSP00000343676.4	33.0	999
EQFKFKNMV	9	11.4239	FAM192A	ENSG00000172775.17	ENSP00000457850.1	56.9	762
RYMQWRETI	9	0.0219	ITPKB	ENSG00000143772.9	ENSP00000272117.3	31.4	2838
DVTLGSVLGR	10	42.0000	ACOX1	ENSG00000161533.12	ENSP00000293217.4	38.3	1980

GQWEVKKI	8	18.6053	SEC13	ENSG00000157020.18	ENSP00000312122.4	81.3	966
YYMKDLPTSF	10	0.0052	PI4KA	ENSG00000241973.10	ENSP00000255882.6	42.5	6306
RFIRPMGLRF	10	0.1038	NSA2	ENSG00000164346.10	ENSP00000483484.1	29.1	780
LYLKEVTKF	9	0.0034	RRAGD	ENSG00000025039.14	ENSP00000352131.2	38.7	747
TYMKDLYQL	9	0.0083	ARHGAP18	ENSG00000146376.11	ENSP00000357131.2	28.5	1989
RYDPNIDQW	9	0.0269	KLHL12	ENSG00000117153.16	ENSP00000356230.3	30.2	1704
KYHGYPYSF	9	0.0019	MAGT1	ENSG00000102158.19	ENSP00000478379.1	28.2	1101
RYSSAFTNRIFF	12	0.1923	MAGT1	ENSG00000102158.19	ENSP00000478379.1	28.2	1101
HYAHLIKTF	9	0.0078	DOCK1	ENSG00000150760.12	ENSP00000280333.6	34.2	5595
MYIRYLYKL	9	0.0257	DOCK1	ENSG00000150760.12	ENSP00000280333.6	34.2	5595
QYADMLNKKF	10	0.0440	DOCK1	ENSG00000150760.12	ENSP00000280333.6	34.2	5595
KYMDINFDF	9	0.0063	MYO1D	ENSG00000176658.17	ENSP00000324527.5	35.1	3018
IYASVRTLL	9	0.0337	SH3BP4	ENSG00000130147.16	ENSP00000340237.4	39.1	2889
KYTKIFNDF	9	0.0077	ZMYND11	ENSG00000015171.19	ENSP00000371017.5	55.4	1446
IYQFIMDRF	9	0.0122	FOXC1	ENSG00000054598.9	ENSP00000493906.1	27.7	1659
EQYPGGMAR	9	15.0580	FOXC1	ENSG00000054598.9	ENSP00000493906.1	27.7	1659
KYAMMFAEL	9	0.1095	HUWE1	ENSG00000086758.16	ENSP00000479451.1	50.5	13095
DYILNVMKF	9	0.0438	HUWE1	ENSG00000086758.16	ENSP00000479451.1	50.5	13095
RYTDMESEDYHF	12	0.1578	HUWE1	ENSG00000086758.16	ENSP00000479451.1	50.5	13095
KYAHMINGF	9	0.0156	ADSS	ENSG00000035687.10	ENSP00000355493.3	29.3	1368
KYGPVFSF	8	0.0255	CYP51A1	ENSG00000001630.17	ENSP00000003100.8	29.4	1527
NYIKRNWRI	9	0.0872	CSE1L	ENSG00000124207.17	ENSP00000262982.2	28.2	2913
EYMEHVYLI	9	0.0119	OSBPL2	ENSG00000130703.16	ENSP00000494549.1	38.6	1440
YYEEQHPEL	9	0.1204	IL32	ENSG00000008517.16	ENSP00000433866.3	125.7	663
TFKELANEF	9	0.1342	ERBB3	ENSG00000065361.15	ENSP00000448729.1	75.1	1386
HQLPLPHNV	9	1.7648	HIF1A	ENSG00000100644.17	ENSP00000338018.4	55.1	2478
RYAEEVGIF	9	0.0345	NANS	ENSG00000095380.11	ENSP00000210444.5	32.8	1077

LYGNIIDNL	9	0.1878	IDH3B	ENSG00000101365.21	ENSP00000482773.1	116.5	1161
YYPSTPGRYSI	11	0.1783	FLNB	ENSG00000136068.14	ENSP00000295956.4	154.1	7806
VFVNTHHIL	9	0.2321	B3GNT2	ENSG00000170340.11	ENSP00000305595.4	26.6	1191
RYAQNVAFF	9	0.0039	ZNF32	ENSG00000169740.14	ENSP00000363556.2	48.2	819
EMISDEIHER	10	24.2857	SH3BP5	ENSG00000131370.16	ENSP00000373301.3	45.2	1365
ELVHKETAAR	10	49.0000	SH3BP5	ENSG00000131370.16	ENSP00000373301.3	45.2	1365
KYPNVFKKI	9	0.0209	AHCYL1	ENSG00000168710.18	ENSP00000377238.4	84.5	1449
RYKQDVYLL	9	0.0154	AHCYL1	ENSG00000168710.18	ENSP00000377238.4	84.5	1449
HYLDTTTLI	9	0.0353	CRKL	ENSG00000099942.13	ENSP00000346300.3	26.2	909
VMSQALKATF	10	1.4321	ETS2	ENSG00000157557.13	ENSP00000499540.1	50.3	1512
EYGPVYSTW	9	0.0089	SNX30	ENSG00000148158.17	ENSP00000363349.3	27.0	1311
VYLPNINKI	9	0.0031	RHOBTB1	ENSG00000072422.17	ENSP00000338671.5	35.4	2088
LYPDHFHLL	9	0.0048	PPP5C	ENSG00000011485.14	ENSP00000012443.4	41.8	1497
TEPLPEKTQESL	12	19.4839	TPD52	ENSG00000076554.15	ENSP00000368391.3	63.0	672
ETRAEFAER	9	35.7500	TPM2	ENSG00000198467.15	ENSP00000367542.3	107.2	852
IYGLLKNPF	9	0.1190	YME1L1	ENSG00000136758.18	ENSP00000318480.3	47.3	2319
GQTRLLVV	8	42.0000	SLC9A3R2	ENSG00000065054.14	ENSP00000402857.2	158.8	978
ELIQEITQR	9	20.1111	RDX	ENSG00000137710.16	ENSP00000496414.1	57.2	1812
VYVQNVVKL	9	0.0130	AP3D1	ENSG00000065000.18	ENSP00000495274.1	85.7	3645
EYLTKVDKL	9	0.1835	CLTC	ENSG00000141367.11	ENSP00000479606.1	86.3	5037
IYIDSNNNPERF	12	0.0352	CLTC	ENSG00000141367.11	ENSP00000479606.1	86.3	5037
LYDPVISKL	9	0.0417	MCRS1	ENSG00000187778.14	ENSP00000349640.4	57.9	1425
SVVEIASLR	9	17.9333	MCRS1	ENSG00000187778.14	ENSP00000349640.4	57.9	1425
KYGKDFNDI	9	0.4760	MTA2	ENSG00000149480.7	ENSP00000278823.2	31.6	2004
SYLPSPEKL	9	0.0061	PLCL1	ENSG00000115896.16	ENSP00000402861.1	27.5	3285
KYPVLVERI	9	0.0235	ARHGEF28	ENSG00000214944.9	ENSP00000296799.4	36.4	4176
IYLSKPTHW	9	0.0061	POLR2B	ENSG00000047315.16	ENSP00000491706.1	45.0	3297

VYAILTHGI	9	0.0592	PRPS2	ENSG00000101911.12	ENSP00000370043.5	32.3	954
TYIESASEL	9	0.0940	GALNT14	ENSG00000158089.15	ENSP00000288988.6	86.3	1656
RYKAPFHQL	9	0.0113	SMARCA1	ENSG00000102038.15	ENSP00000360162.4	26.9	3210
RQVKLVNI	8	10.5725	PLEC	ENSG00000178209.15	ENSP00000434583.1	45.3	13710
IYLEKLKTI	9	0.0161	PLEC	ENSG00000178209.15	ENSP00000434583.1	45.3	13710
YVIDPIKGL	9	3.7410	PLEC	ENSG00000178209.15	ENSP00000434583.1	45.3	13710
VYAALQRQLL	10	0.2393	PLEC	ENSG00000178209.15	ENSP00000434583.1	45.3	13710
SQRPIQMV	8	18.9211	EHD3	ENSG00000013016.16	ENSP00000327116.5	25.1	1605
AYVEMLQHL	9	0.0160	FGD5	ENSG00000154783.11	ENSP00000285046.5	26.6	4386
RMITAMNTI	9	2.1085	KEAP1	ENSG00000079999.14	ENSP00000377245.1	36.8	1872
QQQTMLRV	8	28.6250	PACS1	ENSG00000175115.12	ENSP00000316454.4	29.9	2889
KYRDQYNWF	9	0.0904	C1GALT1C1	ENSG00000171155.8	ENSP00000304364.5	25.1	954
VYLPTHTSL	9	0.0083	MYLIP	ENSG00000007944.15	ENSP00000349298.3	28.5	1335
TYQVLAVTF	9	0.0055	SNRNP40	ENSG00000060688.13	ENSP00000263694.4	28.3	1071
KYGMVTYLL	9	0.0229	GPAT4	ENSG00000158669.11	ENSP00000380184.3	62.4	1368
KYGTKIEHF	9	0.0054	SCP2	ENSG00000116171.18	ENSP00000435194.1	142.7	1098
RYVVTSVSW	9	0.0274	EIF3B	ENSG00000106263.18	ENSP00000354125.4	102.4	2442
PEKLATKF	8	19.0000	ECPAS	ENSG00000136813.14	ENSP00000339889.5	37.2	5517
VYPEKLATKF	10	0.0074	ECPAS	ENSG00000136813.14	ENSP00000339889.5	37.2	5517
KYISKPENL	9	0.0178	CAB39	ENSG00000135932.11	ENSP00000258418.5	27.2	1023
KYISKPENLKL	11	0.1414	CAB39	ENSG00000135932.11	ENSP00000258418.5	27.2	1023
VYADQPHIF	9	0.0016	ABHD4	ENSG00000100439.10	ENSP00000414558.2	27.0	1026
KYIHSANVL	9	0.0925	MAPK1	ENSG00000100030.14	ENSP00000215832.6	25.6	1080
ELKQLAAAR	9	38.3333	SPTAN1	ENSG00000197694.15	ENSP00000487444.1	86.7	7494
TQPGTGWVQF	10	0.6774	NECAP2	ENSG00000157191.20	ENSP00000338746.5	36.3	789
EVIEKFDYV	9	9.2330	PMM1	ENSG00000100417.12	ENSP00000216259.7	111.6	786
IQHLIPKI	8	9.3447	PSME2	ENSG00000100911.16	ENSP00000216802.5	70.2	717

VYGSFASKL	9	0.0827	AZIN1	ENSG00000155096.14	ENSP00000337180.5	31.7	1344
VYLIEHPSL	9	0.0372	ASPA	ENSG00000108381.11	ENSP00000263080.2	25.0	939
RYMPFAPAIQTF	12	0.0060	UBA7	ENSG00000182179.13	ENSP00000333266.3	59.8	3036
RYSRKGFLF	9	0.0090	SUPT5H	ENSG00000196235.14	ENSP00000384505.2	85.9	3249
EVTEEGVRFR	10	37.0000	QTRT1	ENSG00000213339.9	ENSP00000250237.4	42.7	1209
SYIAHLRQL	9	0.0534	TCF21	ENSG00000118526.6	ENSP00000356857.4	41.9	537
DVRDRMIHR	9	26.4000	ABLIM1	ENSG00000099204.20	ENSP00000376682.4	56.9	2154
AVVGPDMKRR	10	40.5000	ABLIM1	ENSG00000099204.20	ENSP00000376682.4	56.9	2154
RYVIIPTTF	9	0.0029	CAPN5	ENSG00000149260.18	ENSP00000498132.1	28.4	1920
QYQKILERL	9	0.0217	TAB1	ENSG00000100324.14	ENSP00000216160.6	25.4	1512
KYGVHEAIF	9	0.0971	PPARD	ENSG00000112033.14	ENSP00000310928.4	26.6	1323
NYAPAFTML	9	0.0303	TTC37	ENSG00000198677.11	ENSP00000351596.2	28.3	4692
RYPSLWRRL	9	0.0443	SMARCB1	ENSG00000099956.19	ENSP00000383984.3	37.4	1128
YYDPKHVIF	9	0.0039	ABCF3	ENSG00000161204.11	ENSP00000411471.2	58.9	2127
RYFHWKMNL	9	0.0632	GPR89A	ENSG00000117262.19	ENSP00000432248.1	38.8	1290
RYDEIRRHW	9	0.0370	RPL7A	ENSG00000148303.17	ENSP00000361071.1	1509.0	453
NYNDRYDEI	9	0.3807	RPL7A	ENSG00000148303.17	ENSP00000361071.1	1509.0	453
SFLKAKVI	8	4.5604	RBM42	ENSG00000126254.12	ENSP00000262633.3	57.3	1440
VYITRAQLM	9	0.0807	NACC1	ENSG00000160877.6	ENSP00000292431.3	27.9	1581
KYVDKLEKI	9	0.0196	USP5	ENSG00000111667.13	ENSP00000373883.5	44.8	2505
RYPPDIRATF	10	0.0042	SBF1	ENSG00000100241.21	ENSP00000370196.2	33.1	5679
STGISYETL	9	3.8540	ATXN7L3	ENSG00000087152.15	ENSP00000467092.1	35.8	558
SYTPVLNQF	9	0.0012	UNC13B	ENSG00000198722.14	ENSP00000367756.3	35.9	4773
LYQHEINLF	9	0.0061	CEBPZ	ENSG00000115816.15	ENSP00000234170.5	23.7	3162
TYISKTIAL	9	0.0533	EXOC3L2	ENSG00000283632.2	ENSP00000400713.2	23.3	2406
EYPRETFSF	9	0.0083	DENND2D	ENSG00000162777.17	ENSP00000358767.5	31.1	1404
EQYYVRKV	8	22.1053	BICD2	ENSG00000185963.14	ENSP00000364662.3	24.5	2472

HYMSINDSF	9	0.0118	BICD2	ENSG00000185963.14	ENSP00000364662.3	24.5	2472
QYLPHVARL	9	0.0178	SURF4	ENSG00000148248.14	ENSP00000361057.3	119.2	807
VYPYKLYRL	9	0.0213	ZBTB38	ENSG00000177311.11	ENSP00000372635.5	37.0	3585
KYPNVDARL	9	0.1058	UGCG	ENSG00000148154.10	ENSP00000363397.3	23.8	1182
RWMSQHNRF	9	0.0504	PAFAH1B2	ENSG00000168092.14	ENSP00000435289.1	24.4	687
YSAPATLSSR	10	24.7143	PDLIM2	ENSG00000120913.23	ENSP00000477546.1	105.5	438
YYLTHGLYL	9	0.0587	PIGU	ENSG00000101464.10	ENSP00000217446.3	24.2	1305
GQLKSLTI	8	15.6087	DNAJB6	ENSG00000105993.15	ENSP00000397556.2	57.8	723
KYCYLQNEVF	10	0.1894	DSP	ENSG00000096696.14	ENSP00000369129.3	29.4	8613
SYSGTFHSL	9	0.0117	NBPF1	ENSG00000219481.10	ENSP00000474456.1	41.8	3417
VFTPVVQRI	9	0.0740	CMIP	ENSG00000153815.16	ENSP00000446100.2	30.1	2319
QYRIDFEQF	9	0.0666	TBC1D9	ENSG00000109436.8	ENSP00000411197.2	22.8	3798
VYLHLRQTW	9	0.0164	SLC44A2	ENSG00000129353.15	ENSP00000466664.1	124.8	2133
AYLDALQTL	9	0.0105	KANSL3	ENSG00000114982.19	ENSP00000499674.1	29.8	2712
PYNPLWAQL	9	0.0583	FNIP2	ENSG00000052795.13	ENSP00000264433.6	38.3	3342
AQAEALRI	8	19.1613	CBX6	ENSG00000183741.11	ENSP00000384490.3	24.1	1236
RYNPRILFQL	10	0.0938	WASHC5	ENSG00000164961.16	ENSP00000429676.1	30.9	3033
RYTDVSTRY	9	0.3363	TMX2	ENSG00000213593.10	ENSP00000278422.4	91.8	888
KYVDPICTF	9	0.0059	SLC30A2	ENSG00000158014.14	ENSP00000363394.3	31.7	1116
RYTAILNQI	9	0.0441	COL14A1	ENSG00000187955.12	ENSP00000311809.4	37.9	5340
EVIEAVRNL	9	5.3436	COL14A1	ENSG00000187955.12	ENSP00000311809.4	37.9	5340
GQFKIGLI	8	21.0000	VPS29	ENSG00000111237.18	ENSP00000353786.7	69.3	558
EYLDRAEKL	9	0.2097	VPS4B	ENSG00000119541.10	ENSP00000238497.4	26.6	1332
LYQHAVQYF	9	0.0039	VPS4B	ENSG00000119541.10	ENSP00000238497.4	26.6	1332
EVIKNFIQY	9	5.4624	EIF3L	ENSG00000100129.18	ENSP00000499067.1	246.8	1692
KYGPVVSLL	9	0.0049	UHMK1	ENSG00000152332.16	ENSP00000420270.1	22.5	1257
RYGDVFQI	8	0.3817	CYP1B1	ENSG00000138061.12	ENSP00000478561.1	37.2	1629

KYREMYEEF	9	0.0122	MTREX	ENSG00000039123.16	ENSP00000230640.5	27.8	3126
EYMLEKSFYQF	11	0.1285	MTREX	ENSG00000039123.16	ENSP00000230640.5	27.8	3126
EQLSILKV	8	22.4211	MYH11	ENSG00000133392.18	ENSP00000498314.1	70.5	1905
VYVVGTAHF	9	0.0062	TRABD	ENSG00000170638.9	ENSP00000379171.1	30.1	1128
RQALMPTL	8	7.1087	EP300	ENSG00000100393.13	ENSP00000263253.7	23.8	7242
SYLDSVHFF	9	0.0010	EP300	ENSG00000100393.13	ENSP00000263253.7	23.8	7242
VYLHDFQRF	9	0.0021	PLCG2	ENSG00000197943.10	ENSP00000482457.1	33.0	3795
KYMQMNHALF	10	0.0136	PLCG2	ENSG00000197943.10	ENSP00000482457.1	33.0	3795
IYDPNLAFLRF	11	0.0338	PLCG2	ENSG00000197943.10	ENSP00000482457.1	33.0	3795
KYSLVKNKI	9	0.0825	S100A16	ENSG00000188643.11	ENSP00000357692.1	53.3	309
YYIRGATTTF	10	0.0094	DPT	ENSG00000143196.5	ENSP00000356791.3	21.6	603
RYGIHPAKF	9	0.0094	VWA8	ENSG00000102763.18	ENSP00000368612.3	22.5	5715
GYPSRASFHEL	11	0.4562	MYO6	ENSG00000196586.15	ENSP00000358992.1	37.7	3759
RYEPANSHFF	10	0.0169	WDFY3	ENSG00000163625.15	ENSP00000295888.4	34.3	10578
RYSVASFKF	9	0.0048	MYORG	ENSG00000164976.9	ENSP00000297625.8	21.5	2142
IQTKALQV	8	20.0000	AP2A1	ENSG00000196961.12	ENSP00000346246.4	46.0	2865
EYIPDLYNHF	10	0.0109	RABGAP1	ENSG00000011454.17	ENSP00000362751.4	28.2	3207
TYLPDHMTL	9	0.0055	AQP7	ENSG00000165269.13	ENSP00000297988.1	52.9	1026
KYDEIFYNL	9	0.0149	EHD2	ENSG00000024422.12	ENSP00000263277.2	22.7	1629
KYFAKHPRL	9	0.0541	UTRN	ENSG00000152818.18	ENSP00000356515.3	32.3	10299
EIFDGNVAHI	10	7.8626	UTRN	ENSG00000152818.18	ENSP00000356515.3	32.3	10299
KYQELQVLF	9	0.0015	VPS37B	ENSG00000139722.7	ENSP00000267202.2	21.9	855
RYLRVEHHF	9	0.0028	ACACB	ENSG00000076555.15	ENSP00000367079.3	38.8	7374
RYFQPPGTW	9	0.0260	NMD3	ENSG00000169251.12	ENSP00000419004.1	25.7	1509
KYGRIVEVL	9	0.0696	RBMX	ENSG00000147274.14	ENSP00000405117.2	69.2	876
HGMIMLRI	8	23.6875	ARCN1	ENSG00000095139.14	ENSP00000264028.4	31.3	1533
QQSVLQRI	8	15.8406	SMC1A	ENSG00000072501.17	ENSP00000476416.1	40.7	837

EYIDSPVIL	9	0.1151	PKHD1	ENSG00000170927.14	ENSP00000360158.3	20.6	12222
DYTGALAVF	9	0.1970	F8A1	ENSG00000277203.1	ENSP00000479624.1	20.4	1113
KYSEVFEAI	9	0.0179	CSNK2A1	ENSG00000101266.19	ENSP00000494445.1	32.4	264
FFKPHWDEKF	10	0.2620	SERPINH1	ENSG00000149257.14	ENSP00000434412.1	29.4	1254
AYVVKVFSL	9	0.0716	C3	ENSG00000125730.16	ENSP00000245907.4	22.8	4989
HWESASLLR	9	8.6555	C3	ENSG00000125730.16	ENSP00000245907.4	22.8	4989
RIHWESASLLR	11	18.0263	C3	ENSG00000125730.16	ENSP00000245907.4	22.8	4989
HRIHWESASLLR	12	27.6000	C3	ENSG00000125730.16	ENSP00000245907.4	22.8	4989
LYIDEAHSI	9	0.0215	SPTLC2	ENSG00000100596.6	ENSP00000216484.2	20.9	1686
LYMRIRDNW	9	0.0883	SPTLC2	ENSG00000100596.6	ENSP00000216484.2	20.9	1686
RYLTRGYSL	9	0.0444	FLT1	ENSG00000102755.12	ENSP00000282397.4	29.4	4014
KYGNLSNYL	9	0.1072	FLT1	ENSG00000102755.12	ENSP00000282397.4	29.4	4014
AYAQEIQKL	9	0.0248	NFIL3	ENSG00000165030.4	ENSP00000297689.2	20.2	1386
RYYGNISRF	9	0.0041	EHMT2	ENSG00000204371.11	ENSP00000364687.4	33.1	3630
RYLDELMKL	9	0.0132	IQGAP1	ENSG00000140575.13	ENSP00000488618.1	49.4	3573
KYGIQMPAF	9	0.0474	IQGAP1	ENSG00000140575.13	ENSP00000488618.1	49.4	3573
RYRDRLQYF	9	0.0357	IQGAP1	ENSG00000140575.13	ENSP00000488618.1	49.4	3573
YFHPPAHL	8	0.8542	MBNL1	ENSG00000152601.17	ENSP00000418427.1	34.7	906
KYFHPPAHL	9	0.0180	MBNL1	ENSG00000152601.17	ENSP00000418427.1	34.7	906
KYLHPPPHL	9	0.0152	MBNL1	ENSG00000152601.17	ENSP00000418427.1	34.7	906
QYSRFSLENNF	11	0.2339	STAT1	ENSG00000115415.18	ENSP00000438703.1	45.4	579
EYAVLTSTI	9	0.1604	IRF2	ENSG00000168310.11	ENSP00000377218.3	21.4	1047
RYITKGNLF	9	0.0081	PPP4R3B	ENSG00000275052.5	ENSP00000484116.1	34.0	2451
NQYRLIVNV	9	8.1306	MCM3	ENSG00000112118.19	ENSP00000472940.2	28.5	2424
EVTGFGVTR	9	23.1250	KLC4	ENSG00000137171.15	ENSP00000418546.1	79.1	135
IYGYVAEQF	9	0.0035	SPCS1	ENSG00000114902.14	ENSP00000478310.2	66.0	306
ETILRTNKR	9	28.3750	SH3KBP1	ENSG00000147010.18	ENSP00000369020.4	24.1	1884

FVIDAVRTK	9	21.2273	EIF3M	ENSG00000149100.13	ENSP00000436049.1	34.8	1122
ETYKKMLAR	9	17.3111	RCN3	ENSG00000142552.8	ENSP00000270645.2	24.5	984
SVVGFLSQR	9	13.2613	DCAF5	ENSG00000139990.17	ENSP00000451845.1	35.7	2823
SYVQVTSNF	9	0.0048	AFF4	ENSG00000072364.13	ENSP00000265343.5	21.8	3489
HYLQEAKKL	9	0.2118	AFF4	ENSG00000072364.13	ENSP00000265343.5	21.8	3489
KYVESFRRF	9	0.0054	HNMT	ENSG00000150540.14	ENSP00000280097.3	81.7	876
KYMSVIAEL	9	0.0139	ATPAF1	ENSG00000123472.12	ENSP00000460964.1	34.6	1053
VYAVIPAEKF	10	0.0179	ATPAF1	ENSG00000123472.12	ENSP00000460964.1	34.6	1053
WQAKVPQI	8	6.7285	DYNC1H1	ENSG00000197102.12	ENSP00000348965.4	69.6	13938
TQRVISFI	8	22.2105	DYNC1H1	ENSG00000197102.12	ENSP00000348965.4	69.6	13938
RYKLYQEMF	9	0.0326	DYNC1H1	ENSG00000197102.12	ENSP00000348965.4	69.6	13938
ETFDFQAMGR	10	23.7500	DYNC1H1	ENSG00000197102.12	ENSP00000348965.4	69.6	13938
LYTEKFEEF	9	0.0048	TXLNA	ENSG00000084652.16	ENSP00000362712.3	24.5	1638
EAIQRTEAY	9	12.6667	SEC16A	ENSG00000148396.18	ENSP00000403525.1	31.5	3177
KYLDYMQKI	9	0.0092	LACTB	ENSG00000103642.12	ENSP00000261893.4	24.7	1641
VYMNVMTRL	9	0.0185	PPP2R3A	ENSG00000073711.11	ENSP00000264977.3	20.2	3450
SYMWTINNF	9	0.0050	SPOP	ENSG00000121067.19	ENSP00000499367.1	30.9	495
TYQSITERI	9	0.0213	EXOC4	ENSG00000131558.15	ENSP00000253861.4	26.9	2922
SYSMIVNNL	9	0.0581	CACYBP	ENSG00000116161.17	ENSP00000356652.2	28.6	684
AYPHRLNLF	9	0.0134	RPRD2	ENSG00000163125.15	ENSP00000358064.4	23.6	4383
KYLTEGLLQF	10	0.0110	NFE2L1	ENSG00000082641.16	ENSP00000355190.3	119.3	2283
KQVQQPSV	8	21.2273	WTAP	ENSG00000146457.16	ENSP00000336911.4	34.0	453
VYIKHLRDI	9	0.1886	SPATA13	ENSG00000182957.16	ENSP00000371527.4	26.1	1956
KYRRYHPLF	9	0.1047	ARAP1	ENSG00000186635.14	ENSP00000335506.8	60.2	3615
NYLSHHLTI	9	0.0216	PAIP1	ENSG00000172239.14	ENSP00000425675.1	48.5	600
SFSPKTYSF	9	0.0057	PIK3C2A	ENSG00000011405.13	ENSP00000265970.6	21.9	5058
SYEYRFLEF	9	0.0483	RFTN1	ENSG00000131378.14	ENSP00000334153.4	20.0	1734

YYQNYFEKL	9	0.0119	PATL1	ENSG00000166889.14	ENSP00000300146.9	19.7	2310
EVNPNTRVM	9	7.9958	ORMDL3	ENSG00000172057.10	ENSP00000304858.2	25.0	459
YYEYNHDLF	9	0.0995	SAV1	ENSG00000151748.14	ENSP00000324729.4	23.1	1149
VFYAVKVL	8	3.5233	SGK1	ENSG00000118515.11	ENSP00000396242.3	44.8	1335
SYAPPTDSF	9	0.0041	SGK1	ENSG00000118515.11	ENSP00000396242.3	44.8	1335
KYLTAEAFGF	10	0.1492	FSCN1	ENSG00000075618.18	ENSP00000371798.3	23.9	1479
MQQILTRV	8	18.4737	GNL2	ENSG00000134697.13	ENSP00000362153.3	22.2	2193
EYISKTYKI	9	0.0142	GNL2	ENSG00000134697.13	ENSP00000362153.3	22.2	2193
EVGPKFRGV	9	16.8545	AAR2	ENSG00000131043.12	ENSP00000363043.3	25.5	1152
EVIDLMIKEY	10	25.6667	PHF10	ENSG00000130024.15	ENSP00000355743.4	27.8	1488
RYIRDAHTF	9	0.0026	ARHGAP35	ENSG00000160007.18	ENSP00000385720.2	19.5	4497
VYGKEGLVHSF	11	0.0370	RHCG	ENSG00000140519.14	ENSP00000453496.1	109.7	711
EVINRSMDTY	10	26.1000	CAMLG	ENSG00000164615.5	ENSP00000297156.2	26.6	888
KQLSILKV	8	14.7556	SCRIB	ENSG00000180900.19	ENSP00000366756.3	32.4	4647
SYMLVNENRF	10	0.0500	LDOC1	ENSG00000182195.8	ENSP00000359557.2	24.0	438
HYNSDLNNLLF	11	0.1085	RHOBTB3	ENSG00000164292.13	ENSP00000369318.3	22.6	1833
VYSSAEFHSL	10	0.1182	LTBP3	ENSG00000168056.16	ENSP00000435530.1	115.7	2199
QYSDVNNRW	9	0.0627	SPTBN1	ENSG00000115306.16	ENSP00000349259.4	224.4	7092
YYLGNPAEF	9	0.0034	RCN1	ENSG00000049449.9	ENSP00000054950.3	33.3	993
YIKQIKTF	8	0.4863	RBL2	ENSG00000103479.16	ENSP00000262133.6	58.9	3417
IYIKQIKTF	9	0.0027	RBL2	ENSG00000103479.16	ENSP00000262133.6	58.9	3417
EYLRFLQTI	9	0.0273	ITPR2	ENSG00000123104.12	ENSP00000370744.3	19.5	8103
RQYLAINQI	9	2.0411	ITPR2	ENSG00000123104.12	ENSP00000370744.3	19.5	8103
QYPVIIHLI	9	0.0054	ELOVL1	ENSG00000066322.15	ENSP00000361536.3	55.3	837
HQFQCSRL	8	20.5556	BABAM2	ENSG00000158019.20	ENSP00000343412.2	43.8	1245
DISHIGSMR	9	42.0000	H2AFY2	ENSG00000099284.14	ENSP00000362352.3	19.0	1116
NYLSIFRKF	9	0.0064	CAPN1	ENSG00000014216.15	ENSP00000279247.6	98.5	2142

NYFRGYEW	8	0.3996	GALC	ENSG00000054983.17	ENSP00000261304.2	23.2	2055
SQFELLKV	8	14.7222	RPS6KA2	ENSG00000071242.12	ENSP00000265678.4	19.1	2199
VYAQVARLF	9	0.0034	SIN3A	ENSG00000169375.15	ENSP00000378403.4	22.9	3819
EVIHRKALQR	10	46.0000	SIN3A	ENSG00000169375.15	ENSP00000378403.4	22.9	3819
EYVNDFDLLKF	11	0.2270	MAFB	ENSG00000204103.4	ENSP00000362410.2	18.2	969
YPDGIRHI	8	2.2267	SF3B3	ENSG00000189091.13	ENSP00000305790.5	23.4	3651
VYPDGIRHI	9	0.0220	SF3B3	ENSG00000189091.13	ENSP00000305790.5	23.4	3651
NYISGIQTI	9	0.0088	SF3B3	ENSG00000189091.13	ENSP00000305790.5	23.4	3651
RTFAHYATF	9	0.3022	FCN3	ENSG00000142748.12	ENSP00000270879.4	23.8	897
FYLEGGFSKF	10	0.0183	DUSP6	ENSG00000139318.8	ENSP00000279488.6	22.3	1143
SYLPFTEAF	9	0.0021	SELENON	ENSG00000162430.17	ENSP00000355141.2	26.6	1770
YYVRILSTI	9	0.0342	PSMC4	ENSG00000013275.7	ENSP00000413869.1	112.1	1161
RYRPQHIFF	9	0.0206	CYB561	ENSG00000008283.16	ENSP00000376702.1	48.0	753
KYMPNVKVAVF	11	0.0325	DDX39B	ENSG00000198563.14	ENSP00000399371.1	153.2	867
HYGKVYLL	8	0.3747	VPS41	ENSG00000006715.16	ENSP00000309457.4	21.1	2562
RYVEIVSQF	9	0.0015	VPS41	ENSG00000006715.16	ENSP00000309457.4	21.1	2562
YQHLLVNSI	9	2.7948	ARMCX2	ENSG00000184867.14	ENSP00000328631.2	27.9	1896
RYLVEVEEL	9	0.0556	PDRG1	ENSG00000088356.6	ENSP00000202017.4	17.8	399
QQWPVRSI	8	8.4347	XPO6	ENSG00000169180.11	ENSP00000302790.4	30.9	3375
EVFANRFTQM	10	7.5680	DENND5A	ENSG00000184014.8	ENSP00000435866.1	28.4	3723
EYLIVFPKL	9	0.0837	MARCH5	ENSG00000198060.10	ENSP00000351813.2	20.1	834
EYAKIEETM	9	0.2458	CADPS2	ENSG00000081803.16	ENSP00000400401.2	24.3	3765
RYGSDFYSM	9	0.0649	AMZ2	ENSG00000196704.12	ENSP00000464133.1	47.8	1080
IYQDSFEQRF	10	0.0057	CUL4B	ENSG00000158290.16	ENSP00000360373.5	19.2	2685
NYQKRFQNL	9	0.0611	OGFR	ENSG00000060491.16	ENSP00000359491.1	34.3	1875
ETVEKFRQR	9	16.7636	COMMD10	ENSG00000145781.9	ENSP00000488332.1	20.7	564
KWLTKSFHF	9	0.0244	SLC35C2	ENSG00000080189.15	ENSP00000318960.9	32.4	1182

KYQDILNEI	9	0.0182	IQGAP2	ENSG00000145703.16	ENSP00000274364.6	63.1	4725
SYLSRLQYF	9	0.0028	IQGAP2	ENSG00000145703.16	ENSP00000274364.6	63.1	4725
TYPEVKNKL	9	0.0417	IQGAP2	ENSG00000145703.16	ENSP00000274364.6	63.1	4725
DVYDRKNIPR	10	17.8222	IQGAP2	ENSG00000145703.16	ENSP00000274364.6	63.1	4725
ETLEGWKATRR	11	51.2500	DDN	ENSG00000181418.8	ENSP00000390590.2	17.5	2133
RYMILGQNGF	10	0.0911	PGM5	ENSG00000154330.13	ENSP00000379678.1	19.0	1701
ETPSAPLLSR	10	24.0714	PNPLA6	ENSG00000032444.16	ENSP00000473211.1	58.0	4095
KYIQGLKRL	9	0.0754	PRPF18	ENSG00000165630.14	ENSP00000367835.3	19.1	1026
EVAQFLTGR	9	20.5556	DESI1	ENSG00000100418.8	ENSP00000263256.6	18.7	504
IYYIDMQKF	9	0.0056	KCTD3	ENSG00000136636.13	ENSP00000259154.2	18.3	2445
SYNEHWNYL	9	0.0269	AC005840.1	ENSG00000111321.11	ENSP00000228918.4	65.2	1305
SYITEECLKKL	11	0.8048	UBE3C	ENSG00000009335.18	ENSP00000309198.8	19.3	3249
AYNPMARDLF	10	0.0647	MTOR	ENSG00000198793.12	ENSP00000354558.4	32.1	7647
DIIADISNR	9	33.0000	SCIN	ENSG00000006747.15	ENSP00000297029.5	30.6	2145
TYPRGQIIYTW	11	0.0825	SCIN	ENSG00000006747.15	ENSP00000297029.5	30.6	2145
RYVMTTTTL	9	0.0529	EIF2S1	ENSG00000134001.13	ENSP00000256383.4	18.4	945
VYIGKLNMI	9	0.0190	XPO1	ENSG00000082898.16	ENSP00000385942.2	33.2	3213
QYEEQFVTL	9	0.0796	XPO1	ENSG00000082898.16	ENSP00000385942.2	33.2	3213
VYLAAVNRL	9	0.0222	PLXND1	ENSG00000004399.12	ENSP00000317128.4	37.0	5775
HQITVLHV	8	19.0968	ELOVL5	ENSG00000012660.14	ENSP00000306640.6	39.2	897
KYITQGQLL	9	0.0246	ELOVL5	ENSG00000012660.14	ENSP00000306640.6	39.2	897
KYITQGQLLQF	11	0.0263	ELOVL5	ENSG00000012660.14	ENSP00000306640.6	39.2	897
NYAWVYYHL	9	0.0406	IFIT3	ENSG00000119917.14	ENSP00000360883.4	22.1	1470
SYFEHNLETW	10	0.0226	GNL1	ENSG00000204590.12	ENSP00000365806.3	25.4	1821
YTDVKHRW	8	1.2624	DNASE1L3	ENSG00000163687.13	ENSP00000378053.2	38.9	915
PYMDSPQSIGF	11	0.0434	PCMT1	ENSG00000120265.17	ENSP00000356354.3	33.8	684
AQLAVTKI	8	17.6000	UBR7	ENSG00000012963.15	ENSP00000013070.6	17.8	1275

KYNDNFFGL	9	0.0439	UBR7	ENSG00000012963.15	ENSP00000013070.6	17.8	1275
VWNPRTHQF	9	0.0075	ATP1B2	ENSG00000129244.9	ENSP00000250111.4	16.7	870
KYTKNIVSW	9	0.0096	ARHGAP29	ENSG00000137962.13	ENSP00000260526.6	18.9	3783
EVFDHMMKR	9	13.0631	USP9X	ENSG00000124486.13	ENSP00000367558.2	24.1	7662
RYMPDICVI	9	0.1224	USP9X	ENSG00000124486.13	ENSP00000367558.2	24.1	7662
EVFDHMMKRM	10	14.7556	USP9X	ENSG00000124486.13	ENSP00000367558.2	24.1	7662
YQIAVTKV	8	8.7479	SMC1A	ENSG00000072501.17	ENSP00000323421.3	40.7	3699
VYLTDPQGL	9	0.1031	FCSK	ENSG00000157353.17	ENSP00000288078.6	25.6	3252
VYNHRFHKI	9	0.0309	USP4	ENSG00000114316.12	ENSP00000341028.4	35.8	2748
VFTEVANLF	9	0.0222	SIN3B	ENSG00000127511.9	ENSP00000248054.4	42.4	3390
TYLDKVENI	9	0.0127	IFIT1	ENSG00000185745.10	ENSP00000360869.3	16.7	1434
NYTDIKDYL	9	0.2450	NUDT12	ENSG00000112874.10	ENSP00000230792.2	18.9	1386
RYQTQPVTL	9	0.0166	SVIL	ENSG00000197321.14	ENSP00000364549.3	50.4	5364
VYNSEYYHF	9	0.0011	PTK7	ENSG00000112655.16	ENSP00000418754.1	22.0	3234
VFIDKQTNL	9	0.2593	CELF1	ENSG00000149187.18	ENSP00000378705.2	29.3	1455
PYYPSPGVLF	10	0.0136	HP1BP3	ENSG00000127483.18	ENSP00000364142.2	98.8	1203
AWPKVLKI	8	1.0777	EPB41L3	ENSG00000082397.17	ENSP00000442091.2	76.1	2595
HQLILSKL	8	12.3333	SMAP1	ENSG00000112305.14	ENSP00000313382.5	20.1	1320
EVFDFRGMRL	10	8.8067	NCKAP1	ENSG00000061676.15	ENSP00000355348.3	19.8	3384
RYASINTHL	9	0.0285	CSNK1E	ENSG00000213923.12	ENSP00000380044.1	76.8	1248
TYKVISNEF	9	0.0172	UBE3A	ENSG00000114062.20	ENSP00000486349.1	25.0	2556
VYSRDPNYL	9	0.0845	UBE3A	ENSG00000114062.20	ENSP00000486349.1	25.0	2556
ELIELRELGAAR	12	56.6667	SMU1	ENSG00000122692.9	ENSP00000380336.3	15.5	1539
RYVPRASYF	9	0.0061	CCND3	ENSG00000112576.12	ENSP00000397545.2	31.7	660
ETTSIATAR	9	32.0000	ZMYM3	ENSG00000147130.14	ENSP00000322845.5	19.0	4110
EVFDERAANF	10	2.5226	VCL	ENSG00000035403.17	ENSP00000361841.3	26.8	3198
PYNPIIGETF	10	0.0216	OSBPL8	ENSG00000091039.17	ENSP00000478240.1	28.2	2541

KYFAQALKL	9	0.0348	EMC2	ENSG00000104412.8	ENSP00000220853.3	19.1	891
ETVNLRSLGF	10	10.4237	CRYBG1	ENSG00000112297.15	ENSP00000358062.3	16.1	5169
SYQKVMALW	9	0.0167	MACF1	ENSG00000127603.26	ENSP00000499399.1	88.3	16137
RYQDIIHSI	9	0.0020	BAZ1B	ENSG00000009954.11	ENSP00000385442.1	22.9	4449
SYAETPLQL	9	0.0237	ABTB2	ENSG00000166016.6	ENSP00000410157.2	15.3	3075
EVISLINTR	9	18.1579	FAF2	ENSG00000113194.13	ENSP00000261942.6	16.6	1335
AYLDKSPQF	9	0.0061	DHX29	ENSG00000067248.10	ENSP00000481966.1	21.5	4110
IYFQAPVKI	9	0.0096	DHX29	ENSG00000067248.10	ENSP00000481966.1	21.5	4110
VYLRETTLI	9	0.0162	DHX29	ENSG00000067248.10	ENSP00000481966.1	21.5	4110
SYLERYQRF	9	0.0021	TNK2	ENSG00000061938.18	ENSP00000329425.6	17.0	3114
DIADFFTTR	9	15.4493	RIPOR3	ENSG00000042062.12	ENSP00000045083.2	43.1	2838
FYMDTSHLF	9	0.0010	PRRC2C	ENSG00000117523.16	ENSP00000410219.3	27.8	8451
EVFPEHLARF	10	0.4834	LATS2	ENSG00000150457.9	ENSP00000372035.4	15.7	3264
KYVENFGLI	9	0.1207	SPCS2	ENSG00000118363.12	ENSP00000263672.6	35.8	678
VGYLVRLV	8	26.9000	FZD8	ENSG00000177283.7	ENSP00000363826.1	15.0	2082
EVHRANITQSR	11	31.6667	FRMD3	ENSG00000172159.16	ENSP00000303508.3	18.2	1791
AYVEKVERL	9	0.0429	AMOTL2	ENSG00000114019.14	ENSP00000424765.1	41.5	2511
KYLEERAMRQF	11	0.0864	AMOTL2	ENSG00000114019.14	ENSP00000424765.1	41.5	2511
KYLGQLHYL	9	0.0108	PTPN23	ENSG00000076201.15	ENSP00000265562.4	18.0	4908
KYMEVHEKASF	11	0.0153	PTPN23	ENSG00000076201.15	ENSP00000265562.4	18.0	4908
IYQREWQRF	9	0.0043	ACTR8	ENSG00000113812.14	ENSP00000419429.1	20.6	1539
EVLKETVSQR	10	36.6667	DCTN1	ENSG00000204843.12	ENSP00000386406.1	63.6	3708
IYSTPLPEKF	10	0.0051	KLF3	ENSG00000109787.13	ENSP00000261438.5	17.4	1035
TYNPNMPFKW	10	0.0422	CCAR1	ENSG00000060339.14	ENSP00000438610.1	41.8	2316
IYIDRFEDL	9	0.0541	ARL14EP	ENSG00000152219.5	ENSP00000282032.3	15.9	780
QYLSSIQHL	9	0.0080	PEX3	ENSG00000034693.15	ENSP00000356563.4	15.4	1119
SYLDSIHFF	9	0.0009	CREBBP	ENSG00000005339.14	ENSP00000262367.5	25.2	7326

KYMDIEFDF	9	0.0087	MYO1B	ENSG00000128641.19	ENSP00000341903.4	25.5	3234
VYTKANPTF	9	0.0032	SPRED2	ENSG00000198369.10	ENSP00000348753.4	17.0	1254
RFIQLLKV	8	4.9357	ABCD4	ENSG00000119688.21	ENSP00000436782.1	52.9	168
YYTPITPHL	9	0.0047	PTBP3	ENSG00000119314.15	ENSP00000363375.1	20.2	1572
PYPPPPPEF	9	0.0069	WBP2	ENSG00000132471.12	ENSP00000466999.1	80.2	693
HGLEMRTI	8	22.2632	JADE2	ENSG00000043143.20	ENSP00000378451.1	38.4	2370
RYLDFKVTEGSF	12	0.1051	GDI2	ENSG00000057608.17	ENSP00000476707.1	178.6	390
KYLESVKPF	9	0.0194	CROT	ENSG00000005469.11	ENSP00000331981.3	17.0	1836
NYNDVYSAF	9	0.0197	RASL11B	ENSG00000128045.7	ENSP00000248706.3	14.8	744
VYRQDCETF	9	0.0353	PPHLN1	ENSG00000134283.17	ENSP00000447168.1	23.9	1155
AFKHKHHLI	9	0.6469	ZEB2	ENSG00000169554.21	ENSP00000302501.4	33.9	3639
AYRRIFQKF	9	0.0296	MYO1E	ENSG00000157483.8	ENSP00000288235.4	15.5	3324
RYAILTKATW	10	0.1595	MYO1E	ENSG00000157483.8	ENSP00000288235.4	15.5	3324
LYSSKLYRF	9	0.0036	KCTD20	ENSG00000112078.14	ENSP00000412205.2	22.3	759
YYSPHGHIL	9	0.0164	EIF2A	ENSG00000144895.12	ENSP00000417229.1	36.5	1755
YYSPHGHILVL	11	0.1875	EIF2A	ENSG00000144895.12	ENSP00000417229.1	36.5	1755
VYLDVQEKW	9	0.0052	DNASE1	ENSG00000213918.10	ENSP00000385905.1	51.3	846
AYGASFLSF	9	0.0097	TMEM69	ENSG00000159596.7	ENSP00000361095.4	18.7	741
KYIQRQETI	9	0.0202	MX1	ENSG00000157601.14	ENSP00000381599.3	20.0	1986
EYLSRHWLF	9	0.0066	GGCX	ENSG00000115486.12	ENSP00000233838.3	19.4	2274
KYLDGLDVCRF	11	0.0759	GGCX	ENSG00000115486.12	ENSP00000233838.3	19.4	2274
EVIQAYETL	9	3.3659	SYMPK	ENSG00000125755.18	ENSP00000478599.1	42.4	3174
SYLHEPAVL	9	0.0862	MYO5B	ENSG00000167306.20	ENSP00000285039.6	21.4	5544
RYKEKVAEL	9	0.0759	KAT7	ENSG00000136504.12	ENSP00000409477.2	19.3	1275
AYKKTHETL	9	0.0673	TPD52L1	ENSG00000111907.21	ENSP00000357387.5	46.9	432
SQRFTVQI	8	14.4444	TAF9B	ENSG00000187325.5	ENSP00000339917.5	14.0	753
KYTTLIAKL	9	0.0439	TES	ENSG00000135269.18	ENSP00000350937.4	19.5	1263

RYPNSHTHYF	10	0.0083	CNOT1	ENSG00000125107.18	ENSP00000320949.5	48.7	7128
EYIRSLNQF	9	0.0080	RMC1	ENSG00000141452.9	ENSP00000467007.1	24.2	1827
SYTRLFSNF	9	0.0087	UBP1	ENSG00000153560.12	ENSP00000395558.2	22.7	1512
AQFQVHHI	8	6.4570	LAMC3	ENSG00000050555.18	ENSP00000354360.4	15.5	4725
TYAELMQTL	9	0.0090	MRPS33	ENSG00000090263.16	ENSP00000420709.1	31.4	288
RQHEIVLKV	9	2.7243	NUP133	ENSG00000069248.12	ENSP00000261396.3	14.4	3468
VFTSVRQKI	9	0.3510	SMARCA4	ENSG00000127616.18	ENSP00000496004.1	46.7	4365
SYSHIMALI	9	0.0869	FDPS	ENSG00000160752.14	ENSP00000391755.1	99.4	1059
DIMDSSLTRR	10	30.6667	FDPS	ENSG00000160752.14	ENSP00000391755.1	99.4	1059
PYTKVEESF	9	0.0412	ALG12	ENSG00000182858.14	ENSP00000333813.5	15.7	1464
VYTSVVEEL	9	0.0209	MAN2B2	ENSG00000013288.8	ENSP00000285599.3	26.3	3027
YYPNPPLVL	9	0.0318	NT5DC1	ENSG00000178425.14	ENSP00000326858.3	18.1	1365
EYLENALKL	9	0.1132	GBP4	ENSG00000162654.9	ENSP00000359490.5	14.2	1920
AYINRASLL	9	0.1419	COPS4	ENSG00000138663.9	ENSP00000424655.1	27.8	1260
KYHIVKQIF	9	0.0238	HERC3	ENSG00000138641.18	ENSP00000385684.1	16.3	3150
NYITAALKL	9	0.1203	HERC3	ENSG00000138641.18	ENSP00000385684.1	16.3	3150
MYLKTVDKF	9	0.0039	TRAPPC2B	ENSG00000256060.2	ENSP00000442778.1	14.2	420
YYQDTPKQI	9	0.0313	NOP16	ENSG00000048162.20	ENSP00000480832.1	20.7	534
EVLETRVMER	10	32.4000	CSTF2T	ENSG00000177613.8	ENSP00000332444.4	13.6	1848
RYFPDRNVALF	11	0.0098	INPP5E	ENSG00000148384.13	ENSP00000360777.3	14.2	1932
RYPLLLQKI	9	0.0121	ARHGEF37	ENSG00000183111.12	ENSP00000328083.6	14.5	2025
RYIHPQQEAF	10	0.0198	CPSF1	ENSG00000071894.17	ENSP00000484669.1	49.7	4329
RYAALRELI	9	0.1220	CCDC97	ENSG00000142039.4	ENSP00000269967.2	17.2	1029
NYIKSLSSF	9	0.0075	SMG1	ENSG00000157106.16	ENSP00000402515.2	19.4	10983
RYMPQNPHI	9	0.0093	RBBP7	ENSG00000102054.17	ENSP00000369427.2	52.8	1275
RYMPQNPHII	10	0.0583	RBBP7	ENSG00000102054.17	ENSP00000369427.2	52.8	1275
AYLEAYKEF	9	0.0072	ETNK1	ENSG00000139163.15	ENSP00000266517.3	15.3	1356

DLPSFGRVR	9	26.7000	TRAPPC11	ENSG00000168538.16	ENSP00000335371.6	14.5	3399
TYLKAVKLF	9	0.0042	IREB2	ENSG00000136381.13	ENSP00000258886.8	15.5	2889
KYITDWQNVF	10	0.0204	PDCD6	ENSG00000249915.8	ENSP00000423815.1	63.5	567
NYIRITSEI	9	0.0812	DHX32	ENSG00000089876.11	ENSP00000284690.3	19.9	2229
SYGSVFKAI	9	0.1405	STK3	ENSG00000104375.17	ENSP00000390500.2	14.7	1473
EVMQAVARL	9	7.4740	TCAF1	ENSG00000198420.10	ENSP00000419235.1	15.8	2763
AYLPEWKENI	10	0.3860	TCAF1	ENSG00000198420.10	ENSP00000419235.1	15.8	2763
LYVVDLKKF	9	0.0867	UGGT1	ENSG00000136731.12	ENSP00000259253.6	14.4	4665
SYTSVKENF	9	0.0066	FAM120B	ENSG00000112584.13	ENSP00000417970.1	14.1	2730
VQYDVRGI	8	16.3455	MYO10	ENSG00000145555.15	ENSP00000274203.10	21.9	6207
VYNNNSSRF	9	0.0213	MYO10	ENSG00000145555.15	ENSP00000274203.10	21.9	6207
SYGKVTAEF	9	0.0034	ALPK2	ENSG00000198796.7	ENSP00000354991.3	18.5	6510
IYIDRGVVF	9	0.0083	TFIP11	ENSG00000100109.17	ENSP00000384297.1	25.1	2511
YYAEVETRI	9	0.0558	EXOC2	ENSG00000112685.14	ENSP00000230449.4	13.9	2772
HYQQHPLHL	9	0.0370	KIF1B	ENSG00000054523.17	ENSP00000366290.1	19.0	5448
VYLKEANAI	9	0.0941	KIF1B	ENSG00000054523.17	ENSP00000366290.1	19.0	5448
VYNDIGKEMLL	11	0.2005	KIF1B	ENSG00000054523.17	ENSP00000366290.1	19.0	5448
KYGLGFQII	9	0.1269	PTPN13	ENSG00000163629.13	ENSP00000408368.2	29.8	7398
AVIGYLSTR	9	9.8029	NOP9	ENSG00000196943.14	ENSP00000267425.3	13.7	1908
RWFTHASPTL	10	0.5035	RRM1	ENSG00000167325.15	ENSP00000300738.5	17.4	2376
SQYNIRGV	8	26.4000	UBA3	ENSG00000144744.17	ENSP00000354340.4	29.9	1389
GYASRFIVI	9	0.2298	RPIA	ENSG00000153574.9	ENSP00000283646.3	13.1	933
VYIHPSSALF	10	0.0059	DHX8	ENSG00000067596.11	ENSP00000262415.2	19.9	3660
VYLPLTSHI	9	0.0047	SNX8	ENSG00000106266.11	ENSP00000222990.3	14.3	1395
RYMQWRETM	9	0.0438	ITPKC	ENSG00000086544.3	ENSP00000263370.1	13.1	2049
SYQSQINHI	9	0.0181	DDX50	ENSG00000107625.13	ENSP00000362687.3	27.6	2211
RYMDLAENARF	11	0.0206	SPECC1L	ENSG00000100014.20	ENSP00000499052.1	15.9	3408

KYAKLEEKL	9	0.0889	GOLGB1	ENSG00000173230.15	ENSP00000377275.3	23.6	9807
RYQEKISAL	9	0.0577	GOLGB1	ENSG00000173230.15	ENSP00000377275.3	23.6	9807
FYNLLTRTF	9	0.0171	PIGT	ENSG00000124155.18	ENSP00000443963.3	57.5	1152
AYTDCIPQL	9	0.1422	KIFAP3	ENSG00000075945.13	ENSP00000444622.1	22.7	2142
NYNEKIYEL	9	0.0292	UFD1	ENSG00000070010.19	ENSP00000263202.9	31.1	921
HQGILSRV	8	31.1667	NUP153	ENSG00000124789.11	ENSP00000262077.2	13.6	4425
RYLNSQQQYF	10	0.0203	NFRKB	ENSG00000170322.14	ENSP00000400476.2	15.6	3897
RFYNHPLHF	9	0.0064	CNNM3	ENSG00000168763.16	ENSP00000305449.3	14.5	2121
KYQDVYVEL	9	0.0072	TRIOBP	ENSG00000100106.22	ENSP00000496394.1	28.9	7095
SQLQALHI	8	12.2778	ROCK2	ENSG00000134318.14	ENSP00000317985.6	13.2	4164
SSYVVKKV	8	25.4167	HERC2	ENSG00000128731.17	ENSP00000261609.7	17.4	14502
VYIHHFDRI	9	0.0327	MOB3A	ENSG00000172081.14	ENSP00000466249.1	26.2	651
EYDNFFQHL	9	0.1338	CLASP1	ENSG00000074054.18	ENSP00000386442.3	23.3	4413
GVISMPVAAR	10	41.5000	FP565260.6	ENSG00000280433.1	ENSP00000485615.1	13.4	609
VYTVHHVVW	9	0.0522	MAST3	ENSG00000099308.10	ENSP00000262811.4	13.6	3927
VYTGLFYKL	9	0.0073	NUDT6	ENSG00000170917.13	ENSP00000306070.5	15.1	948
KYFEKQFEL	9	0.0092	TATDN1	ENSG00000147687.19	ENSP00000430274.1	26.3	966
VYQEVQAMF	9	0.0022	FAM98B	ENSG00000171262.11	ENSP00000380734.2	12.8	1299
IYKDYQYYF	9	0.0028	MRC1	ENSG00000260314.3	ENSP00000455897.1	12.4	4368
KYTFPAEHRF	10	0.0095	GDA	ENSG00000119125.16	ENSP00000436619.1	27.2	1362
KQQKLWHLF	9	0.1720	C16orf72	ENSG00000182831.12	ENSP00000331720.7	15.5	825
KYFEVPSVL	9	0.0163	ACTR10	ENSG00000131966.14	ENSP00000254286.4	32.5	1251
IYFEYSHAF	9	0.0022	MSH3	ENSG00000113318.11	ENSP00000499502.1	23.6	3216
RYTPFSNGL	9	0.1258	WDR59	ENSG00000103091.15	ENSP00000262144.6	31.2	2922
TYADTLNHL	9	0.0244	NBAS	ENSG00000151779.13	ENSP00000281513.5	15.4	7113
RYLVISEKL	9	0.0254	LRRC42	ENSG00000116212.15	ENSP00000360421.3	18.3	1284
VYLDYIRVI	9	0.0297	NUP98	ENSG00000110713.17	ENSP00000316032.7	20.9	5400

RYLEKSGVL	9	0.2609	MYCBP	ENSG00000214114.9	ENSP00000380702.2	13.7	309
YYITGNLETF	10	0.0090	SEL1L3	ENSG00000091490.11	ENSP00000264868.5	20.2	3291
RYKDTINKI	9	0.0230	ROCK1	ENSG00000067900.8	ENSP00000382697.1	13.5	4062
RYPAIIYNI	9	0.0087	SLC35B1	ENSG00000121073.14	ENSP00000423323.1	39.1	681
VYPEEHSRW	9	0.0249	RSRP1	ENSG00000117616.18	ENSP00000243189.7	39.5	870
RYLPQTYVV	9	0.0334	ITM2A	ENSG00000078596.11	ENSP00000362395.2	25.7	789
KYAPRFNGF	9	0.0078	USP32	ENSG00000170832.13	ENSP00000300896.3	16.7	4812
TWTEVSYTF	9	0.0087	FBXO6	ENSG00000116663.11	ENSP00000365944.4	12.3	879
AYAENIALL	9	0.0387	PIK3R4	ENSG00000196455.8	ENSP00000349205.3	17.3	4074
SYKYEHLRW	9	0.1621	FLT4	ENSG00000037280.16	ENSP00000261937.6	16.5	4089
KYGNLSNFL	9	0.1237	FLT4	ENSG00000037280.16	ENSP00000261937.6	16.5	4089
QTPQILVK	8	36.3333	AKAP9	ENSG00000127914.16	ENSP00000378042.2	39.3	5307
EVITNRPKGF	10	7.9164	AKAP9	ENSG00000127914.16	ENSP00000378042.2	39.3	5307
VYVKHSISF	9	0.0038	AP3M1	ENSG00000185009.12	ENSP00000347408.4	15.0	1254
KYLHPPTHL	9	0.0187	MBNL2	ENSG00000139793.18	ENSP00000432422.1	24.1	765
IFTNTVARF	9	0.0604	AKAP11	ENSG00000023516.9	ENSP00000025301.2	11.9	5703
VYFGDPVSL	9	0.0276	GNPAT	ENSG00000116906.13	ENSP00000355607.4	24.1	2040
HYLNHWNHF	9	0.0057	FN3KRP	ENSG00000141560.15	ENSP00000269373.6	20.3	927
AYGGAFYAF	9	0.0102	L3HYPDH	ENSG00000126790.12	ENSP00000247194.4	23.4	1062
DLIGQDLNSR	10	42.5000	FBRS	ENSG00000156860.15	ENSP00000348489.5	19.9	2940
RYVFQSENTF	10	0.0357	SUFU	ENSG00000107882.11	ENSP00000358918.3	12.4	1452
RQPSLFYHL	9	0.2162	MAP3K5	ENSG00000197442.10	ENSP00000351908.4	12.0	4122
SYLPISPTF	9	0.0007	SLC23A2	ENSG00000089057.15	ENSP00000344322.1	12.3	1950
QYVTQINRL	9	0.0954	PREX1	ENSG00000124126.14	ENSP00000361009.3	15.4	4977
EAYHRVFVNR	10	15.9130	NT5C2	ENSG00000076685.18	ENSP00000383960.3	41.4	1683
HYIDAGYLGF	10	0.0205	GTF3C2	ENSG00000115207.13	ENSP00000264720.3	23.3	2733
PYCSPPMTF	9	0.0224	HCFC1R1	ENSG00000103145.10	ENSP00000460639.1	42.6	414

ELPIVTPALR	10	26.0000	KPNA2	ENSG00000182481.9	ENSP00000332455.3	12.7	1587
EQYRQVISV	9	8.4538	VPS11	ENSG00000160695.14	ENSP00000481126.1	40.7	2823
TQRIFQEAV	9	18.5789	NRARP	ENSG00000198435.4	ENSP00000349041.2	11.7	342
AYSAKIALF	9	0.0062	DPYD	ENSG00000188641.13	ENSP00000359211.3	12.7	3075
AQRAFILTV	9	6.4542	CABIN1	ENSG00000099991.18	ENSP00000381364.2	31.8	6660
WYMDNPQNL	9	0.0562	PLEKHA2	ENSG00000169499.15	ENSP00000482228.1	17.0	1275
IYHPNVDKL	9	0.0167	UBE2N	ENSG00000177889.10	ENSP00000316176.2	13.8	456
QYLMDNPTF	9	0.0240	PRPF40A	ENSG00000196504.16	ENSP00000386458.2	21.2	2982
NYAWVYYHM	9	0.1280	IFIT2	ENSG00000119922.10	ENSP00000490935.1	12.4	1416
PYFSGSSTF	9	0.0164	KIF21A	ENSG00000139116.18	ENSP00000445606.2	26.7	4911
PYLGQAPFL	9	0.1656	SYNRG	ENSG00000275066.5	ENSP00000483453.1	16.0	3942
GYPEVALHF	9	0.0050	COPA	ENSG00000122218.16	ENSP00000357048.3	75.3	3699
SYNPAENAVLL	11	0.2088	COPA	ENSG00000122218.16	ENSP00000357048.3	75.3	3699
NYPDEFTKL	9	0.0704	NF1	ENSG00000196712.17	ENSP00000351015.4	16.0	8517
HYTIVFNTF	9	0.0053	ATP2B4	ENSG00000058668.14	ENSP00000340930.2	14.1	3510
IFIDPGYQTF	10	0.0117	ZMYM2	ENSG00000121741.16	ENSP00000372324.2	18.7	4131
AYAPPPHVI	9	0.0079	APOL2	ENSG00000128335.14	ENSP00000351292.5	15.9	1011
YYIFIPSKF	9	0.0026	DDX47	ENSG00000213782.7	ENSP00000350698.3	30.3	1365
WVIGSVVAR	9	32.6000	GART	ENSG00000159131.17	ENSP00000371236.4	20.6	3030
RYNEKCFKL	9	0.0692	KLHL24	ENSG00000114796.16	ENSP00000395012.1	16.3	1800
YWMHVQNTF	9	0.0130	KLHL24	ENSG00000114796.16	ENSP00000395012.1	16.3	1800
QSTRLPLI	8	18.3947	PRKDC	ENSG00000253729.7	ENSP00000345182.4	16.3	12291
PYRLIFEKF	9	0.1154	PRKDC	ENSG00000253729.7	ENSP00000345182.4	16.3	12291
LYQRAFQHL	9	0.0160	PRKDC	ENSG00000253729.7	ENSP00000345182.4	16.3	12291
TYKDYVDLF	9	0.0058	PRKDC	ENSG00000253729.7	ENSP00000345182.4	16.3	12291
VYTPVLEHL	9	0.0038	PRKDC	ENSG00000253729.7	ENSP00000345182.4	16.3	12291
RYQRLYVKF	9	0.0045	PRR12	ENSG00000126464.14	ENSP00000394510.1	12.5	6108

AYTPFHAVL	9	0.0646	INTS14	ENSG00000138614.15	ENSP00000326379.2	19.1	1554
IYAGGIKSI	9	0.0969	SLC12A4	ENSG00000124067.17	ENSP00000395983.2	27.3	3261
SYLTSASSL	9	0.1760	UBR5	ENSG00000104517.13	ENSP00000429084.1	24.6	8397
VYSQIPAAVKL	11	0.1193	UBR5	ENSG00000104517.13	ENSP00000429084.1	24.6	8397
TYLTNHLRL	9	0.0482	ZNF697	ENSG00000143067.5	ENSP00000396857.2	11.3	1635
RYLSKATTL	9	0.0202	CDC16	ENSG00000130177.16	ENSP00000252458.6	58.0	1425
PYHPHPHVF	9	0.0090	FOXN3	ENSG00000053254.15	ENSP00000343288.4	21.1	1470
STVGGWVSTR	10	27.4000	AGPS	ENSG00000018510.15	ENSP00000264167.4	11.8	1974
TYKDTHRKF	9	0.0762	IFT88	ENSG00000032742.17	ENSP00000261632.5	24.3	2472
RYTPEQDTMTF	11	0.0105	RARA	ENSG00000131759.18	ENSP00000377648.3	24.4	1434
LYQDGVFKF	9	0.0022	AKTIP	ENSG00000166971.17	ENSP00000454771.1	29.5	381
RYAVEILTW	9	0.0128	UBR2	ENSG00000024048.10	ENSP00000361992.1	14.5	5265
RYGDGGSTF	9	0.0128	HNRNPH1	ENSG00000169045.17	ENSP00000327539.6	139.4	1416
YQFNVKTV	8	9.1772	PTPRB	ENSG00000127329.15	ENSP00000438927.1	31.0	5721
KYMATVTSI	9	0.0393	PTPRB	ENSG00000127329.15	ENSP00000438927.1	31.0	5721
VYMNRVKEI	9	0.0801	C1D	ENSG00000197223.11	ENSP00000386468.3	23.6	423
VYHILKVLF	9	0.0118	ELP1	ENSG00000070061.15	ENSP00000363779.5	17.7	3996
SQALILKI	8	9.8147	NUP188	ENSG00000095319.14	ENSP00000361658.2	13.6	5247
NYSNIRFQF	9	0.0096	MTMR6	ENSG00000139505.11	ENSP00000371221.5	11.1	1863
RYSEYAEEF	9	0.0054	MTMR6	ENSG00000139505.11	ENSP00000371221.5	11.1	1863
AYSRVQYQF	9	0.0024	BCAR3	ENSG00000137936.18	ENSP00000260502.6	30.4	2475
KYAQWEESL	9	0.1067	CRNKL1	ENSG00000101343.14	ENSP00000440733.1	12.2	2061
RFEEKHAYF	9	0.1403	CRNKL1	ENSG00000101343.14	ENSP00000440733.1	12.2	2061
HYIERIQKL	9	0.0099	KANK1	ENSG00000107104.18	ENSP00000371740.1	56.2	4056
SYPDNFLHI	9	0.0090	ATG5	ENSG00000057663.16	ENSP00000343313.3	17.4	825
LYDIVFKHF	9	0.0202	SP100	ENSG00000067066.17	ENSP00000386427.1	48.1	2064
VYVDLGGSHVF	11	0.0196	BMS1	ENSG00000165733.8	ENSP00000363642.4	10.9	3846

VYPSSLSKI	9	0.0257	CAND1	ENSG00000111530.13	ENSP00000442318.1	14.1	3690
YATHPFKF	8	0.3811	NCOA7	ENSG00000111912.20	ENSP00000357341.3	116.8	2826
AYATHPFKF	9	0.0033	NCOA7	ENSG00000111912.20	ENSP00000357341.3	116.8	2826
ETASAVATR	9	21.3636	NCOA7	ENSG00000111912.20	ENSP00000357341.3	116.8	2826
TYIDTRTVF	9	0.0198	CAPN6	ENSG00000077274.9	ENSP00000317214.1	10.8	1923
GYIESVQHI	9	0.0253	MGME1	ENSG00000125871.14	ENSP00000366939.5	12.0	1032
VYYPVRHHL	9	0.0050	TRRAP	ENSG00000196367.13	ENSP00000403708.3	16.4	11544
RYFENPQVI	9	0.0138	TRRAP	ENSG00000196367.13	ENSP00000403708.3	16.4	11544
EVVSLLLDR	9	43.0000	ANKHD1-EIF4EBP3	ENSG00000254996.5	ENSP00000432016.1	28.6	7851
EVYDGPKNVR	10	11.4293	PCM1	ENSG00000078674.17	ENSP00000429054.1	34.2	2292
EQIDKKLER	9	15.9275	PTPN12	ENSG00000127947.16	ENSP00000248594.6	26.8	2340
IYNKIKQII	9	0.0481	DLG3	ENSG00000082458.12	ENSP00000441393.1	22.3	1098
SYGDILHVI	9	0.0092	DLG3	ENSG00000082458.12	ENSP00000441393.1	22.3	1098
RFLNDPGHLLW	11	0.1454	RPP21	ENSG00000241370.5	ENSP00000394320.2	26.1	531
RYLELISSI	9	0.0205	IFT122	ENSG00000163913.11	ENSP00000324005.3	25.8	3723
TMLGKFYHF	9	0.0873	IFT122	ENSG00000163913.11	ENSP00000324005.3	25.8	3723
SYNPITHQL	9	0.0025	IFT122	ENSG00000163913.11	ENSP00000324005.3	25.8	3723
RYIEFHSQSGF	11	0.0514	NOL10	ENSG00000115761.16	ENSP00000371101.5	12.1	2064
RYLEAGAAGLRW	12	0.2881	HSPBP1	ENSG00000133265.11	ENSP00000398244.1	28.2	1077
RYFKGPELL	9	0.0100	CSNK2A2	ENSG00000070770.9	ENSP00000262506.3	39.3	1050
EVVDVSTMHR	10	25.8333	HERC1	ENSG00000103657.14	ENSP00000390158.2	17.6	14583
EYNSALPLL	9	0.1042	GTF3C3	ENSG00000119041.11	ENSP00000263956.3	14.2	2658
YYLQGGFNKF	10	0.0064	DUSP7	ENSG00000164086.10	ENSP00000417183.1	10.9	1257
NYQNVVHKL	9	0.0081	ZMYM4	ENSG00000146463.11	ENSP00000322915.6	12.5	4644
EIYSGCTKR	9	28.1250	DNAJB4	ENSG00000162616.9	ENSP00000359799.5	23.0	1011
TYGIKHMKF	9	0.0173	PIGK	ENSG00000142892.15	ENSP00000388854.1	17.6	903
GYCPNHPVI	9	0.3044	NEDD4L	ENSG00000049759.18	ENSP00000411947.1	78.6	2562

SYINELRTF	9	0.0040	SPG11	ENSG00000104133.15	ENSP00000445278.2	28.3	6990
SYASLQQNKW	10	0.1314	SPG11	ENSG00000104133.15	ENSP00000445278.2	28.3	6990
VYNNSRMLHF	10	0.0568	ATXN2L	ENSG00000168488.18	ENSP00000457613.1	32.9	3204
TYDYNFHSF	9	0.0054	ASH1L	ENSG00000116539.13	ENSP00000357330.3	14.0	8907
EVIGMVTRA	9	43.0000	NR1D2	ENSG00000174738.13	ENSP00000310006.3	11.2	1737
ETAFGYKGL	9	15.4783	HAT1	ENSG00000128708.13	ENSP00000264108.4	27.4	1257
EYGVIRDVL	9	0.8919	H6PD	ENSG00000049239.12	ENSP00000366620.1	10.9	2373
VYVKHNISF	9	0.0059	AP3M2	ENSG00000070718.12	ENSP00000428787.1	12.7	1254
AQRLMQLI	8	19.2581	MED12	ENSG00000184634.16	ENSP00000363193.3	14.7	6531
GYSSRFGLF	9	0.1254	GBA3	ENSG00000249948.6	ENSP00000480447.1	161.5	483
IYVHDLLTF	9	0.0061	VPS39	ENSG00000166887.15	ENSP00000326534.5	48.8	2625
IYLDTHFRL	9	0.0074	ZNFX1	ENSG00000124201.15	ENSP00000379412.1	15.2	5754
HQKSILTQI	9	2.4012	KIDINS220	ENSG00000134313.15	ENSP00000418974.1	23.5	5256
KYGTTPLVW	9	0.0472	KIDINS220	ENSG00000134313.15	ENSP00000418974.1	23.5	5256
SYSSIASEF	9	0.0066	TMX3	ENSG00000166479.10	ENSP00000299608.2	16.6	1362
VYDPLFSQL	9	0.0201	SRRD	ENSG00000100104.13	ENSP00000215917.6	10.7	1017
RYIADHSYF	9	0.0082	FAM160A2	ENSG00000051009.10	ENSP00000431773.1	57.2	2514
NYIMKIHNF	9	0.0043	ADNP	ENSG00000101126.17	ENSP00000495540.1	12.5	2622
NYNRAFQVW	9	0.0636	SLC44A3	ENSG00000143036.17	ENSP00000431836.1	29.6	1860
TVISRVIGR	9	15.8406	ANKRD17	ENSG00000132466.18	ENSP00000427151.2	26.4	7470
KYQRILERL	9	0.0301	OPA1	ENSG00000198836.10	ENSP00000494273.1	21.4	1707
VYCARFVEL	9	0.1363	THOC2	ENSG00000125676.20	ENSP00000245838.8	19.0	4779
EVISKTPTL	9	2.3082	CREBRF	ENSG00000164463.12	ENSP00000296953.2	11.0	1917
EYIKFLRSI	9	0.1105	HDAC2	ENSG00000196591.12	ENSP00000357621.2	25.4	1374
KYADKIYSI	9	0.0033	FOLH1	ENSG00000086205.18	ENSP00000431463.1	23.2	2112
YYLTDIDRI	9	0.1415	GNA14	ENSG00000156049.7	ENSP00000365807.4	10.1	1065
TYAQRTQLF	9	0.0047	MKLN1	ENSG00000128585.18	ENSP00000323527.6	16.5	2205

LQPERYELW	9	0.2782	KDM4B	ENSG00000127663.15	ENSP00000440495.1	14.9	3390
VYNTATNQWF	10	0.0599	HCFC1	ENSG00000172534.14	ENSP00000359001.4	15.6	6240
SFIKVSHV	8	5.0112	INPP5K	ENSG00000132376.20	ENSP00000318476.6	25.3	1116
PYQSQIAVF	9	0.0263	RAD17	ENSG00000152942.19	ENSP00000482775.1	18.3	2010
HAYIIAKV	8	32.6000	PTPRC	ENSG00000081237.20	ENSP00000306782.7	14.1	3435
TYTLRVFEL	9	0.0961	PTPRC	ENSG00000081237.20	ENSP00000306782.7	14.1	3435
AYVGDLQTL	9	0.0679	ASB1	ENSG00000065802.12	ENSP00000264607.4	11.0	1005
DYMKTTSNF	9	0.0203	NEMF	ENSG00000165525.18	ENSP00000298310.5	15.2	3228
QQFKILNV	8	15.6812	UPF2	ENSG00000151461.20	ENSP00000380244.2	13.3	3816
RYVWWKKSL	9	0.2932	UPF2	ENSG00000151461.20	ENSP00000380244.2	13.3	3816
DLIFKTGGR	9	33.7500	UPF2	ENSG00000151461.20	ENSP00000380244.2	13.3	3816
ILGPPPPSF	9	0.1364	MATR3	ENSG00000015479.18	ENSP00000482895.1	42.0	2541
GYIERPQLI	9	0.0382	TENT2	ENSG00000164329.13	ENSP00000421966.1	18.2	1323
EVVGIIESR	9	24.0000	NUP160	ENSG00000030066.13	ENSP00000367721.2	17.6	4308
EVADRLFSSF	10	4.4254	TAPT1	ENSG00000169762.17	ENSP00000385347.2	15.5	1701
RYCFQITSF	9	0.0336	APPL1	ENSG00000157500.12	ENSP00000288266.3	23.5	2127
DIISQQLVER	10	49.0000	MPV17	ENSG00000115204.15	ENSP00000385175.1	62.6	339
EIISKLQGR	9	20.0000	PHKA2	ENSG00000044446.11	ENSP00000369274.4	30.0	3705
RYKFHNSRW	9	0.2913	TBX3	ENSG00000135111.16	ENSP00000257567.2	11.2	2169
RYFPVFEKI	9	0.0037	GSTA4	ENSG00000170899.11	ENSP00000360002.4	24.9	666
SYLNTVERW	9	0.0099	KLHL5	ENSG00000109790.16	ENSP00000425512.1	24.0	846
ALPSKLPTF	9	0.0710	TRIM25	ENSG00000121060.18	ENSP00000323889.4	18.3	1890
EYTRALFLL	9	0.1123	MAU2	ENSG00000129933.21	ENSP00000262815.9	19.2	1839
KYYDKKYQVF	10	0.0196	FGGY	ENSG00000172456.17	ENSP00000360262.4	38.6	1725
VYVERAEVL	9	0.0898	BRAP	ENSG00000089234.16	ENSP00000403524.3	9.8	1776
KYQEYTNEL	9	0.0277	TBK1	ENSG00000183735.10	ENSP00000498885.1	12.9	2031
EYTDVLQKI	9	0.0705	EPS8L1	ENSG00000131037.15	ENSP00000437541.1	33.7	1977

VYIPMSPGAHHF	12	0.0076	GAB2	ENSG00000033327.13	ENSP00000354952.4	9.7	2028
RYPQSIFSTF	10	0.0036	SBF2	ENSG00000133812.15	ENSP00000256190.8	21.7	5547
QYTPHSHQF	9	0.0027	RPTOR	ENSG00000141564.15	ENSP00000307272.3	11.7	4005
KYGSVIQLL	9	0.0052	ITPR3	ENSG00000096433.11	ENSP00000475177.1	9.5	8013
AYLEALSHL	9	0.0271	PIK3CD	ENSG00000171608.15	ENSP00000366563.4	11.5	3132
YYQSSVQYL	9	0.0083	DNAAF5	ENSG00000164818.16	ENSP00000297440.6	22.3	2565
KYHTHLLQF	9	0.0065	ABCD1	ENSG00000101986.12	ENSP00000218104.3	9.6	2235
EYVRVSEHF	9	0.0132	ZC3HAV1	ENSG00000105939.13	ENSP00000242351.5	11.6	2706
NSALVLKV	8	27.7000	RB1	ENSG00000139687.15	ENSP00000267163.4	13.3	2784
QYVVDLTSF	9	0.0385	NTPCR	ENSG00000135778.12	ENSP00000355587.4	21.0	570
EVIDFSHGL	9	5.1162	ABHD17C	ENSG00000136379.12	ENSP00000258884.4	11.9	987
KYSDVSGLL	9	0.1292	INO80C	ENSG00000153391.15	ENSP00000391457.1	24.4	684
RYFDHALTL	9	0.0072	FAM91A1	ENSG00000176853.16	ENSP00000429491.1	17.8	2367
TYHGVFVNI	9	0.0410	VPS26C	ENSG00000157538.14	ENSP00000311399.6	22.9	891
AQYKFIYV	8	13.0631	PTPN6	ENSG00000111679.17	ENSP00000326010.9	44.5	1785
RYTVGGLETF	10	0.0291	PTPN6	ENSG00000111679.17	ENSP00000326010.9	44.5	1785
KYMEDYHQF	9	0.0005	PPL	ENSG00000118898.16	ENSP00000340510.2	12.3	5268
NYKDLNGNVF	10	0.2590	SSR1	ENSG00000124783.14	ENSP00000244763.4	11.7	858
IYPMKNHMF	9	0.0089	RALGAPA2	ENSG00000188559.15	ENSP00000202677.6	13.6	5619
EVIFHVSTR	9	23.2500	RALGAPA2	ENSG00000188559.15	ENSP00000202677.6	13.6	5619
KYADAVKNF	9	0.0051	RASA3	ENSG00000185989.11	ENSP00000335029.7	9.1	2502
AYGFVARI	8	1.8018	VWF	ENSG00000110799.13	ENSP00000261405.5	9.6	8439
EVIASYAHL	9	5.6274	VWF	ENSG00000110799.13	ENSP00000261405.5	9.6	8439
HQEVLREI	8	18.5000	TAP2	ENSG00000204267.15	ENSP00000498827.1	18.8	1959
TYGERFCYL	9	0.1785	DPY19L4	ENSG00000156162.16	ENSP00000389630.2	10.5	2169
NYSPYVNYF	9	0.0028	DPY19L4	ENSG00000156162.16	ENSP00000389630.2	10.5	2169
VYQHLFTRI	9	0.0176	AP5Z1	ENSG00000242802.9	ENSP00000497395.1	16.6	1464

VYAKLLHRY	9	0.1186	FBXO31	ENSG00000103264.18	ENSP00000310841.4	12.2	1617
LYSRVQATF	9	0.0045	FBXO31	ENSG00000103264.18	ENSP00000310841.4	12.2	1617
ETMGRALRY	9	10.0038	ELF1	ENSG00000120690.16	ENSP00000239882.3	21.7	1857
SYLEKQVVTF	10	0.0074	TTC27	ENSG00000018699.13	ENSP00000313953.4	9.2	2529
EYIAKQFGF	9	0.0500	ITPR1	ENSG00000150995.19	ENSP00000349597.4	64.0	8130
VYPVITARL	9	0.0206	ZNF106	ENSG00000103994.17	ENSP00000263805.4	15.8	5649
AYGSLFNTI	9	0.0435	KYAT3	ENSG00000137944.18	ENSP00000359522.3	21.6	1260
RYIFAKNLF	9	0.0116	DGUOK	ENSG00000114956.20	ENSP00000264093.4	32.6	831
VYPGDPLRF	9	0.0068	TSEN34	ENSG00000170892.12	ENSP00000379667.1	30.4	930
SYLEKVVTL	9	0.0043	ABI3	ENSG00000108798.9	ENSP00000225941.1	11.5	1098
TQVLIKLI	8	19.6452	COPS2	ENSG00000166200.15	ENSP00000373553.5	16.0	1329
KYLVLANML	9	0.1245	COPS2	ENSG00000166200.15	ENSP00000373553.5	16.0	1329
GQFLVRII	8	22.9474	GUCY1B1	ENSG00000061918.13	ENSP00000420842.1	16.4	1758
EVVTGVIGQR	10	45.5000	GUCY1B1	ENSG00000061918.13	ENSP00000420842.1	16.4	1758
VYSIVPQSW	9	0.0078	LARP4	ENSG00000161813.22	ENSP00000293618.8	19.9	1959
TYNPAFSRL	9	0.0144	TMEM181	ENSG00000146433.8	ENSP00000356057.3	8.8	1836
EAIGIISKM	9	7.1005	NCAPD2	ENSG00000010292.13	ENSP00000325017.5	15.6	4203
VYTGIDHHW	9	0.0124	DCAF13	ENSG00000164934.14	ENSP00000484962.1	17.8	1335
LYLLNTTKL	9	0.0714	MLH1	ENSG00000076242.14	ENSP00000398272.2	19.2	1545
IWISKLPHF	9	0.0086	TDRD7	ENSG00000196116.8	ENSP00000347444.4	9.1	3294
LFIATSQKF	9	0.0541	NCAPH2	ENSG00000025770.19	ENSP00000410088.2	24.3	1815
EYVEKFYRI	9	0.0259	RAD50	ENSG00000113522.14	ENSP00000368100.4	12.5	3936
RYIKALETL	9	0.0088	RAD50	ENSG00000113522.14	ENSP00000368100.4	12.5	3936
VYSQFITQL	9	0.0094	RAD50	ENSG00000113522.14	ENSP00000368100.4	12.5	3936
TYTDKLFKF	9	0.0025	CRTAC1	ENSG00000095713.14	ENSP00000359629.3	9.8	1983
RYQEVDRRF	9	0.0112	YTHDC1	ENSG00000083896.12	ENSP00000347888.3	51.3	2127
IYAPPLPSL	9	0.0054	AKAP1	ENSG00000121057.13	ENSP00000478212.1	25.0	2709

TYRNVMEQF	9	0.0218	BAIAP2L1	ENSG00000006453.14	ENSP00000005260.8	8.8	1533
IYLLIHNNF	9	0.0094	CD53	ENSG00000143119.14	ENSP00000497382.1	13.5	657
IYTNLNQKI	9	0.0304	EXOC6B	ENSG00000144036.15	ENSP00000272427.6	9.5	2433
VYNQIKEFI	9	0.0505	EXOC6B	ENSG00000144036.15	ENSP00000272427.6	9.5	2433
SVYDGEEHGR	10	12.8750	EXOC6B	ENSG00000144036.15	ENSP00000272427.6	9.5	2433
RFVMKAHSF	9	0.1245	SOAT1	ENSG00000057252.13	ENSP00000356591.3	9.1	1650
TYTSNLVRL	9	0.0535	DNAJC16	ENSG00000116138.13	ENSP00000480224.1	10.7	1410
EVWPHSTER	9	13.3694	LYSMD1	ENSG00000163155.12	ENSP00000357904.5	8.8	681
KYGRDFQAI	9	0.1605	RCOR1	ENSG00000089902.10	ENSP00000262241.5	9.4	1455
VYPPIRHHL	9	0.0075	VPS37A	ENSG00000155975.10	ENSP00000318629.4	11.2	1191
KYKSAFNKL	9	0.0281	DENND4B	ENSG00000198837.10	ENSP00000354597.4	24.9	4488
TYVRWYTQL	9	0.0521	CLCC1	ENSG00000121940.15	ENSP00000358987.3	11.5	1503
VFIGTGHLL	9	0.1314	LRRK2	ENSG00000188906.16	ENSP00000341930.2	23.4	3813
NYIGLINRI	9	0.0555	EIF2AK2	ENSG00000055332.18	ENSP00000233057.4	10.1	1653
RFKNKAYLL	9	0.2653	DICER1	ENSG00000100697.14	ENSP00000376783.1	10.8	5766
VYQNHVQHL	9	0.0069	ARHGAP5	ENSG00000100852.13	ENSP00000371897.1	25.4	4506
SYTHIQYLF	9	0.0022	SH2D3C	ENSG00000095370.20	ENSP00000485866.1	11.1	2100
VYTTTRSHL	9	0.2157	CLPTM1L	ENSG00000049656.14	ENSP00000313854.5	133.4	1614
LYGRHFNYL	9	0.1117	TENT4B	ENSG00000121274.12	ENSP00000455837.1	9.2	1764
RYPVGRFPSL	10	0.1852	FEM1A	ENSG00000141965.4	ENSP00000269856.3	8.2	2007
YYLNDLDRI	9	0.1787	GNAI1	ENSG00000127955.17	ENSP00000498091.1	45.7	1062
ASPTVCFLKER	11	31.3333	GC	ENSG00000145321.13	ENSP00000273951.8	32.7	1422
KYVSGEFRF	9	0.0074	RIOK1	ENSG00000124784.9	ENSP00000369162.2	8.6	1704
AYLSKAMEI	9	0.0860	RIOK1	ENSG00000124784.9	ENSP00000369162.2	8.6	1704
RYSPVLSRF	9	0.0012	COG1	ENSG00000166685.12	ENSP00000299886.4	26.2	2940
RYNPRTNQW	9	0.0172	KLHL20	ENSG00000076321.11	ENSP00000209884.4	9.9	1827
RYDPKTNQW	9	0.0114	KLHL20	ENSG00000076321.11	ENSP00000209884.4	9.9	1827

RYGLVTNEI	9	0.1078	IFIH1	ENSG00000115267.8	ENSP00000497271.1	11.5	3075
VYNNIMRHYL	10	0.2372	IFIH1	ENSG00000115267.8	ENSP00000497271.1	11.5	3075
EVAEHVQYM	9	5.5985	GMNN	ENSG00000112312.10	ENSP00000230056.3	11.6	627
IYAHITKHY	9	0.2827	FOXK1	ENSG00000164916.11	ENSP00000328720.4	9.2	2199
RYGPIVDVY	9	0.1877	SRSF10	ENSG00000188529.14	ENSP00000344149.4	34.3	783
EIFPGGIRGR	10	18.0526	SLC2A12	ENSG00000146411.5	ENSP00000275230.5	8.0	1851
RYFDRAALF	9	0.0038	WDR11	ENSG00000120008.16	ENSP00000263461.5	48.1	3672
SYPSAFSKL	9	0.0211	MARF1	ENSG00000166783.22	ENSP00000450309.1	37.8	5226
TYLEKFQNL	9	0.0057	CLUAP1	ENSG00000103351.13	ENSP00000460889.1	26.0	741
EVIDVVRTM	9	3.9045	TAF1	ENSG00000147133.15	ENSP00000276072.3	14.3	5679
SYQRAFNEF	9	0.0054	TNFAIP2	ENSG00000185215.9	ENSP00000452634.1	38.7	1962
DYDSVFHHL	9	0.0811	EPC1	ENSG00000120616.16	ENSP00000318559.6	12.8	2439
SYIDLLQRF	9	0.0013	WDR24	ENSG00000127580.17	ENSP00000293883.4	8.6	2370
KYPNMFKKI	9	0.0449	AHCYL2	ENSG00000158467.16	ENSP00000413639.2	19.5	1830
KYPENDLFRKL	11	0.2237	KDM5A	ENSG00000073614.12	ENSP00000382688.2	9.7	5070
SFKQRSDLF	9	0.2681	ZNF394	ENSG00000160908.15	ENSP00000337363.6	11.9	1683
SFYKIRTI	8	1.6850	NEK9	ENSG00000119638.13	ENSP00000238616.5	29.8	2937
QYNPHVHQL	9	0.0061	DLG5	ENSG00000151208.17	ENSP00000361467.2	11.2	5757
VYVDKVEKM	9	0.0346	AMOT	ENSG00000126016.15	ENSP00000305557.1	14.3	2025
KYLEENVMRHF	11	0.0369	AMOT	ENSG00000126016.15	ENSP00000305557.1	14.3	2025
HWTKVSHKF	9	0.0250	PDE2A	ENSG00000186642.16	ENSP00000365633.3	21.3	2055
KYVKGLISI	9	0.0479	WDR35	ENSG00000118965.14	ENSP00000281405.4	9.5	3510
KYGALVNNF	9	0.0052	NSUN4	ENSG00000117481.10	ENSP00000471937.1	13.8	453
KYADLSHNRL	10	0.1365	RARS	ENSG00000113643.9	ENSP00000231572.3	27.5	1980
RYSLVHQRL	9	0.0179	FYCO1	ENSG00000163820.15	ENSP00000441178.1	14.8	4494
VYIHHFDSI	9	0.0278	MOB3C	ENSG00000142961.14	ENSP00000361008.2	10.2	648
DVIGNEILRR	10	48.0000	CLIP3	ENSG00000105270.15	ENSP00000353732.3	8.0	1641

KYGIVQEF	8	0.0790	SOGA1	ENSG00000149639.15	ENSP00000237536.4	8.1	4983
KYGIVQEFF	9	0.0093	SOGA1	ENSG00000149639.15	ENSP00000237536.4	8.1	4983
KYSNMEQSLF	10	0.0586	AQR	ENSG00000021776.11	ENSP00000156471.5	9.2	4455
HQIIVQNV	8	19.4194	E4F1	ENSG00000167967.16	ENSP00000301727.4	13.9	2352
TYMPHVHIL	9	0.0026	FBXO38	ENSG00000145868.16	ENSP00000342023.5	17.0	3564
QYFPKAPEF	9	0.0029	USP48	ENSG00000090686.15	ENSP00000363864.3	28.5	1563
NYVPVNHFW	9	0.0101	IFT140	ENSG00000187535.14	ENSP00000406012.2	22.5	4386
EVSPPNLRGR	10	26.7000	SLC2A13	ENSG00000151229.13	ENSP00000280871.4	8.5	1944
EYPKFLKRF	9	0.0262	COL4A3BP	ENSG00000113163.16	ENSP00000369862.5	16.1	1572
VYQVLQEHL	9	0.0341	BMP7	ENSG00000101144.13	ENSP00000379204.3	9.2	1293
ESISEFILRR	10	41.0000	TARBP1	ENSG00000059588.10	ENSP00000040877.1	12.7	4863
RYQKLIVQI	9	0.0102	CHN2	ENSG00000106069.22	ENSP00000486515.1	14.4	996
RYASINAHL	9	0.0369	CSNK1A1	ENSG00000113712.18	ENSP00000499757.1	26.8	975
KYITKSFNF	9	0.0024	PARN	ENSG00000140694.17	ENSP00000498898.1	20.9	1338
KYITKSFNFY	10	0.5365	PARN	ENSG00000140694.17	ENSP00000498898.1	20.9	1338
VYFPDQHINF	10	0.0037	PARD3B	ENSG00000116117.18	ENSP00000482649.1	11.6	3090
HYMPPPYASL	10	0.0343	RNF111	ENSG00000157450.15	ENSP00000453015.1	10.8	3009
KQPEEYHYL	9	0.2733	MYO9A	ENSG00000066933.16	ENSP00000348349.5	14.3	7644
SYIEFPAVM	9	0.0901	MYO9A	ENSG00000066933.16	ENSP00000348349.5	14.3	7644
PYVNNVPHL	9	0.0854	MARS	ENSG00000166986.15	ENSP00000446168.2	44.9	1638
DYSSILQKF	9	0.0077	FNBP1	ENSG00000187239.17	ENSP00000415602.2	20.4	1704
KYIDQKFVL	9	0.0265	PPP2R5C	ENSG00000078304.19	ENSP00000329009.5	46.3	1620
IYPEVVHMF	9	0.0005	PPP2R5C	ENSG00000078304.19	ENSP00000329009.5	46.3	1620
QYLPSPPLL	9	0.0066	KCNJ5	ENSG00000120457.12	ENSP00000433295.1	7.5	1257
AYGKDFHLI	9	0.0208	MIER1	ENSG00000198160.14	ENSP00000347514.3	9.4	1536
VMYRVIQV	8	13.3514	USF1	ENSG00000158773.14	ENSP00000435005.1	56.6	651
EVLQGGSQR	9	32.2000	USF1	ENSG00000158773.14	ENSP00000435005.1	56.6	651

TYLPTSPLL	9	0.0084	NRDC	ENSG00000078618.21	ENSP00000262679.8	57.0	3453
ETVGTGIMGR	10	47.0000	INPP5B	ENSG00000204084.13	ENSP00000362118.1	11.6	2247
KYVKIFDNF	9	0.0045	ZNF195	ENSG00000005801.18	ENSP00000435828.1	12.3	1830
SAGLILHQRI	10	26.2000	ZKSCAN8	ENSG00000198315.11	ENSP00000332750.5	7.7	1734
RYLEAALRL	9	0.0225	P3H4	ENSG00000141696.13	ENSP00000377505.1	15.2	1311
NYATRIVTL	9	0.0542	BIRC6	ENSG00000115760.14	ENSP00000393596.2	27.0	14571
TYGPSFPAF	9	0.0025	RALGAPB	ENSG00000170471.15	ENSP00000262879.6	11.0	4482
SYLPLAHMF	9	0.0009	ACSL5	ENSG00000197142.10	ENSP00000346223.4	9.6	2049
DIAQSQLKTR	10	50.0000	MED21	ENSG00000152944.9	ENSP00000282892.3	9.3	432
SYSSTFHSL	9	0.0086	NBPF19	ENSG00000271383.7	ENSP00000498781.1	10.9	11529
KYIKDDFRF	9	0.0068	RIOK3	ENSG00000101782.15	ENSP00000462548.1	25.0	1548
TYQDIFRDF	9	0.0094	ACAP2	ENSG00000114331.14	ENSP00000324287.6	8.7	2334
SQLILQKL	8	8.2707	TTC21B	ENSG00000123607.15	ENSP00000243344.7	11.2	3948
RYDSVINRL	9	0.0451	PCF11	ENSG00000165494.11	ENSP00000298281.4	29.8	4665
IYMNLASHI	9	0.0349	GATAD2B	ENSG00000143614.10	ENSP00000357644.4	11.0	1779
LYAHTIAGF	9	0.0549	CTDP1	ENSG00000060069.16	ENSP00000075430.7	16.2	2601
IYQKYIARF	9	0.0022	CASZ1	ENSG00000130940.15	ENSP00000366221.3	12.2	5277
KYNQVNSHF	9	0.0065	CASZ1	ENSG00000130940.15	ENSP00000366221.3	12.2	5277
RYKQDVERF	9	0.0141	SMC5	ENSG00000198887.9	ENSP00000354957.5	8.1	3303
ENISQVKER	9	46.0000	SMC5	ENSG00000198887.9	ENSP00000354957.5	8.1	3303
EIIDKRRER	9	23.3750	SMC5	ENSG00000198887.9	ENSP00000354957.5	8.1	3303
PYSEKMTVLF	10	0.0749	SMC5	ENSG00000198887.9	ENSP00000354957.5	8.1	3303
IYNKVTPTF	9	0.0011	SPRED1	ENSG00000166068.13	ENSP00000299084.4	8.0	1332
VFMRWKEQF	9	0.0335	GID4	ENSG00000141034.9	ENSP00000268719.4	7.2	900
LYGRLQELF	9	0.0098	KIAA1109	ENSG00000138688.15	ENSP00000264501.4	36.1	15015
EYGFISQTL	9	0.0943	TMCC3	ENSG00000057704.13	ENSP00000261226.4	8.7	1431
RYLLFARQF	9	0.0237	MCM6	ENSG00000076003.5	ENSP00000264156.2	6.9	2463

EVTDYAIARR	10	49.0000	MCM6	ENSG00000076003.5	ENSP00000264156.2	6.9	2463
SYLENVDHF	9	0.0037	CAMTA2	ENSG00000108509.21	ENSP00000370712.5	26.8	3591
EIIEQPKQR	9	28.1250	RELA	ENSG00000173039.19	ENSP00000311508.9	39.1	1644
YYVNGTYRL	9	0.0241	RNF145	ENSG00000145860.11	ENSP00000482720.1	9.4	1989
RYSDSTFTF	9	0.0014	PARP8	ENSG00000151883.18	ENSP00000422217.2	9.5	2562
VYKTIMEQF	9	0.0045	BAIAP2	ENSG00000175866.15	ENSP00000316338.6	42.4	1656
KYSLIKGNF	9	0.0796	S100A8	ENSG00000143546.10	ENSP00000357722.3	8.5	279
AFIEKHWTF	9	0.0057	KAT14	ENSG00000149474.13	ENSP00000392318.2	24.1	2346
RYLDVSILGKF	11	0.0210	METTL3	ENSG00000165819.12	ENSP00000298717.3	27.8	1740
LYSEVDVHF	9	0.0052	POGZ	ENSG00000143442.22	ENSP00000376484.1	21.5	4071
RYQFLEEAF	9	0.0506	TRIM33	ENSG00000197323.12	ENSP00000358556.2	15.9	3330
VYAQKHQQL	9	0.0232	TRIM33	ENSG00000197323.12	ENSP00000358556.2	15.9	3330
KYQIINEEF	9	0.0066	HPS3	ENSG00000163755.8	ENSP00000296051.2	24.0	3012
KFQDAHFEF	9	0.0116	DDX60	ENSG00000137628.17	ENSP00000377344.3	6.8	5136
SYITKPEKW	9	0.0102	MANEA	ENSG00000172469.16	ENSP00000351669.4	6.8	1386
QYMERLQLL	9	0.0112	MTX1	ENSG00000173171.14	ENSP00000476632.1	23.7	951
NYVEVTRKW	9	0.0602	MTX1	ENSG00000173171.14	ENSP00000476632.1	23.7	951
IYCPAPPQI	9	0.0168	CD55	ENSG00000196352.15	ENSP00000356030.2	20.0	1332
QYQEAFQKL	9	0.0083	GEMIN5	ENSG00000082516.9	ENSP00000285873.6	6.7	4524
SYARHFLDF	9	0.0206	ZADH2	ENSG00000180011.7	ENSP00000323678.3	27.8	1131
HYNSHYEKF	9	0.0073	ASTN2	ENSG00000148219.16	ENSP00000363098.3	7.7	3186
EYAKIHDLL	9	0.0843	ZNF318	ENSG00000171467.16	ENSP00000475748.1	12.6	3351
RYALIMHKL	9	0.0091	ZBTB11	ENSG00000066422.4	ENSP00000326200.4	7.2	3159
RYLEKNVKL	9	0.0248	TGS1	ENSG00000137574.10	ENSP00000260129.5	7.1	2559
VYQLRFQFL	9	0.0324	POLR1A	ENSG00000068654.16	ENSP00000263857.6	8.2	5160
IYPELQIERF	10	0.0069	APBB1IP	ENSG00000077420.16	ENSP00000365411.4	7.0	1998
SYQKLLFKF	9	0.0027	RB1CC1	ENSG00000023287.13	ENSP00000396067.2	13.7	4773

RYINNPLLI	9	0.0196	TTLL5	ENSG00000119685.20	ENSP00000298832.9	10.3	3843
IYQWVRDEL	9	0.1508	SATB1	ENSG00000182568.17	ENSP00000341024.5	9.5	2289
KYIEGVSDF	9	0.0087	IPP	ENSG00000197429.10	ENSP00000379739.3	8.6	1752
VYDPLSKRW	9	0.0269	IPP	ENSG00000197429.10	ENSP00000379739.3	8.6	1752
RYACLVTLH	9	4.9154	TENT5B	ENSG00000158246.8	ENSP00000289166.5	6.4	1275
VFIKRCFTF	9	0.0554	DOCK9	ENSG00000088387.19	ENSP00000413781.2	30.1	3762
EYKTVHEEL	9	0.2091	DOCK9	ENSG00000088387.19	ENSP00000413781.2	30.1	3762
FYHKYFNYL	9	0.0550	EXT2	ENSG00000151348.14	ENSP00000431173.1	9.7	2154
TYDRVESLF	9	0.0432	EXT2	ENSG00000151348.14	ENSP00000431173.1	9.7	2154
DYAYLREHF	9	0.1404	ZNF598	ENSG00000167962.14	ENSP00000455308.2	12.1	2712
RYFKTPRKF	9	0.0066	CIZ1	ENSG00000148337.21	ENSP00000398011.1	40.7	2460
LYQDQILEKF	10	0.0214	TUBE1	ENSG00000074935.14	ENSP00000357651.5	14.2	1425
ESVGGRVAER	10	53.7500	LIMA1	ENSG00000050405.13	ENSP00000448779.1	19.8	1800
AYLKDLQKL	9	0.0278	SHOC2	ENSG00000108061.11	ENSP00000358464.4	8.4	1746
SQYLTRITI	9	2.3817	ANAPC1	ENSG00000153107.13	ENSP00000339109.3	8.3	5832
EIFDKGRVR	9	27.3000	MYOM2	ENSG00000036448.10	ENSP00000262113.4	18.8	4395
YYMIGEQKF	9	0.0041	NNMT	ENSG00000166741.7	ENSP00000299964.3	9.7	792
DYLEKYYKF	9	0.0085	NNMT	ENSG00000166741.7	ENSP00000299964.3	9.7	792
YVEKVEKL	8	1.9464	AMOTL1	ENSG00000166025.18	ENSP00000387739.2	9.2	2868
AYVEKVEKL	9	0.0288	AMOTL1	ENSG00000166025.18	ENSP00000387739.2	9.2	2868
KYLEESTIRHF	11	0.0224	AMOTL1	ENSG00000166025.18	ENSP00000387739.2	9.2	2868
IYQRHVYNL	9	0.0095	TMEM209	ENSG00000146842.17	ENSP00000380747.2	7.6	1683
KYMQKSLEL	9	0.0284	TTC14	ENSG00000163728.11	ENSP00000372027.4	9.6	1959
RFIGATANF	9	0.0477	RNASEH2C	ENSG00000172922.9	ENSP00000308193.4	26.1	492
VYQSKPATL	9	0.0122	C17orf80	ENSG00000141219.15	ENSP00000268942.8	8.9	1719
LYGRNFNYL	9	0.1239	TENT4A	ENSG00000112941.14	ENSP00000230859.7	10.4	2376
EVFTGGISSY	10	10.9008	TENT4A	ENSG00000112941.14	ENSP00000230859.7	10.4	2376

DLGGLEALRQR	11	45.0000	FLYWCH1	ENSG00000059122.16	ENSP00000344122.5	31.0	1482
AYIERMNYI	9	0.0535	YES1	ENSG00000176105.14	ENSP00000324740.4	11.2	1629
YYITTRAQF	9	0.0132	YES1	ENSG00000176105.14	ENSP00000324740.4	11.2	1629
RYFDVGLHNF	10	0.0031	HHAT	ENSG00000054392.13	ENSP00000261458.3	6.8	1479
RYAVIGADL	9	0.4147	LCMT1	ENSG00000205629.12	ENSP00000382021.3	33.7	1002
EVQDLHLAQR	10	36.0000	MBOAT7	ENSG00000125505.17	ENSP00000375634.1	29.4	1032
AWAPKPYHKF	10	0.0280	MINK1	ENSG00000141503.17	ENSP00000347427.6	49.5	3996
RYPPKSGNYF	10	0.0139	MGRN1	ENSG00000102858.13	ENSP00000382487.4	28.4	1656
KYNIFRSTF	9	0.0240	ZNF181	ENSG00000197841.15	ENSP00000419435.1	9.8	1710
YYFEVVQKL	9	0.0048	CPNE3	ENSG00000085719.13	ENSP00000477590.1	50.5	1611
QYNPKFQTL	9	0.0043	YY1AP1	ENSG00000163374.19	ENSP00000352134.5	31.0	2217
TYKPNPNQI	9	0.0304	MTA3	ENSG00000057935.13	ENSP00000384249.1	14.1	1611
KYAALFSQM	9	0.0872	SAMD4B	ENSG00000179134.15	ENSP00000470237.1	57.4	2076
SYLTIHHRI	9	0.0074	ZNF12	ENSG00000164631.19	ENSP00000385939.1	10.4	2091
SYDPDFNQL	9	0.0814	FTSJ1	ENSG00000068438.15	ENSP00000326948.2	14.0	987
TQVTVQKI	8	14.9111	UVRAG	ENSG00000198382.9	ENSP00000348455.3	7.2	2097
FYIHEVQEL	9	0.0347	HERC4	ENSG00000148634.15	ENSP00000362804.4	15.9	3147
KYIGFLVRL	9	0.0258	LYL1	ENSG00000104903.4	ENSP00000264824.3	6.8	840
NYSNVSIHL	9	0.0769	CCDC82	ENSG00000149231.14	ENSP00000498266.1	10.2	1563
IYHKLKHFKF	10	0.0942	CCDC82	ENSG00000149231.14	ENSP00000498266.1	10.2	1563
IYYTGKYQSL	10	0.1767	MYCBP2	ENSG00000005810.18	ENSP00000444596.2	19.9	14034
RYIDNSICGF	10	0.0360	AC092724.1	ENSG00000140876.11	ENSP00000457566.1	22.8	510
TYSPALNKMF	10	0.0206	TP53	ENSG00000141510.17	ENSP00000482537.1	13.5	1062
EVHFPGGAER	10	19.1613	RTL5	ENSG00000242732.4	ENSP00000418667.1	7.1	1707
EAVGVTSQR	9	26.8000	TRIM24	ENSG00000122779.18	ENSP00000390829.2	7.8	3048
TYVSGTLRF	9	0.0075	CEPT1	ENSG00000134255.14	ENSP00000477632.1	12.7	840
KYTEITATYF	10	0.0092	AGL	ENSG00000162688.17	ENSP00000355106.3	11.0	4596

YYSHLEGARF	10	0.0970	URB1	ENSG00000142207.7	ENSP00000372199.3	5.9	6813
SYATLLHVL	9	0.0234	GPR39	ENSG00000183840.7	ENSP00000327417.3	6.1	1359
IYQKAFEHL	9	0.0057	BBS4	ENSG00000140463.14	ENSP00000268057.4	15.5	1557
QYASAFHFL	9	0.0114	BBS4	ENSG00000140463.14	ENSP00000268057.4	15.5	1557
RYLDASNEL	9	0.0559	NEK4	ENSG00000114904.13	ENSP00000233027.5	10.3	2523
RYIEELQKF	9	0.0011	RALGPS1	ENSG00000136828.19	ENSP00000259351.5	13.6	1671
TYAPAIHQI	9	0.0020	ARID2	ENSG00000189079.17	ENSP00000335044.6	6.3	5505
KYLINLETL	9	0.0319	JAK2	ENSG00000096968.13	ENSP00000371067.3	7.3	3396
NYSRNFSSF	9	0.0183	PDZD2	ENSG00000133401.16	ENSP00000402033.1	17.6	8517
KYQKGISLW	9	0.0113	TRAM1L1	ENSG00000174599.5	ENSP00000309402.4	5.7	1107
KYPLLLQSI	9	0.0188	ARHGEF1	ENSG00000076928.17	ENSP00000470715.1	56.1	2904
IYKIDFVRF	9	0.0145	UNC119	ENSG00000109103.11	ENSP00000337040.3	14.8	720
PYVGGLEEF	9	0.0597	TEP1	ENSG00000129566.13	ENSP00000262715.5	12.8	7881
YVIESARQR	9	18.2895	SUPT20H	ENSG00000102710.20	ENSP00000419754.1	22.4	2199
KYPGHLAAITL	11	0.1704	ACTR5	ENSG00000101442.10	ENSP00000243903.4	5.6	1821
AYSPAIQMF	9	0.0013	UGGT2	ENSG00000102595.20	ENSP00000365938.3	10.5	4548
AYLVHIEHL	9	0.0239	MCC	ENSG00000171444.18	ENSP00000305617.4	6.6	2487
GWYDEEHPLVF	11	0.2655	CLPB	ENSG00000162129.14	ENSP00000441518.1	7.6	2031
NYAAALETF	9	0.0071	SUGT1	ENSG00000165416.14	ENSP00000367208.4	11.3	1095
KYDEGLYRF	9	0.0053	DCLRE1A	ENSG00000198924.8	ENSP00000355185.2	6.3	3120
EYVKQTWNL	9	0.0598	SYK	ENSG00000165025.15	ENSP00000364899.1	21.5	1836
KYLEESNFV	9	0.2650	SYK	ENSG00000165025.15	ENSP00000364899.1	21.5	1836
VYLDKFIRL	9	0.0061	GNL3L	ENSG00000130119.16	ENSP00000354091.2	5.9	1746
VGYRVTQI	8	18.4474	HERC5	ENSG00000138646.9	ENSP00000264350.3	5.5	3072
VYSQILRKL	9	0.0181	NUP85	ENSG00000125450.11	ENSP00000462749.1	18.3	1833
RYKGPGFEYF	10	0.0083	RPUSD2	ENSG00000166133.18	ENSP00000323288.7	9.4	1635
VYNPVRAEW	9	0.0093	KBTBD6	ENSG00000165572.7	ENSP00000368799.1	5.5	2022

VYKPAQNSW	9	0.0224	KBTBD6	ENSG00000165572.7	ENSP00000368799.1	5.5	2022
IFIQNSKLYF	10	0.1828	SLC35A5	ENSG00000138459.9	ENSP00000417654.1	9.8	1272
QQVILVQV	8	31.6667	FNDC3B	ENSG00000075420.13	ENSP00000411242.2	14.4	3612
IFTDVALKF	9	0.0213	GTF3C4	ENSG00000125484.12	ENSP00000361219.4	5.9	2466
RYTERVDYL	9	0.0795	USP13	ENSG00000058056.9	ENSP00000263966.3	5.6	2589
VYVQELQKL	9	0.0133	EEA1	ENSG00000102189.16	ENSP00000317955.8	5.8	4233
KYQHLKAEF	9	0.0187	EEA1	ENSG00000102189.16	ENSP00000317955.8	5.8	4233
KYTNIVKEM	9	0.1708	CCDC146	ENSG00000135205.15	ENSP00000285871.4	7.4	2865
KYTWHITNI	9	0.0900	C2orf42	ENSG00000115998.7	ENSP00000404515.1	8.2	1722
KYIDYIFNV	9	0.0937	HERC6	ENSG00000138642.14	ENSP00000264346.7	7.5	3066
AYLEAHETF	9	0.0026	NUP107	ENSG00000111581.10	ENSP00000229179.4	6.6	2775
QYEKLFHKW	9	0.0203	ORC2	ENSG00000115942.9	ENSP00000234296.2	7.9	1731
VYLKKLKKL	9	0.0587	LEMD3	ENSG00000174106.3	ENSP00000308369.2	16.9	2733
PYFNAPVYL	9	0.0836	GAR1	ENSG00000109534.17	ENSP00000378127.3	18.1	651
QYMELFNKI	9	0.0087	MED20	ENSG00000124641.16	ENSP00000265350.4	11.1	636
SMPDVLKI	8	3.5362	TASOR	ENSG00000163946.13	ENSP00000399410.2	16.3	3699
NQLVALVR	8	53.7500	MON1A	ENSG00000164077.14	ENSP00000296473.3	13.0	1956
TYTQDFNKF	9	0.0084	CALCA	ENSG00000110680.12	ENSP00000331746.4	8.1	423
IYPTAPPRF	9	0.0031	LYPLAL1	ENSG00000143353.12	ENSP00000355894.3	22.2	663
KMLDNFYNF	9	0.0692	HIKESHI	ENSG00000149196.16	ENSP00000482151.1	14.5	393
RQHVLTRI	8	14.1000	CHD4	ENSG00000111642.15	ENSP00000496543.1	51.5	5697
HYDPTINKW	9	0.0182	ENC1	ENSG00000171617.14	ENSP00000306356.4	5.4	1767
AYCLKYEKF	9	0.0574	ASB3	ENSG00000115239.22	ENSP00000378206.2	10.8	1335
AYVEWFNRL	9	0.0344	RASGEF1B	ENSG00000138670.17	ENSP00000425393.1	13.4	1416
SYISKVDVL	9	0.0520	ZFYVE26	ENSG00000072121.16	ENSP00000251119.5	8.1	7617
RYQEVIQEL	9	0.0036	ZFYVE26	ENSG00000072121.16	ENSP00000251119.5	8.1	7617
KYQPRIAVF	9	0.0027	TDG	ENSG00000139372.15	ENSP00000376611.3	8.9	1230

IYNQVKQII	9	0.0475	DLG1	ENSG00000075711.21	ENSP00000499602.1	31.3	2613
IYTFPHASF	9	0.0101	BTN3A3	ENSG00000111801.16	ENSP00000244519.2	14.0	1752
RYAEAVQLL	9	0.0058	TTC30A	ENSG00000197557.7	ENSP00000347915.4	5.1	1995
NYIEKVVAI	9	0.0630	ABI1	ENSG00000136754.17	ENSP00000365309.2	23.8	1428
HYHPIVQRF	9	0.0015	NOC3L	ENSG00000173145.11	ENSP00000360412.3	5.5	2400
QYLLHLNNL	9	0.1021	NSMAF	ENSG00000035681.9	ENSP00000038176.3	11.8	2751
HSTIMPRL	8	16.4909	MCM4	ENSG00000104738.18	ENSP00000497093.1	17.0	2247
TYIQKIFRM	9	0.0217	ACOX3	ENSG00000087008.16	ENSP00000413994.2	12.7	1872
KYLLHSLVF	9	0.0206	ACOX3	ENSG00000087008.16	ENSP00000413994.2	12.7	1872
EVFDKDLSKL	10	6.6548	DAAM1	ENSG00000100592.15	ENSP00000378557.1	16.9	3234
KYMEDVTQI	9	0.0173	PPM1D	ENSG00000170836.11	ENSP00000306682.2	6.8	1815
RFEEAHFTF	9	0.0219	PIAS3	ENSG00000131788.16	ENSP00000376765.2	20.1	1884
KYNLVTHIL	9	0.0219	ZNF710	ENSG00000140548.10	ENSP00000268154.3	6.4	1992
EVITKFINV	9	9.9000	RASGRP3	ENSG00000152689.18	ENSP00000385886.3	11.8	2070
AYIQEFQEF	9	0.0029	OLFML1	ENSG00000183801.8	ENSP00000332511.3	6.8	1206
VYTSGVYHI	9	0.0076	TNS2	ENSG00000111077.17	ENSP00000319756.3	85.1	4257
RYISYFSGL	9	0.1287	TNS2	ENSG00000111077.17	ENSP00000319756.3	85.1	4257
AYLNITKHL	9	0.0573	POLK	ENSG00000122008.15	ENSP00000423526.1	9.7	1281
TYPAELNNI	9	0.0939	AHR	ENSG00000106546.14	ENSP00000242057.4	5.2	2544
TYLNYVVHL	9	0.0154	ZNF292	ENSG00000188994.13	ENSP00000358590.3	5.9	8169
HYPPVQVLF	9	0.0012	MSH6	ENSG00000116062.15	ENSP00000438580.1	7.8	3174
TYVTILPEL	9	0.0485	XRN1	ENSG00000114127.10	ENSP00000264951.4	7.3	5118
IMPSSSHLF	9	0.0176	XRN1	ENSG00000114127.10	ENSP00000264951.4	7.3	5118
RYAWIKRQL	9	0.1307	VPS53	ENSG00000141252.20	ENSP00000401435.2	6.8	2496
QYGEVANLL	9	0.0729	VPS53	ENSG00000141252.20	ENSP00000401435.2	6.8	2496
EAFPSQGTKR	10	18.0000	VPS53	ENSG00000141252.20	ENSP00000401435.2	6.8	2496
KYPSDLPYM	9	0.0753	ERG	ENSG00000157554.19	ENSP00000288319.7	10.5	1437

VYSLIKNKI	9	0.0528	PRKAG2	ENSG00000106617.14	ENSP00000499103.1	24.8	984
QQTELSRI	8	24.8571	AKAP9	ENSG00000127914.16	ENSP00000350813.3	39.3	9378
KYITTTLFL	9	0.0306	SLC4A11	ENSG00000088836.14	ENSP00000369396.3	19.6	2673
RYISQPPLL	9	0.0072	EDEM3	ENSG00000116406.19	ENSP00000318147.7	6.3	2796
RYLERLIKL	9	0.0121	AC006538.1	ENSG00000172009.15	ENSP00000304467.5	14.6	2067
KYPHYFPLL	9	0.0063	AC006538.1	ENSG00000172009.15	ENSP00000304467.5	14.6	2067
KYFLKPEVL	9	0.0347	NCEH1	ENSG00000144959.10	ENSP00000442464.1	13.5	1344
KYLEETNSL	9	0.0234	L3MBTL3	ENSG00000198945.7	ENSP00000437185.1	6.5	2265
VFVDSVPEF	9	0.0235	INTS9	ENSG00000104299.15	ENSP00000430943.1	10.5	1902
TYARNLPAF	9	0.0080	TTI2	ENSG00000129696.12	ENSP00000428401.1	9.8	1431
KYIDFDHVF	9	0.0027	CRAMP1	ENSG00000007545.15	ENSP00000380559.2	7.9	3807
RYDPHLNRW	9	0.0184	KLHL26	ENSG00000167487.12	ENSP00000300976.3	10.7	1845
TYITSVSRL	9	0.0426	OSGIN2	ENSG00000164823.11	ENSP00000297438.2	9.2	1515
RYLSKVLEL	9	0.0106	C15orf40	ENSG00000169609.14	ENSP00000403987.3	15.4	501
IYTKIMDLI	9	0.0965	DPY19L1	ENSG00000173852.14	ENSP00000490722.1	5.2	2244
QQVQIHQI	8	11.8478	BANP	ENSG00000172530.20	ENSP00000487304.1	15.6	1491
KYVKVFHKF	9	0.0007	ZNF680	ENSG00000173041.12	ENSP00000309330.6	6.2	1590
RYKEENNDHL	10	0.4608	USP8	ENSG00000138592.14	ENSP00000412682.3	23.8	3036
KYPHYFPVM	9	0.0445	NLN	ENSG00000123213.23	ENSP00000423214.1	8.2	1800
RYLNKAFHIW	10	0.0835	NCAPD3	ENSG00000151503.12	ENSP00000433681.2	8.6	4494
LYSEVSHLL	9	0.0087	RICTOR	ENSG00000164327.13	ENSP00000349959.3	6.7	5124
KYVKVFHQF	9	0.0007	ZNF486	ENSG00000256229.8	ENSP00000335042.7	4.9	1389
TYISWKEEL	9	0.0517	VPS13C	ENSG00000129003.18	ENSP00000249837.3	16.5	11130
IYHPDIQLL	9	0.0039	VPS13C	ENSG00000129003.18	ENSP00000249837.3	16.5	11130
KYLEQQFNL	9	0.0194	SLCO3A1	ENSG00000176463.14	ENSP00000320634.6	12.4	2130
EVMLRVKEM	9	11.2446	CDYL2	ENSG00000166446.15	ENSP00000476295.1	4.7	1518
KYFLWEEKF	9	0.0073	GRAP	ENSG00000154016.13	ENSP00000284154.5	5.1	651

NYGDQVQHF	9	0.0077	GRAP	ENSG00000154016.13	ENSP00000284154.5	5.1	651
ETISTATGR	9	36.3333	TM7SF2	ENSG00000149809.14	ENSP00000279263.7	29.1	1254
KYMLKANLI	9	0.0535	NAA15	ENSG00000164134.13	ENSP00000381920.1	6.9	2595
DVHNFPGTSR	10	24.8571	KPTN	ENSG00000118162.14	ENSP00000337850.2	12.1	1308
RYTLQASTF	9	0.0214	CUL1	ENSG00000055130.17	ENSP00000499276.1	30.2	2277
YYLNEIQSF	9	0.0017	SPATC1L	ENSG00000160284.15	ENSP00000333869.6	7.3	558
SYAKGLQKL	9	0.0186	NOSTRIN	ENSG00000163072.15	ENSP00000318921.7	27.2	1518
IYADNQVMHF	10	0.0050	POLE	ENSG00000177084.16	ENSP00000322570.5	6.7	6858
SNQMLVPVI	9	10.3015	WDR3	ENSG00000065183.16	ENSP00000308179.4	5.8	2829
PYADQVFRI	9	0.0718	HELZ	ENSG00000198265.12	ENSP00000351524.5	11.3	5826
HYILHNSFF	9	0.0389	TIPARP	ENSG00000163659.13	ENSP00000420612.1	7.1	1971
SYLRESSLL	9	0.0912	FLI1	ENSG00000151702.17	ENSP00000433488.2	11.0	1356
AYPTVKFYF	9	0.0076	DNAJC10	ENSG00000077232.18	ENSP00000264065.6	8.1	2379
KYMDVVKERI	10	0.1639	DDX24	ENSG00000089737.17	ENSP00000452145.1	37.4	2448
LYGQVQVF	8	0.2918	NOL9	ENSG00000162408.11	ENSP00000366934.5	5.5	2106
VYTDFAFRI	9	0.0366	NOL9	ENSG00000162408.11	ENSP00000366934.5	5.5	2106
AVIEQEVRER	10	35.7500	CC2D2A	ENSG00000048342.16	ENSP00000421809.1	36.6	4860
VYATMPINSF	10	0.0113	ZNF148	ENSG00000163848.19	ENSP00000353863.4	7.2	2382
HVVGQGVLHR	10	35.0000	OGFOD2	ENSG00000111325.16	ENSP00000442476.1	19.6	480
KYPVLFQRI	9	0.0107	AKAP13	ENSG00000170776.21	ENSP00000378026.2	25.5	8439
SYIDVAVKL	9	0.0170	TXNDC16	ENSG00000087301.9	ENSP00000281741.4	4.4	2475
EIMEVAKER	9	26.0000	ESS2	ENSG00000100056.12	ENSP00000252137.6	11.1	1428
IFHEKTSEF	9	0.0529	SLC7A2	ENSG00000003989.17	ENSP00000419140.1	4.6	1974
TYLDKIKKF	9	0.0058	SYNE2	ENSG00000054654.16	ENSP00000341781.4	137.7	20655
KYLKAVEEL	9	0.0180	SYNE2	ENSG00000054654.16	ENSP00000341781.4	137.7	20655
TYVENLRLL	9	0.0305	SYNE2	ENSG00000054654.16	ENSP00000341781.4	137.7	20655
SYMMRAQQL	9	0.0725	SYNE2	ENSG00000054654.16	ENSP00000341781.4	137.7	20655

STISEVLGR	9	21.3636	ZFAT	ENSG00000066827.16	ENSP00000428483.1	19.9	3693
FYILTSKEL	9	0.1829	STK17B	ENSG00000081320.11	ENSP00000263955.4	4.7	1116
FEEEESDKRE	10	67.5000	MCCC1	ENSG00000078070.13	ENSP00000419898.1	121.9	1848
EVSWKVLER	9	21.6364	PHKB	ENSG00000102893.16	ENSP00000313504.5	60.8	3279
KYLEHPLLL	9	0.0043	TTL	ENSG00000114999.8	ENSP00000233336.5	4.8	1131
VYAEVSRLL	9	0.0155	TTL	ENSG00000114999.8	ENSP00000233336.5	4.8	1131
VYAEVSRLLL	10	0.1287	TTL	ENSG00000114999.8	ENSP00000233336.5	4.8	1131
VYMPASSYQSL	11	0.0476	FAM126B	ENSG00000155744.9	ENSP00000393667.2	5.5	1590
VLPGHFNTF	9	0.0411	MBLAC2	ENSG00000176055.10	ENSP00000314776.6	4.3	837
TYMDRVFKQF	10	0.0085	ABHD3	ENSG00000158201.10	ENSP00000462578.1	10.2	642
PWPKVLKI	8	1.6966	EPB41	ENSG00000159023.21	ENSP00000497875.1	14.6	1626
EVITAVRKM	9	8.2245	COL6A3	ENSG00000163359.15	ENSP00000418285.1	11.0	7710
KYPKVWEGF	9	0.0047	SYMPK	ENSG00000125755.18	ENSP00000470957.1	42.4	285
VYCLRTEYI	9	0.2183	TOPBP1	ENSG00000163781.13	ENSP00000260810.5	7.5	4566
KYKDAANLL	9	0.0484	KLC1	ENSG00000126214.21	ENSP00000452487.1	42.1	1680
VQPSLFYHL	9	0.2026	MAP3K6	ENSG00000142733.17	ENSP00000363152.2	13.9	3840
SYLNSVQRL	9	0.0162	NUPL2	ENSG00000136243.17	ENSP00000415511.1	16.7	672
KYITDVVKL	9	0.0227	UTP20	ENSG00000120800.5	ENSP00000261637.4	4.0	8355
KYPVEWAKF	9	0.0055	ZC2HC1A	ENSG00000104427.12	ENSP00000263849.3	4.6	975
KYRNIGSETW	10	0.1997	IL13RA2	ENSG00000123496.8	ENSP00000243213.1	7.0	1140
RYKPGEPITF	10	0.0074	DENND1B	ENSG00000213047.13	ENSP00000479816.1	6.2	2325
VYTLLTTHL	9	0.0290	C11orf80	ENSG00000173715.16	ENSP00000494645.1	15.5	1731
IYLPEVRKI	9	0.0098	TULP4	ENSG00000130338.13	ENSP00000356064.3	10.1	4629
VYSEKVMHMF	10	0.0071	MYO18A	ENSG00000196535.16	ENSP00000437073.1	28.1	6162
ETASKTLSR	9	15.2754	AC124319.1	ENSG00000173821.19	ENSP00000425956.2	40.9	15768
IYPQVLHSL	9	0.0045	AC124319.1	ENSG00000173821.19	ENSP00000425956.2	40.9	15768
KYLKVFYKF	9	0.0014	ZNF254	ENSG00000213096.10	ENSP00000349494.3	4.8	1977

EQPYKWEKF	9	0.5648	ZNF254	ENSG00000213096.10	ENSP00000349494.3	4.8	1977
NYSSAVQKF	9	0.0051	OPHN1	ENSG00000079482.13	ENSP00000347710.5	5.0	2406
KYPDIISRI	9	0.0067	LIG1	ENSG00000105486.14	ENSP00000483027.1	10.6	2403
YAEKLHRL	8	1.3663	DST	ENSG00000151914.20	ENSP00000498983.1	35.8	7149
TYAEKLHRL	9	0.0099	DST	ENSG00000151914.20	ENSP00000498983.1	35.8	7149
RYAEQISVL	9	0.0454	CCDC121	ENSG00000176714.9	ENSP00000339087.2	4.7	834
RYQALFHDF	9	0.0059	VCPIP1	ENSG00000175073.8	ENSP00000309031.4	3.7	3666
TYLVDMQRF	9	0.0095	FZD2	ENSG00000180340.6	ENSP00000323901.3	3.7	1695
VYSFVTPTF	9	0.0016	PLXNA3	ENSG00000130827.6	ENSP00000358696.3	6.9	5613
AYAPVLQDF	9	0.0033	F8	ENSG00000185010.15	ENSP00000353393.4	9.6	7053
EYAKIFTTY	9	0.1573	LPCAT2	ENSG00000087253.13	ENSP00000262134.5	5.2	1632
TYISAIRERF	10	0.0194	NXF1	ENSG00000162231.14	ENSP00000294172.2	53.2	1857
IYSTKLYRF	9	0.0022	BTBD10	ENSG00000148925.10	ENSP00000435257.1	9.1	1281
KYGLFQHI	8	0.4548	DHCR24	ENSG00000116133.13	ENSP00000360316.3	21.8	1548
EVIAYILER	9	22.2632	MED23	ENSG00000112282.18	ENSP00000357047.3	12.8	4104
VYITGKEVF	9	0.0138	VPS13A	ENSG00000197969.14	ENSP00000496361.1	10.0	9207
EVFSFKMVSY	10	14.2667	VPS13A	ENSG00000197969.14	ENSP00000496361.1	10.0	9207
TYSEQWQQL	9	0.0200	FES	ENSG00000182511.12	ENSP00000331504.3	12.5	2466
TYHNWRHGF	9	0.0525	PDE6B	ENSG00000133256.12	ENSP00000420295.1	8.4	2562
RFQERFFLL	9	0.0866	ARAP3	ENSG00000120318.16	ENSP00000421468.1	14.2	3579
EVAEKIWSNR	10	27.1000	ARAP3	ENSG00000120318.16	ENSP00000421468.1	14.2	3579
KYGVFEESL	9	0.2812	TRIM6-TRIM34	ENSG00000258588.3	ENSP00000346916.5	3.4	2526
RYMEHLEKI	9	0.0075	KIAA0895	ENSG00000164542.12	ENSP00000319251.6	7.2	1407
IQMSVQRI	8	6.7323	POLR2B	ENSG00000047315.16	ENSP00000370625.1	45.0	3522
NYVQAVEEF	9	0.0143	NASP	ENSG00000132780.17	ENSP00000438871.1	35.3	2172
RYPDNLKHL	9	0.0512	CCDC59	ENSG00000133773.12	ENSP00000256151.7	19.5	723
RYPDNLKHLYL	11	0.1569	CCDC59	ENSG00000133773.12	ENSP00000256151.7	19.5	723

KYPLLISRI	9	0.0231	ARHGEF2	ENSG00000116584.18	ENSP00000315325.7	35.8	2874
KYFSDGSSNTF	11	0.0197	BTBD3	ENSG00000132640.15	ENSP00000384545.1	13.2	1566
VYSFGFERL	9	0.0620	SLC30A6	ENSG00000152683.14	ENSP00000384041.1	6.3	1161
RYAQQKHRI	9	0.0664	HDAC6	ENSG00000094631.20	ENSP00000496013.1	149.5	3645
IYKWITDNF	9	0.0111	FOXJ1	ENSG00000129654.8	ENSP00000323880.4	3.3	1263
YYITTRVQF	9	0.0072	FGR	ENSG00000000938.13	ENSP00000363115.3	7.6	1587
RYTTEFHEL	9	0.0150	WEE1	ENSG00000166483.11	ENSP00000402084.2	6.9	1938
IYIINVHSM	9	0.0272	DENND3	ENSG00000105339.10	ENSP00000410594.1	16.2	3438
SQLRTTVV	8	23.1875	MTSS1	ENSG00000170873.18	ENSP00000429064.1	62.4	2265
VYTYIQSRF	9	0.0084	DYRK2	ENSG00000127334.10	ENSP00000342105.3	4.8	1803
YTADPYHAL	9	1.0020	RBFOX2	ENSG00000100320.23	ENSP00000405651.2	21.1	1128
VYTLYTHSF	9	0.0023	ENTPD3	ENSG00000168032.9	ENSP00000301825.3	3.3	1587
YQFGEIRTI	9	1.3180	RBM22	ENSG00000086589.12	ENSP00000412118.2	32.4	1113
KYVKVIHKF	9	0.0009	AC008554.1	ENSG00000237440.9	ENSP00000395733.3	4.8	1608
EYVKFLHKF	9	0.0036	ZNF506	ENSG00000081665.14	ENSP00000440625.1	10.6	1332
SIVDSILMER	10	27.5000	RBM25	ENSG00000119707.14	ENSP00000261973.7	22.5	2529
AVVPFLPLQR	10	20.0000	TOR2A	ENSG00000160404.18	ENSP00000362381.5	6.4	963
TYQEVAQKF	9	0.0014	ADA2	ENSG00000093072.18	ENSP00000496894.1	11.8	207
KFVETHPEF	9	0.0294	ADA2	ENSG00000093072.18	ENSP00000496894.1	11.8	207
IYTKLNQKI	9	0.0210	EXOC6	ENSG00000138190.16	ENSP00000260762.6	5.6	2412
EVIRTLPSL	9	4.4804	INPP5D	ENSG00000168918.14	ENSP00000405338.2	8.5	3567
RYLADLPTL	9	0.0275	CEP85	ENSG00000130695.15	ENSP00000252992.4	7.4	2286
KYPELFANL	9	0.0117	PEX1	ENSG00000127980.16	ENSP00000394413.1	15.7	3678
MYINEVERL	9	0.0286	PTPN14	ENSG00000152104.12	ENSP00000355923.4	4.3	3561
AQYKFVYQV	9	2.6444	PTPN14	ENSG00000152104.12	ENSP00000355923.4	4.3	3561
YYAKEIHKF	9	0.0007	TGFB3	ENSG00000119699.7	ENSP00000238682.3	7.2	1236
LYKPKQEVKF	10	0.0920	CD109	ENSG00000156535.15	ENSP00000388062.2	10.9	4284

IYLRDPVQV	9	0.1470	TPP2	ENSG00000134900.12	ENSP00000365220.3	11.2	3786
TYERGTYYF	9	0.0164	AC092881.1	ENSG00000111596.12	ENSP00000449260.1	19.4	1620
FTAPSTVGKR	10	27.5000	PARN	ENSG00000140694.17	ENSP00000498650.1	20.9	1743
TQYIFTEKL	9	2.3914	NAF1	ENSG00000145414.9	ENSP00000274054.2	7.9	1482
RYQTKFRHL	9	0.0887	CEP89	ENSG00000121289.18	ENSP00000465141.1	16.8	1284
RYTEITREKF	10	0.0135	CEP89	ENSG00000121289.18	ENSP00000465141.1	16.8	1284
RYILTLHEL	9	0.0085	RASGRF2	ENSG00000113319.13	ENSP00000265080.4	5.3	3711
AYQAVVWKI	9	0.0383	STON1	ENSG00000243244.6	ENSP00000384615.1	6.9	2205
EAIGTLTAR	9	26.1000	APBB3	ENSG00000113108.19	ENSP00000346378.5	19.8	1479
DYKRLFFRF	9	0.1027	TRANK1	ENSG00000168016.14	ENSP00000494480.1	4.0	8907
VYENVSHFL	9	0.0503	SLC25A32	ENSG00000164933.12	ENSP00000297578.4	8.9	945
TYLSIIHSL	9	0.0045	MPRIP	ENSG00000133030.21	ENSP00000317786.6	74.7	5382
SYIEVSHGL	9	0.0316	SYNPO2	ENSG00000172403.11	ENSP00000425496.1	7.9	3465
YYEKLHTYF	9	0.0121	ITGB4	ENSG00000132470.14	ENSP00000200181.3	8.3	5466
KYGKVWGF	8	0.2160	CYP3A4	ENSG00000160868.15	ENSP00000337915.3	5.1	1602
KYWTEPEKF	9	0.0062	CYP3A4	ENSG00000160868.15	ENSP00000337915.3	5.1	1602
EIIQAQLVNR	10	43.0000	CEP63	ENSG00000182923.17	ENSP00000336524.3	15.8	2109
NYLTKIYGL	9	0.1445	PODN	ENSG00000174348.13	ENSP00000308315.5	3.7	1983
TYKDKMTFF	9	0.0248	SRL	ENSG00000185739.13	ENSP00000382518.3	3.2	1419
EYDLIVHQL	9	0.1080	VPS26A	ENSG00000122958.15	ENSP00000263559.6	30.8	981
TYPDVNNSI	9	0.0636	VPS26A	ENSG00000122958.15	ENSP00000263559.6	30.8	981
VLPQRFPQF	9	0.0393	CNOT4	ENSG00000080802.18	ENSP00000399108.2	11.3	1716
KYVKVFNKF	9	0.0017	ZNF675	ENSG00000197372.9	ENSP00000352836.3	4.2	1704
KYLTAASYL	9	0.0951	ZBTB6	ENSG00000186130.5	ENSP00000362763.3	2.9	1272
RYLDEINLL	9	0.0090	GNA15	ENSG00000060558.3	ENSP00000262958.2	2.9	1122
AYTLLLHTW	9	0.0151	DOCK2	ENSG00000134516.17	ENSP00000429283.2	4.8	5490
RYLPSHRLI	9	0.0189	GIMAP6	ENSG00000133561.15	ENSP00000418304.1	14.4	186

NYIKDLNIL	9	0.1257	CTDSPL2	ENSG00000137770.14	ENSP00000260327.4	4.8	1398
SYFSGSHMF	9	0.0020	CCNJL	ENSG00000135083.15	ENSP00000257536.7	3.3	1161
SYVNHLYVI	9	0.0396	RAPGEF5	ENSG00000136237.19	ENSP00000343656.6	11.8	2190
YDTVFKHF	8	0.8126	SP140L	ENSG00000185404.16	ENSP00000395195.1	6.4	1314
VYDTVFKHF	9	0.0053	SP140L	ENSG00000185404.16	ENSP00000395195.1	6.4	1314
IYIRYLYKL	9	0.0139	DOCK5	ENSG00000147459.18	ENSP00000276440.7	4.1	5610
RYLPQCSYF	9	0.0063	CCND2	ENSG00000118971.8	ENSP00000261254.3	3.1	867
EYIPKWEQF	9	0.0029	C3orf14	ENSG00000114405.10	ENSP00000232519.5	6.6	384
RYSGNQVLF	9	0.0057	RAPGEF6	ENSG00000158987.20	ENSP00000421684.1	4.1	4803
HVRDSYSQM	9	9.0583	ATP9B	ENSG00000166377.20	ENSP00000398076.2	15.7	3441
KYSSGFRNI	9	0.0808	ATM	ENSG00000149311.18	ENSP00000435747.1	13.5	4107
TYVIYAHLL	9	0.0434	HECTD2	ENSG00000165338.16	ENSP00000298068.5	4.3	2328
KYNANFVTF	9	0.0016	KDM2A	ENSG00000173120.15	ENSP00000432786.1	45.1	3486
EFVAKGHLF	9	0.1996	MED18	ENSG00000130772.14	ENSP00000362948.4	5.9	624
EYFRVPDSATF	11	0.0650	VPS13B	ENSG00000132549.18	ENSP00000349685.2	7.9	11991
KYLSGLVQW	9	0.0113	EMC3	ENSG00000125037.12	ENSP00000408803.1	40.5	609
RYGGPLGITI	10	0.1872	GRIP2	ENSG00000144596.13	ENSP00000481670.2	5.4	996
RYAVYWDEL	9	0.2374	DDHD2	ENSG00000085788.13	ENSP00000380352.2	24.5	2133
KYVKIVEKW	9	0.0083	RWDD3	ENSG00000122481.17	ENSP00000263893.6	11.0	585
AYHNWRHAF	9	0.0553	PDE5A	ENSG00000138735.16	ENSP00000347046.3	6.3	2625
VYEYVVERF	9	0.0088	TUT4	ENSG00000134744.14	ENSP00000257177.4	19.1	4935
KYLDVKFEYL	10	0.0921	PICK1	ENSG00000100151.16	ENSP00000385205.3	31.8	1245
SYLIQHQRF	9	0.0091	ZNF71	ENSG00000197951.9	ENSP00000328245.5	3.7	1467
VYERELQTF	9	0.0106	EEF1AKMT2	ENSG00000203791.15	ENSP00000357829.2	5.2	873
RYDNVTILF	9	0.0074	GUCY1B1	ENSG00000061918.13	ENSP00000264424.8	16.4	1857
TYHPGVPVF	9	0.0025	CHORDC1	ENSG00000110172.12	ENSP00000319255.6	8.1	996
EYLKHHNRI	9	0.1628	GZF1	ENSG00000125812.16	ENSP00000366250.2	5.3	2133

YLEKAIKI	8	2.3986	USP7	ENSG00000187555.15	ENSP00000343535.4	39.7	3306
TYLEKAIKI	9	0.0206	USP7	ENSG00000187555.15	ENSP00000343535.4	39.7	3306
RYNHITATY	9	0.1472	SNRK	ENSG00000163788.14	ENSP00000296088.7	6.6	2295
RYNHITATYF	10	0.0305	SNRK	ENSG00000163788.14	ENSP00000296088.7	6.6	2295
TYPQLEGFKF	10	0.0214	WDFY4	ENSG00000128815.19	ENSP00000320563.5	3.7	9552
KYTAVVKMI	9	0.1167	KIN	ENSG00000151657.12	ENSP00000368881.3	3.3	1179
KYIENLDNITF	11	0.0112	ZFYVE16	ENSG00000039319.17	ENSP00000426848.1	9.3	4617
EYLGKLVRF	9	0.0124	RSAD2	ENSG00000134321.12	ENSP00000371471.3	3.0	1083
IYEQVFAKL	9	0.0513	NRDE2	ENSG00000119720.18	ENSP00000346335.3	6.1	3492
SYANVQDGF	9	0.0646	ANKRD22	ENSG00000152766.6	ENSP00000360998.4	2.6	573
KYQNVKHNL	9	0.0261	ZMYM1	ENSG00000197056.11	ENSP00000352920.4	3.5	3426
KYITEVSRW	9	0.0214	CEP135	ENSG00000174799.10	ENSP00000257287.3	4.6	3420
SFTQKSHLF	9	0.0377	ZNF175	ENSG00000105497.8	ENSP00000262259.2	3.2	2133
IYKDSSTFL	9	0.0775	METTL14	ENSG00000145388.15	ENSP00000373474.3	8.0	1368
YYNPVTDKW	9	0.0049	KLHL3	ENSG00000146021.15	ENSP00000312397.4	8.4	1761
NYENKQYLF	9	0.0346	WDR66	ENSG00000158023.10	ENSP00000380595.2	7.8	2823
EYNKSWQTF	9	0.0089	ZNF583	ENSG00000198440.9	ENSP00000291598.7	3.1	1707
NYKSHHLQL	9	0.1715	CWF19L1	ENSG00000095485.18	ENSP00000326411.6	8.5	1614
SYVHKVPSF	9	0.0040	MED13L	ENSG00000123066.8	ENSP00000281928.3	16.7	6630
TYEAQFFGF	9	0.0574	MIS12	ENSG00000167842.15	ENSP00000461252.1	6.6	615
KYMETIEKL	9	0.0049	PPP1R21	ENSG00000162869.16	ENSP00000281394.4	40.5	2307
KYFALPHKI	9	0.0062	TUT7	ENSG00000083223.18	ENSP00000365130.3	7.9	4485
SYTANLAAF	9	0.0495	GRIK5	ENSG00000105737.9	ENSP00000470251.1	12.2	2940
VYAVVRGLF	9	0.0388	MFSD13A	ENSG00000138111.14	ENSP00000238936.3	4.7	1551
KYNDFGNSF	9	0.0084	ZNF3	ENSG00000166526.17	ENSP00000299667.4	12.3	1338
RYSAGWDAKFF	11	0.3270	HSD17B6	ENSG00000025423.11	ENSP00000318631.1	2.3	951
RWFQPAIPSW	10	0.0985	UNC13D	ENSG00000092929.11	ENSP00000207549.3	5.8	3270

TYNKHINISF	10	0.0544	THAP2	ENSG00000173451.7	ENSP00000310796.2	3.4	684
TFTFSHATF	9	0.0994	TRIM27	ENSG00000204713.11	ENSP00000366404.3	14.6	1539
EVSFYNVTER	10	47.0000	TRIM27	ENSG00000204713.11	ENSP00000366404.3	14.6	1539
EIKERIAKR	9	22.6842	BIN2	ENSG00000110934.11	ENSP00000483983.2	5.3	1695
TYLEKAVEV	9	0.2187	ATM	ENSG00000149311.18	ENSP00000278616.4	13.5	9168
KYVSSVLSF	9	0.0043	LTN1	ENSG00000198862.14	ENSP00000478783.1	7.3	5436
SYLENPTSY	9	0.1439	TFEB	ENSG00000112561.18	ENSP00000343948.6	26.4	1686
KYTEKLEEI	9	0.0543	CEP41	ENSG00000106477.19	ENSP00000342738.5	6.2	903
LYKDVRDLL	9	0.1675	ANKZF1	ENSG00000163516.13	ENSP00000321617.5	23.1	2178
KYVKVFHKY	9	0.0542	ZNF708	ENSG00000182141.10	ENSP00000349401.2	4.1	1689
SYLDVKGNHF	10	0.0117	LRRC8C	ENSG00000171488.15	ENSP00000359483.4	2.2	2409
RYQEMIQKL	9	0.0039	SCLT1	ENSG00000151466.12	ENSP00000281142.5	2.9	2064
RYIHKLYDL	9	0.0583	DOCK4	ENSG00000128512.22	ENSP00000499282.1	10.3	2694
MYIRYIHKL	9	0.0063	DOCK4	ENSG00000128512.22	ENSP00000499282.1	10.3	2694
LYAKIAEAF	9	0.0114	GIPC3	ENSG00000179855.8	ENSP00000493901.1	2.3	936
NYPVWDTQF	9	0.0277	TAF5	ENSG00000148835.11	ENSP00000358854.3	2.0	2400
QYAVLLNRF	9	0.0115	NSUN3	ENSG00000178694.10	ENSP00000318986.4	3.9	1020
ELLHFQASQR	10	43.5000	IKBKG	ENSG00000269335.5	ENSP00000471166.1	8.0	1257
KYLSHDVV	8	4.2269	SLC38A6	ENSG00000139974.15	ENSP00000437190.1	10.1	687
RYLPKGFLNQF	11	0.0095	PTK2	ENSG00000169398.19	ENSP00000429082.1	62.1	3288
KYMEGSKLHYF	11	0.0054	PIGW	ENSG00000277161.2	ENSP00000482202.1	2.4	1512
VYWDSAGAAHF	11	0.0353	CHST12	ENSG00000136213.10	ENSP00000411207.1	3.6	735
IYKEDFNEL	9	0.0365	WDR17	ENSG00000150627.15	ENSP00000422763.1	4.2	3849
EYMIQFNRL	9	0.0961	HELZ2	ENSG00000130589.16	ENSP00000393257.2	4.6	6240
NYIEGTKLF	9	0.0106	CNDP1	ENSG00000150656.15	ENSP00000351682.3	2.7	1521
KYFDKVVTL	9	0.0039	KIF7	ENSG00000166813.15	ENSP00000377934.3	2.7	4029
NYIPYLTKL	9	0.0120	SAMD9	ENSG00000205413.8	ENSP00000369292.2	2.7	4767

EYNNNFHVL	9	0.0774	IPMK	ENSG00000151151.6	ENSP00000363046.3	1.8	1248
EVVNYVQKR	9	28.0000	RMI1	ENSG00000178966.16	ENSP00000317039.3	4.8	1875
QYIDVSHVF	9	0.0019	RFX2	ENSG00000087903.13	ENSP00000306335.4	4.0	2169
RYDTITNQW	9	0.0190	KLHL29	ENSG00000119771.15	ENSP00000420659.1	4.5	2625
AFIKKSHLL	9	0.1565	ZNF684	ENSG00000117010.17	ENSP00000497154.1	5.8	1134
AQILTVKV	8	24.0714	LRRK2	ENSG00000188906.16	ENSP00000298910.7	23.4	7581
EYLLVPSSL	9	0.1273	LRRK2	ENSG00000188906.16	ENSP00000298910.7	23.4	7581
IYGEGFQTW	9	0.0041	ALG9	ENSG00000086848.15	ENSP00000482437.1	7.1	1854
RVQDKYFHI	9	0.8341	ABLIM2	ENSG00000163995.20	ENSP00000393511.2	5.2	1935
SFSTVHEKF	9	0.0219	WDR36	ENSG00000134987.11	ENSP00000424628.3	4.9	2685
AYGNVFSAF	9	0.0209	WDR36	ENSG00000134987.11	ENSP00000424628.3	4.9	2685
VQTAILRV	8	21.6818	PPP1R13B	ENSG00000088808.17	ENSP00000202556.9	25.0	3270
IFHEVPLKF	9	0.0046	ICE2	ENSG00000128915.12	ENSP00000261520.4	8.0	2946
RYPDTIALTF	10	0.0026	MAPKBP1	ENSG00000137802.14	ENSP00000426154.1	7.1	3693
VYNDHSIYVW	10	0.0502	MAPKBP1	ENSG00000137802.14	ENSP00000426154.1	7.1	3693
ELIKISEGTGFR	12	53.7500	DDX52	ENSG00000278053.5	ENSP00000479504.1	9.4	1797
AYLTVHQRI	9	0.0237	ZNF570	ENSG00000171827.10	ENSP00000331540.1	2.5	1608
IYQDVTLKW	9	0.0040	BBS9	ENSG00000122507.20	ENSP00000242067.6	8.7	2661
KFVNKSYLL	9	0.1769	SHQ1	ENSG00000144736.14	ENSP00000315182.8	5.9	1731
KYELDFSHF	9	0.0484	TLR2	ENSG00000137462.8	ENSP00000494425.1	3.1	2352
RYMNHMQSL	9	0.0187	ARIH1	ENSG00000166233.15	ENSP00000369217.4	10.9	1671
RYFDGNLEKL	10	0.0944	ARIH1	ENSG00000166233.15	ENSP00000369217.4	10.9	1671
VYSGGVYLF	9	0.0015	TMEM161A	ENSG00000064545.14	ENSP00000162044.7	18.8	1437
YFINRSWEW	9	0.0796	FAR2	ENSG00000064763.11	ENSP00000443291.2	2.6	1545
EVVGVYGSF	9	3.4547	DGKE	ENSG00000153933.10	ENSP00000284061.3	2.8	1701
VMPKKVFKI	9	0.1848	XYLT1	ENSG00000103489.11	ENSP00000261381.6	1.5	2877
RYLPRLHLV	9	0.0182	MGA	ENSG00000174197.16	ENSP00000457035.1	6.8	9195

EVIGLLGGR	9	42.0000	MYSM1	ENSG00000162601.11	ENSP00000478391.1	5.7	702
EVIGLLGGRY	10	39.0000	MYSM1	ENSG00000162601.11	ENSP00000478391.1	5.7	702
EVIEMVRKR	9	23.6875	ROR1	ENSG00000185483.12	ENSP00000441637.2	2.7	2646
NQVILRTV	8	20.9259	DDX60L	ENSG00000181381.13	ENSP00000260184.7	3.8	5118
LYIGHLTTL	9	0.0267	ERC1	ENSG00000082805.20	ENSP00000354158.3	10.0	3348
VYILQPEVI	9	0.0364	CPLANE1	ENSG00000197603.14	ENSP00000498265.1	5.9	6852
HYKPTPLYF	9	0.0037	SMC4	ENSG00000113810.16	ENSP00000417964.1	7.5	3789
RYFEVGAPVYF	11	0.0147	NPC1L1	ENSG00000015520.14	ENSP00000370552.3	1.7	3996
AYALLLQHL	9	0.0246	SLC26A6	ENSG00000225697.13	ENSP00000401813.1	32.0	540
RYNPNLNTW	9	0.0060	KLHL11	ENSG00000178502.6	ENSP00000314608.3	1.4	2124
VYNPELDKW	9	0.0360	KLHL11	ENSG00000178502.6	ENSP00000314608.3	1.4	2124
YVSRPSHF	8	1.0365	STAT2	ENSG00000170581.14	ENSP00000450751.1	25.6	2541
VYVSRPSHF	9	0.0058	STAT2	ENSG00000170581.14	ENSP00000450751.1	25.6	2541
AYVEDTVLF	9	0.0089	ARHGEF26	ENSG00000114790.13	ENSP00000424446.1	2.3	2373
RYPGKFYRV	9	0.0511	MAP3K20	ENSG00000091436.17	ENSP00000364361.3	8.2	2400
KYTHFIQSF	9	0.0021	GBGT1	ENSG00000148288.13	ENSP00000361110.3	5.0	1041
ETISQHIAKR	10	41.5000	GBGT1	ENSG00000148288.13	ENSP00000361110.3	5.0	1041
KYVTVYNLI	9	0.0238	USP8	ENSG00000138592.14	ENSP00000379721.3	23.8	3354
KYLADLPTL	9	0.0317	CEP85L	ENSG00000111860.14	ENSP00000357477.3	1.5	2415
SYMESVVTF	9	0.0008	BCAS3	ENSG00000141376.22	ENSP00000466078.1	21.4	2784
EYMAQHLVF	9	0.0233	STRIP1	ENSG00000143093.15	ENSP00000358811.1	20.4	2226
VWSDVTPLTF	10	0.0205	MMP11	ENSG00000099953.10	ENSP00000215743.3	5.2	1464
YFFEEKQPLQF	11	0.1048	CPNE6	ENSG00000100884.9	ENSP00000453352.1	16.4	678
VYLDGIVRI	9	0.0109	KIAA0895L	ENSG00000196123.13	ENSP00000290881.7	18.7	1413
SYIHYVFRL	9	0.0262	DOCK7	ENSG00000116641.17	ENSP00000340742.5	12.6	6327
RYLAINHAYF	10	0.0272	PTGER4	ENSG00000171522.6	ENSP00000302846.3	15.9	1464
KYVKVFDKF	9	0.0034	ZNF107	ENSG00000196247.11	ENSP00000378789.1	2.0	2349

AYNPVTHQL	9	0.0042	CDON	ENSG00000064309.14	ENSP00000436755.1	3.5	1950
RYAAQLPAL	9	0.1300	PELP1	ENSG00000141456.15	ENSP00000460300.2	13.0	3390
DIIAKVTRR	9	26.2000	ARHGAP27	ENSG00000159314.11	ENSP00000432762.1	19.9	2001
TYFTSGETF	9	0.0060	PCLO	ENSG00000186472.20	ENSP00000388393.2	4.8	14805
RLPSETYFF	9	0.0783	ZBED6	ENSG00000257315.2	ENSP00000447879.1	1.2	2937
KYGDILHVI	9	0.0077	DLG2	ENSG00000150672.17	ENSP00000381355.2	3.9	2610
VYGWTQHKF	9	0.0024	TMEM116	ENSG00000198270.13	ENSP00000347620.2	22.8	906
RYGPVVSF	8	0.0385	CYP20A1	ENSG00000119004.15	ENSP00000407860.2	10.7	1410
LYEEINQVF	9	0.0325	CYP20A1	ENSG00000119004.15	ENSP00000407860.2	10.7	1410
RYGPVVSFW	9	0.0056	CYP20A1	ENSG00000119004.15	ENSP00000407860.2	10.7	1410
RYTRWINQL	9	0.0280	B3GNTL1	ENSG00000175711.8	ENSP00000319979.3	3.5	1083
KYGNFIDKL	9	0.0902	GTPBP10	ENSG00000105793.15	ENSP00000222511.6	3.4	1161
FYIHHPVHL	9	0.0158	ALMS1	ENSG00000116127.18	ENSP00000479094.1	4.6	11577
RYMPPAHRNF	10	0.0021	IDO1	ENSG00000131203.13	ENSP00000430950.1	1.8	1209
VYSLVTRTW	9	0.0205	TRPM4	ENSG00000130529.16	ENSP00000407492.1	28.2	3207
IYDTSTDRLLW	11	0.2313	ISG20	ENSG00000172183.15	ENSP00000453638.1	2.7	543
VYLDHAGATLF	11	0.0119	MOCOS	ENSG00000075643.6	ENSP00000261326.4	1.2	2664
LYRLQFNEF	9	0.1323	ARL4A	ENSG00000122644.13	ENSP00000385236.1	2.8	600
SYSQSSNLF	9	0.0098	ZNF24	ENSG00000172466.16	ENSP00000382015.2	3.6	1104
GYKAIHEYL	9	0.2256	SYNE1	ENSG00000131018.23	ENSP00000356224.5	53.8	26391
MYLDAVHEF	9	0.0010	SYNE1	ENSG00000131018.23	ENSP00000356224.5	53.8	26391
DYTSRFSEF	9	0.1275	SYNE1	ENSG00000131018.23	ENSP00000356224.5	53.8	26391
SYLDRTEQL	9	0.0233	WDR18	ENSG00000065268.10	ENSP00000476117.2	57.8	1296
RYTPTTTQL	9	0.0126	UNC5D	ENSG00000156687.11	ENSP00000385143.2	1.7	2859
KYLTVKDYL	9	0.0991	ATAD2	ENSG00000156802.13	ENSP00000287394.5	1.6	4170
VYVPHIHVW	9	0.0021	ATAD2	ENSG00000156802.13	ENSP00000287394.5	1.6	4170
AYAIIKEEL	9	0.1534	ATAD2	ENSG00000156802.13	ENSP00000287394.5	1.6	4170

KYVKVFHKL	9	0.0035	ZNF493	ENSG00000196268.11	ENSP00000376110.2	4.5	2322
NYTLVSHLI	9	0.0455	MARS	ENSG00000166986.15	ENSP00000262027.5	44.9	2700
RWMDQRLVF	9	0.0754	GABRP	ENSG00000094755.17	ENSP00000265294.4	2.0	1320
KYIKEAVRL	9	0.0236	FRMD6	ENSG00000139926.15	ENSP00000379068.2	5.3	1842
VYMELSQKL	9	0.0043	FRMD6	ENSG00000139926.15	ENSP00000379068.2	5.3	1842
LYQKDFLRI	9	0.0559	FRMD6	ENSG00000139926.15	ENSP00000379068.2	5.3	1842
RAARYYYYW	9	1.0602	FRMD6	ENSG00000139926.15	ENSP00000379068.2	5.3	1842
RYMSHKLKI	9	0.0478	GBP3	ENSG00000117226.12	ENSP00000359512.4	20.4	1785
FYSRLLQKF	9	0.0046	ZWILCH	ENSG00000174442.12	ENSP00000311429.5	2.6	1773
PFIKSQHELLF	11	0.3613	ZWILCH	ENSG00000174442.12	ENSP00000311429.5	2.6	1773
VYQKIHNEKF	10	0.0195	ZNF529	ENSG00000186020.13	ENSP00000334695.7	3.0	1374
SYIQRLVQI	9	0.0142	SAMHD1	ENSG00000101347.9	ENSP00000494979.1	7.2	381
SYLTQHQRI	9	0.0377	AC092835.1	ENSG00000233757.6	ENSP00000478941.1	1.3	1566
VYPERTPLL	9	0.0092	AL669918.1	ENSG00000250264.1	ENSP00000391806.2	0.8	2529
KYLLTPVNF	9	0.0028	DCLRE1B	ENSG00000118655.7	ENSP00000498042.1	2.0	1596
GYFGNPQKF	9	0.0074	LAMA3	ENSG00000053747.16	ENSP00000324532.8	2.1	9999
IYKGIPLQL	9	0.0139	USP6NL	ENSG00000148429.14	ENSP00000476462.1	3.1	2484
EIYHGSTKR	9	22.0000	DNAJB5	ENSG00000137094.14	ENSP00000312517.5	2.7	1044
VYSDYLQTI	9	0.0057	PCED1B	ENSG00000179715.13	ENSP00000448000.1	1.9	501
KWFDNSNMTF	10	0.0348	CD302	ENSG00000241399.7	ENSP00000451446.2	7.9	585
SFITHSLKF	9	0.0430	CEP192	ENSG00000101639.18	ENSP00000427550.1	5.5	7611
HYINMPVQF	9	0.0032	CEP192	ENSG00000101639.18	ENSP00000427550.1	5.5	7611
SYLKSANSW	9	0.0214	KIAA0513	ENSG00000135709.12	ENSP00000446439.1	1.4	1203
IYPPKLHQF	9	0.0012	CCNE1	ENSG00000105173.14	ENSP00000262643.3	1.6	1230
VFKEKHHSW	9	0.1530	RNASEH2B	ENSG00000136104.20	ENSP00000494019.1	7.7	687
TYSKKHFRI	9	0.0510	CHEK2	ENSG00000183765.22	ENSP00000384835.1	3.5	1542
VYTSKLVRL	9	0.0291	PLXNA4	ENSG00000221866.9	ENSP00000323194.4	0.8	5682

RYVDRVTEF	9	0.0042	CDK5RAP3	ENSG00000108465.15	ENSP00000464286.1	19.1	816
RYTSQFIRL	9	0.0233	DCAF17	ENSG00000115827.14	ENSP00000400335.1	7.4	384
YYEHVKARF	9	0.0315	MLPH	ENSG00000115648.14	ENSP00000386780.1	4.6	1440
DIIEKVMAR	9	19.8065	TSC2	ENSG00000103197.18	ENSP00000494514.1	58.4	5289
IYPSKWIARL	10	0.1874	TSC2	ENSG00000103197.18	ENSP00000494514.1	58.4	5289
KYMKIFHKF	9	0.0004	ZNF681	ENSG00000196172.9	ENSP00000384000.3	0.7	1935
VYGPVFTL	8	0.1231	CYP2C9	ENSG00000138109.11	ENSP00000260682.6	1.4	1470
KYVKVMHKF	9	0.0016	ZNF138	ENSG00000197008.9	ENSP00000399528.1	5.8	861
YSHVIQKL	8	1.0537	SDS	ENSG00000135094.11	ENSP00000257549.4	0.6	984
VYSHVIQKL	9	0.0033	SDS	ENSG00000135094.11	ENSP00000257549.4	0.6	984
EYKKDFEKW	9	0.1576	NEB	ENSG00000183091.19	ENSP00000484342.1	5.5	25680
EYKKEFEKW	9	0.1379	NEB	ENSG00000183091.19	ENSP00000484342.1	5.5	25680
RYLTVATVF	9	0.0105	TUBB3	ENSG00000258947.7	ENSP00000320295.7	1.1	1350
DVAQEHLKER	10	53.7500	GLI2	ENSG00000074047.21	ENSP00000390436.1	1.1	4758
EVFTGGIGSY	10	15.8116	TENT4B	ENSG00000121274.12	ENSP00000350054.4	9.2	909
HGVILPRV	8	31.6667	MTMR10	ENSG00000166912.17	ENSP00000402537.1	31.4	2331
NYAQVLDKF	9	0.0094	ASAP1	ENSG00000153317.15	ENSP00000429900.1	9.9	3387
SYGTVSQIF	9	0.0060	CLEC5A	ENSG00000258227.7	ENSP00000449999.1	0.5	564
TFESKSYKM	9	1.1172	FGA	ENSG00000171560.16	ENSP00000385981.3	0.4	1932
FLAEGGGVR	9	36.3333	FGA	ENSG00000171560.16	ENSP00000385981.3	0.4	1932
KYVKDFHKF	9	0.0014	ZNF724	ENSG00000196081.9	ENSP00000413411.1	0.5	1857
QYIDKLNEL	9	0.0257	CCDC88A	ENSG00000115355.16	ENSP00000405080.1	4.3	3606
KYLQSTISF	9	0.0031	TOP3B	ENSG00000100038.19	ENSP00000349705.5	12.9	2586
KYGVVIASF	9	0.0050	DOCK4	ENSG00000128512.22	ENSP00000404179.1	10.3	5898
SYQKVIELF	9	0.0009	PBK	ENSG00000168078.10	ENSP00000301905.4	0.3	966
EYNTIKDKF	9	0.0842	PARP15	ENSG00000173200.13	ENSP00000419488.1	0.6	1125
EYQKVWNLF	9	0.0077	PARP12	ENSG00000059378.12	ENSP00000263549.3	16.7	2103

KHNYELDFKAF	11	1.3328	PARP12	ENSG00000059378.12	ENSP00000263549.3	16.7	2103
KFSEPLYTF	9	0.0038	FAT2	ENSG00000086570.12	ENSP00000261800.5	0.3	13047
IYRFITERF	9	0.0202	FOXE3	ENSG00000186790.6	ENSP00000334472.2	0.3	957
TYLRKIPRF	9	0.0044	CCDC14	ENSG00000175455.15	ENSP00000386866.4	8.3	2715
YADPSILYI	9	2.2250	HDAC9	ENSG00000048052.21	ENSP00000384657.3	4.0	3198
VAVAGVVLAKG	11	90.0000	SLC10A6	ENSG00000145283.7	ENSP00000273905.6	0.2	1131
LYRDIFQHL	9	0.0486	NFE2	ENSG00000123405.14	ENSP00000447558.1	0.3	1119
EAQVERAM	8	45.5000	KIF18B	ENSG00000186185.13	ENSP00000465377.1	0.2	2499
TYPEGLEVLHF	11	0.0123	CENPJ	ENSG00000151849.15	ENSP00000371308.4	2.5	4014
KYGFYTHVF	9	0.0058	F2	ENSG00000180210.14	ENSP00000308541.5	0.2	1866
ESLLLLPR	8	65.0000	DIPK2A	ENSG00000181744.8	ENSP00000419947.2	5.1	639
SYLESLVKF	9	0.0018	HSD17B13	ENSG00000170509.12	ENSP00000333300.4	0.1	900
AVADMLRENRS	11	70.0000	TMOD4	ENSG00000163157.15	ENSP00000295314.4	0.2	1035
QQYRALSV	8	22.7895	TUBB1	ENSG00000101162.3	ENSP00000217133.1	0.1	1353

%rank, %rank score of NetMHCpan4.1; Expression, gene expression calculated using RNA-seq data; ORF length, nucleotide lengths of the open reading frame encoding the peptide
